# Prognostic and immunological roles of ammonia-induced cell death-related genes in non-small cell lung cancer

**DOI:** 10.1186/s12890-026-04181-7

**Published:** 2026-02-21

**Authors:** Hongbin Li, Kai Xue, Jianying Pei, Xiaoli Ma, Xueshan Zhao, Chong Zhang

**Affiliations:** 1 School of Life Science and Engineering, Lanzhou University of Technology, No.36 Pengjiaping Road, Lanzhou, Gansu Province 730050 China; 2 Department of Clinical Laboratory center, Gansu Provincial Maternity and Child-Care Hospital (Gansu Provincial Central Hospital), Lanzhou, 730050 China; 3 Affiliated Hospital of Qinghai University & Affiliated Cancer Hospital of Qinghai University, Xining, 810000 China

**Keywords:** Ammonia-induced cell death, Non-small cell lung cancer, Tumor microenvironment, T cells, SLC7A5, SLC2A1, CAV1, SPP1

## Abstract

**Background:**

Lung cancer remains the predominant cause of cancer-related mortality globally, with non-small cell lung cancer (NSCLC) comprising approximately 85% of cases, despite advancements in immunotherapy. This challenge is primarily due to the persistent dysfunction of effector T cells within the tumor microenvironment (TME). Ammonia-induced cell death (AICD), a newly identified form of programmed cell death, leads to the attrition of CD8⁺ T cells and contributes to immune suppression. However, the clinical significance of AICD in NSCLC has yet to be elucidated. In light of this context, the current study seeks to investigate AICD-related genes and their prognostic and immunological implications in NSCLC.

**Methods:**

Transcriptomic data from The Cancer Genome Atlas (TCGA) and Gene Expression Omnibus (GEO) cohorts were integrated with single-cell RNA sequencing and functional assays. Glutamine metabolism–related genes were retrieved from GeneCards and intersected with NSCLC differentially expressed genes to identify candidate AICD regulators. Prognostic value was evaluated by Kaplan–Meier survival and ROC analyses. Single-cell and immune infiltration analyses were used to define gene expression across malignant and immune subsets. Finally, Jurkat T cells were exposed to ammonia stress to validate gene function in AICD.

**Results:**

Four genes—SLC7A5, SLC2A1, CAV1, and SPP1—emerged as key regulators of AICD in NSCLC. These genes were aberrantly expressed in tumors, stratified patient survival, and correlated with immune checkpoint activity and TME immunosuppression. A gene signature derived from the four regulators effectively predicted overall survival and immune response. Single-cell analysis confirmed their distribution across malignant and immune subpopulations. Functional assays demonstrated that CAV1, SPP1, and SLC7A5 protected against ammonia-induced T cell death, whereas SLC2A1 increased susceptibility.

**Conclusion:**

This study establishes AICD as a clinically relevant metabolic cell death mechanism in NSCLC and identifies SLC7A5, SLC2A1, CAV1, and SPP1 as potential prognostic biomarkers and therapeutic targets. Targeting ammonia metabolism may offer new strategies to overcome immune resistance and enhance immunotherapy efficacy in NSCLC.

**Supplementary Information:**

The online version contains supplementary material available at 10.1186/s12890-026-04181-7.

## Introduction

Lung cancer remains the leading cause of cancer-related mortality worldwide, and non-small cell Lung cancer (NSCLC) accounts for approximately 85% of all cases [1]. The advent of immunotherapy, particularly immune checkpoint inhibitors, has improved outcomes for some NSCLC patients. However, many patients derive only limited benefit, as the efficacy of immunotherapy is often blunted by an immunosuppressive tumor microenvironment and T cell dysfunction [2, 3]. Tumor-infiltrating T cells, especially effector CD8⁺ T lymphocytes, frequently become exhausted or undergo premature cell death in NSCLC, losing their antitumor activity despite therapy. While classical studies have attributed T cell attrition to continuous antigen stimulation and checkpoint-mediated exhaustion, emerging evidence suggests that metabolic factors within the tumor microenvironment also critically impair T cell survival [4]. 

Ammonia has recently been recognized as a key immunosuppressive metabolite in the tumor milieu. As a toxic byproduct of glutamine metabolism, ammonia can accumulate in solid tumors [5, 6]. Analogous to lactate and kynurenine, elevated ammonia in the TME creates a biochemical barrier to effective immunity [4]. In NSCLC and other cancers, excess ammonia contributes to chronic inflammation, T cell exhaustion, and loss of effector function, ultimately fostering an immunosuppressive milieu that enables tumor progression [4, 7]. Zhang et al. recently uncovered a mechanism by which ammonia directly induces T cell death [8, 9]. In activated CD8⁺ T cells, glutaminolysis-driven ammonia accumulates in lysosomes, raising their pH and causing ammonia to reflux into mitochondria; this cascade provokes lysosomal and mitochondrial damage and culminates in T cell apoptosis [8]. Notably, inhibiting glutamine metabolism or preventing lysosomal alkalinization can rescue T cells from ammonia-induced death and improve T cell-mediated antitumor immunity [8]. This form of cell demise, termed ammonia-induced cell death (AICD), is biochemically distinct from traditional apoptotic or exhaustion pathways and offers a metabolic explanation for the rapid loss of effector T cells after they exert anti-tumor effects [8, 9]. The identification of AICD points to an underappreciated metabolic checkpoint in cancer immunology, suggesting that ammonia accumulation is not merely a metabolic byproduct but also a driver of immune escape [4, 8–10].

These insights show that genes involved in ammonia production, detoxification, or the AICD pathway could have prognostic and immunological significance in NSCLC. Here, we define ammonia-induced cell death-related genes (ADRGs) as those participating in glutamine metabolism pathways that promote ammonia accumulation and consequent organelle damage in T cells. ADRGs are directly linked to glutaminolysis, wherein excess ammonia—a byproduct of glutamine breakdown—drives AICD through lysosomal alkalinization and mitochondrial reflux. In other malignancies, ammonia-related programs have been linked to T cell exhaustion, adverse outcomes, and reduced response to checkpoint blockade [4, 11–13]. However, no systematic study has investigated the clinical relevance of ADRGs in NSCLC.

In this study, we present a comprehensive investigation of ADRGs in NSCLC, integrating large-scale bioinformatics with experimental validation. Specifically, we identified and validated four key ADRGs—SPP1, SLC7A5, SLC2A1, and CAV1—that play roles in metabolic reprogramming, nutrient transport, and lysosomal–mitochondrial integrity. Prior work supports the functional relevance of these molecules in immune–metabolic control: SLC7A5 (LAT1) coordinates amino-acid uptake and T cell differentiation [14]; SLC2A1 (GLUT1) is essential for T cell activation and effector survival [15]; CAV1 intersects with membrane trafficking and autophagy pathways that influence organelle integrity [16]; and SPP1 (osteopontin) can restrain CD8⁺ T cell activation via CD44 and is emerging as a biomarker in NSCLC treated with pembrolizumab [17, 18]. Using transcriptomic datasets from NSCLC patient cohorts, we evaluated their association with survival outcomes and immune-microenvironmental features. Functional assays further confirmed their involvement in regulating cellular susceptibility to ammonia toxicity and shaping T cell activity. Through this integrative approach, our study establishes the prognostic and immunological implications of ADRGs in NSCLC. We propose that SPP1, SLC7A5, SLC2A1, and CAV1 may serve not only as biomarkers of immune landscape and prognosis, but also as potential therapeutic targets for mitigating AICD and enhancing immunotherapy efficacy in lung cancer.

## Methods

### Data acquisition and processing

NSCLC RNA-seq data and clinical information were obtained from The Cancer Genome Atlas (TCGA) (Supplementary Table 1) and Gene Expression Omnibus (GEO) [19]. Given the link between glutamine metabolism and AICD, glutamine-related genes (GeneCards score ≥ 6.2; Supplementary Table 2) were defined as ADRGs. This threshold reflects the mean relevance score (6.2) as a cutoff within GeneCards. Differentially expressed genes (DEGs) were determined using R "limma" (|log₂FC|≥ 2, adj P < 0.05), visualized using "ggplot2", and intersected with ADRGs using "VennDiagram" [20]. TPM-normalized expression was compared between tumor and normal tissues using the Wilcoxon test (R v4.0.3), with GTEx (V8) validating normal tissue expression.

### Identification of molecular subtypes

Through analysis of the TCGA transcriptomic dataset, 30 ADRGs exhibiting differential expression patterns were identified. Subsequent consensus clustering analysis was conducted utilizing these molecular markers with the "ConsensusClusterPlus" software package (version 1.54.0) implemented in the R statistical environment [21].

### Identification and enrichment analysis of DEGs

Differential expression analysis between C1/C2 subtypes was performed using the R package “Limma” v3.40.2 (|log₂FC|> 1, adj P < 0.05) [20]. Hierarchical clustering for gene expression profiles was performed using the “heatmap” package. DEG functional enrichment (Gene Ontology/Kyoto Encyclopedia of Genes and Genomes) was analyzed via R “clusterProfiler” v3.18.0 [22]. Potential biological pathways were identified via Gene Set Enrichment Analysis (GSEA) on the Broad Institute portal (https://software.broadinstitute.org/gsea/index.jsp), with 10,000 gene set permutations per analysis; significant pathways met adj *P <* 0.05 and FDR < 0.25 [23].

### Analysis of genetic alterations

Using the Gene Set Cancer Analysis (GSCA) platform (https://guolab.wchscu.cn/GSCA) [24], we conducted extensive genomic analysis of ADRGs in NSCLC. This included systematic evaluation of diverse mutational patterns: single-nucleotide variations (amino acid substitutions, premature termination codons), insertion-induced “frameshift” disruptions, exon–intron boundary alterations, deletion-mediated “frameshifts”, and complex genomic aberrations.

### Development and validation of a prognostic signature based on ADRGs

Kaplan–Meier survival analysis and univariate Cox models were used to identify prognostic ADRGs. A predictive signature was built via LASSO-Cox regression. Its discrimination was evaluated using the R package “timeROC”. External validation used GEO cohorts (GSE19188, GSE18842).

### Immune cell infiltration and immune therapy response analysis

For C1/C2 immune microenvironment analysis, we used the immunedeconv R package and TIMER2.0 platform (to analyze prognostic ADRGs related to immunity). ssGSEA (GSVA) was used to quantify immune infiltration; the Wilcoxon test was used to compare ADRG expression, and Spearman was used to assess immune ADRGs.

### Building a predictive nomogram

Demographic/clinicopathological data (age, sex, T/N/M, stage, smoking) and risk scores were from TCGA. Risk score correlated with clinical parameters for prognosis. Nomogram (univariate/multivariate Cox, results as HRs, 95% CIs, p-values) and 1-/3-/5-year overall survival (OS) predicted via R’s “rms” package; validated by calibration/ROC curves, DCA.

### Expression analysis of immune checkpoint-related genes

The expression profiles of key immune checkpoint molecules (CD274, CTLA4, HAVCR2, LAG3, PDCD1, PDCD1LG2, TIGIT, SIGLEC15, ITPRIPL1, IGSF8) were systematically examined, with predictions of response to immune checkpoint blockade generated using the TIDE computational framework [25]. Furthermore, the therapeutic implications of ADRGs in immune checkpoint inhibition were comprehensively assessed by analyzing TCGA cohort data.

### scRNA-Seq data analysis

scRNA-seq data (.h5) and annotation files were obtained from TISCH2.0. Using the R package “MAESTRO/Seurat”, “t-SNE” was applied for dimensionality reduction and clustering. ADRGs' expression in TME cell subsets was analyzed, with t-SNE plots and heatmaps (NSCLC_GSE179373/GSE99254) illustrating patterns.

### Pan-RNA epigenetic modification-related gene selection

A comprehensive analysis was performed using the GSCA platform to investigate the relationship between DNA methylation patterns of prognostically significant ADRGs and their transcriptional regulation in NSCLC. Statistical significance was determined through the Wilcoxon rank-sum test, and graphical outputs were generated using the ggplot2 package implemented in R software (version 4.0.3).

### Cell culture

The Jurkat clone E6-1 cell line (Servicebio, China) used in this study was authenticated by short tandem repeat profiling and confirmed to be mycoplasma-free before experiments. Jurkat cells were cultured in RPMI-1640 medium (Sangon Biotech, China, E600028) supplemented with 10% fetal bovine serum (Sangon Biotech, China, E600001). Cultures were maintained at 37 °C in a humidified incubator with 5% CO₂ for optimal growth.

### Plasmid and siRNA transfection

Overexpression plasmids for SPP1, SLC2A1, SLC7A5, and CAV1 were purchased from Miaolingbio. Plasmid transfection was performed using Lipo8000 (Beyotime, China, C0533), while siRNA transfection was carried out using LipoRNAi (Beyotime, China, C0535). After a 4-h incubation, the culture medium was replaced with fresh complete medium. Cells were harvested 48 h post-transfection for subsequent analyses. The siRNA sequences were presented in Supplementary Table 3.

### RT-qPCR

Total RNA was extracted with RNAeasy™ Kit (Beyotime Biotechnology, China, R0026) per instructions. Reverse transcription was performed using Takara PrimeScript Kit (Takara, Japan, RR036A). Target gene expression was analyzed via Takara TB Green® Premix (Takara, Japan, RR820A) on the QuantGene 9600 system (Bioer, China, FQD-96C). Target gene expression was quantified by the 2^−ΔΔCt^ method, normalized to GAPDH. The primer sequences used for real-time PCR are listed in Supplementary Table 3.

### Immunoblotting

Proteins were extracted using a chilled lysis buffer with inhibitors, and concentrations were determined with a BCA kit (Servicebio, China, G2026-200 T). Samples were dissolved in loading buffer, denatured, separated by SDS-PAGE, and transferred to PVDF membranes. Membranes were blocked with milk, then incubated overnight at 4 °C with primary antibodies for SLC2A1 (1:1000, Proteintech, China, 66290–1-Ig), SPP1 (1:1000, Proteintech, China, 83341–1-RR), SLC7A5 (1:1000, Selleck, China, F3212), CAV1 (1:1000, Proteintech, China, 16447–1-AP), and GAPDH (1:5000, Proteintech, China, 60004–1-Ig). After washing, HRP secondary antibodies were applied for 1 h at room temperature, and signals were detected using a Bio-Rad ChemiDoc with ECL substrate (Biosharp, China; BB520A).

### Immunofluorescence staining

Cells were collected by centrifugation and washed twice with PBS. The cell pellet was resuspended in pre-chilled methanol and fixed at 4℃ for 30 min. After centrifugation, cells were resuspended in PBS and dropped onto glass slides. Following air-drying, cells were additionally fixed with 4% paraformaldehyde for 10 min at room temperature and permeabilized with 0.1% Triton X-100. Nonspecific binding was blocked using 5% bovine serum albumin (BSA) for 1 h at room temperature. Cells were then incubated with primary antibodies at 4℃ overnight. After three washes with PBS, cells were incubated with the corresponding secondary antibodies for 1 h at room temperature. Nuclei were counterstained with DAPI (Beyotime, China, C1005) for 10 min, followed by three additional PBS washes. Finally, samples were mounted with antifade mounting medium (Beyotime, China, P0126) and imaged using a confocal laser scanning microscope.

### Cell proliferation assay

 Jurkat cells were seeded at a density of 10,000 cells per well in a 96-well plate and incubated overnight. Treat cells with different concentrations of NH₄Cl for 24 h to assess their inhibitory effects. CCK-8 solution was added to each well to achieve a final concentration of 10%. The plate was incubated at 37 °C for 2 hours. After incubation, measure the absorbance at 450 nm.

### Measurement of mitochondrial DNA copy number

The mitochondrial DNA (mtDNA) copy number was quantified via Human mtDNA Monitoring Primer Set (Takara, Japan, C7246) per instructions. qPCR amplified mitochondrial genes (ND1, ND5) and nuclear reference genes (SLCO2B1, SERPINA1). Ct values gave ΔCt₁ = Ct (SLCO2B1) – Ct (ND1), ΔCt₂ = Ct (SERPINA1) – Ct (ND5); Relative mtDNA copy number used the 2^ΔCt^ method (2^ΔCt₁^, 2^ΔCt₂^), final value as their average (normalized to nuclear DNA).

### Quantification of lysosomal pH

Lysosomal pH was assessed using the ratiometric dye LysoSensor Yellow/Blue DND-160. Cells were incubated with 2 μM dye in pre-warmed medium at 37 °C for 5 min, followed by two PBS washes. Fluorescence was then recorded on a microplate reader (TECAN, Switzerland, Infinite® 200 PRO) with excitation at 360 nm and emission collected at 440 nm and 550 nm.

### Statistical analysis

All statistical analyses were performed using R 4.2.1. Two-group comparisons used Student’s t-test/Wilcoxon rank-sum test per data distribution; paired variable correlations used Pearson/Spearman as needed. Survival analysis used univariate/multivariate Cox models. Datasets, R packages, and bioinformatics resources were customized per section; *P <* 0.05 was considered indicative of statistical significance.

## Results

### Identification of ADRGs and expression dysregulation in NSCLC

**Fig. 1:**
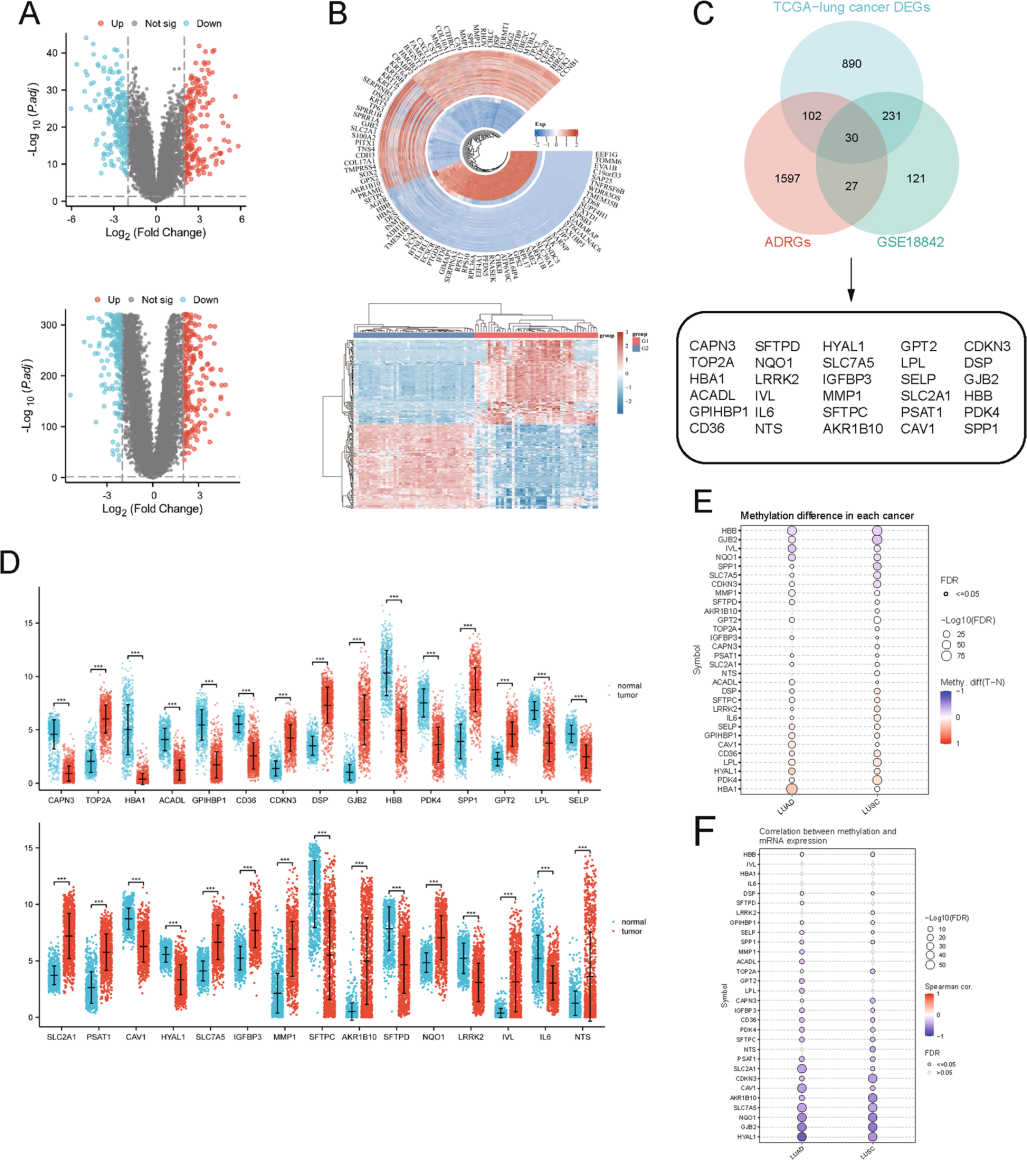
Identification of ammonia-induced cell death-related genes (ADRGs) in NSCLC. **A** Volcano plots of DEGs in TCGA-NSCLC and GSE18842 datasets, with upregulated and downregulated genes marked in red and blue, respectively (adjusted *P <* 0.05, |log2FC|> 2). **B** Heatmap of differentially expressed genes (DEGs) in TCGA-NSCLC and GSE18842 datasets, with red indicating upregulation and blue indicating downregulation. **C** Venn diagram illustrating the overlap among differentially expressed genes (DEGs) from TCGA-NSCLC, GSE18842, and predefined ADRGs, yielding 30 candidate genes. **D** Dot plot depicting expression levels of 30 ADRGs in tumor (red) versus normal (cyan) tissues (*P <* 0.001). **E** Bubble chart of methylation differences (tumor and normal) for 30 ADRGs in LUAD and LUSC, with FDR ≤ 0.05 indicated. **F** Bubble chart showing Spearman correlations between methylation and mRNA expression for 30 ADRGs in LUAD and LUSC (FDR ≤ 0.05)

Volcano plots and heatmaps were used to visualize differential gene expression in the TCGA-NSCLC and GSE18842 datasets (Fig. 1A, B). From GeneCards, we retrieved 1,756 glutamine metabolism-related genes. Intersecting these with the NSCLC tumor vs. normal DEGs yielded 30 overlapping ADRGs for further analysis (Fig. 1C).

Comparative analysis of these 30 ADRGs in NSCLC tumors versus normal lung tissue revealed widespread dysregulation. A subset of these genes—such as TOP2A, CDKN3, DSP, GJB2, SPP1, GPT2, SLC2A1, PSAT1, SLC7A5, IGFBP3, MMP1, AKR1B10, NQO1, IVL, and NTS—was significantly upregulated in tumors. Conversely, another subset (including CAPN3, HBA1, ACADL, GPIHBP1, CD36, HBB, PDK4, LPL, SELP, CAV1, HYAL1, SFTPC, SFTPD, LRRK2, and IL6) was notably downregulated in tumors (Fig. 1D).

DNA methylation patterns of these dysregulated genes were then examined. In both lung adenocarcinoma (LUAD) and lung squamous cell carcinoma (LUSC), many genes with altered expression also exhibited significant promoter methylation differences, suggesting a link between methylation status and expression changes (Fig. 1E, F). Such methylation–expression relationships may contribute to NSCLC pathogenesis and warrant further investigation of the underlying regulatory mechanisms. Additionally, UALCAN analysis quantified promoter methylation for four key ADRGs in tumors versus matched normal tissues (Supplementary Fig. 1).

### Identification of two NSCLC subtypes via ADRGs expression clustering

**Fig. 2:**
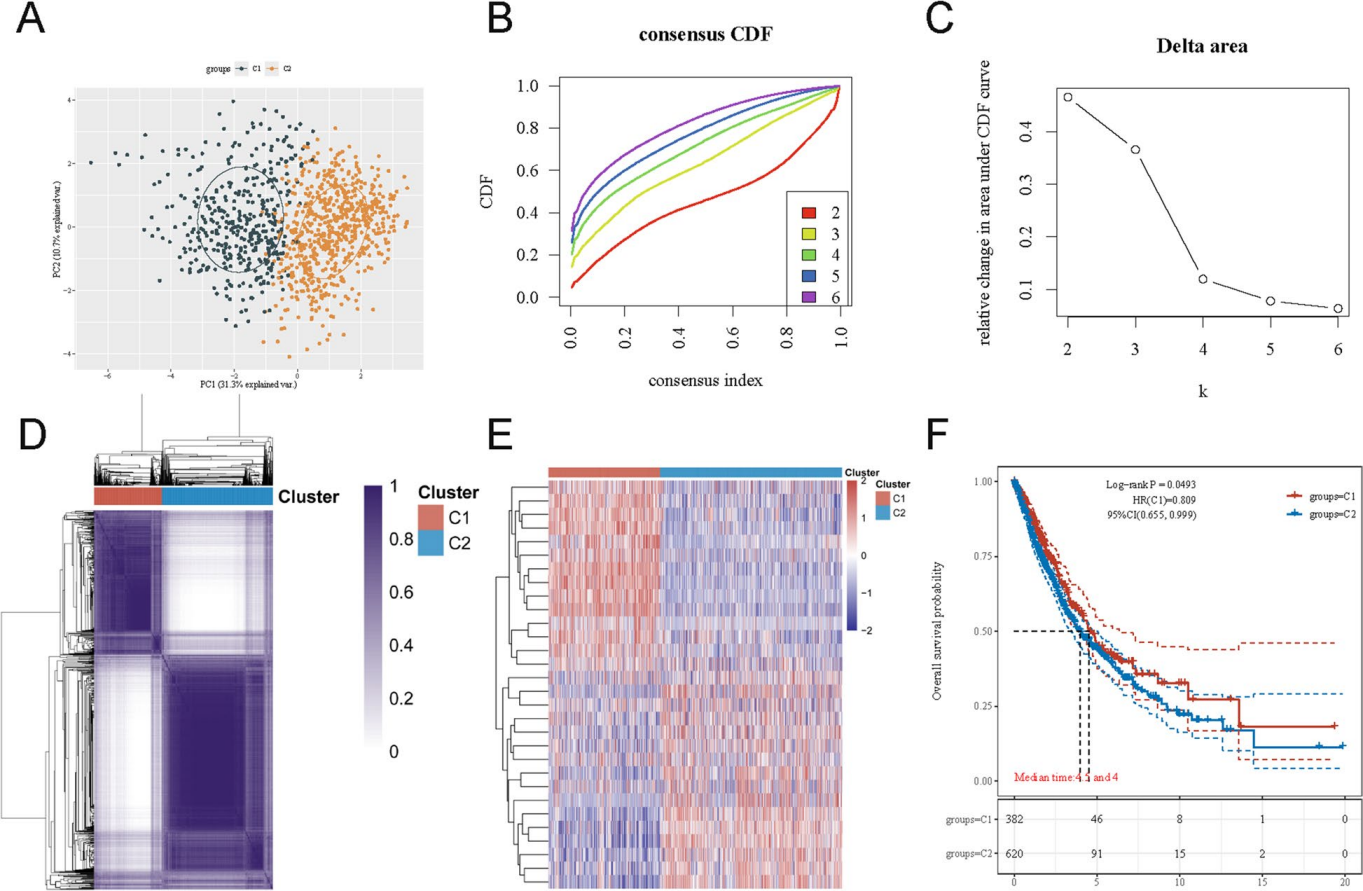
Consensus clustering of NSCLC samples based on ADRGs expression. **A** Principal component analysis (PCA) of transcriptomic profiles distinguishing two molecular subtypes (C1 and C2). **B** Cumulative distribution function (CDF) curves for consensus clustering with k = 2–6. **C** Delta area plot showing relative changes in CDF area under the curve for k = 2–6. **D** Consensus matrix heatmap for k = 2, illustrating sample clustering based on 30 ADRGs. **E** Heatmap of ADRGs expression in different subtypes, with red indicating high expression and blue indicating low expression. **F** Kaplan–Meier overall survival curves for C1 and C2 subtypes (log-rank *P =* 0.0493; HR = 0.809, 95% CI: 0.655–0.999)

Principal component analysis (PCA) of the TCGA NSCLC dataset revealed a clear separation between two molecular subtypes, labeled C1 and C2 (Fig. 2A). This finding indicates that these subgroups have markedly different transcriptional profiles. Consensus clustering analysis further supported k = 2 as the optimal number of clusters: the CDF curves showed the most pronounced inflection at k = 2 (Fig. 2B), and the CDF plot confirmed that dividing the samples into two clusters best captured the data structure (Fig. 2C). The consensus matrix for k = 2 (Fig. 2D) illustrates the stability of this classification, with distinct blocks corresponding to C1 and C2 (darker colors indicate higher sample similarity). These results demonstrate that the samples segregate into two groups based on ADRGs expression.

ADRGs expression patterns differed markedly between the C1 and C2 subtypes (Fig. 2E). Many ADRGs were differentially expressed (some highly expressed in C1 but low in C2, and vice versa), likely contributing to the biological and prognostic differences between the subtypes. Consistently, Kaplan–Meier analysis showed that patients in subtype C1 had significantly better overall survival than those in C2 (Fig. 2F). The C1 group exhibited a longer median survival and higher survival probabilities over time. Log-rank test and hazard ratio results further supported a survival advantage for C1 relative to C2.

### Differential gene expression and functional enrichment between NSCLC subtypes

**Fig. 3:**
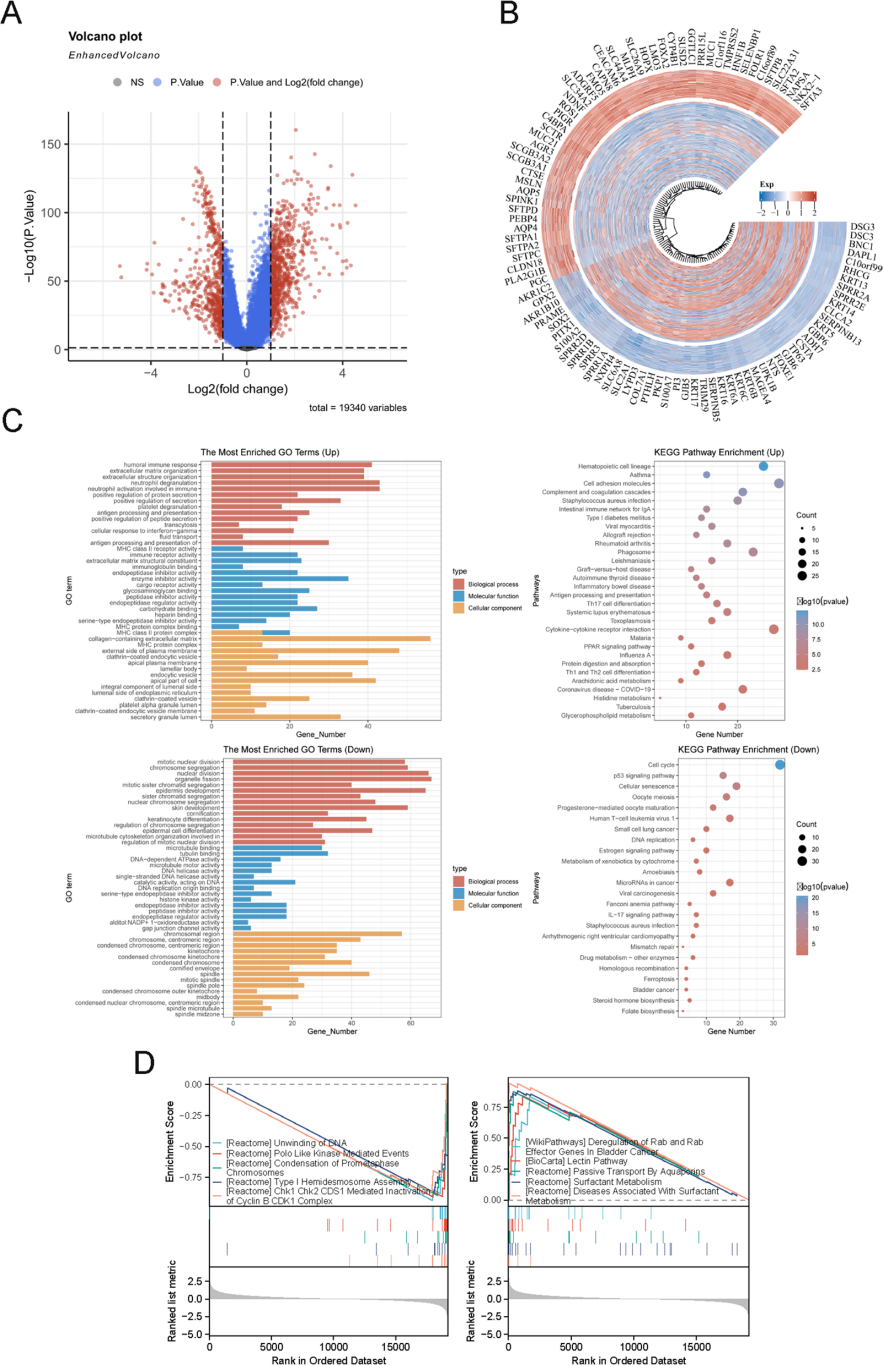
Differential gene expression and functional enrichment between NSCLC subtypes C1 and C2. **A** Volcano plot of DEGs between C1 and C2, with 789 upregulated (red) and 560 downregulated (blue) genes in C1 (adjusted *P <* 0.05, |log2FC|> 1). **B** Heatmap of top DEGs, clustered by subtype, with expression levels color-coded (red: high; blue: low). **C** Bar plots of Gene Ontology (GO) terms and Kyoto Encyclopedia of Genes and Genomes (KEGG) pathways enriched in upregulated (top) and downregulated (bottom) DEGs, highlighting immune processes (e.g., humoral immune response) and cell cycle pathways (e.g., p53 signaling), respectively. **D** Gene set enrichment analysis (GSEA) plots for top positively (e.g., surfactant metabolism) and negatively (e.g., DNA unwinding) enriched pathways in C1 versus C2 (*P <* 0.05, FDR-corrected q-value)

A total of 789 genes were upregulated and 560 were downregulated in C1 relative to C2 (Fig. 3A, B). Gene Ontology (GO) enrichment analysis revealed that genes upregulated in C1 were primarily involved in immune responses – for example, humoral immunity and neutrophil activation – as well as extracellular matrix organization (e.g., collagen, collagen-containing extracellular matrix, glycosaminoglycan binding). These findings suggest that subtype C1 is characterized by enhanced inflammatory activity, active tissue remodeling, and other immune-related pathways. In contrast, genes upregulated in C2 (i.e., downregulated in C1) were enriched in cell division and mitotic cell-cycle processes (such as nuclear division, organelle fission, microtubule binding), along with developmental pathways like epidermal development. This pattern implies that C2 has more active cell proliferation and potentially disrupted cell-cycle control. Consistently, KEGG pathway analysis showed that genes higher in C1 mapped to pathways such as hematopoietic cell lineage, asthma, cell adhesion molecules, and complement/coagulation cascades, whereas genes higher in C2 were enriched in pathways including cell cycle, p53 signaling, and cellular senescence (Fig. 3C).

GSEA further underscored the functional dichotomy between the subtypes, identifying 724 pathways with significant enrichment differences (*P <* 0.05). The top five pathways negatively enriched in C1 (relative to C2) were mainly cell cycle–related (e.g., DNA unwinding, PLK-mediated events).

Condensation of Prometaphase Chromosomes, indicating substantially higher mitotic activity in C2. Two additional pathways with lower enrichment in C1 – type I hemidesmosome assembly and CHK1/CHK2-mediated inactivation of Cyclin B–CDK1 – suggest that C2 also has more active cell–matrix adhesion processes and a relaxed DNA damage checkpoint (Fig. 3D). Conversely, the top positively enriched pathways in C1 (higher activity in C1 than C2) involved processes such as fluid homeostasis (e.g. surfactant metabolism), innate immune responses (e.g. lectin pathway), and certain tumor-related mechanisms (e.g. Deregulation of Rab and Rab Effector Genes In Bladder Cancer) (Fig. 3D). These findings provide pathway-level evidence that C1 exhibits distinct immunologic and homeostatic activity, while C2 is associated with heightened cell-cycle progression and proliferation. Together, these results delineate clear molecular and functional differences between the C1 and C2 subtypes, offering insight into their divergent biology.

### Somatic mutation and copy number alterations in ADRGs

Among 313 NSCLC samples, 240 (76.68%) harbored somatic alterations in at least one of 30 selected ADRGs (Supplementary Fig. 2A). The most frequently mutated genes were LRRK2 (mutated in 28% of samples), SELP (17%), DSP (17%), and TOP2A (6%). Observed mutations included missense, frameshift deletion, splice-site, and nonsense mutations, with distinct patterns for each gene. Copy number variation (CNV) analysis revealed differences between LUAD and LUSC: a pie chart (Supplementary Fig. 2B) shows the proportions of samples with gene amplifications or deletions in each subtype, highlighting distinct CNV profiles between these two histologies. Notably, genes like LRRK2, SELP, and DSP had relatively high mutation burdens, and LRRK2 in particular had more mutations in LUAD than in LUSC (Supplementary Fig. 2C).

The mutation type with the highest frequency was missense mutation (Supplementary Fig. 2D), and when considering broader variant categories, single-nucleotide polymorphisms (SNPs) — a class that includes missense mutations as a subset — were by far the most prevalent type of variant compared to insertions or deletions (Supplementary Fig. 2E). Among SNPs, C > A and C > T substitutions were the most common classes (Supplementary Fig. 2F). The median number of variants per sample was 1, and this distribution was consistent across different variant categories (Supplementary Fig. 2G, H). The ten most frequently mutated genes in this cohort were LRRK2 (28%), SELP (17%), DSP (17%), TOP2A (6%), IVL (5%), CAPN3 (4%), HBB (4%), MMP1 (4%), GPT2 (4%), and AKR1B10 (4%) (Supplementary Fig. 2I). Differences in CNV patterns were also observed between subtypes: for example, homozygous amplifications were more common in LUAD, affecting genes such as IVL, GPIHBP1, SELP, and IL6, whereas homozygous deletions were rare. Heterozygous CNV gains and losses were widespread in both LUAD and LUSC, with genes like GPIHBP1, SELP, IL6, IGFBP3, CAV1, IVL, AKR1B10, PDK4, and CD36 frequently amplified or deleted (Supplementary Fig. 2J, K). These results suggest that genetic alterations in specific ADRGs – through various mutations and CNVs – may play distinct roles in the tumorigenesis of LUAD versus LUSC.

### Prognostic significance of selected ADRGs

**Fig. 4:**
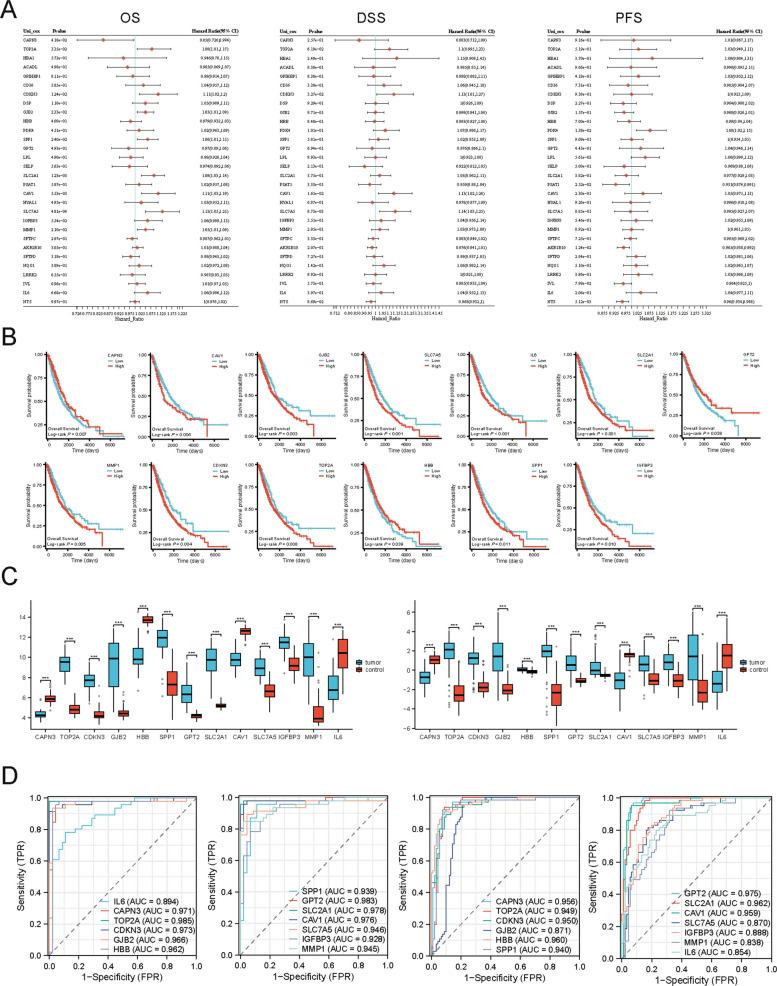
Prognostic evaluation and validation of ADRGs in NSCLC. **A** Forest plots from univariate Cox regression analysis for overall survival (OS), progression-free survival (PFS), and disease-specific survival (DSS), showing hazard ratios (HR) and 95% confidence intervals for 30 ADRGs. **B** Kaplan–Meier survival curves for overall survival (OS) of 13 key ADRGs. **C** Box plots of mRNA expression for prognostic ADRGs in tumor versus control samples from GSE19188 and GSE18842. **D** ROC curves assessing diagnostic performance of prognostic ADRGs in GSE19188 and GSE18842 datasets (AUC values indicated)

In univariate Cox regression analysis for OS, CAPN3 emerged as a significant protective factor (*P =* 0.0418, hazard ratio HR = 0.85), indicating that high CAPN3 expression corresponds to lower mortality risk. In contrast, eight genes were significant risk factors for worse OS (*P <* 0.05, HR > 1). SLC7A5 showed the strongest association with poor outcome (*P =* 4.81 × 10^–4^, HR = 1.13), followed by SLC2A1 (*P =* 0.00123, HR = 1.09), CAV1 (*P =* 0.00535, HR = 1.11), and CDKN3 (*P =* 0.0124, HR = 1.11). For disease-specific survival (DSS), three genes – SLC7A5 (*P =* 0.00873, HR = 1.14), CAV1 (*P =* 0.0165, HR = 1.13), and CDKN3 (*P =* 0.0327, HR = 1.13) – were significant risk factors linked to higher disease-specific mortality. In the progression-free survival (PFS) analysis, elevated PDK4 was associated with shorter time to progression (*P =* 0.0138, HR = 1.08), whereas three genes (NTS, AKR1B10, and PSAT1) appeared protective. Notably, NTS had the strongest protective effect (*P =* 0.00512, HR = 0.96), with higher NTS expression associated with longer PFS. Twenty genes (e.g., HBA1 and ACADL) showed no significant impact on any survival endpoint. Overall, this analysis highlighted SLC7A5, CAV1, and CDKN3 as consistent adverse prognostic markers (especially for OS and DSS), whereas CAPN3 and some PFS-associated genes (NTS, AKR1B10, PSAT1) may serve as favorable prognostic indicators. These genes merit further investigation as prognostic biomarkers and potential therapeutic targets in NSCLC (Fig. 4A).

In Kaplan–Meier survival analyses, high CAPN3 expression showed a trend toward better OS (log-rank *P =* 0.007). By contrast, high expression of several genes — including CAV1 (*P =* 0.004), GJB2 (*P =* 0.003), SLC7A5 (*P <* 0.001), IL6 (*P <* 0.001), SLC2A1 (*P <* 0.001), MMP1 (*P =* 0.005), CDKN3 (*P =* 0.004), TOP2A (*P =* 0.008), SPP1 (*P =* 0.011), and IGFBP3 (*P =* 0.01) — was associated with significantly worse OS, as evidenced by steeper declines in the survival curves. High GPT2 (*P =* 0.038) and HBB (*P =* 0.039) expression was also linked to better survival. These Kaplan–Meier results visually confirm that overexpression of the identified “risk” genes corresponds to inferior outcomes, consistent with the univariate Cox findings (Fig. 4B). In two external cohorts (GSE18842 and GSE19188), tumors with high expression of the prognostic genes had significantly higher transcript levels than those with low expression (Fig. 4C), in line with their upregulation in NSCLC. Moreover, ROC curve analyses demonstrated strong diagnostic performance for these genes. In GSE18842, many individual genes – for example, IL6, CAPN3, TOP2A, CDKN3, GJB2, HBB, SPP1, GPT2, SLC2A1, CAV1, SLC7A5, IGFBP3, and MMP1 – each achieved area-under-the-curve (AUC) values around 0.90 or above for distinguishing NSCLC from normal tissue (Fig. 4D). Similarly, in GSE19188, these genes showed high predictive accuracy (AUC ~ 0.84–0.96). In summary, elevated expression of this panel of genes is consistently associated with NSCLC and may serve as a set of prognostic biomarkers (Fig. 4D).

### Development of an ADRGs-based prognostic model

**Fig. 5:**
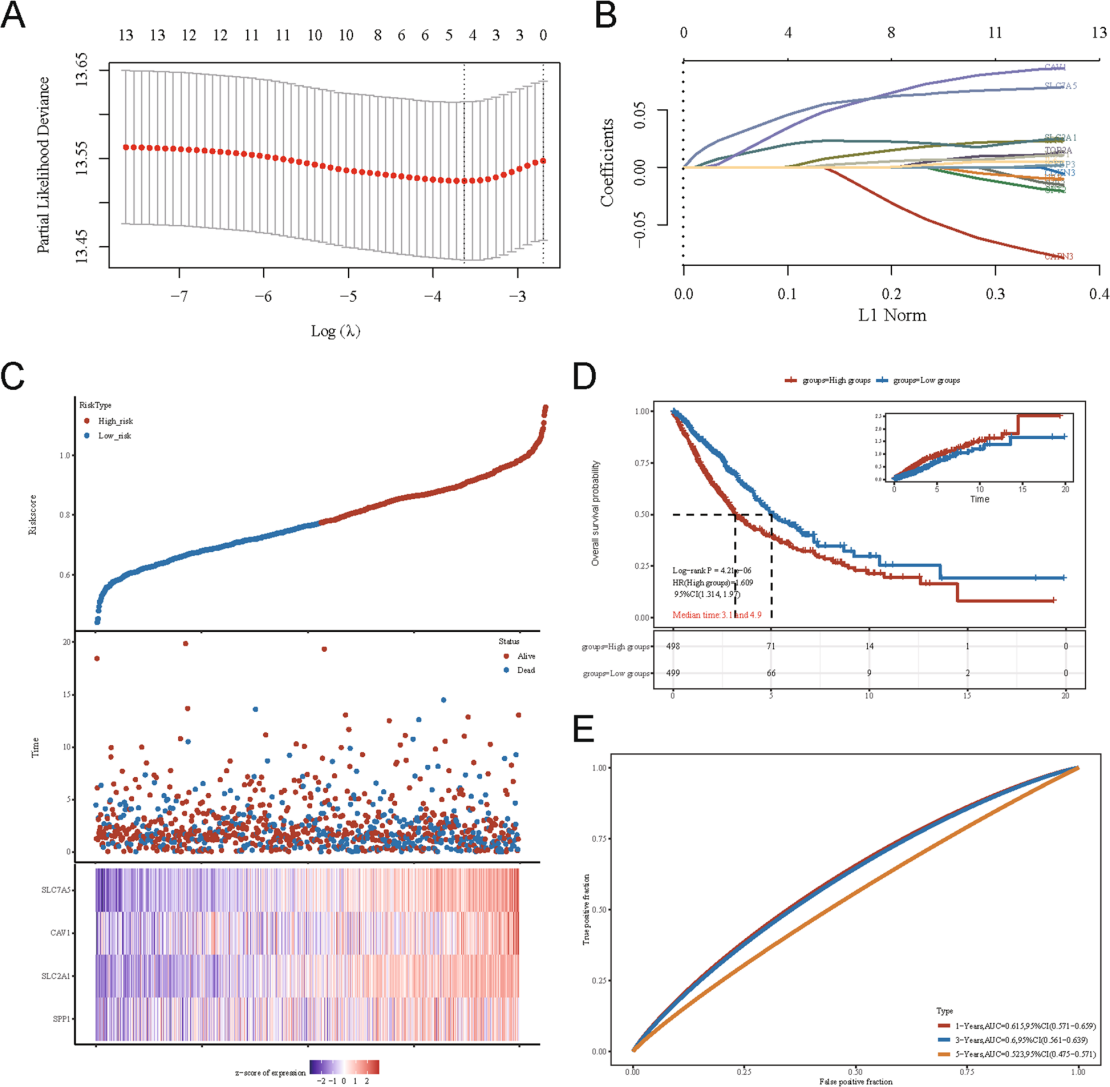
Development of an ADRGs-based prognostic risk model in NSCLC. **A** Cross-validation plot for tuning parameter selection (lambda). **B** LASSO coefficient profiles for prognostic ADRGs. **C** Risk score distribution, patient survival status, and heatmap of 13 selected ADRGs expression in high- versus low-risk groups. **D** Kaplan–Meier OS curves for high- and low-risk groups (log-rank *P =* 4.21e-06; HR = 1.609, 95% CI: 1.314, 1.97). **E** Time-dependent ROC curves for 1-, 3-, and 5-year OS prediction (AUC: 0.615, 0.600, 0.523, respectively)

A four-gene prognostic signature was constructed by applying LASSO Cox regression to the candidate genes. The optimal model (at the minimum lambda) incorporated four genes for OS prediction: Risk Score = 0.0036 × SPP1 + 0.0218 × SLC2A1 + 0.0415 × CAV1 + 0.0502 × SLC7A5 (Fig. 5A, B). NSCLC patients in the TCGA cohort were categorized into high-risk and low-risk groups according to a risk score, with the median risk score serving as the threshold. Consistent with expectations, the high-risk group exhibited an increased mortality risk and reduced survival, as illustrated in Fig. 5C. Additionally, a subgroup analysis was conducted to elucidate the prognostic significance of the signature across various NSCLC histological subtypes, specifically comparing lung adenocarcinoma (LUAD) and lung squamous cell carcinoma (LUSC), to determine its relevance to each subtype (Supplementary Fig. 3). Kaplan–Meier analysis confirmed that the high-risk group had significantly worse OS than the low-risk group (median 3.1 vs. 4.9 years; log-rank *P =* 4.21 × 10^–6^; HR = 1.609) (Fig. 5D). The model’s predictive performance was modest, with AUCs of 0.615, 0.600, and 0.523 for predicting 1-year, 3-year, and 5-year OS, respectively (Fig. 5E).

### Association of ADRGs risk signature with clinicopathological features

We examined the relationship between the ADRGs-based risk signature and clinical characteristics. Using TCGA clinical data, patients were stratified by primary tumor size/invasion (T1–T4) to assess how the key genes’ expression varies with tumor progression. SLC2A1 and SPP1 were highly expressed across all T stages, whereas CAV1 expression remained low in tumors regardless of T stage (Supplementary Fig. 4 A). Notably, SLC2A1 showed significantly higher expression in more advanced (mid-late T2–T3) tumors compared to early-stage (T1) disease; similarly, SLC7A5 also exhibited higher expression in mid-late T2–T3 tumors. For SLC2A1 specifically, this expression pattern suggests it could serve as an indicator of tumor progression: lower SLC2A1 levels were associated with better patient outcomes, whereas higher levels implied greater tumor aggressiveness, an increased likelihood of progression, and consequently worse prognosis. We next generated a heatmap integrating each patient’s risk score and key ADRGs expression levels with various clinicopathological factors (age, TNM stage, overall stage, smoking history, sex, vital status, treatment type, etc.) (Supplementary Fig. 4B). The risk score was significantly associated with certain clinical variables – notably patient sex, survival status, and smoking history. Moreover, the expression patterns of SLC7A5, SLC2A1, CAV1, and SPP1 were consistent with their contributions to the risk score (patients with high risk scores tended to have high SLC7A5/SLC2A1/SPP1 and low CAV1). We also divided patients into two groups based on the risk signature and compared their distributions across clinical characteristics (Supplementary Fig. 4C). The higher-risk group (Group 2) contained a greater proportion of patients with features such as older age, male sex, a history of smoking, and advanced tumor burden (higher T stage, N stage, and presence of metastasis). Group 2 patients were also more likely to have certain ethnic backgrounds and to have received aggressive treatments (e.g., radiotherapy or neoadjuvant therapy) compared to Group 1. These findings indicate that the ADRGs-derived risk profile correlates not only with tumor molecular features but also with patient demographics and disease presentation.

### Construction of a predictive nomogram for survival

**Fig. 6:**
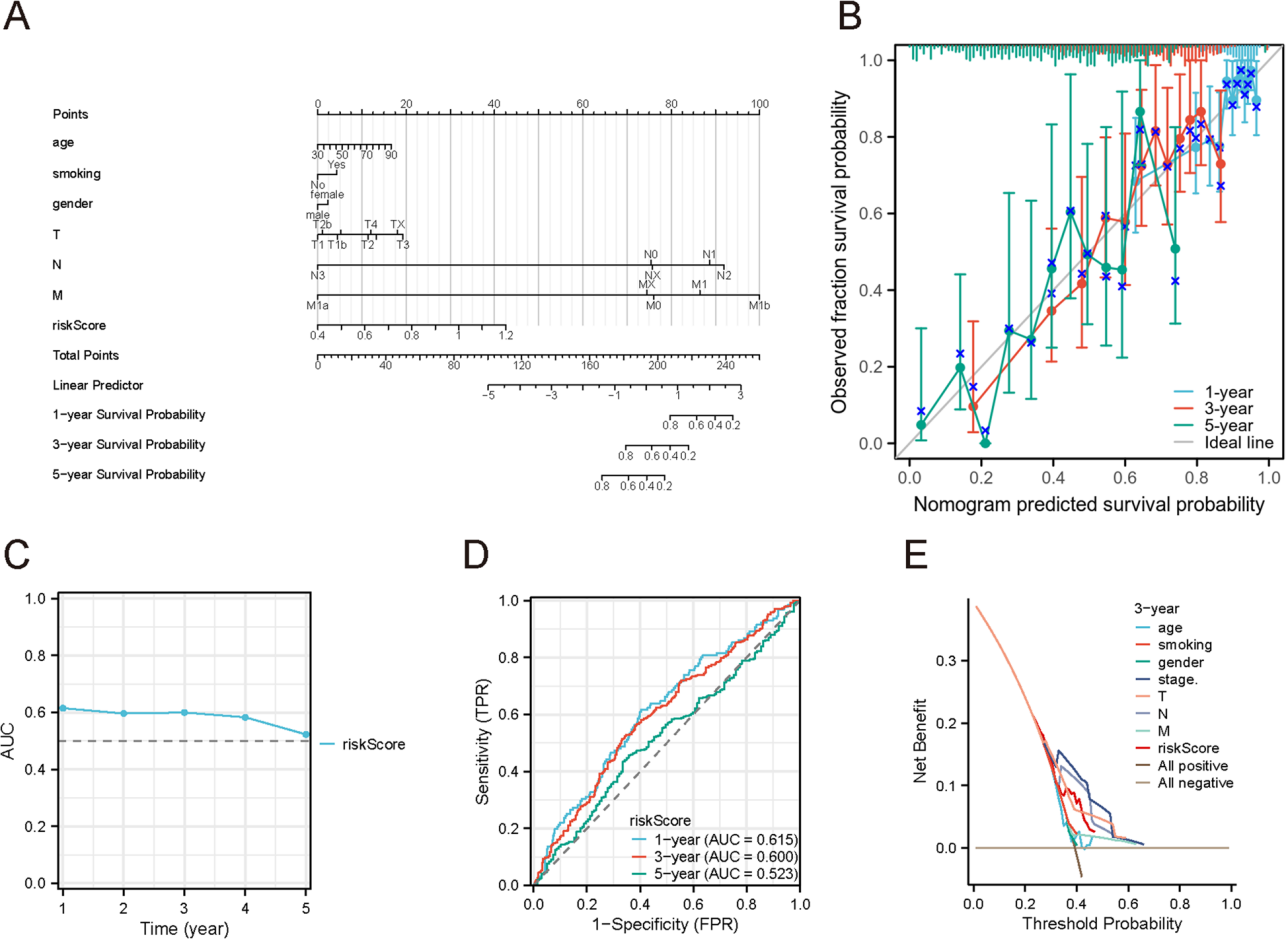
Nomogram for prognostic prediction in NSCLC. **A** Nomogram integrating age, smoking, gender, TNM stage, and risk score to predict 1-, 3-, and 5-year OS probabilities. **B** Calibration curves comparing predicted versus observed OS at 1-, 3-, and 5-years (diagonal line: ideal reference). **C** Time-dependent AUC plot for OS prediction. **D** ROC curves for 1-, 3-, and 5-year OS prediction. **E** Decision curve analysis (DCA) evaluates the net benefit of the nomogram versus individual predictors or strategies (all positive/negative)

To facilitate clinical application, we constructed a prognostic nomogram that integrates the ADRGs risk score with other significant clinical factors. Multivariate Cox analysis confirmed that the risk score was an independent predictor of OS (Supplementary Table 4). We therefore combined the risk score with select clinicopathological variables (*P <* 0.05) in a multivariable model to predict 1-, 3-, and 5-year OS probabilities (Fig. 6A).

The nomogram showed good short-term predictive accuracy. Calibration analysis demonstrated excellent agreement between the nomogram-predicted and actual observed 1-year OS (Fig. 6B). However, calibration for 3- and 5-year OS was less optimal, likely because long-term outcomes are influenced by additional factors (e.g., subsequent treatments, comorbidities) beyond the initial model. Time-dependent AUC analysis indicated that the nomogram achieved higher AUC values for 1-, 2-, and 3-year OS prediction than any single clinical factor (Fig. 6C), and this performance was validated using ROC curve (Fig. 6D). Finally, decision curve analysis showed that the nomogram provided a greater net benefit across a range of threshold probabilities, underscoring its utility and reliability as a prognostic tool (Fig. 6E).

### Immune cell infiltration patterns in subtypes and risk groups

**Fig. 7:**
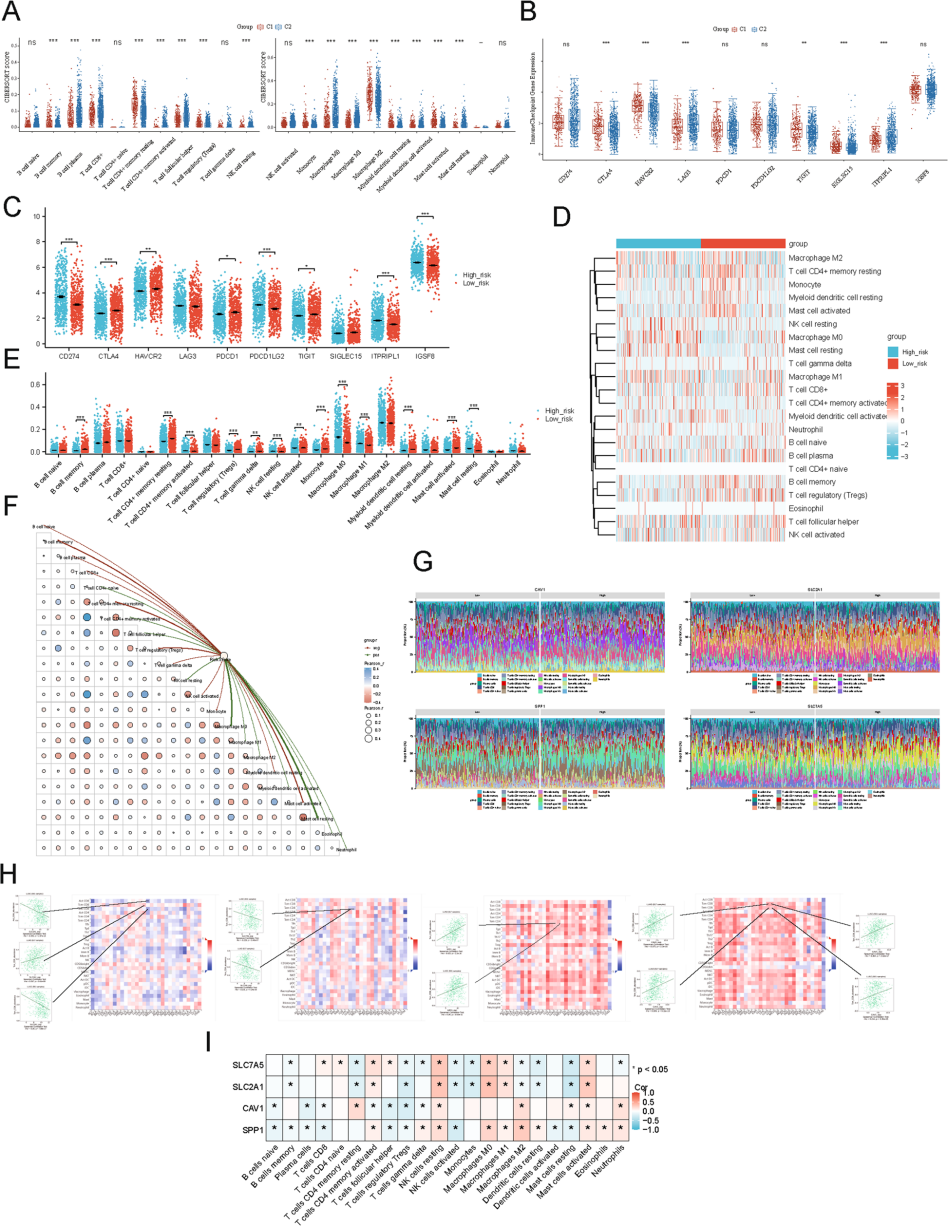
Immune landscape and checkpoint gene expression in NSCLC risk groups and subtypes. **A**, **B** Box plots comparing immune cell infiltration (CIBERSORT) and checkpoint gene expression between C1 and C2 subtypes (***P < 0.001, **P < 0.01, *P < 0.05, ns: not significant). **C** Box plots of checkpoint gene expression in high- versus low-risk groups (***P < 0.001, **P < 0.01, *P < 0.05). **D** Heatmap of Pearson correlations between high- and low-risk groups and 22 immune cell types. **E** Comparison of tumor-infiltrating immune cell proportions between high- and low-expression groups of four prognostic ADRGs. Colors denote high- and low-expression groups, with the x-axis showing immune cell types and the y-axis indicating relative immune cell abundance. (***P < 0.001, **P < 0.01). **F** Bubble chart of immune scores across risk groups. **G** Stacked bar plots of immune cell proportions in high- and low-expression groups for CAV1, SLC2A1, SLC7A5, and SPP1. **H** Correlation heatmap of four prognostic ADRGs with immune cell types (pan-cancer; *P < 0.05 **P < 0.01). **I** Heatmap of immune scores with four ADRGs (***P < 0.001, **P < 0.01, *P < 0.05, ns: not significant)

We analyzed the tumor immune microenvironment in the C1 and C2 subtypes. Twenty-two immune cell subsets showed significantly different infiltration levels between C1 and C2. For instance, resting memory CD4^+^ T cells and activated mast cells were more abundant in one subtype than the other (Fig. 7A). Additionally, immune checkpoint genes such as CTLA4 and HAVCR2 (TIM-3) were expressed at significantly different levels in C1 vs. C2 tumors (Fig. 7B), indicating that the two subtypes differ in their immunological landscapes.

We next evaluated immune profiles in high- vs. low-risk patient groups defined by the ADRGs signature. Multiple checkpoint genes, including CD274 (PD-L1), CTLA4, and others, were differentially expressed between high- and low-risk tumors (Fig. 7C). Likewise, we observed significant differences in the infiltration of at least 13 immune cell types between the high-risk and low-risk groups (Fig. 7D, E). In general, high-risk tumors exhibited an immune infiltrate profile distinct from that of low-risk tumors. A CIBERSORT-based correlation analysis confirmed that most immune cell fractions were significantly associated with the risk score, underscoring a strong link between our gene signature and the immune microenvironment (Fig. 7F). We also visualized immune cell composition relative to the expression of the four prognostic genes: a stacked bar chart illustrated how the proportions of various immune cells shifted with differing gene expression profiles (Fig. 7G). Finally, a pan-cancer analysis using the TISIDB database revealed that SLC7A5, SLC2A1, CAV1, and SPP1 expression is strongly correlated with T cell abundance across multiple cancer types. Further correlation analyses (Fig. 7H, I) helped clarify the roles of these genes in shaping the tumor immune milieu, suggesting that SLC7A5, SLC2A1, CAV1, and SPP1 may influence T cell infiltration or function in NSCLC.

### Single-cell transcriptomic analysis of T cells in NSCLC

**Fig. 8:**
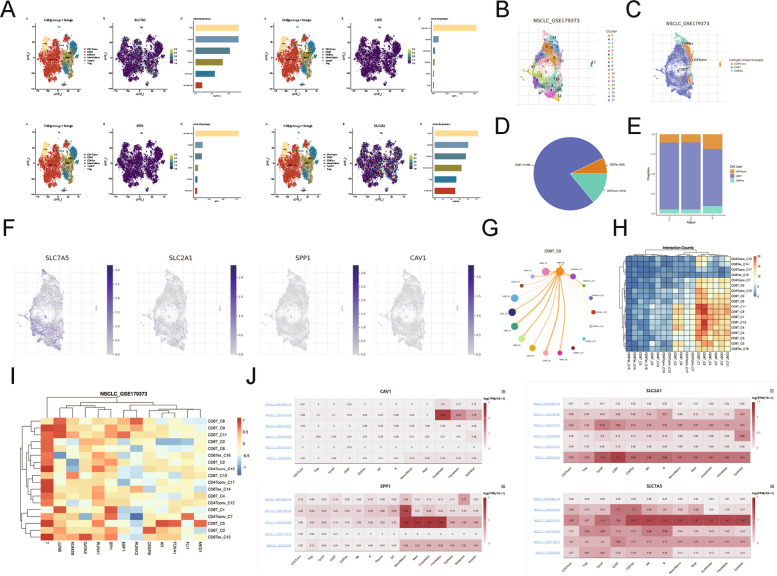
Single-cell expression profiles of prognostic ADRGs in NSCLC immune cells. **A** UMAP plot of ADRGs expression patterns across T-cell populations in GSE99254. **B** UMAP clustering of cell types from scRNA-seq data. **C** UMAP annotation of immune cell lineages in NSCLC tissues (GSE179373). **D** Pie chart depicting proportions of each cell type. **E** Stacked bar plot of subtype proportions in GSE179373. **F** Feature plots for SLC7A5, SLC2A1, SPP1, and CAV1. **G** Pseudotime trajectory network centered on CD8T_C0 cluster. **H** Heatmap of cell cluster interaction counts. **I** Heatmap of transcription factor enrichment across cell clusters. **J** Heatmap of SLC7A5, SLC2A1, SPP1, and CAV1 expression in scRNA-seq data

We analyzed ADRGs expression in immune cell subsets, focusing on T cells, using a NSCLC single-cell RNA sequencing dataset (GSE99254). CAV1 exhibited uniformly low expression across all CD8^+^ T cells and the entire dataset. SLC2A1 and SPP1 were also generally low but elevated in specific subpopulations: CD8^+^ T cells showed higher SLC2A1 than proliferating T cells, exhausted CD8^+^ T cells (CD8 Tex), or CD4^+^ conventional T cells (CD4 Tconv); conversely, they displayed higher SPP1 than CD4 Tconv. SLC7A5 was low in most cells but enriched in high-expression subclusters of regulatory T cells (Tregs), proliferating T cells, CD8^+^ T cells, and CD8 Tex (Fig. 8A). These patterns reveal distinct ADRGs distributions among T cell subsets: SLC7A5 and SLC2A1 associate with activated states, while CAV1 and SPP1 are largely absent.

Using the TISCH2.0 database for NSCLC_GSE179373, which identifies 17 clusters in 3 major cell types, we confirmed SLC7A5 localization to CD8^+^ T and CD8 Tex clusters, low SLC2A1 confinement to CD8^+^ T cells, and minimal SPP1 and CAV1 in T cell clusters (Fig. 8B–F). Pie and bar plots illustrated the immune landscape, with CD8^+^ T cells dominating T cell fractions in NSCLC samples (Fig. 8D, E).

Pseudotime analysis positioned CD8T_C0 as an early, progenitor-like node linking to multiple downstream CD8⁺ clusters (C1–C13) and, weaklier, to CD4 Tconv (C7, C10, C12, C17) and CD8 Tex (C14–C16), suggesting limited lineage plasticity (Fig. 8G). Interaction mapping showed broad communication from CD8T_C0/C3–C5, whereas CD8Tex_C14–C15 and CD4Tconv_C17 were relatively isolated (Fig. 8H). Transcription-factor profiling reinforced subset heterogeneity: Transcription-factors such as T and JUNB were enriched in most CD8⁺ and CD4 Tconv clusters and depleted elsewhere (Fig. 8I). Cross-dataset comparisons supported generalizability—CAV1 was stromal-enriched (fibroblasts/endothelium) in some datasets, whereas SLC2A1 and SLC7A5 consistently marked proliferating or CD8⁺ T cells and select stromal compartments; SPP1 was largely myeloid/fibroblast-restricted (Fig. 8J). Overall, SLC2A1/SLC7A5 show broader immune–stromal distribution, while CAV1/SPP1 remain context-dependent and cell-type specific.

### Functional impact of key ADRGs on AICD in T cells

**Fig. 9:**
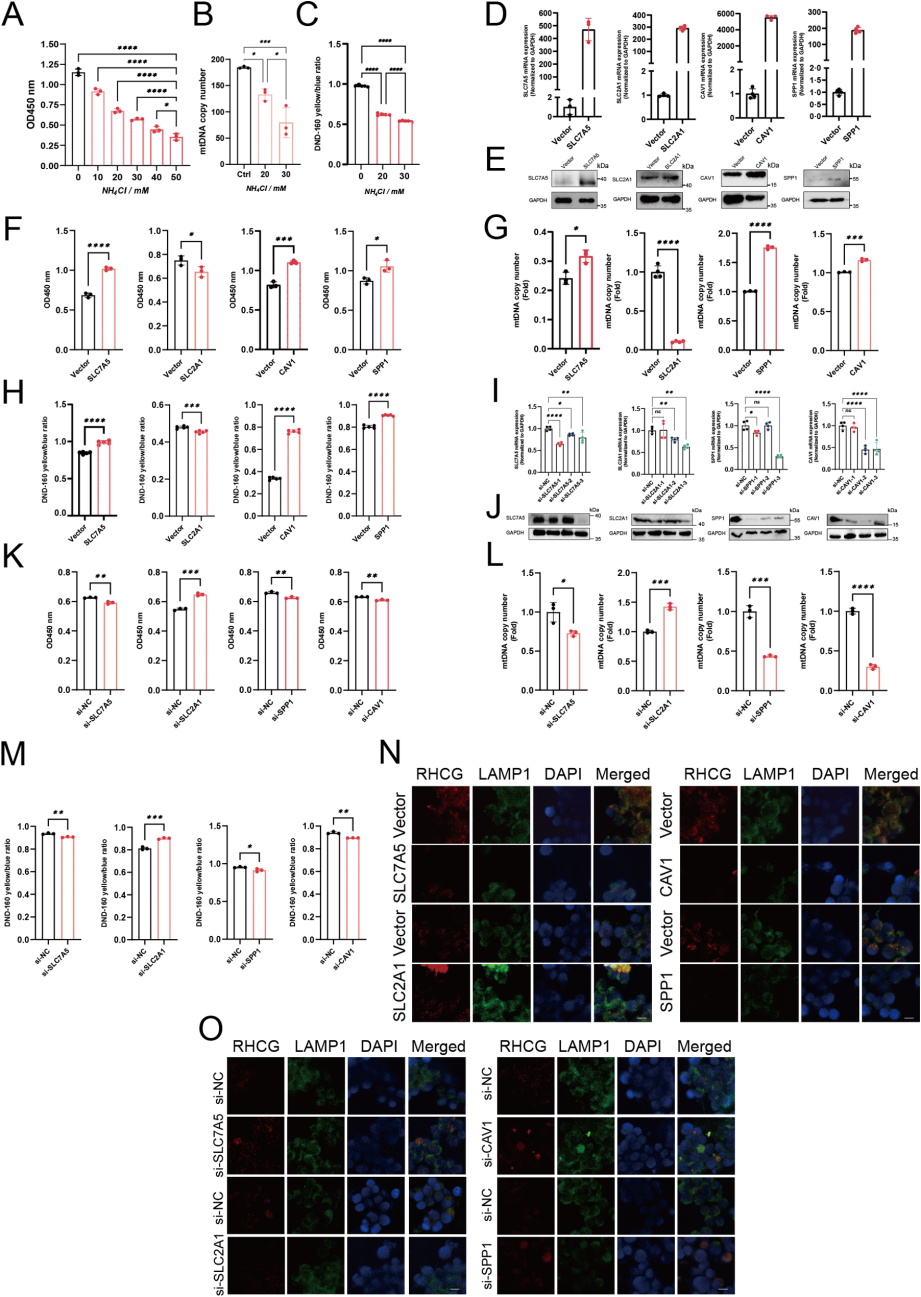
**A** Proliferation of Jurkat cells determined by CCK-8 assay following 24 h exposure to increasing concentrations of NH₄Cl (0–50 mM) (*n*=3). **B** Relative mitochondrial DNA (mtDNA) copy number under the same NH₄Cl treatment conditions (*n*=3). C Lysosomal pH values of Jurkat cells treated with NH₄Cl for 24 h (*n*=5). **D–E** Validation of CAV1, SPP1, SLC2A1, and SLC7A5 overexpression in Jurkat cells by quantitative PCR (*n*=3) (D) and Western blotting (E). **F** Jurkat cells stably overexpressing each candidate gene were exposed to 30 mM NH₄Cl for 24 h, and cell viability (CCK-8 assay) of control versus gene-overexpressing Jurkat cells under NH₄Cl treatment (*n*=3). **G** Relative mtDNA copy number in gene-overexpressing Jurkat cells exposed to 30 mM NH₄Cl (*n*=3). **H** Lysosomal pH measurements in gene-overexpressing Jurkat cells after 30 mM NH₄Cl treatment (n=5).** I–J** siRNA-mediated knockdown of SLC7A5, SLC2A1, SPP1, and CAV1 was performed, and knockdown efficiency was validated by quantitative PCR(I) and Western blotting (J). **K** Jurkat cells with stable knockdown of each indicated gene were treated with 30 mM NH₄Cl for 24 h, followed by assessment of cell viability using the CCK-8 assay (*n*=3). **L** Relative mitochondrial DNA copy number in Jurkat cells after gene knockdown upon exposure to 30 mM NH₄Cl (*n*=3). **M** Lysosomal pH in gene-knockdown Jurkat cells following treatment with 30 mM NH₄Cl. **N–O** Immunofluorescence staining showing RHCG expression and lysosomal localization after gene overexpression (N) or knockdown (O), as indicated by LAMP staining. Statistical significance was determined by two-tailed Student’s t-test (*P < 0.05, **P < 0.01, ***P < 0.001)

Finally, we tested the roles of four key ammonia‐associated genes in T cell ammonia toxicity. We exposed Jurkat T cells to escalating concentrations of NH₄Cl. This ammonia stress induced pronounced, dose-dependent cytotoxicity, characterized by inhibited cell proliferation (Fig. 9A), diminished mtDNA copy number (Fig. 9B), and increased lysosomal pH, reflecting alkalinization and impaired lysosomal function (Fig. 9C).

We subsequently generated stable Jurkat cell lines overexpressing four candidate ADRGs—CAV1, SPP1, SLC2A1, and SLC7A5—with overexpression verified at the mRNA and protein levels via qPCR and Western blotting, respectively (Fig. 9D, E). These lines were challenged with 30 mM NH₄Cl for 24 h, and functional assessments were performed. Overexpression of CAV1, SPP1, or SLC7A5 enhanced cell viability relative to vector controls, whereas SLC2A1 overexpression aggravated ammonia-induced cytotoxicity (Fig. 9F). In line with this, SLC7A5, CAV1, and SPP1 overexpression mitigated mtDNA depletion, while SLC2A1 exacerbated it (Fig. 9G). Similarly, CAV1-, SPP1-, and SLC7A5-overexpressing cells resisted lysosomal alkalinization, preserving acidity, whereas SLC2A1 overexpression intensified this dysfunction (Fig. 9H). We further performed siRNA-mediated knockdown experiments targeting SLC7A5, SLC2A1, CAV1, and SPP1 in Jurkat cells (Fig. 9I, J). Results show that knockdown of SLC7A5, CAV1, or SPP1 increases sensitivity to ammonia-induced cytotoxicity (reduced viability, mtDNA depletion, lysosomal alkalinization), while SLC2A1 knockdown confers resistance, mirroring the opposing effects seen in overexpression (Fig. 9K-M).

Previous studies have shown that rhesus family glycoproteins (RHAG, RHBG, RHCG) serve as ammonia transporters [26–28], and that RHCG is highly upregulated during ammonia-induced cell death [8]. In this study, we found that ADRGs modulate RHCG expression under ammonia-induced cytotoxic conditions. Immunofluorescence analysis demonstrated that SLC7A5, SPP1, and CAV1 suppressed lysosomal RHCG expression, whereas SLC2A1 promoted its expression (Fig. 9N, O).

Collectively, these findings indicate that CAV1, SPP1, and SLC7A5 confer protection against ammonia-mediated T cell death by supporting mitochondrial integrity and lysosomal homeostasis, in contrast to SLC2A1, which heightens vulnerability to ammonia toxicity.

## Discussion

Immunotherapy has revolutionized NSCLC treatment, yet its efficacy is often limited by T cell dysfunction within the TME. Recent discoveries have highlighted AICD as a novel mechanism contributing to effector CD8⁺ T cell demise, driven by glutaminolysis-derived ammonia accumulation that disrupts lysosomal and mitochondrial integrity [8, 9, 29]. This metabolic vulnerability not only explains rapid T cell attrition post-activation but also underscores ammonia's role as an active immunosuppressive metabolite, akin to lactate or kynurenine [4, 30, 31]. Strategies targeting AICD, such as ammonia neutralization or autophagy enhancement, hold promise for bolstering T cell persistence and antitumor immunity [11].

Glutamine metabolism facilitates T-cell activation, proliferation, and biosynthesis through TCA cycle anaplerosis and nucleotide synthesis [32, 33]. By contrast, AICD represents an excessive accumulation of ammonia increases the pH value of lysosomes, leading to the termination of lysosomal ammonia storage and the reflux of ammonia into mitochondria, resulting in mitochondrial damage and cell death. This mode of cell death is different from the mechanisms of other forms of cell death discovered previously [8, 29].

Furthermore, unlike T-cell exhaustion—a reversible functional impairment driven by chronic antigen stimulation, immunosuppressive cytokines, and checkpoint molecules (e.g., PD-1, CTLA-4) without inherent lethality [34]—AICD is a lethal process marked by direct organelle disruption. This distinction highlights the potential for targeting AICD pathways (e.g., RHCG inhibition or ammonia scavengers such as sodium phenylbutyrate) to synergize with checkpoint inhibitors, thereby enhancing CD8^+^ T-cell persistence in NSCLC [29].

This study elucidates the prognostic and immunological significance of ADRGs in NSCLC, identifying SLC7A5, SLC2A1, CAV1, and SPP1 as key regulators. Mechanistically, these genes modulate metabolic reprogramming and organelle integrity in the TME. SLC7A5 and SLC2A1 facilitate amino acid and glucose transport, respectively, influencing glutamine uptake and subsequent ammonia production, which drives lysosomal alkalinization via the RHCG transporter, mitochondrial dysfunction, ROS accumulation, and impaired autophagy, culminating in T-cell apoptosis [8]. CAV1 supports membrane trafficking and autophagic flux, potentially mitigating ammonia-induced lysosomal permeabilization [35], while SPP1 restrains CD8^+^ T-cell activation through CD44 signaling, exacerbating immune suppression [36]. Functional assays confirmed these roles: overexpression of CAV1, SPP1, and SLC7A5 conferred resistance to ammonia stress in Jurkat cells by preserving mitochondrial DNA copy number and lysosomal pH, whereas SLC2A1 heightened susceptibility, aligning with AICD's organelle-targeted toxicity distinct from broader glutaminolysis pathways.

Translationally, the four-gene signature predicts overall survival and immunotherapy response, with high-risk patients showing elevated TIDE scores and reduced response rates in cohorts, highlighting its utility for patient stratification [25]. Analysis of the correlation between the four-gene signature and immunotherapy response in clinical cohorts also suggests its potential value in immunotherapy (Supplementary Fig. 6). Targeting AICD pathways—such as RHCG inhibition or ammonia scavengers (e.g., sodium phenylbutyrate)—could synergize with checkpoint inhibitors to enhance CD8^+^ T-cell persistence and overcome immune evasion in NSCLC [11]. Limitations include modest AUC values (< 0.65), indicating constrained predictive accuracy. We implemented multiple modeling approaches—LASSO regression, Cox proportional hazards analysis, and stepwise Cox modeling—to comprehensively assess the predictive performance of our 4 -gene signature. Importantly, all three independent modeling strategies consistently identified statistically significant differences in patient outcomes between risk groups, strongly supporting that our signature captures biologically meaningful prognostic information. While the area under the curve (AUC) values for these models were relatively modest (all below 0.65, as shown in Supplementary Fig. 7), we believe this primarily reflects inherent characteristics of our dataset rather than methodological issues. Several factors likely contribute to these moderate predictive values, including the biological diversity within our patient cohort, the small-to-moderate effect sizes of individual genes in the signature, and our study's limited sample size. Furthermore, while Jurkat cells serve as a dependable immortalized model for studying T-cell biology due to their genetic stability, ease of manipulation, and consistent responses to metabolic stressors like ammonia, it is imperative that future research validates these findings in primary T cells and through prospective trials to advance the modulation of AICD as a therapeutic strategy.

## Conclusion

Our findings demonstrate that ammonia-induced metabolic stress in NSCLC exerts a dual influence, damaging tumor cells while simultaneously weakening antitumor immunity. We defined AICD as a distinct programmed cell death initiated by impaired ammonia clearance, which disrupts lysosomal and mitochondrial integrity and links metabolic reprogramming to immune escape. Four genes-SLC7A5, SLC2A1, CAV1, and SPP1-emerge as critical nodes connecting ammonia metabolism with tumor–immune interactions. These insights suggest that therapeutic strategies directed at ammonia homeostasis, pH regulation, or nutrient competition could reinforce immunotherapy and potentially drive selective metabolic collapse in cancer cells. Further work is required to validate these mechanisms in vivo, explore their interplay with existing treatments, and develop clinically applicable approaches. By repositioning ammonia as an active regulator of tumor progression and immune surveillance, this study broadens the conceptual framework of NSCLC biology and provides a rationale for metabolic–immunological interventions in lung cancer.

## Supplementary Information

Supplementary Material 1.

Supplementary Material 2.

## Data Availability

The code and datasets analyzed during the current study are available from the corresponding author upon reasonable request. All data used in this study are publicly accessible to ensure reproducibility. Transcriptomic data for NSCLC were obtained from The Cancer Genome Atlas (TCGA) database. Normal lung tissue expression data were retrieved from the Genotype-Tissue Expression (GTEx) database (V8 release) to supplement normal controls. Additional microarray datasets (GSE19188, GSE18842, GSE179373, and GSE99254) were downloaded from the Gene Expression Omnibus (GEO) database for validation and single-cell analyses.

## Abbreviations

AICD: Ammonia-induced cell death
ADRGs: Ammonia-induced cell death-related genes
CAV1: Caveolin-1
CNV: Copy number variation
DEGs: Differentially expressed genes
DCA: Decision curve analysis
GEO: Gene Expression Omnibus
GSEA: Gene Set Enrichment Analysis
GSCA: Gene Set Cancer Analysis
LASSO: Least absolute shrinkage and selection operator
LUAD: Lung adenocarcinoma
LUSC: Lung squamous cell carcinoma
mtDNA: Mitochondrial DNA
NSCLC: Non-small cell lung cancer
OS: Overall survival
PCA: Principal component analysis
PFS: Progression-free survival
qPCR: Quantitative real-time polymerase chain reaction
SLC2A1: Solute Carrier Family 2 Member 1
SLC7A5: Solute Carrier Family 7 Member 5
SNP: Single-nucleotide polymorphism
SPP1: Secreted Phosphoprotein 1
TME: Tumor microenvironment
TCGA: The Cancer Genome Atlas
TIDE: Tumor Immune Dysfunction and Exclusion
DSS: Disease-specific survival
