# Supplementary material for: Prognostic and immunological roles of ammonia-induced cell death-related genes in non-small cell lung cancer
Source: BMC Pulm Med. 2026 Feb 21;26:138. doi: 10.1186/s12890-026-04181-7 (PMC13032429; doi:10.1186/s12890-026-04181-7)
Supplement: Supplementary file 1 — Supplementary Material 1. [file 12890_2026_4181_MOESM1_ESM.zip › Supplementary Table 1.docx]

| **Table S1. The clinical characters of NSCLC patients in TCGA cohort** | | | | | | | | | | | | | | | | | | | | | |  |
| --- | --- | --- | --- | --- | --- | --- | --- | --- | --- | --- | --- | --- | --- | --- | --- | --- | --- | --- | --- | --- | --- | --- |
|  | **Age** | **Gender** | **Race** | **pT_**  **stage** | **pN_**  **stage** | **pM_stage** | **pTNM_stage** | **Grade** | **new_tumor_event_type** | **Smoking** | **radiation_**  **therapy** | **neoadjuvant_**  **treatment** | **therapy_type** | **OS** | **OS.**  **time** | **DSS** | **DSS.**  **time** | **DFI** | **DFI.**  **time** | **PFI** | **PFI.**  **time** | **Groups** |
| TCGA-05-4244 | 70 | MALE | NA | T2 | N2 | M1 | IV | NA | NA | Yes | NA | No | NA | 0 | 0 | 0 | 0 | NA | NA | 0 | 0 | G1 |
| TCGA-05-4249 | 67 | MALE | NA | T2 | N0 | M0 | I | NA | NA | Yes | NA | No | NA | 0 | 1523 | 0 | 1523 | NA | NA | 0 | 1523 | G1 |
| TCGA-05-4384 | 66 | MALE | NA | T2 | N2 | M0 | III | NA | Metastasis | Yes | NA | No | NA | 0 | 426 | 0 | 426 | NA | NA | 1 | 183 | G1 |
| TCGA-05-4396 | 76 | MALE | NA | T4 | N1 | M0 | III | NA | NA | Yes | NA | No | NA | 1 | 303 | NA | 303 | NA | NA | 0 | 303 | G1 |
| TCGA-05-4403 | 76 | MALE | NA | T2 | N0 | M0 | I | NA | NA | Yes | NA | No | NA | 0 | 578 | 0 | 578 | NA | NA | 0 | 578 | G1 |
| TCGA-05-4405 | 74 | FEMALE | NA | T2 | N0 | M0 | I | NA | NA | Yes | NA | No | NA | 0 | 610 | 0 | 610 | NA | NA | 0 | 610 | G1 |
| TCGA-05-4422 | 68 | MALE | NA | T2 | N0 | M0 | I | NA | NA | Yes | NA | No | NA | 0 | 365 | 0 | 365 | NA | NA | 0 | 365 | G1 |
| TCGA-05-4424 | 70 | MALE | NA | T3 | N0 | M0 | II | NA | Metastasis | Yes | NA | No | Immunotherapy | 0 | 913 | 0 | 913 | NA | NA | 1 | 153 | G1 |
| TCGA-05-4426 | 71 | MALE | NA | T2 | N0 | M0 | I | NA | NA | Yes | NA | No | NA | 0 | 791 | 0 | 791 | 1 | 457 | 1 | 457 | G1 |
| TCGA-05-4430 | 59 | FEMALE | NA | T2 | N0 | M0 | I | NA | NA | Yes | NA | No | NA | 0 | 761 | 0 | 761 | NA | NA | 0 | 761 | G1 |
| TCGA-05-4433 | 82 | MALE | NA | T2 | N0 | M0 | I | NA | NA | Yes | NA | No | NA | 0 | 730 | 0 | 730 | NA | NA | 0 | 730 | G1 |
| TCGA-05-5423 | 65 | MALE | NA | T2 | N1 | M0 | II | NA | NA | Yes | NA | No | NA | 0 | 151 | 0 | 151 | NA | NA | 0 | 151 | G1 |
| TCGA-05-5715 | 69 | FEMALE | NA | T2 | N0 | M0 | I | NA | NA | No | NA | No | NA | 0 | 62 | 0 | 62 | NA | NA | 0 | 62 | G1 |
| TCGA-22-1002 | 69 | MALE | WHITE | T1 | N0 | M0 | I | NA | NA | Yes | NA | No | NA | 1 | 131 | 0 | 131 | NA | NA | 0 | 131 | G1 |
| TCGA-22-1016 | 65 | MALE | WHITE | T2 | N0 | M0 | I | NA | NA | Yes | NA | No | NA | 1 | 822 | 0 | 822 | 0 | 822 | 0 | 822 | G1 |
| TCGA-22-5472 | 67 | MALE | WHITE | T2 | N0 | M0 | I | NA | NA | Yes | NA | No | Chemotherapy | 1 | 1975 | 0 | 1975 | 1 | 746 | 1 | 746 | G1 |
| TCGA-38-4626 | 57 | FEMALE | WHITE | T2 | N0 | M0 |  | NA | Primary | Yes | NA | No | NA | 0 | 3674 | 0 | 3674 | 0 | 3674 | 1 | 2518 | G1 |
| TCGA-38-4627 | 64 | FEMALE | WHITE | T1 | N1 | M0 | II | NA | NA | Yes | NA | No | NA | 1 | 1147 | NA | 1147 | 0 | 1147 | 0 | 1147 | G1 |
| TCGA-38-6178 | 70 | FEMALE | WHITE | T2 | N2 | NA | III | NA | NA | No | NA | No | Chemotherapy | 0 | 448 | 0 | 448 | NA | NA | 0 | 448 | G1 |
| TCGA-38-7271 | 72 | FEMALE | WHITE | T1 | N0 | M0 | I | NA | NA | Yes | NA | No | Chemotherapy | 1 | 800 | 1 | 800 | 1 | 304 | 1 | 304 | G1 |
| TCGA-38-A44F | 80 | MALE | WHITE | T2 | N0 | M0 | I | NA | NA | Yes | NO | No | NA | 0 | 133 | 0 | 133 | 0 | 133 | 0 | 133 | G1 |
| TCGA-44-2655 | 65 | FEMALE | WHITE | T1 | N0 | M0 | I | NA | Primary | Yes | NA | No | NA | 0 | 1324 | 0 | 1324 | 0 | 1324 | 1 | 1009 | G1 |
| TCGA-44-2656 | 59 | MALE | WHITE | T2 | N0 | M0 | I | NA | Primary | Yes | NA | No | NA | 0 | 1429 | 0 | 1429 | 0 | 1429 | 1 | 568 | G1 |
| TCGA-44-2657 | 74 | FEMALE | WHITE | T2 | NA | M0 | I | NA | NA | Yes | NA | No | NA | 0 | 1351 | 0 | 1351 | 0 | 1351 | 0 | 1351 | G1 |
| TCGA-44-2659 | 65 | FEMALE | WHITE | T1 | N1 | M0 | II | NA | Recurrence | Yes | NA | No | Chemotherapy | 0 | 1367 | 0 | 1367 | 1 | 1146 | 1 | 1146 | G1 |
| TCGA-44-2661 | 69 | FEMALE | WHITE | T1 | N0 | M0 | I | NA | NA | No | NA | No | NA | 0 | 1159 | 0 | 1159 | 0 | 1159 | 0 | 1159 | G1 |
| TCGA-44-2662 | 65 | MALE | WHITE | T2 | N0 | M0 | I | NA | NA | Yes | NA | No | NA | 0 | 1280 | 0 | 1280 | NA | NA | 1 | 245 | G1 |
| TCGA-44-2665 | 55 | FEMALE | WHITE | T2 | N1 | M0 | II | NA | NA | No | NA | No | NA | 0 | 1301 | 0 | 1301 | NA | NA | 0 | 1301 | G1 |
| TCGA-44-2666 | 43 | MALE | WHITE | T2 | N0 | M0 | I | NA | NA | Yes | NA | No | NA | 1 | 97 | 1 | 97 | NA | NA | 1 | 97 | G1 |
| TCGA-44-2668 | 51 | MALE | WHITE | T2 | N0 | M0 | I | NA | NA | Yes | NA | No | NA | 1 | 761 | 1 | 761 | 1 | 433 | 1 | 433 | G1 |
| TCGA-44-3398 | 77 | FEMALE | WHITE | T1 | N0 | M0 | I | NA | NA | Yes | NA | No | NA | 0 | 1163 | 0 | 1163 | 0 | 1163 | 0 | 1163 | G1 |
| TCGA-44-3917 | 33 | FEMALE | WHITE | T2 | N0 | M0 | I | NA | NA | Yes | NA | No | NA | 0 | 1183 | 0 | 1183 | 0 | 1183 | 0 | 1183 | G1 |
| TCGA-44-3918 | 60 | FEMALE | WHITE | T1 | N0 | M0 | I | NA | NA | Yes | NA | No | Chemotherapy | 0 | 1036 | 0 | 1036 | 1 | 511 | 1 | 511 | G1 |
| TCGA-44-3919 | 71 | FEMALE | WHITE | T1 | N0 | M0 | I | NA | Recurrence | No | NA | No | NA | 1 | 1026 | 1 | 1026 | 1 | 921 | 1 | 921 | G1 |
| TCGA-44-4112 | 60 | FEMALE | WHITE | T2 | N0 | M0 | I | NA | NA | Yes | NA | No | Chemotherapy | 1 | 808 | 1 | 808 | 1 | 619 | 1 | 619 | G1 |
| TCGA-44-5645 | 61 | FEMALE | BLACK | T1 | NA | NA | I | NA | NA | Yes | NA | No | NA | 0 | 852 | 0 | 852 | 0 | 852 | 0 | 852 | G1 |
| TCGA-44-6146 | 64 | MALE | WHITE | T3 | N0 | M0 | II | NA | Recurrence | Yes | NA | No | Chemotherapy | 0 | 728 | 0 | 728 | 1 | 631 | 1 | 631 | G1 |
| TCGA-44-6147 | 67 | FEMALE | WHITE | T1 | NA | M0 | I | NA | NA | Yes | NA | No | NA | 0 | 845 | 0 | 845 | 0 | 845 | 0 | 845 | G1 |
| TCGA-44-6148 | 60 | MALE | WHITE | T1 | N0 | M0 | I | NA | NA | Yes | NA | No | NA | 0 | 704 | 0 | 704 | 0 | 704 | 0 | 704 | G1 |
| TCGA-44-6775 | 72 | FEMALE | WHITE | T2 | N0 | NA | I | NA | Recurrence | Yes | NA | No | NA | 0 | 705 | 0 | 705 | 1 | 684 | 1 | 684 | G1 |
| TCGA-44-6776 | 60 | FEMALE | WHITE | T1 | N0 | NA | I | NA | NA | Yes | NA | No | NA | 0 | 2616 | 0 | 2616 | 0 | 2616 | 0 | 2616 | G1 |
| TCGA-44-6777 | 85 | FEMALE | WHITE | T2 | NA | NA | I | NA | NA | Yes | NA | No | NA | 1 | 987 | 0 | 987 | 0 | 987 | 0 | 987 | G1 |
| TCGA-44-6778 | 59 | MALE | BLACK | T1 | N0 | NA | I | NA | NA | Yes | NA | No | NA | 0 | 1864 | 0 | 1864 | 0 | 1864 | 0 | 1864 | G1 |
| TCGA-44-7659 | 70 | MALE | WHITE | T1 | N0 | NA | I | NA | NA | Yes | NA | No | NA | 0 | 691 | 0 | 691 | 0 | 691 | 0 | 691 | G1 |
| TCGA-44-7669 | 59 | MALE | BLACK | T1 | N1 | NA | II | NA | NA | Yes | NA | No | Chemotherapy | 1 | 574 | 1 | 574 | 1 | 390 | 1 | 390 | G1 |
| TCGA-44-7671 | 64 | MALE | BLACK | T2 | N0 | M0 | I | NA | Recurrence | Yes | NA | No | NA | 0 | 889 | 0 | 889 | 1 | 883 | 1 | 883 | G1 |
| TCGA-44-8120 | 58 | MALE | BLACK | T2 | N0 | M0 | I | NA | NA | Yes | NO | No | NA | 0 | 260 | 0 | 260 | 0 | 260 | 0 | 260 | G1 |
| TCGA-44-A47A | 78 | FEMALE | WHITE | T2 | N0 | NA | I | NA | Metastasis | Yes | NO | No | NA | 0 | 466 | 0 | 466 | 1 | 397 | 1 | 397 | G1 |
| TCGA-44-A47B | 79 | MALE | WHITE | T2 | N0 | M0 | I | NA | NA | Yes | NO | No | NA | 0 | 287 | 0 | 287 | 0 | 287 | 0 | 287 | G1 |
| TCGA-44-A47G | 73 | FEMALE | WHITE | T1 | N0 | M0 | I | NA | NA | Yes | NO | No | NA | 0 | 351 | 0 | 351 | 0 | 351 | 0 | 351 | G1 |
| TCGA-44-A4SU | 67 | FEMALE | WHITE | T1 | N0 | NA | I | NA | Recurrence | Yes | NO | No | NA | 1 | 409 | 1 | 409 | 1 | 260 | 1 | 260 | G1 |
| TCGA-49-4490 | 45 | FEMALE | WHITE | T3 | N2 | M0 | III | NA | NA | Yes | NA | No | Chemotherapy | 1 | 385 | 1 | 385 | NA | NA | 1 | 385 | G1 |
| TCGA-49-4501 | 67 | FEMALE | WHITE | T2 | N0 | M0 | I | NA | NA | No | NA | No | Chemotherapy | 1 | 1421 | 1 | 1421 | 1 | 545 | 1 | 545 | G1 |
| TCGA-49-4505 | 61 | FEMALE | WHITE | T2 | N1 | M0 | II | NA | NA | Yes | NA | No | NA | 1 | 428 | 1 | 428 | 1 | 417 | 1 | 417 | G1 |
| TCGA-49-4510 | 51 | FEMALE | BLACK | T2 | N1 | M0 | II | NA | Metastasis | Yes | NA | No | NA | 1 | 896 | 1 | 896 | NA | NA | 1 | 502 | G1 |
| TCGA-49-4512 | 69 | FEMALE | WHITE | T2 | N2 | NA | III | NA | NA | No | NA | No | Chemotherapy | 1 | 905 | 1 | 905 | NA | NA | 1 | 905 | G1 |
| TCGA-49-6744 | 64 | FEMALE | WHITE | T2 | N1 | NA | II | NA | NA | Yes | NA | No | Chemotherapy | 0 | 1683 | 0 | 1683 | 0 | 1683 | 0 | 1683 | G1 |
| TCGA-49-AAR0 | 57 | MALE | BLACK | T1 | N0 | NA | I | NA | NA | Yes | NO | No | NA | 0 | 4765 | 0 | 4765 | 0 | 4765 | 0 | 4765 | G1 |
| TCGA-49-AARN | 56 | FEMALE | BLACK | T1 | N0 | NA | I | NA | NA | Yes | NO | No | NA | 1 | 1135 | NA | 1135 | 0 | 1135 | 0 | 1135 | G1 |
| TCGA-49-AARO | 39 | FEMALE | BLACK | T1 | N0 | NA | I | NA | Recurrence | Yes | NO | No | NA | 0 | 3759 | 0 | 3759 | 1 | 1144 | 1 | 1144 | G1 |
| TCGA-49-AARR | 68 | MALE | BLACK | T1 | N0 | NA | I | NA | Primary | Yes | NO | No | NA | 0 | 4992 | 0 | 4992 | 1 | 4812 | 1 | 1099 | G1 |
| TCGA-50-5045 | 57 | FEMALE | BLACK | T2 | N1 | M0 |  | NA | Recurrence | NA | NA | No | NA | 1 | 2174 | 1 | 2174 | NA | NA | 1 | 1433 | G1 |
| TCGA-50-5055 | 79 | FEMALE | WHITE | T1 | N1 | M0 | II | NA | NA | NA | NA | No | NA | 1 | 1830 | 1 | 1830 | NA | NA | 1 | 771 | G1 |
| TCGA-50-5932 | 75 | MALE | WHITE | T2 | N1 | M0 | II | NA | Recurrence | NA | NA | No | NA | 1 | 1235 | 1 | 1235 | NA | NA | 1 | 1090 | G1 |
| TCGA-50-5935 | 86 | FEMALE | WHITE | T1 | N0 | M0 | I | NA | NA | NA | NA | No | NA | 1 | 653 | 0 | 653 | 0 | 653 | 0 | 653 | G1 |
| TCGA-50-5942 | 67 | FEMALE | WHITE | T1 | N0 | M0 | I | NA | Recurrence | Yes | NA | No | NA | 0 | 1847 | 0 | 1847 | NA | NA | 1 | 1378 | G1 |
| TCGA-50-5944 | 69 | FEMALE | WHITE | T1 | N0 | M0 | I | NA | NA | NA | NA | No | NA | 0 | 1750 | 0 | 1750 | 0 | 1750 | 0 | 1750 | G1 |
| TCGA-50-5946 | 62 | MALE | WHITE | T1 | N0 | NA | I | NA | Metastasis | Yes | NA | No | NA | 0 | 1617 | 0 | 1617 | NA | NA | 1 | 221 | G1 |
| TCGA-50-6593 | 49 | FEMALE | WHITE | T1 | N2 | M0 | III | NA | Metastasis | Yes | NA | No | NA | 1 | 336 | 1 | 336 | NA | NA | 1 | 266 | G1 |
| TCGA-50-6673 | 84 | FEMALE | WHITE | T1 | N0 | M0 | I | NA | NA | No | NA | No | NA | 1 | 22 | NA | 22 | NA | NA | 0 | 22 | G1 |
| TCGA-50-8457 | 63 | FEMALE | BLACK | T1 | N0 | M0 | I | NA | NA | Yes | NO | No | NA | 0 | 1125 | 0 | 1125 | 0 | 1125 | 0 | 1125 | G1 |
| TCGA-50-8459 | 68 | MALE | WHITE | T3 | N0 | M0 | II | NA | Recurrence | Yes | NO | No | Chemotherapy | 0 | 1119 | 0 | 1119 | NA | NA | 1 | 432 | G1 |
| TCGA-50-8460 | 74 | MALE | WHITE | T1 | N0 | M0 | I | NA | NA | NA | YES | No | NA | 0 | 829 | 0 | 829 | 0 | 829 | 0 | 829 | G1 |
| TCGA-53-7626 | 76 | FEMALE | WHITE | T1 | N1 | M0 | II | NA | Recurrence | Yes | NA | No | Chemotherapy | 1 | 929 | 1 | 929 | NA | NA | 1 | 865 | G1 |
| TCGA-55-1592 | 65 | MALE | WHITE | T2 | N0 | M0 | I | NA | NA | Yes | NA | No | NA | 1 | 701 | 1 | 701 | 1 | 452 | 1 | 452 | G1 |
| TCGA-55-1595 | 74 | FEMALE | ASIAN | T1 | N0 | M0 | I | NA | NA | Yes | NA | No | NA | 0 | 1479 | 0 | 1479 | 0 | 1479 | 0 | 1479 | G1 |
| TCGA-55-6543 | 60 | FEMALE | WHITE | T1 | N0 | NA | I | NA | NA | Yes | NA | No | NA | 0 | 435 | 0 | 435 | 0 | 435 | 0 | 435 | G1 |
| TCGA-55-6970 | 67 | FEMALE | WHITE | T2 | N2 | NA | III | NA | Recurrence | Yes | NA | No | Chemotherapy | 1 | 464 | 1 | 464 | NA | NA | 1 | 457 | G1 |
| TCGA-55-6971 | 59 | FEMALE | WHITE | T2 | N0 | NA | I | NA | NA | Yes | NA | No | NA | 0 | 1400 | 0 | 1400 | 0 | 1400 | 0 | 1400 | G1 |
| TCGA-55-6980 | 56 | MALE | WHITE | T1 | N0 | M0 | I | NA | NA | No | NA | No | NA | 0 | 2109 | 0 | 2109 | 0 | 2109 | 0 | 2109 | G1 |
| TCGA-55-6981 | 53 | FEMALE | WHITE | T1 | N2 | M0 | III | NA | NA | Yes | NA | No | Chemotherapy | 1 | 1379 | 0 | 1379 | 0 | 1379 | 0 | 1379 | G1 |
| TCGA-55-6985 | 58 | FEMALE | WHITE | T2 | N0 | NA | I | NA | NA | Yes | NA | No | NA | 0 | 1233 | 0 | 1233 | 0 | 1233 | 0 | 1233 | G1 |
| TCGA-55-6986 | 74 | FEMALE | WHITE | T2 | N0 | M0 | I | NA | NA | No | NA | No | NA | 0 | 3261 | 0 | 3261 | 0 | 3261 | 0 | 3261 | G1 |
| TCGA-55-7227 | 77 | MALE | WHITE | T3 | N1 | NA | III | NA | Recurrence | Yes | NA | No | Chemotherapy | 1 | 952 | 1 | 952 | 1 | 255 | 1 | 255 | G1 |
| TCGA-55-7283 | 76 | FEMALE | WHITE | T3 | N2 | NA | III | NA | NA | Yes | NA | No | Chemotherapy | 0 | 609 | 0 | 609 | NA | NA | 0 | 609 | G1 |
| TCGA-55-7284 | 74 | MALE | WHITE | T3 | N0 | NA | II | NA | Metastasis | Yes | NA | No | NA | 1 | 243 | 1 | 243 | 1 | 231 | 1 | 231 | G1 |
| TCGA-55-7573 | 72 | FEMALE | WHITE | T1 | N0 | NA | I | NA | NA | Yes | NA | No | NA | 0 | 487 | 0 | 487 | 0 | 487 | 0 | 487 | G1 |
| TCGA-55-7574 | 64 | FEMALE | WHITE | T2 | N0 | M0 | I | NA | Metastasis | Yes | NA | No | Chemotherapy | 1 | 995 | 1 | 995 | 1 | 478 | 1 | 478 | G1 |
| TCGA-55-7725 | 68 | FEMALE | WHITE | T1 | N0 | NA | I | NA | NA | Yes | NA | No | NA | 0 | 442 | 0 | 442 | 0 | 442 | 0 | 442 | G1 |
| TCGA-55-7727 | 70 | MALE | WHITE | T1 | N2 | NA | III | NA | NA | Yes | NA | No | NA | 0 | 119 | 0 | 119 | 0 | 119 | 0 | 119 | G1 |
| TCGA-55-7728 | 64 | FEMALE | WHITE | T2 | N0 | NA | I | NA | NA | Yes | NA | No | NA | 0 | 704 | 0 | 704 | 0 | 704 | 0 | 704 | G1 |
| TCGA-55-7816 | 49 | FEMALE | WHITE | NA | NA | NA | IV | NA | NA | No | NA | No | NA | 1 | 468 | 1 | 468 | NA | NA | 1 | 468 | G1 |
| TCGA-55-7903 | 64 | MALE | WHITE | T1 | N0 | NA | I | NA | NA | Yes | NA | No | NA | 0 | 567 | 0 | 567 | 0 | 567 | 0 | 567 | G1 |
| TCGA-55-7911 | 70 | FEMALE | WHITE | T1 | N0 | NA | I | NA | Primary | Yes | NA | No | NA | 0 | 537 | 0 | 537 | 0 | 537 | 1 | 515 | G1 |
| TCGA-55-7994 | 81 | MALE | WHITE | T3 | N0 | NA | II | NA | NA | Yes | NA | No | Chemotherapy | 0 | 603 | 0 | 603 | 0 | 603 | 0 | 603 | G1 |
| TCGA-55-8087 | 59 | FEMALE | WHITE | T2 | N0 | NA | I | NA | NA | No | NA | No | NA | 0 | 462 | 0 | 462 | 0 | 462 | 0 | 462 | G1 |
| TCGA-55-8090 | 80 | MALE | WHITE | T1 | N0 | M0 | I | NA | Metastasis | Yes | NA | No | NA | 1 | 598 | 1 | 598 | 1 | 548 | 1 | 548 | G1 |
| TCGA-55-8091 | 74 | MALE | WHITE | T2 | N0 | NA | I | NA | NA | Yes | NA | No | NA | 0 | 600 | 0 | 600 | 0 | 600 | 0 | 600 | G1 |
| TCGA-55-8096 | 67 | FEMALE | WHITE | T2 | N0 | NA | I | NA | Metastasis | Yes | NA | No | NA | 1 | 719 | 1 | 719 | 1 | 566 | 1 | 566 | G1 |
| TCGA-55-8097 | 60 | FEMALE | WHITE | T1 | N0 | NA | I | NA | NA | Yes | NA | No | NA | 0 | 476 | 0 | 476 | 0 | 476 | 0 | 476 | G1 |
| TCGA-55-8206 | 56 | MALE | WHITE | T1 | N0 | M0 | I | NA | NA | No | NO | No | NA | 0 | 888 | 0 | 888 | 0 | 888 | 0 | 888 | G1 |
| TCGA-55-8207 | 73 | MALE | WHITE | T2 | N0 | NA | I | NA | NA | Yes | NO | No | NA | 0 | 977 | 0 | 977 | 0 | 977 | 0 | 977 | G1 |
| TCGA-55-8299 | 61 | FEMALE | WHITE | T1 | N0 | NA | I | NA | Metastasis | Yes | NO | No | NA | 1 | 469 | 1 | 469 | 1 | 274 | 1 | 274 | G1 |
| TCGA-55-8302 | 54 | MALE | WHITE | T2 | N0 | NA | I | NA | NA | Yes | NO | No | NA | 0 | 478 | 0 | 478 | 0 | 478 | 0 | 478 | G1 |
| TCGA-55-8507 | 53 | MALE | WHITE | T1 | N0 | NA | I | NA | NA | Yes | NO | No | NA | 0 | 418 | 0 | 418 | 0 | 418 | 0 | 418 | G1 |
| TCGA-55-8510 | 55 | FEMALE | WHITE | T2 | N0 | NA | I | NA | NA | Yes | NO | No | NA | 0 | 539 | 0 | 539 | 0 | 539 | 0 | 539 | G1 |
| TCGA-55-8512 | 41 | MALE | WHITE | T1 | N1 | M1 | IV | NA | NA | Yes | NA | No | NA | 1 | 607 | 1 | 607 | NA | NA | 1 | 607 | G1 |
| TCGA-55-8513 | 77 | FEMALE | WHITE | T3 | N0 | NA | II | NA | Metastasis | No | NO | No | Chemotherapy | 0 | 791 | 0 | 791 | 1 | 317 | 1 | 317 | G1 |
| TCGA-55-8616 | 58 | FEMALE | WHITE | T2 | N0 | M0 | I | NA | NA | Yes | NA | No | NA | 0 | 48 | 0 | 48 | NA | NA | 0 | 48 | G1 |
| TCGA-55-8619 | 72 | FEMALE | WHITE | T3 | N0 | NA | II | NA | NA | No | NO | No | NA | 0 | 416 | 0 | 416 | 0 | 416 | 0 | 416 | G1 |
| TCGA-55-8621 | 75 | FEMALE | WHITE | T1 | N0 | NA | I | NA | NA | Yes | NO | No | NA | 0 | 515 | 0 | 515 | 0 | 515 | 0 | 515 | G1 |
| TCGA-55-A48X | 63 | FEMALE | WHITE | T1 | N1 | M0 | II | NA | Primary | Yes | NA | No | Chemotherapy | 0 | 689 | 0 | 689 | 0 | 689 | 1 | 615 | G1 |
| TCGA-55-A492 | 70 | FEMALE | WHITE | T1 | N0 | NA | I | NA | NA | Yes | NA | No | NA | 0 | 596 | 0 | 596 | 0 | 596 | 0 | 596 | G1 |
| TCGA-55-A494 | 61 | FEMALE | WHITE | T2 | N0 | NA | I | NA | NA | Yes | NA | No | NA | 0 | 481 | 0 | 481 | 0 | 481 | 0 | 481 | G1 |
| TCGA-55-A4DG | 71 | MALE | WHITE | T1 | N0 | NA | I | NA | NA | Yes | NO | No | NA | 0 | 608 | 0 | 608 | 0 | 608 | 0 | 608 | G1 |
| TCGA-55-A57B | 80 | FEMALE | BLACK | T1 | N0 | M0 | I | NA | NA | No | NA | No | NA | 0 | 546 | 0 | 546 | 0 | 546 | 0 | 546 | G1 |
| TCGA-56-8623 | 71 | MALE | WHITE | T2 | N0 | NA | I | NA | Primary | Yes | NA | No | NA | 1 | 692 | 0 | 692 | 0 | 692 | 1 | 645 | G1 |
| TCGA-56-8626 | 59 | MALE | WHITE | T1 | N0 | NA | I | NA | NA | Yes | NO | No | NA | 1 | 302 | 0 | 302 | 0 | 302 | 0 | 302 | G1 |
| TCGA-56-8628 | 78 | MALE | WHITE | T1 | N0 | NA | I | NA | NA | Yes | NO | No | NA | 0 | 616 | 0 | 616 | 0 | 616 | 0 | 616 | G1 |
| TCGA-56-A4ZJ | 75 | FEMALE | WHITE | T1 | N0 | M0 | I | NA | NA | Yes | NA | No | NA | 0 | 640 | 0 | 640 | 0 | 640 | 0 | 640 | G1 |
| TCGA-60-2697 | 41 | MALE | WHITE | T2 | N2 | M0 | II | NA | NA | NA | NA | Yes | Chemotherapy | 1 | 372 | 1 | 372 | 1 | 200 | 1 | 200 | G1 |
| TCGA-60-2715 | 51 | MALE | WHITE | T1 | N0 | M0 | I | NA | NA | Yes | NA | No | NA | 1 | 1075 | 1 | 1075 | NA | NA | 1 | 1075 | G1 |
| TCGA-62-8395 | 80 | FEMALE | WHITE | T3 | N0 | M0 | II | NA | Recurrence | No | NO | No | NA | 0 | 1216 | 0 | 1216 | 1 | 395 | 1 | 395 | G1 |
| TCGA-62-8397 | 70 | FEMALE | WHITE | T3 | N0 | M0 | II | NA | NA | No | NO | No | NA | 0 | 1289 | 0 | 1289 | 0 | 1289 | 0 | 1289 | G1 |
| TCGA-62-8399 | 62 | MALE | WHITE | T2 | N2 | M0 | III | NA | NA | Yes | NO | No | NA | 0 | 2696 | 0 | 2696 | 0 | 2696 | 0 | 2696 | G1 |
| TCGA-62-A46P | 65 | MALE | WHITE | T2 | N0 | M0 | I | NA | Metastasis | Yes | NO | No | NA | 1 | 594 | 0 | 594 | 1 | 267 | 1 | 267 | G1 |
| TCGA-62-A46R | 54 | FEMALE | WHITE | T2 | N0 | M0 | I | NA | NA | Yes | NO | No | NA | 1 | 1725 | 0 | 1725 | 0 | 1725 | 0 | 1725 | G1 |
| TCGA-62-A46S | 73 | MALE | WHITE | T2 | N0 | M0 | I | NA | NA | Yes | NO | No | Targeted Molecular therapy | 1 | 1653 | 1 | 1653 | 1 | 527 | 1 | 527 | G1 |
| TCGA-62-A46V | 78 | FEMALE | WHITE | T2 | N0 | M0 | I | NA | NA | Yes | NO | No | NA | 0 | 2199 | 0 | 2199 | 0 | 2199 | 0 | 2199 | G1 |
| TCGA-62-A46Y | 70 | FEMALE | WHITE | T2 | N2 | M0 | III | NA | Metastasis | No | YES | No | Chemotherapy | 1 | 414 | 1 | 414 | 1 | 314 | 1 | 314 | G1 |
| TCGA-62-A470 | 84 | MALE | WHITE | T2 | N0 | M0 | I | NA | Recurrence | Yes | NO | No | NA | 1 | 1194 | 1 | 1194 | 1 | 539 | 1 | 539 | G1 |
| TCGA-62-A472 | 70 | MALE | WHITE | T3 | N0 | M0 | II | NA | Recurrence | Yes | NO | No | NA | 0 | 910 | 0 | 910 | 1 | 290 | 1 | 290 | G1 |
| TCGA-64-1680 | 63 | MALE | WHITE | T2 | N2 | M1 | IV | NA | NA | Yes | NA | No | Chemotherapy | 0 | 1126 | 0 | 1126 | NA | NA | 0 | 1126 | G1 |
| TCGA-64-1681 | 61 | FEMALE | WHITE | T1 | N0 | M0 | I | NA | NA | Yes | NA | No | Chemotherapy | 1 | 1167 | NA | 1167 | NA | NA | 1 | 439 | G1 |
| TCGA-64-5815 | 74 | MALE | WHITE | T2 | N1 | M0 | II | NA | NA | Yes | NA | No | Chemotherapy | 0 | 866 | 0 | 866 | 0 | 866 | 0 | 866 | G1 |
| TCGA-67-3770 | 70 | FEMALE | WHITE | T1 | N0 | M0 | I | NA | NA | Yes | NA | No | NA | 0 | 610 | 0 | 610 | NA | NA | 0 | 610 | G1 |
| TCGA-67-3772 | 82 | FEMALE | WHITE | T2 | N0 | M0 | I | NA | NA | No | NA | No | NA | 0 | 573 | 0 | 573 | NA | NA | 0 | 573 | G1 |
| TCGA-67-3773 | 84 | FEMALE | WHITE | T2 | N0 | M0 | I | NA | NA | Yes | NA | No | NA | 0 | 427 | 0 | 427 | NA | NA | 0 | 427 | G1 |
| TCGA-67-3774 | 73 | FEMALE | WHITE | T2 | N0 | M0 | I | NA | NA | Yes | NA | No | NA | 0 | 385 | 0 | 385 | NA | NA | 0 | 385 | G1 |
| TCGA-67-4679 | 69 | MALE | WHITE | T3 | N0 | M0 |  | NA | NA | Yes | NA | No | NA | 0 | 448 | 0 | 448 | NA | NA | 0 | 448 | G1 |
| TCGA-67-6215 | 52 | FEMALE | WHITE | T2 | N0 | M0 | I | NA | NA | No | NA | No | Chemotherapy | 0 | 174 | 0 | 174 | 0 | 174 | 0 | 174 | G1 |
| TCGA-67-6216 | 57 | FEMALE | WHITE | T1 | N0 | M0 | I | NA | NA | No | NA | No | NA | 0 | 141 | 0 | 141 | 0 | 141 | 0 | 141 | G1 |
| TCGA-67-6217 | 73 | FEMALE | WHITE | T2 | N1 | M0 | II | NA | NA | Yes | NA | No | Chemotherapy | 0 | 422 | 0 | 422 | NA | NA | 1 | 296 | G1 |
| TCGA-69-7763 | 69 | MALE | WHITE | T1 | N0 | M0 | I | NA | NA | Yes | NA | No | NA | 0 | 690 | 0 | 690 | NA | NA | 0 | 690 | G1 |
| TCGA-69-7764 | 75 | MALE | WHITE | T1 | N0 | M0 | I | NA | NA | Yes | NA | No | NA | 0 | 414 | 0 | 414 | NA | NA | 0 | 414 | G1 |
| TCGA-69-7765 | 56 | MALE | BLACK | T4 | N0 | NA |  | NA | NA | Yes | NA | No | Chemotherapy | 0 | 165 | 0 | 165 | NA | NA | 0 | 165 | G1 |
| TCGA-69-7973 | 42 | FEMALE | WHITE | T2 | N0 | M0 | I | NA | NA | Yes | NA | No | Chemotherapy | 0 | 230 | 0 | 230 | 0 | 230 | 0 | 230 | G1 |
| TCGA-69-7980 | 70 | FEMALE | WHITE | T1 | N0 | M0 | I | NA | NA | Yes | NA | No | NA | 0 | 411 | 0 | 411 | 0 | 411 | 0 | 411 | G1 |
| TCGA-69-8253 | 59 | FEMALE | BLACK | T1 | N1 | NA | II | NA | NA | Yes | NO | No | Chemotherapy | 0 | 426 | 0 | 426 | 0 | 426 | 0 | 426 | G1 |
| TCGA-69-8254 | 85 | MALE | WHITE | T2 | NA | NA |  | NA | NA | Yes | NO | No | NA | 0 | 409 | 0 | 409 | 0 | 409 | 0 | 409 | G1 |
| TCGA-69-8453 | 77 | MALE | WHITE | T3 | N0 | NA | II | NA | Recurrence | Yes | NO | No | Chemotherapy | 0 | 813 | 0 | 813 | NA | NA | 1 | 344 | G1 |
| TCGA-71-8520 | 60 | FEMALE | ASIAN | T2 | N0 | M0 | I | NA | Recurrence | No | NO | No | NA | 1 | 210 | 1 | 210 | 1 | 179 | 1 | 179 | G1 |
| TCGA-73-4658 | 80 | FEMALE | WHITE | T2 | N0 | M0 | I | NA | NA | Yes | NA | No | NA | 1 | 1600 | 0 | 1600 | 0 | 1600 | 0 | 1600 | G1 |
| TCGA-73-4659 | 66 | MALE | WHITE | T2 | N2 | M0 | III | NA | NA | Yes | NA | No | NA | 1 | 711 | 1 | 711 | NA | NA | 1 | 35 | G1 |
| TCGA-73-4662 | 65 | FEMALE | WHITE | T1 | N0 | M0 | I | NA | NA | Yes | NA | No | NA | 0 | 2515 | 0 | 2515 | 1 | 21 | 1 | 21 | G1 |
| TCGA-73-4677 | 74 | MALE | WHITE | T2 | N0 | M0 |  | NA | NA | Yes | NA | No | NA | 1 | 38 | 1 | 38 | NA | NA | 1 | 38 | G1 |
| TCGA-73-7498 | 58 | FEMALE | WHITE | T1 | N0 | M0 | I | NA | NA | Yes | NO | No | Chemotherapy | 0 | 1189 | 0 | 1189 | 0 | 1189 | 0 | 1189 | G1 |
| TCGA-75-5146 | NA | MALE | NA | T2 | N0 | M0 | I | NA | Primary | Yes | NA | No | NA | 0 | 2368 | 0 | 2368 | 0 | 2368 | 1 | 1773 | G1 |
| TCGA-75-6203 | NA | FEMALE | NA | T2 | N2 | M0 | III | NA | NA | No | NA | No | NA | 0 | NA | 0 | NA | 0 | NA | 0 | NA | G1 |
| TCGA-75-6206 | NA | MALE | NA | T2 | N0 | M0 | I | NA | NA | Yes | NA | No | NA | 0 | 2590 | 0 | 2590 | 0 | 2590 | 0 | 2590 | G1 |
| TCGA-75-6212 | NA | FEMALE | NA | T2 | N1 | M0 | II | NA | Primary | No | NA | No | Chemotherapy | 1 | 1516 | 1 | 1516 | 0 | 1516 | 1 | 1340 | G1 |
| TCGA-75-7025 | NA | MALE | NA | T2 | N0 | M0 | I | NA | NA | Yes | NA | No | NA | 0 | 3305 | 0 | 3305 | 1 | 1481 | 1 | 1481 | G1 |
| TCGA-75-7030 | NA | MALE | NA | T3 | N0 | M0 | II | NA | NA | No | NA | No | Chemotherapy | 0 | NA | 0 | NA | 0 | NA | 0 | NA | G1 |
| TCGA-77-A5FZ | 64 | MALE | NA | T4 | N0 | M0 | III | NA | NA | Yes | NO | No | NA | 1 | 3838 | NA | 3838 | 0 | 3838 | 0 | 3838 | G1 |
| TCGA-78-7143 | 62 | FEMALE | WHITE | T2 | N0 | M0 | I | NA | NA | No | NA | No | NA | 1 | 4961 | NA | 4961 | NA | NA | 1 | 1500 | G1 |
| TCGA-78-7149 | 71 | MALE | WHITE | T4 | N0 | M0 | III | NA | NA | Yes | NA | No | NA | 0 | 3940 | 0 | 3940 | 0 | 3940 | 0 | 3940 | G1 |
| TCGA-78-7152 | 65 | MALE | WHITE | T2 | N0 | M0 | I | NA | NA | Yes | NA | No | NA | 1 | 1215 | 1 | 1215 | 1 | 1202 | 1 | 1202 | G1 |
| TCGA-78-7156 | 62 | MALE | WHITE | T4 | N1 | M1 | IV | NA | NA | Yes | NA | No | NA | 1 | 976 | NA | 976 | NA | NA | 0 | 976 | G1 |
| TCGA-78-7158 | 59 | FEMALE | WHITE | T4 | N2 | M0 | III | NA | NA | Yes | NA | No | Chemotherapy | 1 | 179 | 1 | 179 | NA | NA | 1 | 132 | G1 |
| TCGA-78-7160 | 61 | MALE | WHITE | T4 | N2 | M1 | IV | NA | NA | Yes | NA | No | Chemotherapy | 1 | 697 | NA | 697 | NA | NA | 0 | 697 | G1 |
| TCGA-78-7162 | 75 | MALE | WHITE | T1 | N0 | M0 | I | NA | NA | Yes | NA | No | NA | 1 | 3169 | NA | 3169 | NA | NA | 1 | 2218 | G1 |
| TCGA-78-7167 | 77 | MALE | WHITE | T2 | N0 | M1 | IV | NA | NA | Yes | NA | No | NA | 1 | 2681 | 1 | 2681 | NA | NA | 1 | 726 | G1 |
| TCGA-78-7537 | 72 | MALE | NA | T2 | N0 | M0 | I | NA | NA | Yes | NA | No | NA | 1 | 1622 | NA | 1622 | NA | NA | 0 | 1622 | G1 |
| TCGA-78-7539 | 75 | FEMALE | WHITE | T2 | N0 | M0 | II | NA | Metastasis | Yes | NA | No | Chemotherapy | 0 | 791 | 0 | 791 | 1 | 688 | 1 | 688 | G1 |
| TCGA-78-7633 | 67 | MALE | WHITE | T2 | N0 | M0 | I | NA | Recurrence | Yes | NA | No | NA | 1 | 1528 | 1 | 1528 | 1 | 1450 | 1 | 1450 | G1 |
| TCGA-78-8648 | 58 | FEMALE | NA | T3 | N0 | M0 | II | NA | Recurrence | Yes | NO | No | NA | 1 | 1209 | NA | 1209 | NA | NA | 1 | 720 | G1 |
| TCGA-78-8655 | 77 | FEMALE | NA | T1 | N0 | M0 | I | NA | NA | Yes | NO | No | NA | 0 | 2360 | 0 | 2360 | 0 | 2360 | 0 | 2360 | G1 |
| TCGA-80-5607 | NA | FEMALE | NA | T2 | N1 | M0 | II | NA | NA | Yes | NA | No | NA | 0 | NA | 0 | NA | NA | NA | 0 | NA | G1 |
| TCGA-85-8048 | 62 | MALE | WHITE | T1 | N0 | M0 | I | NA | NA | Yes | NA | No | NA | 0 | 765 | 0 | 765 | 0 | 765 | 0 | 765 | G1 |
| TCGA-85-A4PA | 61 | MALE | WHITE | T2 | N0 | M0 | I | NA | NA | Yes | NA | No | NA | 0 | 741 | 0 | 741 | 0 | 741 | 0 | 741 | G1 |
| TCGA-85-A513 | 60 | FEMALE | ASIAN | T1 | NA | M0 | I | NA | NA | No | NA | No | NA | 0 | 910 | 0 | 910 | 0 | 910 | 0 | 910 | G1 |
| TCGA-86-7714 | 61 | FEMALE | BLACK | T1 | N2 | M0 | III | NA | NA | Yes | NA | No | NA | 1 | 625 | 1 | 625 | NA | NA | 1 | 625 | G1 |
| TCGA-86-8055 | 79 | MALE | WHITE | T2 | N1 | M0 | II | NA | NA | Yes | NA | No | NA | 1 | 124 | 0 | 124 | 0 | 124 | 0 | 124 | G1 |
| TCGA-86-8056 | 63 | FEMALE | WHITE | T4 | N0 | M0 | III | NA | NA | Yes | NA | No | NA | 0 | 139 | 0 | 139 | 0 | 139 | 0 | 139 | G1 |
| TCGA-86-8073 | 58 | MALE | WHITE | T2 | N0 | M0 | I | NA | NA | Yes | NA | No | NA | 0 | 740 | 0 | 740 | 0 | 740 | 0 | 740 | G1 |
| TCGA-86-8074 | 62 | FEMALE | WHITE | T1 | N1 | M0 | II | NA | NA | Yes | NA | No | NA | 0 | 24 | 0 | 24 | NA | NA | 0 | 24 | G1 |
| TCGA-86-8075 | 66 | FEMALE | WHITE | T2 | N0 | M0 | I | NA | Recurrence | No | NA | No | NA | 1 | 694 | 0 | 694 | 1 | 199 | 1 | 199 | G1 |
| TCGA-86-8076 | 42 | MALE | WHITE | T1 | N0 | M0 | I | NA | NA | No | NA | No | Chemotherapy | 0 | 993 | 0 | 993 | 0 | 993 | 0 | 993 | G1 |
| TCGA-86-8278 | 63 | FEMALE | WHITE | T2 | N1 | M0 | II | NA | NA | No | NA | No | NA | 0 | 944 | 0 | 944 | 1 | 29 | 1 | 29 | G1 |
| TCGA-86-8280 | 54 | FEMALE | WHITE | T2 | N0 | M0 | II | NA | NA | No | NA | No | Chemotherapy | 0 | 701 | 0 | 701 | 0 | 701 | 0 | 701 | G1 |
| TCGA-86-8281 | 75 | MALE | WHITE | T1 | NA | M0 | I | NA | NA | Yes | NA | No | NA | 0 | 0 | 0 | 0 | 0 | 0 | 0 | 0 | G1 |
| TCGA-86-8668 | 61 | FEMALE | WHITE | T1 | N0 | M0 | I | NA | NA | No | NA | No | NA | 0 | 423 | 0 | 423 | 0 | 423 | 0 | 423 | G1 |
| TCGA-86-8671 | 72 | FEMALE | WHITE | T2 | N1 | M0 | II | NA | NA | No | NA | No | Chemotherapy | 0 | 839 | 0 | 839 | 0 | 839 | 0 | 839 | G1 |
| TCGA-86-A456 | 78 | FEMALE | ASIAN | T1 | N0 | M0 | I | NA | NA | Yes | NA | No | NA | 0 | 896 | 0 | 896 | 0 | 896 | 0 | 896 | G1 |
| TCGA-86-A4P7 | 63 | FEMALE | WHITE | T2 | N0 | M0 | I | NA | NA | No | NA | No | NA | 0 | 415 | 0 | 415 | 0 | 415 | 0 | 415 | G1 |
| TCGA-86-A4P8 | 59 | FEMALE | WHITE | T1 | N2 | NA | III | NA | NA | No | NA | No | Chemotherapy | 0 | 805 | 0 | 805 | 0 | 805 | 0 | 805 | G1 |
| TCGA-91-6828 | 70 | MALE | WHITE | T1 | N0 | M0 | I | NA | NA | Yes | NA | No | NA | 0 | 323 | 0 | 323 | 0 | 323 | 0 | 323 | G1 |
| TCGA-91-6830 | 65 | FEMALE | WHITE | T1 | N1 | NA | II | NA | Metastasis | Yes | NA | No | NA | 0 | 60 | 0 | 60 | NA | NA | 1 | 18 | G1 |
| TCGA-91-6835 | 81 | FEMALE | WHITE | T1 | N0 | M0 | I | NA | NA | Yes | NA | No | NA | 0 | 79 | 0 | 79 | 0 | 79 | 0 | 79 | G1 |
| TCGA-91-6849 | 75 | FEMALE | BLACK | T2 | N2 | NA | III | NA | NA | Yes | NA | No | NA | 0 | 35 | 0 | 35 | NA | NA | 0 | 35 | G1 |
| TCGA-91-7771 | 62 | MALE | WHITE | T3 | N0 | NA | II | NA | NA | Yes | NA | No | Chemotherapy | 0 | 492 | 0 | 492 | 0 | 492 | 0 | 492 | G1 |
| TCGA-91-8496 | 63 | FEMALE | WHITE | T2 | NA | NA | I | NA | NA | No | NO | No | NA | 0 | 505 | 0 | 505 | 0 | 505 | 0 | 505 | G1 |
| TCGA-91-8497 | 75 | FEMALE | WHITE | T1 | N0 | NA | I | NA | NA | No | NO | No | NA | 1 | 434 | 0 | 434 | 0 | 434 | 0 | 434 | G1 |
| TCGA-91-A4BD | 78 | MALE | WHITE | T1 | N1 | NA | II | NA | NA | Yes | NO | No | NA | 0 | 603 | 0 | 603 | 0 | 603 | 0 | 603 | G1 |
| TCGA-93-7347 | 76 | FEMALE | WHITE | T1 | N0 | NA | I | NA | NA | Yes | NA | No | NA | 0 | 683 | 0 | 683 | 0 | 683 | 0 | 683 | G1 |
| TCGA-93-7348 | 75 | FEMALE | WHITE | T1 | N0 | NA | I | NA | NA | Yes | NA | No | NA | 0 | 531 | 0 | 531 | 0 | 531 | 0 | 531 | G1 |
| TCGA-93-A4JN | 71 | MALE | WHITE | T2 | N0 | M1 | IV | NA | NA | Yes | NO | No | Chemotherapy | 0 | 718 | 0 | 718 | NA | NA | 0 | 718 | G1 |
| TCGA-93-A4JO | 70 | MALE | WHITE | T1 | N0 | NA | I | NA | NA | Yes | NO | No | NA | 1 | 33 | 0 | 33 | NA | NA | 0 | 33 | G1 |
| TCGA-93-A4JP | 64 | MALE | ASIAN | NA | NA | M1 | IV | NA | Metastasis | No | NO | No | Chemotherapy | 0 | 578 | 0 | 578 | NA | NA | 1 | 497 | G1 |
| TCGA-93-A4JQ | 49 | MALE | WHITE | T1 | N0 | NA | I | NA | NA | Yes | NO | No | NA | 0 | 526 | 0 | 526 | 0 | 526 | 0 | 526 | G1 |
| TCGA-95-7039 | 54 | FEMALE | WHITE | T3 | N0 | NA | II | NA | Recurrence | Yes | NA | No | NA | 0 | 1272 | 0 | 1272 | 1 | 1258 | 1 | 1258 | G1 |
| TCGA-95-7947 | 67 | MALE | WHITE | T1 | N0 | M0 | I | NA | NA | Yes | NA | No | NA | 0 | 477 | 0 | 477 | 0 | 477 | 0 | 477 | G1 |
| TCGA-95-8039 | 72 | MALE | WHITE | T1 | N0 | NA | I | NA | Recurrence | No | NO | No | NA | 0 | 830 | 0 | 830 | 1 | 228 | 1 | 228 | G1 |
| TCGA-95-A4VK | 74 | FEMALE | WHITE | T2 | N2 | M0 | III | NA | Metastasis | Yes | YES | No | Chemotherapy | 0 | 651 | 0 | 651 | NA | NA | 1 | 496 | G1 |
| TCGA-95-A4VP | 66 | FEMALE | WHITE | T2 | N2 | M0 | III | NA | Metastasis | Yes | NO | No | Chemotherapy | 0 | 605 | 0 | 605 | NA | NA | 1 | 216 | G1 |
| TCGA-97-7546 | 76 | FEMALE | WHITE | T1 | N0 | NA | I | NA | Primary | Yes | NA | No | Chemotherapy | 0 | 1285 | 0 | 1285 | NA | NA | 1 | 1255 | G1 |
| TCGA-97-7547 | 67 | FEMALE | WHITE | T2 | N0 | NA | I | NA | NA | Yes | NA | No | Chemotherapy | 0 | 1965 | 0 | 1965 | NA | NA | 1 | 775 | G1 |
| TCGA-97-7552 | 70 | MALE | WHITE | T2 | N0 | NA | I | NA | NA | Yes | NA | No | NA | 0 | 1932 | 0 | 1932 | NA | NA | 1 | 798 | G1 |
| TCGA-97-7553 | 58 | FEMALE | WHITE | T1 | N0 | NA | I | NA | NA | Yes | NA | No | NA | 0 | 1870 | 0 | 1870 | 0 | 1870 | 0 | 1870 | G1 |
| TCGA-97-7938 | 76 | FEMALE | WHITE | T1 | N0 | NA | I | NA | NA | Yes | NA | No | NA | 1 | 18 | 0 | 18 | 0 | 18 | 0 | 18 | G1 |
| TCGA-97-7941 | 72 | FEMALE | WHITE | T1 | N0 | NA | I | NA | NA | Yes | NA | No | NA | 0 | 484 | 0 | 484 | 0 | 484 | 0 | 484 | G1 |
| TCGA-97-8171 | 81 | MALE | ASIAN | T2 | N2 | M1 | IV | NA | Metastasis | Yes | NA | No | Targeted Molecular therapy | 0 | 568 | 0 | 568 | NA | NA | 1 | 441 | G1 |
| TCGA-97-8172 | 75 | FEMALE | WHITE | T2 | N0 | M0 | I | NA | NA | Yes | NO | No | NA | 0 | 545 | 0 | 545 | 0 | 545 | 0 | 545 | G1 |
| TCGA-97-8174 | 67 | MALE | WHITE | T2 | N0 | M0 | II | NA | NA | Yes | NO | No | Chemotherapy | 1 | 164 | NA | 164 | NA | NA | 0 | 164 | G1 |
| TCGA-97-8175 | 55 | FEMALE | WHITE | T2 | N0 | M0 | I | NA | Recurrence | Yes | NO | No | Chemotherapy | 0 | 551 | 0 | 551 | 1 | 294 | 1 | 294 | G1 |
| TCGA-97-8177 | 59 | FEMALE | WHITE | T2 | N0 | M0 | I | NA | NA | No | NO | No | NA | 0 | 499 | 0 | 499 | NA | NA | 0 | 499 | G1 |
| TCGA-97-8179 | 72 | MALE | WHITE | T1 | N0 | M0 | I | NA | NA | Yes | NO | No | NA | 0 | 435 | 0 | 435 | 0 | 435 | 0 | 435 | G1 |
| TCGA-97-8547 | 78 | FEMALE | NA | T2 | N2 | NA | III | NA | NA | No | NO | No | NA | 0 | 657 | 0 | 657 | 0 | 657 | 0 | 657 | G1 |
| TCGA-97-8552 | 55 | FEMALE | WHITE | T1 | N0 | NA | I | NA | NA | No | NO | No | NA | 0 | 626 | 0 | 626 | 0 | 626 | 0 | 626 | G1 |
| TCGA-97-A4LX | 81 | MALE | WHITE | T2 | N0 | M0 | I | NA | NA | Yes | NO | No | NA | 0 | 614 | 0 | 614 | 0 | 614 | 0 | 614 | G1 |
| TCGA-97-A4M0 | 60 | FEMALE | WHITE | T2 | N0 | M0 | I | NA | NA | Yes | NO | No | NA | 0 | 652 | 0 | 652 | 0 | 652 | 0 | 652 | G1 |
| TCGA-97-A4M1 | 52 | FEMALE | WHITE | T1 | N0 | M0 | I | NA | NA | Yes | NO | No | NA | 0 | 601 | 0 | 601 | 0 | 601 | 0 | 601 | G1 |
| TCGA-97-A4M2 | 66 | MALE | WHITE | T1 | N0 | M0 | I | NA | NA | Yes | NO | No | NA | 0 | 624 | 0 | 624 | 0 | 624 | 0 | 624 | G1 |
| TCGA-97-A4M3 | 69 | FEMALE | WHITE | T1 | N0 | M0 | I | NA | Primary | Yes | NO | No | NA | 0 | 540 | 0 | 540 | 0 | 540 | 1 | 54 | G1 |
| TCGA-97-A4M5 | 83 | MALE | WHITE | T1 | N0 | M0 | I | NA | NA | Yes | NO | No | NA | 0 | 634 | 0 | 634 | 0 | 634 | 0 | 634 | G1 |
| TCGA-97-A4M6 | 45 | FEMALE | WHITE | T1 | N0 | M0 | I | NA | NA | No | NO | No | NA | 0 | 568 | 0 | 568 | 0 | 568 | 0 | 568 | G1 |
| TCGA-97-A4M7 | 74 | MALE | WHITE | T1 | N0 | M0 | I | NA | NA | Yes | NO | No | NA | 0 | 629 | 0 | 629 | 0 | 629 | 0 | 629 | G1 |
| TCGA-98-7454 | 73 | MALE | WHITE | T2 | N0 | M0 | I | NA | NA | Yes | NA | No | NA | 0 | 652 | 0 | 652 | 0 | 652 | 0 | 652 | G1 |
| TCGA-98-A53C | 77 | FEMALE | WHITE | T1 | N0 | M0 | I | NA | NA | Yes | NO | No | NA | 0 | 822 | 0 | 822 | 0 | 822 | 0 | 822 | G1 |
| TCGA-98-A53D | 68 | MALE | WHITE | T3 | N0 | M0 | II | NA | Recurrence | Yes | NO | No | NA | 1 | 645 | 1 | 645 | 1 | 413 | 1 | 413 | G1 |
| TCGA-98-A53H | 76 | FEMALE | WHITE | T1 | N0 | M0 | I | NA | Primary | Yes | NO | No | NA | 0 | 618 | 0 | 618 | 0 | 618 | 1 | 427 | G1 |
| TCGA-99-7458 | 74 | FEMALE | WHITE | T4 | N0 | M0 | III | NA | NA | Yes | NA | No | Chemotherapy | 0 | 747 | 0 | 747 | NA | NA | 0 | 747 | G1 |
| TCGA-99-8025 | 72 | FEMALE | BLACK | T3 | N2 | M0 | III | NA | NA | Yes | NO | No | Chemotherapy | 0 | 1060 | 0 | 1060 | 0 | 1060 | 0 | 1060 | G1 |
| TCGA-99-AA5R | 70 | FEMALE | BLACK | T1 | N0 | M0 | I | NA | NA | Yes | NO | No | NA | 0 | 658 | 0 | 658 | 0 | 658 | 0 | 658 | G1 |
| TCGA-J2-8192 | 65 | FEMALE | WHITE | T2 | N1 | NA | II | NA | Recurrence | No | NO | No | Chemotherapy | 0 | 739 | 0 | 739 | NA | NA | 1 | 482 | G1 |
| TCGA-J2-8194 | 69 | FEMALE | WHITE | T3 | N0 | NA | II | NA | Metastasis | Yes | NO | No | NA | 0 | 724 | 0 | 724 | NA | NA | 1 | 470 | G1 |
| TCGA-J2-A4AD | 61 | FEMALE | WHITE | T1 | N0 | NA | I | NA | NA | Yes | NO | No | NA | 1 | 550 | 1 | 550 | NA | NA | 1 | 524 | G1 |
| TCGA-J2-A4AE | 77 | FEMALE | WHITE | T1 | N0 | NA | I | NA | NA | No | NO | No | NA | 0 | 1079 | 0 | 1079 | 0 | 1079 | 0 | 1079 | G1 |
| TCGA-J2-A4AG | 66 | FEMALE | WHITE | T1 | N0 | NA | I | NA | NA | Yes | NO | No | NA | 0 | 988 | 0 | 988 | 0 | 988 | 0 | 988 | G1 |
| TCGA-L4-A4E6 | 67 | MALE | WHITE | T1 | N0 | M0 | I | NA | NA | Yes | NO | No | NA | 0 | 435 | 0 | 435 | 0 | 435 | 0 | 435 | G1 |
| TCGA-L9-A443 | 63 | FEMALE | WHITE | T1 | N0 | NA | I | NA | NA | Yes | NO | No | NA | 1 | 193 | 0 | 193 | 0 | 193 | 0 | 193 | G1 |
| TCGA-L9-A50W | 75 | MALE | BLACK | T1 | N1 | NA | II | NA | Metastasis | Yes | NO | No | NA | 1 | 442 | 1 | 442 | 1 | 389 | 1 | 389 | G1 |
| TCGA-L9-A743 | 56 | MALE | BLACK | T2 | N1 | M0 | II | NA | NA | Yes | NO | No | Chemotherapy | 0 | 664 | 0 | 664 | 0 | 664 | 0 | 664 | G1 |
| TCGA-L9-A7SV | 69 | MALE | BLACK | T2 | N1 | M0 | II | NA | NA | Yes | NO | No | Chemotherapy | 0 | 565 | 0 | 565 | 0 | 565 | 0 | 565 | G1 |
| TCGA-MN-A4N5 | 63 | MALE | WHITE | T1 | N0 | M0 | I | NA | NA | Yes | NO | No | NA | 0 | 84 | 0 | 84 | 0 | 84 | 0 | 84 | G1 |
| TCGA-MP-A4SV | 67 | MALE | NA | T2 | N0 | M0 | I | NA | NA | Yes | NO | No | NA | 1 | 2620 | NA | 2620 | 0 | 2620 | 0 | 2620 | G1 |
| TCGA-MP-A4SW | 53 | MALE | WHITE | T2 | N1 | M0 | II | NA | NA | Yes | NO | No | NA | 1 | 1778 | NA | 1778 | 0 | 1778 | 0 | 1778 | G1 |
| TCGA-MP-A4SY | 61 | MALE | WHITE | T2 | N1 | M0 | II | NA | Recurrence | Yes | NO | No | NA | 1 | 1501 | 0 | 1501 | 1 | 489 | 1 | 489 | G1 |
| TCGA-MP-A4T6 | 76 | FEMALE | WHITE | T1 | N2 | NA | III | NA | NA | Yes | NO | No | NA | 1 | 1790 | 0 | 1790 | 0 | 1790 | 0 | 1790 | G1 |
| TCGA-MP-A4T7 | 75 | FEMALE | NA | T2 | N0 | M1 | IV | NA | NA | Yes | NO | No | NA | 1 | 167 | NA | 167 | NA | NA | 0 | 167 | G1 |
| TCGA-MP-A4T9 | 54 | FEMALE | WHITE | T2 | N2 | NA | III | NA | NA | Yes | YES | No | Chemotherapy | 1 | 1265 | 1 | 1265 | 1 | 341 | 1 | 341 | G1 |
| TCGA-MP-A4TH | 70 | FEMALE | WHITE | T1 | N0 | M0 | I | NA | NA | Yes | NO | No | NA | 0 | 741 | 0 | 741 | 0 | 741 | 0 | 741 | G1 |
| TCGA-MP-A4TJ | 62 | FEMALE | NA | T1 | N0 | M0 | I | NA | NA | Yes | NO | No | NA | 1 | 339 | NA | 339 | 0 | 339 | 0 | 339 | G1 |
| TCGA-MP-A4TK | 56 | FEMALE | NA | T2 | N1 | NA | II | NA | Metastasis | Yes | NO | No | NA | 1 | 582 | 1 | 582 | 1 | 397 | 1 | 397 | G1 |
| TCGA-MP-A5C7 | 76 | FEMALE | WHITE | T2 | N0 | M0 | I | NA | NA | Yes | NO | No | NA | 0 | 2248 | 0 | 2248 | 0 | 2248 | 0 | 2248 | G1 |
| TCGA-NC-A5HJ | 59 | MALE | WHITE | T3 | N0 | M0 | II | NA | Primary | Yes | NO | No | Chemotherapy | 1 | 418 | 1 | 418 | 0 | 418 | 1 | 328 | G1 |
| TCGA-NJ-A4YG | 65 | MALE | WHITE | T2 | N0 | M0 | I | NA | NA | Yes | NO | No | NA | 0 | 2261 | 0 | 2261 | NA | NA | 0 | 2261 | G1 |
| TCGA-NJ-A4YI | 87 | FEMALE | WHITE | T2 | N2 | M0 | III | NA | NA | Yes | NO | No | NA | 1 | 4 | NA | 4 | NA | NA | 0 | 4 | G1 |
| TCGA-NJ-A4YP | 52 | MALE | WHITE | T2 | N0 | M0 | I | NA | NA | Yes | NO | No | NA | 0 | 50 | 0 | 50 | NA | NA | 0 | 50 | G1 |
| TCGA-NJ-A55A | 76 | FEMALE | WHITE | T2 | N0 | M0 | I | NA | NA | Yes | NO | No | NA | 0 | 15 | 0 | 15 | NA | NA | 0 | 15 | G1 |
| TCGA-NJ-A55O | 56 | FEMALE | WHITE | T1 | N1 | M0 | II | NA | NA | Yes | NO | No | NA | 0 | 13 | 0 | 13 | NA | NA | 0 | 13 | G1 |
| TCGA-NJ-A7XG | 49 | MALE | BLACK | T4 | N1 | M0 | III | NA | NA | Yes | NO | No | Chemotherapy | 0 | 617 | 0 | 617 | NA | NA | 0 | 617 | G1 |
| TCGA-O1-A52J | 74 | FEMALE | WHITE | T1 | N0 | NA | I | NA | Recurrence | Yes | NO | No | NA | 1 | 1798 | 1 | 1798 | NA | NA | 1 | 898 | G1 |
| TCGA-S2-AA1A | 68 | FEMALE | BLACK | T1 | N0 | M0 | I | NA | NA | Yes | NO | No | NA | 0 | 513 | 0 | 513 | 0 | 513 | 0 | 513 | G1 |
| TCGA-05-4250 | 79 | FEMALE | NA | T3 | N1 | M0 | III | NA | NA | Yes | NA | No | NA | 1 | 121 | NA | 121 | NA | NA | 0 | 121 | G2 |
| TCGA-05-4382 | 68 | MALE | NA | T2 | N0 | M0 | I | NA | Metastasis | Yes | NA | No | NA | 0 | 607 | 0 | 607 | 1 | 334 | 1 | 334 | G2 |
| TCGA-05-4389 | 70 | MALE | NA | T1 | N0 | M0 | I | NA | NA | Yes | NA | No | NA | 0 | 1369 | 0 | 1369 | NA | NA | 0 | 1369 | G2 |
| TCGA-05-4390 | 58 | FEMALE | NA | T2 | N0 | M0 | I | NA | NA | Yes | NA | No | Chemotherapy | 0 | 1126 | 0 | 1126 | NA | NA | 1 | 395 | G2 |
| TCGA-05-4395 | 76 | MALE | NA | T4 | N2 | M0 | III | NA | NA | Yes | NA | No | NA | 1 | 0 | 0 | 0 | NA | NA | 0 | 0 | G2 |
| TCGA-05-4397 | 65 | MALE | NA | T2 | N1 | M0 | II | NA | NA | Yes | NA | No | NA | 1 | 731 | NA | 731 | NA | NA | 0 | 731 | G2 |
| TCGA-05-4398 | 47 | FEMALE | NA | T4 | N3 | M0 | III | NA | NA | Yes | NA | No | Chemotherapy | 0 | 1431 | 0 | 1431 | 0 | 1431 | 0 | 1431 | G2 |
| TCGA-05-4402 | 57 | FEMALE | NA | T2 | NA | M1 | IV | NA | NA | No | NA | No | NA | 1 | 244 | 0 | 244 | NA | NA | 0 | 244 | G2 |
| TCGA-05-4410 | 62 | MALE | NA | T2 | N0 | M0 | I | NA | NA | Yes | NA | No | NA | 0 | 0 | 0 | 0 | NA | NA | 0 | 0 | G2 |
| TCGA-05-4415 | 57 | MALE | NA | T4 | N2 | M0 | III | NA | NA | Yes | NA | No | NA | 1 | 91 | 1 | 91 | NA | NA | 1 | 60 | G2 |
| TCGA-05-4417 | 51 | FEMALE | NA | T2 | N0 | M0 | I | NA | NA | Yes | NA | No | NA | 0 | 455 | 0 | 455 | NA | NA | 0 | 455 | G2 |
| TCGA-05-4418 | 69 | MALE | NA | T3 | N2 | M0 | III | NA | NA | Yes | NA | No | NA | 1 | 274 | NA | 274 | NA | NA | 0 | 274 | G2 |
| TCGA-05-4420 | 41 | MALE | NA | T2 | N0 | M0 | I | NA | NA | Yes | NA | No | NA | 0 | 912 | 0 | 912 | 0 | 912 | 0 | 912 | G2 |
| TCGA-05-4425 | 70 | FEMALE | NA | T2 | N0 | M1 | IV | NA | NA | Yes | NA | No | Chemotherapy | 0 | 669 | 0 | 669 | NA | NA | 0 | 669 | G2 |
| TCGA-05-4427 | 65 | FEMALE | NA | T2 | N1 | M0 | II | NA | NA | Yes | NA | No | Chemotherapy | 0 | 791 | 0 | 791 | 0 | 791 | 0 | 791 | G2 |
| TCGA-05-4432 | 66 | MALE | NA | T2 | N1 | M0 | II | NA | NA | Yes | NA | No | Chemotherapy | 0 | 761 | 0 | 761 | 0 | 761 | 0 | 761 | G2 |
| TCGA-05-4434 | 67 | FEMALE | NA | T4 | N1 | M1 | IV | NA | NA | Yes | NA | No | NA | 1 | 457 | NA | 457 | NA | NA | 0 | 457 | G2 |
| TCGA-05-5420 | 67 | MALE | NA | T2 | N2 | M0 | III | NA | NA | Yes | NA | No | NA | 0 | 457 | 0 | 457 | NA | NA | 1 | 245 | G2 |
| TCGA-05-5425 | 68 | MALE | NA | T2 | N1 | M0 | II | NA | NA | Yes | NA | No | NA | 0 | 882 | 0 | 882 | NA | NA | 1 | 486 | G2 |
| TCGA-05-5428 | 57 | MALE | NA | T1 | N1 | M0 | II | NA | NA | Yes | NA | No | Chemotherapy | 0 | 670 | 0 | 670 | 0 | 670 | 0 | 670 | G2 |
| TCGA-05-5429 | 60 | MALE | NA | T3 | N2 | M0 | III | NA | NA | No | NA | No | NA | 1 | 275 | 0 | 275 | NA | NA | 0 | 275 | G2 |
| TCGA-18-3406 | 67 | MALE | WHITE | T1 | N0 | M0 | I | NA | Recurrence | Yes | NA | No | NA | 1 | 371 | 1 | 371 | NA | NA | 1 | 357 | G2 |
| TCGA-18-3407 | 72 | MALE | ASIAN | T2 | N0 | M0 | I | NA | NA | Yes | NA | No | NA | 1 | 136 | 0 | 136 | NA | NA | 0 | 136 | G2 |
| TCGA-18-3408 | 77 | FEMALE | WHITE | T2 | N0 | M0 | I | NA | Recurrence | Yes | NA | No | NA | 1 | 2304 | 1 | 2304 | NA | NA | 1 | 1793 | G2 |
| TCGA-18-3409 | 74 | MALE | WHITE | T1 | N0 | M0 | I | NA | Primary | Yes | NA | No | NA | 0 | 3747 | 0 | 3747 | 0 | 3747 | 1 | 2291 | G2 |
| TCGA-18-3410 | 81 | MALE | NA | T3 | N0 | M0 | II | NA | NA | Yes | NA | No | NA | 1 | 146 | 0 | 146 | NA | NA | 0 | 146 | G2 |
| TCGA-18-3411 | 63 | FEMALE | NA | T2 | N2 | M0 | III | NA | NA | Yes | NA | No | NA | 0 | 3576 | 0 | 3576 | NA | NA | 0 | 3576 | G2 |
| TCGA-18-3412 | 52 | MALE | WHITE | T2 | N0 | M0 | I | NA | Metastasis | Yes | NA | No | NA | 1 | 345 | 1 | 345 | NA | NA | 1 | 273 | G2 |
| TCGA-18-3414 | 73 | MALE | ASIAN | T4 | N1 | M1 | IV | NA | NA | Yes | NA | No | NA | 1 | 716 | 0 | 716 | NA | NA | 0 | 716 | G2 |
| TCGA-18-3415 | 77 | MALE | NA | T2 | N0 | M0 | I | NA | NA | Yes | NA | No | NA | 1 | 2803 | 0 | 2803 | 0 | 2803 | 0 | 2803 | G2 |
| TCGA-18-3416 | 83 | MALE | NA | T2 | N1 | M0 | II | NA | Metastasis | Yes | NA | No | NA | 1 | 973 | 1 | 973 | 1 | 940 | 1 | 940 | G2 |
| TCGA-18-3417 | 65 | MALE | NA | T2 | N1 | M1 | IV | NA | NA | Yes | NA | No | Chemotherapy | 1 | 1097 | 0 | 1097 | NA | NA | 0 | 1097 | G2 |
| TCGA-18-3419 | 73 | MALE | NA | T2 | N1 | M0 | II | NA | NA | Yes | NA | No | NA | 0 | 2811 | 0 | 2811 | 0 | 2811 | 0 | 2811 | G2 |
| TCGA-18-3421 | 65 | MALE | WHITE | T2 | N0 | M0 | I | NA | NA | Yes | NA | No | NA | 0 | 2645 | 0 | 2645 | 0 | 2645 | 0 | 2645 | G2 |
| TCGA-18-4083 | 63 | MALE | WHITE | T2 | N1 | M0 | II | NA | NA | Yes | NA | No | Chemotherapy | 1 | 188 | 0 | 188 | NA | NA | 0 | 188 | G2 |
| TCGA-18-4086 | 64 | MALE | NA | T2 | N0 | M0 | I | NA | NA | Yes | NA | No | NA | 1 | 85 | 0 | 85 | NA | NA | 0 | 85 | G2 |
| TCGA-18-4721 | 74 | MALE | WHITE | T1 | N0 | M0 | I | NA | NA | Yes | NA | No | NA | 0 | 4694 | 0 | 4694 | 0 | 4694 | 0 | 4694 | G2 |
| TCGA-18-5592 | 57 | MALE | NA | T3 | N0 | M0 | II | NA | NA | Yes | NA | No | NA | 0 | 1519 | 0 | 1519 | NA | NA | 0 | 1519 | G2 |
| TCGA-18-5595 | 50 | MALE | NA | T2 | N0 | M0 | I | NA | NA | No | NA | No | NA | 1 | 827 | NA | 827 | NA | NA | 0 | 827 | G2 |
| TCGA-21-1070 | 60 | FEMALE | BLACK | T3 | N0 | M0 | III | NA | NA | Yes | NA | No | NA | 0 | 3636 | 0 | 3636 | NA | NA | 0 | 3636 | G2 |
| TCGA-21-1071 | 67 | MALE | WHITE | T2 | N0 | M0 | I | NA | NA | Yes | NA | No | NA | 1 | 1426 | 1 | 1426 | NA | NA | 1 | 993 | G2 |
| TCGA-21-1072 | 75 | MALE | WHITE | T2 | N0 | M0 | I | NA | NA | Yes | NA | No | NA | 0 | 3016 | 0 | 3016 | NA | NA | 0 | 3016 | G2 |
| TCGA-21-1075 | 57 | MALE | WHITE | T2 | N1 | M0 | II | NA | NA | Yes | NA | No | NA | 0 | 2134 | 0 | 2134 | NA | NA | 0 | 2134 | G2 |
| TCGA-21-1076 | 54 | FEMALE | WHITE | T2 | N0 | M0 | I | NA | NA | Yes | NA | No | NA | 0 | 1852 | 0 | 1852 | NA | NA | 1 | 1575 | G2 |
| TCGA-21-1077 | 64 | MALE | WHITE | T2 | N1 | M0 | II | NA | NA | Yes | NA | No | NA | 1 | 1058 | 1 | 1058 | NA | NA | 1 | 644 | G2 |
| TCGA-21-1078 | 77 | MALE | WHITE | T2 | N0 | M0 | I | NA | NA | Yes | NA | No | NA | 1 | 474 | 1 | 474 | NA | NA | 1 | 257 | G2 |
| TCGA-21-1079 | 71 | MALE | WHITE | T3 | N0 | M0 | III | NA | NA | Yes | NA | No | NA | 1 | 965 | 1 | 965 | NA | NA | 1 | 317 | G2 |
| TCGA-21-1080 | 66 | MALE | WHITE | T2 | N0 | M0 | I | NA | NA | Yes | NA | No | NA | 0 | 3724 | 0 | 3724 | NA | NA | 0 | 3724 | G2 |
| TCGA-21-1081 | 69 | MALE | WHITE | T2 | N1 | M0 | II | NA | NA | Yes | NA | No | NA | 1 | 284 | 1 | 284 | NA | NA | 1 | 284 | G2 |
| TCGA-21-1082 | 61 | MALE | WHITE | T2 | N0 | M0 | I | NA | NA | NA | NA | No | NA | 0 | 3644 | 0 | 3644 | NA | NA | 0 | 3644 | G2 |
| TCGA-21-1083 | 75 | MALE | WHITE | T1 | N0 | M0 | I | NA | NA | Yes | NA | No | NA | 1 | 1315 | 0 | 1315 | NA | NA | 0 | 1315 | G2 |
| TCGA-21-5782 | 68 | FEMALE | WHITE | T2 | N0 | M0 | I | NA | NA | Yes | NA | No | NA | 1 | 962 | NA | 962 | 0 | 962 | 0 | 962 | G2 |
| TCGA-21-5783 | 76 | MALE | WHITE | T2 | N0 | M0 | I | NA | NA | Yes | NA | No | NA | 1 | 2680 | NA | 2680 | NA | NA | 0 | 2680 | G2 |
| TCGA-21-5784 | 80 | FEMALE | WHITE | T2 | N0 | M0 | I | NA | NA | Yes | NA | No | NA | 0 | 1268 | 0 | 1268 | 0 | 1268 | 0 | 1268 | G2 |
| TCGA-21-5786 | 64 | MALE | WHITE | T2 | N0 | M0 | I | NA | Recurrence | Yes | NA | No | NA | 0 | 1032 | 0 | 1032 | 1 | 817 | 1 | 817 | G2 |
| TCGA-21-5787 | 65 | MALE | BLACK | T2 | N2 | M0 | III | NA | NA | Yes | NA | No | NA | 1 | 329 | 1 | 329 | NA | NA | 1 | 103 | G2 |
| TCGA-21-A5DI | 77 | MALE | WHITE | T1 | N0 | M0 | I | NA | NA | Yes | NO | No | NA | 0 | 979 | 0 | 979 | 0 | 979 | 0 | 979 | G2 |
| TCGA-22-0940 | 71 | MALE | WHITE | T1 | N1 | M0 | II | NA | NA | Yes | NA | No | NA | 1 | 669 | 1 | 669 | NA | NA | 1 | 669 | G2 |
| TCGA-22-0944 | 61 | MALE | WHITE | T2 | N0 | M0 | I | NA | NA | Yes | NA | No | NA | 1 | 223 | 0 | 223 | NA | NA | 0 | 223 | G2 |
| TCGA-22-1000 | 76 | MALE | WHITE | T2 | N0 | M0 | I | NA | NA | Yes | NA | No | NA | 1 | 454 | 1 | 454 | 1 | 263 | 1 | 263 | G2 |
| TCGA-22-1005 | 63 | MALE | WHITE | T1 | N0 | M0 | I | NA | NA | Yes | NA | No | NA | 1 | 1953 | 0 | 1953 | 0 | 1953 | 0 | 1953 | G2 |
| TCGA-22-1011 | 73 | MALE | WHITE | T2 | N0 | M0 | I | NA | NA | Yes | NA | No | NA | 1 | 53 | 0 | 53 | NA | NA | 0 | 53 | G2 |
| TCGA-22-1012 | 80 | FEMALE | WHITE | T2 | N0 | M0 | I | NA | NA | Yes | NA | No | NA | 1 | 429 | 0 | 429 | 0 | 429 | 0 | 429 | G2 |
| TCGA-22-1017 | 62 | MALE | WHITE | T1 | N0 | M0 | I | NA | NA | Yes | NA | Yes | NA | 1 | 1485 | 1 | 1485 | 1 | 904 | 1 | 904 | G2 |
| TCGA-22-4591 | 80 | MALE | WHITE | T3 | N2 | M0 | III | NA | NA | Yes | NA | No | Chemotherapy | 1 | 623 | 1 | 623 | NA | NA | 1 | 443 | G2 |
| TCGA-22-4593 | 77 | MALE | WHITE | T2 | N0 | M0 | II | NA | NA | Yes | NA | No | NA | 1 | 1067 | 0 | 1067 | NA | NA | 0 | 1067 | G2 |
| TCGA-22-4594 | 60 | FEMALE | NA | T3 | N2 | M0 | III | NA | NA | Yes | NA | No | NA | 1 | 1470 | NA | 1470 | NA | NA | 0 | 1470 | G2 |
| TCGA-22-4595 | 57 | MALE | WHITE | T3 | N2 | NA | III | NA | NA | Yes | NA | No | Chemotherapy | 1 | 734 | 1 | 734 | 1 | 250 | 1 | 250 | G2 |
| TCGA-22-4596 | 69 | FEMALE | WHITE | T1 | N0 | M0 | I | NA | NA | Yes | NA | No | NA | 1 | 17 | 0 | 17 | NA | NA | 0 | 17 | G2 |
| TCGA-22-4599 | 73 | FEMALE | WHITE | T2 | N0 | M0 | I | NA | NA | Yes | NA | No | NA | 1 | 1161 | NA | 1161 | NA | NA | 0 | 1161 | G2 |
| TCGA-22-4601 | 73 | FEMALE | WHITE | T4 | N0 | M0 | III | NA | NA | Yes | NA | No | NA | 1 | 1057 | 1 | 1057 | NA | NA | 1 | 525 | G2 |
| TCGA-22-4604 | 73 | MALE | WHITE | T2 | N1 | M0 | II | NA | NA | Yes | NA | No | Chemotherapy | 1 | 399 | 1 | 399 | NA | NA | 1 | 306 | G2 |
| TCGA-22-4605 | 78 | FEMALE | NA | T2 | N0 | M0 | I | NA | NA | Yes | NA | No | NA | 1 | 974 | 0 | 974 | 0 | 974 | 0 | 974 | G2 |
| TCGA-22-4607 | 75 | MALE | WHITE | T2 | N0 | M0 | I | NA | NA | Yes | NA | No | NA | 1 | 587 | 0 | 587 | NA | NA | 0 | 587 | G2 |
| TCGA-22-4609 | 81 | MALE | WHITE | T1 | N0 | M0 | I | NA | NA | Yes | NA | No | NA | 1 | 291 | 0 | 291 | 0 | 291 | 0 | 291 | G2 |
| TCGA-22-4613 | 73 | FEMALE | WHITE | T1 | N0 | M0 | I | NA | NA | Yes | NA | No | NA | 1 | 358 | NA | 358 | NA | NA | 0 | 358 | G2 |
| TCGA-22-5471 | 75 | MALE | WHITE | T2 | N0 | M0 | I | NA | NA | Yes | NA | No | NA | 0 | 1845 | 0 | 1845 | 1 | 229 | 1 | 229 | G2 |
| TCGA-22-5473 | 78 | MALE | WHITE | T3 | N0 | M0 |  | NA | NA | Yes | NA | No | Chemotherapy | 1 | 1933 | 0 | 1933 | 1 | 1655 | 1 | 1655 | G2 |
| TCGA-22-5474 | 74 | MALE | WHITE | T2 | N0 | M0 | I | NA | NA | Yes | NA | No | NA | 1 | 445 | 0 | 445 | 0 | 445 | 0 | 445 | G2 |
| TCGA-22-5477 | 65 | MALE | WHITE | T1 | N0 | M0 | I | NA | NA | Yes | NA | No | NA | 1 | 1346 | NA | 1346 | 1 | 511 | 1 | 511 | G2 |
| TCGA-22-5478 | 79 | MALE | NA | T2 | N0 | M0 | I | NA | NA | Yes | NA | No | NA | 1 | 24 | 0 | 24 | 0 | 24 | 0 | 24 | G2 |
| TCGA-22-5479 | 64 | MALE | WHITE | T2 | N0 | M0 | I | NA | NA | Yes | NA | No | Chemotherapy | 1 | 2625 | NA | 2625 | NA | NA | 0 | 2625 | G2 |
| TCGA-22-5480 | 66 | FEMALE | WHITE | T1 | N0 | M0 | I | NA | NA | Yes | NA | No | Chemotherapy | 1 | 2170 | 1 | 2170 | 1 | 1779 | 1 | 1779 | G2 |
| TCGA-22-5481 | 72 | FEMALE | WHITE | T2 | N1 | M0 | II | NA | NA | Yes | NA | No | NA | 1 | 2409 | NA | 2409 | 1 | 346 | 1 | 346 | G2 |
| TCGA-22-5482 | 81 | MALE | WHITE | T2 | N0 | M0 | I | NA | NA | Yes | NA | No | NA | 1 | 357 | 0 | 357 | 0 | 357 | 0 | 357 | G2 |
| TCGA-22-5483 | 74 | MALE | WHITE | T1 | N1 | M0 | II | NA | NA | Yes | NA | No | Chemotherapy | 1 | 573 | 1 | 573 | 1 | 568 | 1 | 568 | G2 |
| TCGA-22-5485 | 58 | FEMALE | WHITE | T1 | N0 | M0 | I | NA | NA | Yes | NA | No | NA | 1 | 916 | 1 | 916 | 1 | 840 | 1 | 840 | G2 |
| TCGA-22-5489 | 64 | MALE | WHITE | T1 | N0 | M0 | I | NA | NA | Yes | NA | No | Chemotherapy | 1 | 1912 | 1 | 1912 | 1 | 428 | 1 | 428 | G2 |
| TCGA-22-5491 | 74 | MALE | WHITE | T1 | N0 | M0 | I | NA | NA | Yes | NA | No | NA | 1 | 1713 | NA | 1713 | NA | NA | 0 | 1713 | G2 |
| TCGA-22-5492 | 73 | FEMALE | NA | T2 | N2 | M0 | III | NA | NA | Yes | NA | No | NA | 1 | 506 | NA | 506 | NA | NA | 0 | 506 | G2 |
| TCGA-22-A5C4 | 70 | MALE | WHITE | T2 | N0 | M0 | II | NA | NA | Yes | NO | No | NA | 0 | 671 | 0 | 671 | 0 | 671 | 0 | 671 | G2 |
| TCGA-33-4532 | 68 | MALE | WHITE | T2 | N0 | M0 | I | NA | NA | Yes | NA | No | NA | 1 | 3924 | 0 | 3924 | 0 | 3924 | 0 | 3924 | G2 |
| TCGA-33-4533 | 76 | FEMALE | BLACK | T2 | N0 | M0 | I | NA | NA | Yes | NA | No | NA | 0 | 4068 | 0 | 4068 | 0 | 4068 | 0 | 4068 | G2 |
| TCGA-33-4538 | 66 | MALE | WHITE | T2 | N2 | M0 | III | NA | NA | Yes | NA | Yes | NA | 1 | 2979 | NA | 2979 | 0 | 2979 | 0 | 2979 | G2 |
| TCGA-33-4547 | 68 | MALE | WHITE | T2 | N0 | M0 | I | NA | NA | Yes | NA | No | NA | 0 | 2419 | 0 | 2419 | 0 | 2419 | 0 | 2419 | G2 |
| TCGA-33-4566 | 40 | MALE | WHITE | T2 | N0 | M0 | I | NA | NA | Yes | NA | No | NA | 1 | 5287 | NA | 5287 | NA | NA | 0 | 5287 | G2 |
| TCGA-33-4582 | 55 | MALE | WHITE | T1 | N0 | M0 | I | NA | Recurrence | Yes | NA | No | NA | 1 | 3149 | 1 | 3149 | 1 | 2923 | 1 | 2923 | G2 |
| TCGA-33-4583 | 73 | MALE | WHITE | T1 | N0 | M0 | I | NA | NA | Yes | NA | No | NA | 1 | 4601 | 0 | 4601 | 0 | 4601 | 0 | 4601 | G2 |
| TCGA-33-4586 | 57 | MALE | WHITE | T2 | N2 | M0 | III | NA | Recurrence | Yes | NA | No | NA | 1 | 428 | NA | 428 | 1 | 169 | 1 | 169 | G2 |
| TCGA-33-4587 | 63 | FEMALE | WHITE | T2 | N0 | NA | I | NA | NA | Yes | NA | No | Chemotherapy | 1 | 1656 | 1 | 1656 | NA | NA | 1 | 1656 | G2 |
| TCGA-33-4589 | 62 | FEMALE | WHITE | T2 | N1 | M0 | II | NA | NA | Yes | NA | No | NA | 1 | 47 | 0 | 47 | 0 | 47 | 0 | 47 | G2 |
| TCGA-33-6737 | 71 | MALE | WHITE | T2 | N2 | M0 | III | NA | NA | Yes | NA | No | Chemotherapy | 1 | 601 | 1 | 601 | NA | NA | 1 | 601 | G2 |
| TCGA-33-6738 | 80 | MALE | WHITE | T1 | N2 | NA | III | NA | NA | Yes | NA | No | Chemotherapy | 0 | 1927 | 0 | 1927 | 0 | 1927 | 0 | 1927 | G2 |
| TCGA-33-A4WN | 60 | MALE | WHITE | T2 | N0 | NA | I | NA | NA | Yes | NA | No | NA | 1 | 143 | 0 | 143 | 0 | 143 | 0 | 143 | G2 |
| TCGA-33-A5GW | 67 | MALE | WHITE | T1 | N1 | NA | II | NA | NA | Yes | NA | No | NA | 1 | 9 | 0 | 9 | 0 | 9 | 0 | 9 | G2 |
| TCGA-33-AAS8 | 59 | FEMALE | BLACK | T1 | N0 | NA | I | NA | NA | Yes | NO | No | NA | 1 | 1114 | 0 | 1114 | 0 | 1114 | 0 | 1114 | G2 |
| TCGA-33-AASB | 66 | MALE | BLACK | T2 | N0 | NA | I | NA | Metastasis | Yes | NO | No | NA | 1 | 211 | 1 | 211 | NA | NA | 1 | 30 | G2 |
| TCGA-33-AASD | 83 | MALE | BLACK | T1 | N0 | NA | I | NA | Recurrence | Yes | NO | No | NA | 1 | 3376 | 1 | 3376 | NA | NA | 1 | 1912 | G2 |
| TCGA-33-AASI | 65 | FEMALE | BLACK | T2 | N1 | NA | II | NA | NA | Yes | YES | No | Chemotherapy | 1 | 1344 | 1 | 1344 | NA | NA | 1 | 1344 | G2 |
| TCGA-33-AASJ | 60 | MALE | BLACK | T2 | N0 | NA | I | NA | Recurrence | Yes | NO | No | NA | 1 | 3600 | 1 | 3600 | 1 | 1135 | 1 | 1135 | G2 |
| TCGA-33-AASL | 57 | FEMALE | BLACK | T1 | N0 | NA | I | NA | Recurrence | Yes | NO | No | NA | 1 | 826 | 1 | 826 | NA | NA | 1 | 149 | G2 |
| TCGA-34-2596 | 70 | MALE | WHITE | T2 | N1 | M0 | II | NA | NA | Yes | NA | No | NA | 1 | 80 | 0 | 80 | 0 | 80 | 0 | 80 | G2 |
| TCGA-34-2600 | 76 | FEMALE | WHITE | T1 | N0 | M0 | I | NA | NA | Yes | NA | No | NA | 1 | 1874 | 0 | 1874 | 0 | 1874 | 0 | 1874 | G2 |
| TCGA-34-2608 | 84 | MALE | WHITE | T2 | N0 | M0 | I | NA | NA | Yes | NA | No | NA | 1 | 1000 | 0 | 1000 | 0 | 1000 | 0 | 1000 | G2 |
| TCGA-34-5231 | 72 | MALE | WHITE | T1 | N0 | M0 | I | NA | NA | Yes | NA | No | NA | 1 | 1984 | 0 | 1984 | NA | NA | 0 | 1984 | G2 |
| TCGA-34-5232 | 75 | FEMALE | BLACK | T1 | N1 | M0 | II | NA | NA | NA | NA | No | NA | 0 | 2471 | 0 | 2471 | 0 | 2471 | 0 | 2471 | G2 |
| TCGA-34-5234 | 71 | FEMALE | WHITE | T1 | N0 | M0 | I | NA | NA | Yes | NA | No | NA | 0 | 2271 | 0 | 2271 | 0 | 2271 | 0 | 2271 | G2 |
| TCGA-34-5236 | 60 | MALE | WHITE | T3 | N0 | M0 | II | NA | Recurrence | NA | NA | No | NA | 1 | 276 | 1 | 276 | NA | NA | 1 | 113 | G2 |
| TCGA-34-5239 | 75 | MALE | WHITE | T4 | N0 | M0 | III | NA | Metastasis | NA | NA | No | Chemotherapy | 0 | 1834 | 0 | 1834 | 1 | 1530 | 1 | 1530 | G2 |
| TCGA-34-5240 | 73 | FEMALE | WHITE | T2 | N1 | M0 | II | NA | NA | Yes | NA | No | Chemotherapy | 0 | 1541 | 0 | 1541 | 0 | 1541 | 0 | 1541 | G2 |
| TCGA-34-5241 | 79 | MALE | WHITE | T2 | N0 | M0 | I | NA | NA | Yes | NA | No | NA | 1 | 515 | 0 | 515 | NA | NA | 0 | 515 | G2 |
| TCGA-34-5927 | 70 | FEMALE | WHITE | T1 | N0 | M0 | I | NA | Metastasis | NA | NA | No | Chemotherapy | 0 | 1361 | 0 | 1361 | NA | NA | 1 | 245 | G2 |
| TCGA-34-5928 | 83 | FEMALE | WHITE | T2 | N1 | M0 | II | NA | NA | Yes | NA | No | NA | 0 | 1196 | 0 | 1196 | 0 | 1196 | 0 | 1196 | G2 |
| TCGA-34-5929 | 78 | FEMALE | WHITE | T2 | N0 | M0 | I | NA | NA | Yes | NA | No | NA | 1 | 151 | 0 | 151 | NA | NA | 0 | 151 | G2 |
| TCGA-34-7107 | 70 | MALE | WHITE | T2 | N0 | M0 | II | NA | NA | Yes | NA | No | NA | 1 | 34 | NA | 34 | NA | NA | 0 | 34 | G2 |
| TCGA-34-8454 | 62 | FEMALE | WHITE | T3 | N1 | M0 | III | NA | NA | Yes | NO | No | Chemotherapy | 0 | 1180 | 0 | 1180 | 0 | 1180 | 0 | 1180 | G2 |
| TCGA-34-8455 | 67 | MALE | WHITE | T4 | N0 | M1 | IV | NA | NA | Yes | NO | No | NA | 1 | 123 | 1 | 123 | NA | NA | 1 | 123 | G2 |
| TCGA-34-8456 | 71 | FEMALE | WHITE | T2 | N1 | M0 | II | NA | NA | Yes | NO | No | NA | 0 | 804 | 0 | 804 | 0 | 804 | 0 | 804 | G2 |
| TCGA-34-A5IX | 80 | MALE | WHITE | T3 | N0 | M0 | II | NA | NA | Yes | NO | No | NA | 0 | 1031 | 0 | 1031 | 0 | 1031 | 0 | 1031 | G2 |
| TCGA-35-3615 | 57 | MALE | WHITE | T2 | N0 | M0 | I | NA | NA | No | NA | No | NA | 0 | 14 | 0 | 14 | NA | NA | 0 | 14 | G2 |
| TCGA-35-4122 | 69 | MALE | WHITE | T1 | N0 | M0 | I | NA | NA | Yes | NA | No | NA | 0 | 225 | 0 | 225 | NA | NA | 0 | 225 | G2 |
| TCGA-35-4123 | 38 | MALE | WHITE | T1 | N0 | M0 | I | NA | NA | Yes | NA | No | NA | 0 | 182 | 0 | 182 | NA | NA | 0 | 182 | G2 |
| TCGA-35-5375 | 61 | MALE | WHITE | T2 | N2 | M0 | III | NA | NA | Yes | NA | No | NA | 0 | 264 | 0 | 264 | NA | NA | 0 | 264 | G2 |
| TCGA-37-3783 | 51 | MALE | WHITE | T3 | N2 | M0 | III | NA | NA | NA | NA | No | NA | 0 | 122 | 0 | 122 | NA | NA | 0 | 122 | G2 |
| TCGA-37-3789 | 65 | MALE | WHITE | T2 | N0 | NA | I | NA | NA | No | NA | No | NA | 0 | 13 | 0 | 13 | NA | NA | 0 | 13 | G2 |
| TCGA-37-3792 | 69 | MALE | WHITE | T2 | N0 | M0 | I | NA | NA | No | NA | No | NA | 0 | 12 | 0 | 12 | NA | NA | 0 | 12 | G2 |
| TCGA-37-4129 | 52 | FEMALE | WHITE | T1 | N0 | M0 | I | NA | NA | Yes | NA | No | NA | 0 | 242 | 0 | 242 | 0 | 242 | 0 | 242 | G2 |
| TCGA-37-4130 | 56 | MALE | WHITE | T1 | N0 | M0 | I | NA | NA | Yes | NA | No | NA | 0 | 247 | 0 | 247 | NA | NA | 0 | 247 | G2 |
| TCGA-37-4132 | 61 | FEMALE | WHITE | T2 | N0 | M1 | IV | NA | NA | No | NA | No | NA | 0 | 227 | 0 | 227 | NA | NA | 0 | 227 | G2 |
| TCGA-37-4133 | 63 | MALE | WHITE | T4 | N0 | M0 | III | NA | NA | Yes | NA | No | NA | 0 | 238 | 0 | 238 | NA | NA | 0 | 238 | G2 |
| TCGA-37-4135 | 68 | MALE | WHITE | T2 | N0 | M0 | I | NA | NA | Yes | NA | No | NA | 0 | 207 | 0 | 207 | NA | NA | 0 | 207 | G2 |
| TCGA-37-4141 | 65 | FEMALE | WHITE | T1 | N0 | M0 | I | NA | NA | No | NA | No | NA | 0 | 12 | 0 | 12 | NA | NA | 0 | 12 | G2 |
| TCGA-37-5819 | 64 | MALE | WHITE | T2 | N2 | M0 | III | NA | NA | Yes | NA | No | NA | 0 | 103 | 0 | 103 | NA | NA | 0 | 103 | G2 |
| TCGA-37-A5EL | 53 | MALE | WHITE | T3 | N0 | M0 | II | NA | Metastasis | NA | YES | No | Chemotherapy | 1 | 1143 | 1 | 1143 | NA | NA | 1 | 946 | G2 |
| TCGA-37-A5EM | 49 | MALE | WHITE | T2 | N0 | M0 | II | NA | NA | NA | NO | No | NA | 0 | 867 | 0 | 867 | 0 | 867 | 0 | 867 | G2 |
| TCGA-37-A5EN | 59 | MALE | WHITE | T4 | N2 | M0 | III | NA | NA | NA | NO | No | NA | 0 | 660 | 0 | 660 | 0 | 660 | 0 | 660 | G2 |
| TCGA-38-4625 | 66 | FEMALE | WHITE | T2 | N0 | M0 | I | NA | NA | Yes | NA | No | NA | 0 | 2973 | 0 | 2973 | 0 | 2973 | 0 | 2973 | G2 |
| TCGA-38-4628 | 65 | FEMALE | WHITE | T2 | N1 | M0 | II | NA | NA | No | NA | No | Chemotherapy | 1 | 1492 | 1 | 1492 | 1 | 1083 | 1 | 1083 | G2 |
| TCGA-38-4629 | 68 | MALE | WHITE | T3 | N0 | M0 | II | NA | NA | Yes | NA | No | NA | 1 | 864 | 1 | 864 | 1 | 379 | 1 | 379 | G2 |
| TCGA-38-4630 | 75 | FEMALE | WHITE | T2 | N0 | M0 | I | NA | NA | No | NA | No | NA | 1 | 1073 | 1 | 1073 | 1 | 524 | 1 | 524 | G2 |
| TCGA-38-4631 | 72 | FEMALE | WHITE | T2 | N0 | M0 | I | NA | NA | Yes | NA | No | NA | 1 | 354 | 1 | 354 | NA | NA | 1 | 354 | G2 |
| TCGA-38-4632 | 42 | MALE | BLACK | T2 | N1 | M1 | IV | NA | NA | Yes | NA | No | Chemotherapy | 1 | 1357 | 1 | 1357 | NA | NA | 1 | 680 | G2 |
| TCGA-39-5011 | 70 | FEMALE | WHITE | T1 | N0 | M0 | I | NA | NA | Yes | NA | No | Chemotherapy | 0 | 4053 | 0 | 4053 | 1 | 565 | 1 | 565 | G2 |
| TCGA-39-5016 | 44 | MALE | WHITE | T2 | N1 | M0 | II | NA | NA | Yes | NA | No | NA | 0 | 3850 | 0 | 3850 | 0 | 3850 | 0 | 3850 | G2 |
| TCGA-39-5019 | 70 | MALE | WHITE | T2 | N0 | M0 | I | NA | NA | Yes | NA | No | NA | 0 | 3387 | 0 | 3387 | 1 | 378 | 1 | 378 | G2 |
| TCGA-39-5021 | 70 | MALE | WHITE | T2 | N0 | M0 | I | NA | NA | Yes | NA | No | Chemotherapy | 1 | 2086 | 1 | 2086 | 1 | 1875 | 1 | 1875 | G2 |
| TCGA-39-5022 | 76 | MALE | WHITE | T2 | N0 | M0 | I | NA | NA | Yes | NA | No | NA | 1 | 1679 | 0 | 1679 | NA | NA | 1 | 1448 | G2 |
| TCGA-39-5024 | 65 | FEMALE | WHITE | T2 | N2 | M0 | III | NA | NA | Yes | NA | No | Chemotherapy | 0 | 2510 | 0 | 2510 | 0 | 2510 | 0 | 2510 | G2 |
| TCGA-39-5027 | 73 | MALE | WHITE | T2 | N0 | M0 | I | NA | Primary | Yes | NA | No | NA | 0 | 3108 | 0 | 3108 | 0 | 3108 | 1 | 3081 | G2 |
| TCGA-39-5028 | 75 | MALE | WHITE | T4 | N1 | M0 | III | NA | NA | Yes | NA | No | NA | 1 | 52 | NA | 52 | NA | NA | 0 | 52 | G2 |
| TCGA-39-5029 | 67 | MALE | WHITE | T1 | N2 | M0 | III | NA | NA | Yes | NA | No | Chemotherapy | 1 | 740 | 1 | 740 | NA | NA | 1 | 491 | G2 |
| TCGA-39-5030 | 81 | FEMALE | ASIAN | T2 | N2 | M0 | III | NA | NA | Yes | NA | No | NA | 1 | 59 | NA | 59 | 0 | 59 | 0 | 59 | G2 |
| TCGA-39-5031 | 76 | FEMALE | NA | T1 | N0 | M0 | I | NA | NA | Yes | NA | No | NA | 1 | 1841 | 0 | 1841 | 0 | 1841 | 0 | 1841 | G2 |
| TCGA-39-5034 | 73 | FEMALE | WHITE | T3 | N0 | M0 | II | NA | NA | Yes | NA | No | NA | 1 | 1107 | NA | 1107 | 1 | 352 | 1 | 352 | G2 |
| TCGA-39-5035 | 72 | FEMALE | WHITE | T1 | N0 | M0 | I | NA | NA | Yes | NA | No | NA | 0 | 2080 | 0 | 2080 | 0 | 2080 | 0 | 2080 | G2 |
| TCGA-39-5036 | 73 | MALE | WHITE | T2 | N0 | M0 | I | NA | NA | Yes | NA | No | NA | 0 | 2165 | 0 | 2165 | 0 | 2165 | 0 | 2165 | G2 |
| TCGA-39-5037 | 65 | MALE | WHITE | T1 | N1 | M0 | II | NA | NA | Yes | NA | No | Chemotherapy | 0 | 1690 | 0 | 1690 | 0 | 1690 | 0 | 1690 | G2 |
| TCGA-39-5039 | 76 | MALE | WHITE | T2 | N0 | M0 | II | NA | NA | Yes | NA | No | NA | 1 | 544 | 0 | 544 | 0 | 544 | 0 | 544 | G2 |
| TCGA-39-5040 | 59 | MALE | WHITE | T2 | N2 | M0 | III | NA | NA | Yes | NA | No | Chemotherapy | 1 | 519 | 1 | 519 | NA | NA | 1 | 442 | G2 |
| TCGA-43-2576 | 62 | FEMALE | WHITE | T2 | N2 | M0 | III | NA | NA | Yes | NA | No | Chemotherapy | 0 | 1223 | 0 | 1223 | 0 | 1223 | 0 | 1223 | G2 |
| TCGA-43-2578 | 59 | FEMALE | WHITE | T1 | N0 | M0 | I | NA | NA | Yes | NA | No | NA | 1 | 684 | 0 | 684 | 0 | 684 | 0 | 684 | G2 |
| TCGA-43-2581 | 47 | FEMALE | WHITE | T3 | N1 | M0 | III | NA | NA | Yes | NA | No | Chemotherapy | 0 | 1176 | 0 | 1176 | 0 | 1176 | 0 | 1176 | G2 |
| TCGA-43-3394 | 52 | MALE | BLACK | T2 | N0 | M0 | I | NA | NA | Yes | NA | No | Chemotherapy | 1 | 1190 | 1 | 1190 | 1 | 552 | 1 | 552 | G2 |
| TCGA-43-3920 | 71 | MALE | WHITE | T2 | N0 | M0 | I | NA | NA | Yes | NA | No | NA | 0 | 1007 | 0 | 1007 | 0 | 1007 | 0 | 1007 | G2 |
| TCGA-43-5668 | 78 | MALE | WHITE | T1 | N1 | M0 | II | NA | NA | Yes | NA | No | NA | 1 | 559 | 1 | 559 | 1 | 470 | 1 | 470 | G2 |
| TCGA-43-5670 | 70 | MALE | WHITE | T2 | N0 | M0 | II | NA | NA | Yes | NA | No | Chemotherapy | 0 | 849 | 0 | 849 | 0 | 849 | 0 | 849 | G2 |
| TCGA-43-6143 | 70 | MALE | WHITE | T2 | N0 | M0 | I | NA | NA | Yes | NA | No | NA | 0 | 699 | 0 | 699 | 0 | 699 | 0 | 699 | G2 |
| TCGA-43-6647 | 69 | FEMALE | WHITE | T2 | N1 | NA | II | NA | NA | Yes | NA | No | Chemotherapy | 0 | 757 | 0 | 757 | 0 | 757 | 0 | 757 | G2 |
| TCGA-43-6770 | 59 | FEMALE | BLACK | T2 | N0 | NA | I | NA | NA | Yes | NA | No | Vaccine | 0 | 653 | 0 | 653 | 0 | 653 | 0 | 653 | G2 |
| TCGA-43-6771 | 85 | MALE | WHITE | T2 | N0 | NA | I | NA | NA | Yes | NA | No | NA | 1 | 166 | 1 | 166 | NA | NA | 1 | 154 | G2 |
| TCGA-43-6773 | 76 | MALE | WHITE | T2 | N1 | NA | II | NA | NA | Yes | NA | No | NA | 1 | 116 | 0 | 116 | NA | NA | 0 | 116 | G2 |
| TCGA-43-7656 | 71 | MALE | WHITE | T1 | N0 | NA | I | NA | NA | Yes | NA | No | NA | 0 | 596 | 0 | 596 | 0 | 596 | 0 | 596 | G2 |
| TCGA-43-7657 | 68 | FEMALE | WHITE | T1 | N0 | NA | I | NA | NA | Yes | NA | No | NA | 0 | 236 | 0 | 236 | 0 | 236 | 0 | 236 | G2 |
| TCGA-43-7658 | 75 | FEMALE | WHITE | T1 | N0 | M0 | I | NA | NA | Yes | NA | No | NA | 1 | 2378 | 1 | 2378 | 1 | 2305 | 1 | 2305 | G2 |
| TCGA-43-8115 | 72 | FEMALE | WHITE | T2 | N1 | NA | II | NA | NA | Yes | NO | No | Chemotherapy | 0 | 407 | 0 | 407 | NA | NA | 0 | 407 | G2 |
| TCGA-43-8116 | 73 | MALE | WHITE | T1 | N0 | M0 | I | NA | NA | Yes | NO | No | NA | 0 | 358 | 0 | 358 | 0 | 358 | 0 | 358 | G2 |
| TCGA-43-8118 | 55 | FEMALE | WHITE | T1 | N0 | M0 | I | NA | NA | Yes | NO | No | Chemotherapy | 1 | 89 | NA | 89 | NA | NA | 0 | 89 | G2 |
| TCGA-43-A474 | 66 | MALE | WHITE | T2 | N0 | M0 | II | NA | NA | Yes | NO | No | Chemotherapy | 0 | 353 | 0 | 353 | 0 | 353 | 0 | 353 | G2 |
| TCGA-43-A475 | 67 | FEMALE | WHITE | T3 | N0 | M0 | II | NA | NA | Yes | YES | No | Chemotherapy | 0 | 296 | 0 | 296 | 0 | 296 | 0 | 296 | G2 |
| TCGA-43-A56U | 76 | FEMALE | WHITE | T1 | N0 | NA | I | NA | NA | Yes | NO | No | NA | 0 | 432 | 0 | 432 | 0 | 432 | 0 | 432 | G2 |
| TCGA-43-A56V | 61 | MALE | BLACK | T2 | N2 | M0 | III | NA | NA | Yes | NO | No | Chemotherapy | 0 | 366 | 0 | 366 | 1 | 355 | 1 | 355 | G2 |
| TCGA-44-3396 | 74 | FEMALE | WHITE | T2 | N2 | M0 | III | NA | NA | Yes | NA | No | Chemotherapy | 0 | 1130 | 0 | 1130 | 0 | 1130 | 0 | 1130 | G2 |
| TCGA-44-5643 | 53 | MALE | BLACK | T2 | N2 | M0 | III | NA | NA | Yes | NA | No | Chemotherapy | 0 | 1013 | 0 | 1013 | NA | NA | 0 | 1013 | G2 |
| TCGA-44-5644 | 51 | FEMALE | WHITE | T2 | N0 | NA | I | NA | NA | Yes | NA | No | NA | 0 | 863 | 0 | 863 | 0 | 863 | 0 | 863 | G2 |
| TCGA-44-6145 | 62 | FEMALE | WHITE | T1 | N0 | M0 | I | NA | NA | Yes | NA | No | NA | 0 | 595 | 0 | 595 | 0 | 595 | 0 | 595 | G2 |
| TCGA-44-6774 | 56 | FEMALE | WHITE | T1 | N2 | M0 | III | NA | NA | Yes | NA | No | Chemotherapy | 0 | 658 | 0 | 658 | 0 | 658 | 0 | 658 | G2 |
| TCGA-44-6779 | 50 | FEMALE | WHITE | T2 | N1 | NA | II | NA | NA | Yes | NA | No | Chemotherapy | 1 | 500 | 1 | 500 | NA | NA | 1 | 232 | G2 |
| TCGA-44-7660 | 72 | MALE | WHITE | T2 | N0 | NA | I | NA | Recurrence | Yes | NA | No | Vaccine | 0 | 592 | 0 | 592 | NA | NA | 1 | 253 | G2 |
| TCGA-44-7661 | 69 | FEMALE | WHITE | T2 | N0 | M0 | I | NA | Recurrence | Yes | NA | No | NA | 1 | 557 | 1 | 557 | 1 | 335 | 1 | 335 | G2 |
| TCGA-44-7662 | 61 | MALE | WHITE | T2 | N0 | NA | I | NA | NA | Yes | NA | No | NA | 0 | 218 | 0 | 218 | 0 | 218 | 0 | 218 | G2 |
| TCGA-44-7667 | 49 | FEMALE | WHITE | T3 | N0 | NA | II | NA | NA | Yes | NA | No | NA | 0 | 1097 | 0 | 1097 | 0 | 1097 | 0 | 1097 | G2 |
| TCGA-44-7670 | 47 | FEMALE | WHITE | T1 | N1 | M0 | II | NA | NA | Yes | NA | No | Chemotherapy | 0 | 882 | 0 | 882 | 0 | 882 | 0 | 882 | G2 |
| TCGA-44-7672 | 52 | FEMALE | WHITE | T1 | N0 | M0 | I | NA | NA | Yes | NA | No | NA | 0 | 719 | 0 | 719 | 0 | 719 | 0 | 719 | G2 |
| TCGA-44-8117 | 54 | FEMALE | WHITE | T2 | N0 | M0 | I | NA | NA | Yes | NO | No | Chemotherapy | 0 | 385 | 0 | 385 | 0 | 385 | 0 | 385 | G2 |
| TCGA-44-8119 | 73 | MALE | WHITE | T3 | N0 | M0 | II | NA | NA | Yes | NO | No | NA | 0 | 285 | 0 | 285 | 0 | 285 | 0 | 285 | G2 |
| TCGA-44-A479 | 73 | FEMALE | BLACK | T2 | N0 | NA | I | NA | Primary | Yes | NO | No | NA | 0 | 486 | 0 | 486 | 0 | 486 | 1 | 437 | G2 |
| TCGA-44-A4SS | 73 | MALE | WHITE | T1 | N0 | M0 | I | NA | NA | Yes | NO | No | NA | 0 | 415 | 0 | 415 | 0 | 415 | 0 | 415 | G2 |
| TCGA-46-3765 | 59 | FEMALE | WHITE | T1 | N0 | M0 | I | NA | NA | Yes | NA | No | NA | 0 | 405 | 0 | 405 | 0 | 405 | 0 | 405 | G2 |
| TCGA-46-3766 | 62 | FEMALE | WHITE | T1 | N0 | M0 | I | NA | NA | Yes | NA | No | NA | 0 | 370 | 0 | 370 | 0 | 370 | 0 | 370 | G2 |
| TCGA-46-3767 | 76 | MALE | WHITE | T1 | N0 | M0 | I | NA | NA | Yes | NA | No | NA | 0 | 396 | 0 | 396 | 0 | 396 | 0 | 396 | G2 |
| TCGA-46-3768 | 58 | MALE | WHITE | T3 | N1 | M0 | III | NA | NA | Yes | NA | No | Chemotherapy | 1 | 299 | 0 | 299 | 0 | 299 | 0 | 299 | G2 |
| TCGA-46-3769 | 57 | MALE | WHITE | T4 | N0 | M0 |  | NA | NA | Yes | NA | No | Chemotherapy | 0 | 135 | 0 | 135 | 0 | 135 | 0 | 135 | G2 |
| TCGA-46-6025 | 71 | MALE | WHITE | T2 | N1 | M0 | II | NA | NA | Yes | NA | No | Chemotherapy | 0 | 324 | 0 | 324 | 0 | 324 | 0 | 324 | G2 |
| TCGA-46-6026 | 81 | MALE | WHITE | T2 | N1 | M0 | II | NA | NA | Yes | NA | No | Chemotherapy | 0 | 423 | 0 | 423 | 0 | 423 | 0 | 423 | G2 |
| TCGA-49-4486 | 72 | MALE | WHITE | T1 | N0 | M0 | I | NA | Metastasis | Yes | NA | No | NA | 1 | 2318 | 1 | 2318 | 1 | 2045 | 1 | 2045 | G2 |
| TCGA-49-4487 | 72 | FEMALE | WHITE | T1 | N0 | M0 | I | NA | NA | Yes | NA | No | NA | 1 | 855 | 1 | 855 | 1 | 697 | 1 | 697 | G2 |
| TCGA-49-4488 | 74 | FEMALE | WHITE | T1 | N0 | NA | I | NA | NA | Yes | NA | No | NA | 1 | 869 | NA | 869 | NA | NA | 1 | 634 | G2 |
| TCGA-49-4494 | 77 | MALE | WHITE | T3 | N2 | M0 | III | NA | NA | Yes | NA | No | NA | 1 | 1081 | 1 | 1081 | NA | NA | 1 | 1081 | G2 |
| TCGA-49-4506 | 68 | FEMALE | WHITE | T2 | N1 | M0 | II | NA | Recurrence | Yes | NA | No | NA | 1 | 999 | NA | 999 | 1 | 692 | 1 | 692 | G2 |
| TCGA-49-4507 | 73 | FEMALE | WHITE | T3 | N1 | M0 | III | NA | NA | Yes | NA | No | Chemotherapy | 1 | 268 | 1 | 268 | NA | NA | 1 | 158 | G2 |
| TCGA-49-4514 | 79 | FEMALE | WHITE | T1 | N0 | M0 | I | NA | NA | Yes | NA | No | NA | 0 | 1700 | 0 | 1700 | 0 | 1700 | 0 | 1700 | G2 |
| TCGA-49-6742 | 70 | MALE | WHITE | T2 | N1 | M0 | II | NA | NA | Yes | NA | No | NA | 1 | 488 | 1 | 488 | NA | NA | 1 | 214 | G2 |
| TCGA-49-6743 | 81 | FEMALE | WHITE | T1 | N2 | NA | III | NA | NA | Yes | NA | No | Chemotherapy | 0 | 1621 | 0 | 1621 | 0 | 1621 | 0 | 1621 | G2 |
| TCGA-49-6745 | 82 | MALE | WHITE | T2 | N2 | M0 | III | NA | NA | Yes | NA | No | Chemotherapy | 0 | 522 | 0 | 522 | NA | NA | 0 | 522 | G2 |
| TCGA-49-6761 | 68 | FEMALE | WHITE | T1 | N2 | NA | III | NA | NA | Yes | NA | No | NA | 0 | 354 | 0 | 354 | 0 | 354 | 0 | 354 | G2 |
| TCGA-49-6767 | 46 | FEMALE | WHITE | T3 | N0 | NA | II | NA | NA | Yes | NA | No | NA | 0 | 677 | 0 | 677 | NA | NA | 0 | 677 | G2 |
| TCGA-49-AAQV | 63 | FEMALE | BLACK | T1 | N1 | NA | II | NA | Metastasis | No | NO | No | NA | 1 | 677 | 0 | 677 | 1 | 509 | 1 | 509 | G2 |
| TCGA-49-AAR2 | 64 | MALE | BLACK | T2 | N0 | NA | I | NA | NA | Yes | NO | No | Chemotherapy | 0 | 2224 | 0 | 2224 | 0 | 2224 | 0 | 2224 | G2 |
| TCGA-49-AAR3 | 69 | MALE | BLACK | T2 | N1 | NA | II | NA | Recurrence | NA | NO | No | NA | 0 | 1893 | 0 | 1893 | 1 | 1893 | 1 | 1893 | G2 |
| TCGA-49-AAR4 | 51 | MALE | BLACK | T2 | N2 | NA | III | NA | NA | Yes | YES | No | Chemotherapy | 1 | 879 | 1 | 879 | NA | NA | 1 | 879 | G2 |
| TCGA-49-AAR9 | 61 | MALE | BLACK | T3 | N0 | NA | II | NA | NA | Yes | NO | No | NA | 1 | 260 | 1 | 260 | NA | NA | 1 | 260 | G2 |
| TCGA-49-AARE | 51 | FEMALE | BLACK | T1 | N0 | NA | I | NA | Recurrence | Yes | NO | No | NA | 1 | 1229 | 1 | 1229 | 1 | 388 | 1 | 388 | G2 |
| TCGA-49-AARQ | 41 | FEMALE | BLACK | T2 | N0 | NA | I | NA | NA | Yes | NO | No | NA | 0 | 6732 | 0 | 6732 | 0 | 6732 | 0 | 6732 | G2 |
| TCGA-4B-A93V | 52 | FEMALE | BLACK | T1 | N0 | M0 | I | NA | Metastasis | Yes | YES | No | NA | 1 | 300 | 1 | 300 | NA | NA | 1 | 251 | G2 |
| TCGA-50-5044 | 72 | FEMALE | WHITE | T4 | N1 | M0 | III | NA | Metastasis | NA | NA | No | NA | 1 | 624 | 1 | 624 | NA | NA | 1 | 483 | G2 |
| TCGA-50-5049 | 70 | MALE | WHITE | T2 | N0 | M0 | I | NA | Recurrence | NA | NA | No | NA | 0 | 3094 | 0 | 3094 | NA | NA | 1 | 1568 | G2 |
| TCGA-50-5051 | 42 | FEMALE | WHITE | T2 | N2 | M0 | III | NA | Metastasis | Yes | NA | No | Chemotherapy | 1 | 478 | 1 | 478 | NA | NA | 1 | 184 | G2 |
| TCGA-50-5066 | 72 | MALE | BLACK | T2 | N0 | M0 | I | NA | Metastasis | No | NA | No | Chemotherapy | 0 | 1442 | 0 | 1442 | 1 | 477 | 1 | 477 | G2 |
| TCGA-50-5068 | 59 | FEMALE | WHITE | T2 | N1 | NA | II | NA | Metastasis | NA | NA | No | Chemotherapy | 1 | 1499 | 1 | 1499 | NA | NA | 1 | 686 | G2 |
| TCGA-50-5072 | 74 | MALE | WHITE | T2 | N2 | M0 | III | NA | Metastasis | Yes | NA | Yes | Chemotherapy | 1 | 250 | 1 | 250 | NA | NA | 1 | 213 | G2 |
| TCGA-50-5930 | 47 | MALE | WHITE | T2 | N2 | M0 | III | NA | Metastasis | NA | NA | No | Chemotherapy | 1 | 282 | 1 | 282 | NA | NA | 1 | 177 | G2 |
| TCGA-50-5931 | 75 | FEMALE | WHITE | T2 | N0 | M0 | I | NA | Metastasis | Yes | NA | No | NA | 1 | 434 | 1 | 434 | NA | NA | 1 | 425 | G2 |
| TCGA-50-5933 | 72 | MALE | WHITE | T4 | N2 | M0 | III | NA | NA | NA | NA | No | Chemotherapy | 1 | 2393 | 0 | 2393 | 0 | 2393 | 0 | 2393 | G2 |
| TCGA-50-5936 | 58 | MALE | WHITE | T2 | N2 | M0 | III | NA | NA | NA | NA | No | Chemotherapy | 1 | 257 | 1 | 257 | NA | NA | 1 | 150 | G2 |
| TCGA-50-5939 | 85 | MALE | WHITE | T2 | N0 | M0 | I | NA | NA | Yes | NA | No | NA | 1 | 460 | 0 | 460 | 0 | 460 | 0 | 460 | G2 |
| TCGA-50-5941 | 55 | FEMALE | WHITE | T2 | N2 | M0 | III | NA | NA | Yes | NA | No | Chemotherapy | 0 | 1474 | 0 | 1474 | 0 | 1474 | 0 | 1474 | G2 |
| TCGA-50-6590 | 72 | FEMALE | WHITE | T2 | N0 | M0 | I | NA | NA | Yes | NA | No | Chemotherapy | 1 | 1288 | 0 | 1288 | 0 | 1288 | 0 | 1288 | G2 |
| TCGA-50-6591 | 63 | FEMALE | WHITE | T2 | N0 | M1 | IV | NA | NA | No | NA | No | NA | 1 | 119 | 1 | 119 | NA | NA | 1 | 119 | G2 |
| TCGA-50-6592 | 71 | FEMALE | WHITE | T2 | N0 | M0 | I | NA | NA | Yes | NA | No | NA | 1 | 777 | 1 | 777 | NA | NA | 1 | 777 | G2 |
| TCGA-50-6594 | 79 | FEMALE | BLACK | T3 | N2 | M0 | III | NA | Metastasis | Yes | NA | No | NA | 1 | 370 | 1 | 370 | NA | NA | 1 | 285 | G2 |
| TCGA-50-6595 | 74 | FEMALE | WHITE | T2 | N2 | M0 | III | NA | Metastasis | Yes | NA | No | Chemotherapy | 1 | 189 | 1 | 189 | NA | NA | 1 | 182 | G2 |
| TCGA-50-6597 | 79 | FEMALE | WHITE | T2 | N0 | M0 | I | NA | NA | No | NA | No | NA | 1 | 1268 | 0 | 1268 | NA | NA | 0 | 1268 | G2 |
| TCGA-50-7109 | 60 | MALE | WHITE | T1 | N0 | M0 | I | NA | Metastasis | Yes | NA | No | NA | 1 | 308 | 1 | 308 | NA | NA | 1 | 15 | G2 |
| TCGA-51-4079 | 73 | FEMALE | BLACK | T2 | N0 | NA | I | NA | NA | Yes | NA | No | NA | 1 | 12 | NA | 12 | NA | NA | 0 | 12 | G2 |
| TCGA-51-4080 | 65 | MALE | BLACK | T4 | N1 | NA | III | NA | NA | Yes | NA | No | NA | 1 | 12 | 0 | 12 | NA | NA | 0 | 12 | G2 |
| TCGA-51-4081 | 55 | MALE | WHITE | T2 | N1 | M0 | II | NA | NA | Yes | NA | No | Chemotherapy | 0 | 911 | 0 | 911 | NA | NA | 0 | 911 | G2 |
| TCGA-51-6867 | 72 | FEMALE | WHITE | T1 | N0 | M0 | I | NA | NA | Yes | NA | No | NA | 1 | 1856 | 1 | 1856 | 1 | 788 | 1 | 788 | G2 |
| TCGA-52-7622 | 62 | FEMALE | WHITE | T1 | N0 | M0 | I | NA | NA | Yes | NA | No | NA | 0 | 862 | 0 | 862 | 0 | 862 | 0 | 862 | G2 |
| TCGA-52-7809 | 74 | MALE | WHITE | T2 | N0 | M0 | I | NA | NA | Yes | NA | No | NA | 1 | 166 | 1 | 166 | NA | NA | 1 | 166 | G2 |
| TCGA-52-7810 | 60 | FEMALE | WHITE | T3 | N0 | M0 | II | NA | NA | Yes | NA | No | NA | 0 | 923 | 0 | 923 | 0 | 923 | 0 | 923 | G2 |
| TCGA-52-7811 | 67 | MALE | WHITE | T2 | N0 | M0 | I | NA | Metastasis | Yes | NA | No | NA | 1 | 266 | 1 | 266 | NA | NA | 1 | 153 | G2 |
| TCGA-52-7812 | 68 | MALE | WHITE | T2 | N2 | M0 |  | NA | Metastasis | Yes | NA | No | Chemotherapy | 1 | 835 | 1 | 835 | NA | NA | 1 | 791 | G2 |
| TCGA-53-7624 | 40 | FEMALE | WHITE | T2 | N0 | M1 | IV | NA | Metastasis | Yes | NA | No | Chemotherapy | 1 | 1043 | 1 | 1043 | NA | NA | 1 | 400 | G2 |
| TCGA-53-7813 | 51 | FEMALE | WHITE | T4 | N0 | M0 | III | NA | NA | Yes | NA | No | Chemotherapy | 0 | 424 | 0 | 424 | NA | NA | 0 | 424 | G2 |
| TCGA-53-A4EZ | 63 | MALE | WHITE | T2 | N1 | NA | II | NA | NA | Yes | NO | No | Chemotherapy | 0 | 1071 | 0 | 1071 | NA | NA | 0 | 1071 | G2 |
| TCGA-55-1594 | 68 | MALE | WHITE | T2 | N2 | M0 | III | NA | NA | Yes | NA | No | NA | 0 | 1178 | 0 | 1178 | NA | NA | 0 | 1178 | G2 |
| TCGA-55-1596 | 55 | MALE | ASIAN | T2 | N1 | M0 | II | NA | NA | Yes | NA | No | Chemotherapy | 0 | 2065 | 0 | 2065 | 0 | 2065 | 0 | 2065 | G2 |
| TCGA-55-5899 | 58 | MALE | WHITE | T1 | N1 | M0 |  | NA | NA | Yes | NA | No | Chemotherapy | 0 | 930 | 0 | 930 | 0 | 930 | 0 | 930 | G2 |
| TCGA-55-6642 | 63 | MALE | WHITE | T2 | N0 | NA | I | NA | NA | Yes | NA | No | NA | 0 | 2449 | 0 | 2449 | 0 | 2449 | 0 | 2449 | G2 |
| TCGA-55-6712 | 71 | MALE | WHITE | T2 | N1 | NA | II | NA | NA | Yes | NA | No | Chemotherapy | 1 | 171 | 1 | 171 | NA | NA | 1 | 171 | G2 |
| TCGA-55-6968 | 61 | MALE | WHITE | T1 | N0 | M1 | IV | NA | NA | Yes | NA | No | Chemotherapy | 1 | 1293 | 1 | 1293 | NA | NA | 1 | 1293 | G2 |
| TCGA-55-6969 | 52 | MALE | WHITE | T2 | N0 | M0 | I | NA | NA | Yes | NA | No | NA | 0 | 1239 | 0 | 1239 | NA | NA | 0 | 1239 | G2 |
| TCGA-55-6972 | 72 | MALE | WHITE | T2 | N0 | M0 | I | NA | NA | Yes | NA | No | NA | 1 | 1632 | 0 | 1632 | 0 | 1632 | 0 | 1632 | G2 |
| TCGA-55-6975 | 61 | MALE | WHITE | T2 | N1 | M0 | II | NA | NA | Yes | NA | No | NA | 1 | 118 | 1 | 118 | NA | NA | 1 | 118 | G2 |
| TCGA-55-6978 | 81 | MALE | WHITE | T2 | N0 | NA | II | NA | NA | No | NA | No | NA | 1 | 176 | 1 | 176 | NA | NA | 1 | 42 | G2 |
| TCGA-55-6979 | 59 | FEMALE | WHITE | T2 | N1 | M0 | II | NA | Metastasis | Yes | NA | No | Chemotherapy | 1 | 237 | 1 | 237 | NA | NA | 1 | 195 | G2 |
| TCGA-55-6982 | 79 | FEMALE | WHITE | T2 | N1 | M0 | II | NA | Metastasis | No | NA | No | Chemotherapy | 1 | 995 | 1 | 995 | NA | NA | 1 | 183 | G2 |
| TCGA-55-6983 | 81 | MALE | WHITE | T2 | N1 | M0 | II | NA | NA | Yes | NA | No | Chemotherapy | 0 | 2823 | 0 | 2823 | 0 | 2823 | 0 | 2823 | G2 |
| TCGA-55-6984 | 71 | FEMALE | WHITE | T2 | N1 | M0 | II | NA | NA | NA | NA | No | Chemotherapy | 1 | 760 | 1 | 760 | 1 | 724 | 1 | 724 | G2 |
| TCGA-55-6987 | 77 | MALE | WHITE | T1 | N0 | M0 | I | NA | NA | Yes | NA | No | NA | 0 | 2137 | 0 | 2137 | 0 | 2137 | 0 | 2137 | G2 |
| TCGA-55-7281 | 70 | FEMALE | WHITE | T1 | N0 | M0 | I | NA | Metastasis | Yes | NA | No | Chemotherapy | 0 | 872 | 0 | 872 | 1 | 339 | 1 | 339 | G2 |
| TCGA-55-7570 | 60 | MALE | BLACK | T1 | N0 | NA | I | NA | NA | Yes | NA | No | NA | 0 | 824 | 0 | 824 | 0 | 824 | 0 | 824 | G2 |
| TCGA-55-7576 | 54 | MALE | BLACK | T2 | N0 | M0 | I | NA | NA | Yes | NA | No | Chemotherapy | 0 | 670 | 0 | 670 | 0 | 670 | 0 | 670 | G2 |
| TCGA-55-7724 | 76 | FEMALE | WHITE | T2 | N0 | NA | I | NA | NA | Yes | NA | No | NA | 0 | 705 | 0 | 705 | 0 | 705 | 0 | 705 | G2 |
| TCGA-55-7726 | 72 | FEMALE | WHITE | T1 | N0 | NA | I | NA | NA | Yes | NA | No | NA | 0 | 652 | 0 | 652 | 0 | 652 | 0 | 652 | G2 |
| TCGA-55-7815 | 76 | MALE | NA | T2 | N0 | NA | I | NA | Recurrence | No | NA | No | Chemotherapy | 0 | 773 | 0 | 773 | 1 | 466 | 1 | 466 | G2 |
| TCGA-55-7907 | 77 | MALE | WHITE | T2 | N1 | NA | II | NA | Metastasis | Yes | NA | No | NA | 1 | 343 | 1 | 343 | 1 | 294 | 1 | 294 | G2 |
| TCGA-55-7910 | 50 | FEMALE | BLACK | T2 | N0 | M0 | II | NA | NA | Yes | NA | No | Chemotherapy | 0 | 1040 | 0 | 1040 | 1 | 1018 | 1 | 1018 | G2 |
| TCGA-55-7913 | 61 | FEMALE | BLACK | T1 | N0 | NA | I | NA | Metastasis | Yes | NA | No | NA | 1 | 561 | 1 | 561 | 1 | 480 | 1 | 480 | G2 |
| TCGA-55-7914 | 71 | FEMALE | WHITE | T1 | N1 | NA | II | NA | NA | Yes | NA | No | Chemotherapy | 1 | 187 | 1 | 187 | NA | NA | 1 | 187 | G2 |
| TCGA-55-7995 | 73 | FEMALE | WHITE | T1 | N0 | M0 | I | NA | Recurrence | Yes | NA | No | Chemotherapy | 0 | 889 | 0 | 889 | 1 | 468 | 1 | 468 | G2 |
| TCGA-55-8085 | 64 | MALE | WHITE | T1 | N0 | M0 | I | NA | NA | Yes | NA | No | NA | 0 | 904 | 0 | 904 | 0 | 904 | 0 | 904 | G2 |
| TCGA-55-8089 | 56 | MALE | WHITE | T1 | N0 | M0 | I | NA | NA | Yes | NA | No | NA | 1 | 702 | 0 | 702 | 0 | 702 | 0 | 702 | G2 |
| TCGA-55-8092 | 75 | MALE | WHITE | T3 | N0 | NA | II | NA | Recurrence | Yes | NA | No | NA | 1 | 154 | 1 | 154 | 1 | 127 | 1 | 127 | G2 |
| TCGA-55-8094 | 51 | MALE | WHITE | T2 | N0 | M1 | IV | NA | NA | Yes | NA | No | NA | 0 | 541 | 0 | 541 | NA | NA | 0 | 541 | G2 |
| TCGA-55-8203 | 69 | FEMALE | WHITE | T1 | N0 | M0 | I | NA | NA | Yes | NA | No | NA | 0 | 547 | 0 | 547 | 0 | 547 | 0 | 547 | G2 |
| TCGA-55-8204 | 87 | FEMALE | WHITE | T2 | N0 | NA | I | NA | NA | Yes | NA | No | NA | 0 | 515 | 0 | 515 | 0 | 515 | 0 | 515 | G2 |
| TCGA-55-8205 | 76 | FEMALE | WHITE | T2 | N0 | M0 | II | NA | Metastasis | Yes | NA | No | NA | 0 | 599 | 0 | 599 | NA | NA | 1 | 495 | G2 |
| TCGA-55-8208 | 73 | FEMALE | WHITE | T1 | N0 | M0 | I | NA | Metastasis | Yes | NA | No | NA | 0 | 674 | 0 | 674 | 1 | 511 | 1 | 511 | G2 |
| TCGA-55-8301 | 58 | MALE | WHITE | T2 | N0 | NA | I | NA | Recurrence | Yes | NO | No | Chemotherapy | 0 | 534 | 0 | 534 | 1 | 238 | 1 | 238 | G2 |
| TCGA-55-8505 | 62 | MALE | WHITE | T1 | N2 | NA | III | NA | NA | No | NA | No | Chemotherapy | 0 | 440 | 0 | 440 | 0 | 440 | 0 | 440 | G2 |
| TCGA-55-8506 | 62 | FEMALE | WHITE | T3 | N0 | NA | II | NA | NA | Yes | NA | No | NA | 0 | 11 | 0 | 11 | NA | NA | 0 | 11 | G2 |
| TCGA-55-8508 | 60 | FEMALE | BLACK | T2 | N1 | NA | II | NA | NA | Yes | NO | No | Chemotherapy | 0 | 617 | 0 | 617 | 0 | 617 | 0 | 617 | G2 |
| TCGA-55-8511 | 73 | FEMALE | WHITE | T2 | N0 | NA | I | NA | NA | Yes | NO | No | NA | 0 | 552 | 0 | 552 | 1 | 460 | 1 | 460 | G2 |
| TCGA-55-8514 | 70 | FEMALE | BLACK | T2 | N0 | NA | I | NA | NA | Yes | NO | No | NA | 0 | 520 | 0 | 520 | 0 | 520 | 0 | 520 | G2 |
| TCGA-55-8614 | 76 | MALE | WHITE | T2 | N0 | NA | I | NA | NA | Yes | NO | No | NA | 0 | 536 | 0 | 536 | 0 | 536 | 0 | 536 | G2 |
| TCGA-55-8615 | 67 | MALE | WHITE | T3 | N2 | NA | III | NA | Metastasis | Yes | NO | No | Chemotherapy | 0 | 446 | 0 | 446 | 1 | 224 | 1 | 224 | G2 |
| TCGA-55-8620 | 60 | MALE | WHITE | T1 | N1 | M1 | IV | NA | NA | Yes | YES | No | NA | 1 | 375 | 1 | 375 | NA | NA | 1 | 375 | G2 |
| TCGA-55-A48Y | 69 | MALE | WHITE | T2 | N0 | M0 | II | NA | NA | Yes | NA | No | Chemotherapy | 0 | 630 | 0 | 630 | 0 | 630 | 0 | 630 | G2 |
| TCGA-55-A48Z | 60 | FEMALE | WHITE | T1 | N3 | NA | III | NA | Metastasis | Yes | NA | No | Chemotherapy | 0 | 651 | 0 | 651 | NA | NA | 1 | 536 | G2 |
| TCGA-55-A490 | 78 | MALE | WHITE | T2 | N0 | NA | II | NA | NA | Yes | NA | No | Chemotherapy | 1 | 99 | 0 | 99 | NA | NA | 0 | 99 | G2 |
| TCGA-55-A491 | 81 | FEMALE | WHITE | T1 | N0 | NA | I | NA | NA | Yes | NA | No | NA | 0 | 626 | 0 | 626 | 0 | 626 | 0 | 626 | G2 |
| TCGA-55-A493 | 54 | FEMALE | WHITE | T2 | N0 | M0 | I | NA | NA | Yes | NA | No | NA | 0 | 28 | 0 | 28 | NA | NA | 0 | 28 | G2 |
| TCGA-55-A4DF | 88 | MALE | WHITE | T1 | N0 | NA | I | NA | Metastasis | Yes | NO | No | NA | 1 | 614 | 1 | 614 | 1 | 515 | 1 | 515 | G2 |
| TCGA-56-1622 | 58 | MALE | WHITE | T2 | N0 | M0 | I | NA | NA | Yes | NA | No | NA | 1 | 881 | 1 | 881 | NA | NA | 1 | 881 | G2 |
| TCGA-56-5897 | 74 | MALE | WHITE | T1 | N0 | NA | I | NA | NA | Yes | NA | No | NA | 0 | 378 | 0 | 378 | 0 | 378 | 0 | 378 | G2 |
| TCGA-56-5898 | 69 | MALE | WHITE | T1 | N0 | M0 | I | NA | NA | Yes | NA | No | NA | 0 | 555 | 0 | 555 | 0 | 555 | 0 | 555 | G2 |
| TCGA-56-6545 | 77 | FEMALE | WHITE | T2 | N0 | M0 | I | NA | NA | Yes | NA | No | NA | 0 | 666 | 0 | 666 | 0 | 666 | 0 | 666 | G2 |
| TCGA-56-6546 | 67 | MALE | WHITE | T2 | N0 | NA | II | NA | NA | Yes | NA | No | NA | 0 | NA | 0 | NA | NA | NA | 0 | NA | G2 |
| TCGA-56-7221 | 79 | MALE | WHITE | T2 | N0 | M0 | I | NA | NA | Yes | NA | No | NA | 0 | 608 | 0 | 608 | 0 | 608 | 0 | 608 | G2 |
| TCGA-56-7222 | 60 | MALE | WHITE | T2 | N0 | M0 | I | NA | NA | Yes | NA | No | Chemotherapy | 1 | 562 | 1 | 562 | 1 | 427 | 1 | 427 | G2 |
| TCGA-56-7223 | 66 | MALE | WHITE | T3 | N1 | NA | III | NA | Recurrence | Yes | NA | No | NA | 1 | 442 | 1 | 442 | NA | NA | 1 | 132 | G2 |
| TCGA-56-7579 | 61 | MALE | WHITE | T3 | N1 | M0 | III | NA | Recurrence | Yes | NA | No | NA | 1 | 951 | 1 | 951 | NA | NA | 1 | 149 | G2 |
| TCGA-56-7580 | 84 | MALE | WHITE | T2 | N0 | M0 | I | NA | NA | Yes | NA | No | NA | 0 | 925 | 0 | 925 | 0 | 925 | 0 | 925 | G2 |
| TCGA-56-7582 | 83 | MALE | WHITE | T2 | N0 | M0 | I | NA | NA | Yes | NA | No | NA | 0 | 601 | 0 | 601 | 0 | 601 | 0 | 601 | G2 |
| TCGA-56-7730 | 73 | MALE | WHITE | T2 | N0 | M0 | II | NA | NA | Yes | NA | No | Chemotherapy | 1 | 198 | 1 | 198 | NA | NA | 1 | 198 | G2 |
| TCGA-56-7731 | 66 | FEMALE | WHITE | T2 | N0 | NA | I | NA | NA | Yes | NA | No | NA | 1 | 3 | 0 | 3 | NA | NA | 0 | 3 | G2 |
| TCGA-56-7822 | 75 | MALE | BLACK | T2 | N1 | M0 | II | NA | NA | Yes | NA | No | Chemotherapy | 1 | 532 | 1 | 532 | NA | NA | 1 | 301 | G2 |
| TCGA-56-7823 | 58 | FEMALE | WHITE | T1 | N1 | M0 | II | NA | NA | Yes | NA | No | Chemotherapy | 0 | 1011 | 0 | 1011 | 0 | 1011 | 0 | 1011 | G2 |
| TCGA-56-8082 | 80 | FEMALE | WHITE | T2 | N0 | NA | II | NA | NA | Yes | NA | No | NA | 0 | 455 | 0 | 455 | 0 | 455 | 0 | 455 | G2 |
| TCGA-56-8083 | 56 | MALE | WHITE | T2 | N0 | NA | I | NA | NA | Yes | NA | No | NA | 0 | 150 | 0 | 150 | NA | NA | 0 | 150 | G2 |
| TCGA-56-8201 | 74 | MALE | WHITE | T3 | N0 | NA | II | NA | NA | Yes | NA | No | NA | 1 | 397 | 1 | 397 | NA | NA | 1 | 397 | G2 |
| TCGA-56-8304 | 73 | FEMALE | WHITE | T1 | N0 | NA | I | NA | NA | Yes | NA | No | NA | 0 | 106 | 0 | 106 | 0 | 106 | 0 | 106 | G2 |
| TCGA-56-8305 | 72 | MALE | WHITE | T2 | N0 | M0 | I | NA | NA | Yes | NA | No | NA | 0 | 105 | 0 | 105 | 0 | 105 | 0 | 105 | G2 |
| TCGA-56-8307 | 55 | FEMALE | WHITE | T3 | N0 | M0 | II | NA | NA | Yes | NA | No | NA | 0 | 818 | 0 | 818 | 0 | 818 | 0 | 818 | G2 |
| TCGA-56-8308 | 79 | MALE | WHITE | T3 | N0 | NA | II | NA | NA | Yes | YES | No | Chemotherapy | 0 | 517 | 0 | 517 | NA | NA | 0 | 517 | G2 |
| TCGA-56-8309 | 66 | MALE | WHITE | T1 | N0 | NA | I | NA | NA | Yes | NA | No | NA | 0 | 428 | 0 | 428 | 0 | 428 | 0 | 428 | G2 |
| TCGA-56-8503 | 76 | FEMALE | WHITE | T3 | N0 | M0 | II | NA | NA | Yes | NA | No | NA | 0 | 41 | 0 | 41 | NA | NA | 0 | 41 | G2 |
| TCGA-56-8504 | 74 | MALE | WHITE | T2 | N0 | NA | I | NA | NA | Yes | NO | No | NA | 0 | 510 | 0 | 510 | 0 | 510 | 0 | 510 | G2 |
| TCGA-56-8622 | 68 | MALE | WHITE | T2 | N0 | M0 | I | NA | NA | Yes | NA | No | NA | 0 | 55 | 0 | 55 | NA | NA | 0 | 55 | G2 |
| TCGA-56-8624 | 84 | MALE | WHITE | T3 | N0 | NA | II | NA | NA | Yes | NA | No | Chemotherapy | 0 | 420 | 0 | 420 | 0 | 420 | 0 | 420 | G2 |
| TCGA-56-8625 | 66 | FEMALE | WHITE | T3 | N1 | NA | III | NA | Metastasis | Yes | NO | No | Chemotherapy | 1 | 315 | 1 | 315 | NA | NA | 1 | 272 | G2 |
| TCGA-56-8629 | 63 | MALE | WHITE | T2 | N0 | NA | II | NA | NA | Yes | NO | No | Chemotherapy | 0 | 481 | 0 | 481 | 0 | 481 | 0 | 481 | G2 |
| TCGA-56-A49D | 67 | MALE | WHITE | T2 | N2 | NA | III | NA | NA | Yes | NA | No | NA | 0 | 637 | 0 | 637 | 0 | 637 | 0 | 637 | G2 |
| TCGA-56-A4BW | 55 | MALE | WHITE | T2 | N1 | M0 | II | NA | NA | Yes | NA | No | Chemotherapy | 0 | 585 | 0 | 585 | 0 | 585 | 0 | 585 | G2 |
| TCGA-56-A4BX | 70 | MALE | WHITE | T2 | N0 | NA | II | NA | NA | Yes | NO | No | Chemotherapy | 0 | 405 | 0 | 405 | 0 | 405 | 0 | 405 | G2 |
| TCGA-56-A4BY | 66 | MALE | WHITE | T2 | N0 | NA | I | NA | NA | Yes | NO | No | NA | 1 | 543 | 1 | 543 | NA | NA | 1 | 543 | G2 |
| TCGA-56-A4ZK | 76 | FEMALE | WHITE | T2 | N0 | M0 | I | NA | NA | Yes | NA | No | NA | 0 | 570 | 0 | 570 | 0 | 570 | 0 | 570 | G2 |
| TCGA-56-A5DR | 81 | MALE | WHITE | T1 | N0 | NA | I | NA | NA | No | NO | No | NA | 0 | 4 | 0 | 4 | 0 | 4 | 0 | 4 | G2 |
| TCGA-56-A5DS | 72 | FEMALE | WHITE | T2 | N0 | NA | I | NA | NA | Yes | NA | No | NA | 0 | 8 | 0 | 8 | 0 | 8 | 0 | 8 | G2 |
| TCGA-56-A62T | 78 | MALE | BLACK | T2 | N0 | NA | II | NA | NA | Yes | NA | No | Chemotherapy | 0 | 440 | 0 | 440 | 0 | 440 | 0 | 440 | G2 |
| TCGA-58-8386 | 75 | MALE | NA | T3 | NA | M1 | IV | NA | NA | Yes | NO | No | NA | 1 | 1 | 0 | 1 | NA | NA | 0 | 1 | G2 |
| TCGA-58-8387 | 60 | MALE | WHITE | T2 | N0 | M0 | II | NA | NA | Yes | NO | No | Chemotherapy | 1 | 403 | 0 | 403 | 0 | 403 | 0 | 403 | G2 |
| TCGA-58-8388 | 60 | MALE | WHITE | T2 | N0 | M0 | I | NA | NA | Yes | NO | No | NA | 1 | 412 | 0 | 412 | 0 | 412 | 0 | 412 | G2 |
| TCGA-58-8390 | 70 | MALE | WHITE | T2 | N0 | M0 | II | NA | NA | Yes | NO | No | NA | 0 | 911 | 0 | 911 | 0 | 911 | 0 | 911 | G2 |
| TCGA-58-8391 | 57 | FEMALE | WHITE | T2 | N2 | M0 | III | NA | NA | Yes | YES | No | Chemotherapy | 0 | 2167 | 0 | 2167 | 1 | 2033 | 1 | 2033 | G2 |
| TCGA-58-8392 | 70 | MALE | WHITE | T2 | N0 | M0 | I | NA | Metastasis | Yes | NO | No | NA | 1 | 501 | 1 | 501 | 1 | 96 | 1 | 96 | G2 |
| TCGA-58-8393 | 68 | FEMALE | WHITE | T2 | N0 | M0 | I | NA | NA | Yes | NO | No | NA | 0 | 1058 | 0 | 1058 | 0 | 1058 | 0 | 1058 | G2 |
| TCGA-58-A46J | 64 | MALE | WHITE | T2 | N1 | M0 | II | NA | NA | Yes | NO | No | Chemotherapy | 0 | 2589 | 0 | 2589 | 0 | 2589 | 0 | 2589 | G2 |
| TCGA-58-A46K | 59 | MALE | WHITE | T2 | N2 | M0 | III | NA | NA | Yes | YES | No | Chemotherapy | 1 | 1045 | 1 | 1045 | 1 | 735 | 1 | 735 | G2 |
| TCGA-58-A46L | 73 | MALE | WHITE | T2 | N2 | M0 | III | NA | NA | Yes | NO | No | Chemotherapy | 0 | 1723 | 0 | 1723 | 0 | 1723 | 0 | 1723 | G2 |
| TCGA-58-A46M | 61 | MALE | WHITE | T2 | N1 | M0 | II | NA | NA | Yes | NO | No | NA | 0 | 1072 | 0 | 1072 | 0 | 1072 | 0 | 1072 | G2 |
| TCGA-58-A46N | 52 | MALE | WHITE | T2 | N0 | M0 | I | NA | Recurrence | Yes | NO | No | NA | 0 | 910 | 0 | 910 | 1 | 630 | 1 | 630 | G2 |
| TCGA-60-2695 | 74 | FEMALE | WHITE | T2 | N0 | M0 | I | NA | NA | Yes | NA | No | NA | 0 | 642 | 0 | 642 | 0 | 642 | 0 | 642 | G2 |
| TCGA-60-2696 | 76 | FEMALE | BLACK | T2 | N0 | M0 | II | NA | NA | Yes | NA | Yes | NA | 1 | 109 | NA | 109 | NA | NA | 0 | 109 | G2 |
| TCGA-60-2698 | 62 | MALE | WHITE | T2 | N1 | M0 | II | NA | NA | Yes | NA | No | Chemotherapy | 1 | 311 | 1 | 311 | NA | NA | 1 | 114 | G2 |
| TCGA-60-2703 | 73 | MALE | WHITE | T2 | N1 | M0 | II | NA | NA | Yes | NA | No | NA | 1 | 2945 | NA | 2945 | 1 | 1757 | 1 | 1757 | G2 |
| TCGA-60-2704 | 73 | MALE | WHITE | T2 | N1 | M0 | II | NA | NA | Yes | NA | No | Chemotherapy | 1 | 1154 | 1 | 1154 | NA | NA | 1 | 532 | G2 |
| TCGA-60-2706 | 58 | MALE | WHITE | T1 | N0 | M0 | I | NA | NA | Yes | NA | No | NA | 0 | 2820 | 0 | 2820 | 0 | 2820 | 0 | 2820 | G2 |
| TCGA-60-2707 | 70 | MALE | NA | T2 | N0 | M0 | I | NA | NA | Yes | NA | No | Chemotherapy | 1 | 667 | 1 | 667 | 1 | 579 | 1 | 579 | G2 |
| TCGA-60-2708 | 64 | FEMALE | WHITE | T2 | N1 | M0 | II | NA | NA | Yes | NA | No | Chemotherapy | 0 | 2447 | 0 | 2447 | 0 | 2447 | 0 | 2447 | G2 |
| TCGA-60-2709 | 69 | MALE | BLACK | T2 | N0 | NA | I | NA | NA | Yes | NA | No | NA | 0 | 1505 | 0 | 1505 | 0 | 1505 | 0 | 1505 | G2 |
| TCGA-60-2710 | 67 | FEMALE | WHITE | T1 | N1 | M0 | II | NA | NA | Yes | NA | Yes | Chemotherapy | 0 | 2024 | 0 | 2024 | 0 | 2024 | 0 | 2024 | G2 |
| TCGA-60-2711 | 64 | FEMALE | WHITE | T2 | N0 | M0 | I | NA | NA | Yes | NA | No | NA | 0 | 1260 | 0 | 1260 | 0 | 1260 | 0 | 1260 | G2 |
| TCGA-60-2712 | 79 | FEMALE | WHITE | T2 | N1 | M0 | II | NA | NA | Yes | NA | No | NA | 1 | 274 | NA | 274 | 0 | 274 | 0 | 274 | G2 |
| TCGA-60-2713 | 64 | MALE | WHITE | T2 | N0 | M0 | I | NA | NA | Yes | NA | No | Chemotherapy | 0 | 1731 | 0 | 1731 | 1 | 1356 | 1 | 1356 | G2 |
| TCGA-60-2714 | 66 | FEMALE | WHITE | T2 | N1 | M0 | II | NA | NA | Yes | NA | Yes | Chemotherapy | 0 | 1531 | 0 | 1531 | 0 | 1531 | 0 | 1531 | G2 |
| TCGA-60-2716 | 39 | MALE | NA | T2 | N1 | M0 | II | NA | NA | No | NA | No | Chemotherapy | 0 | 1475 | 0 | 1475 | 0 | 1475 | 0 | 1475 | G2 |
| TCGA-60-2719 | 83 | FEMALE | WHITE | T1 | N0 | M0 | I | NA | NA | Yes | NA | No | NA | 0 | 1297 | 0 | 1297 | 0 | 1297 | 0 | 1297 | G2 |
| TCGA-60-2720 | 60 | FEMALE | WHITE | T2 | N0 | M0 | I | NA | NA | Yes | NA | No | Chemotherapy | 0 | 97 | 0 | 97 | NA | NA | 0 | 97 | G2 |
| TCGA-60-2721 | 73 | MALE | WHITE | T2 | N0 | M0 | I | NA | NA | Yes | NA | No | NA | 0 | 983 | 0 | 983 | 0 | 983 | 0 | 983 | G2 |
| TCGA-60-2722 | 66 | MALE | WHITE | T2 | N1 | M0 | II | NA | NA | Yes | NA | No | Chemotherapy | 0 | 908 | 0 | 908 | 1 | 851 | 1 | 851 | G2 |
| TCGA-60-2723 | 74 | FEMALE | WHITE | T2 | N0 | M0 | I | NA | NA | Yes | NA | No | NA | 0 | 1092 | 0 | 1092 | 0 | 1092 | 0 | 1092 | G2 |
| TCGA-60-2724 | 47 | MALE | WHITE | T3 | N1 | M0 | III | NA | NA | Yes | NA | No | Chemotherapy | 0 | 717 | 0 | 717 | NA | NA | 0 | 717 | G2 |
| TCGA-60-2725 | 74 | MALE | WHITE | T2 | N0 | M0 | I | NA | NA | Yes | NA | No | NA | 0 | 816 | 0 | 816 | 0 | 816 | 0 | 816 | G2 |
| TCGA-60-2726 | 56 | MALE | WHITE | T2 | N1 | M0 | II | NA | NA | Yes | NA | No | NA | 1 | 358 | 0 | 358 | 0 | 358 | 0 | 358 | G2 |
| TCGA-62-8394 | 65 | FEMALE | WHITE | T4 | N2 | M0 | III | NA | NA | No | NO | No | Chemotherapy | 1 | 139 | 0 | 139 | 0 | 139 | 0 | 139 | G2 |
| TCGA-62-8398 | 55 | MALE | WHITE | T2 | N2 | M0 | III | NA | NA | Yes | NO | No | Chemotherapy | 1 | 444 | 0 | 444 | 0 | 444 | 0 | 444 | G2 |
| TCGA-62-8402 | 73 | FEMALE | WHITE | T2 | N2 | M0 | III | NA | Metastasis | No | YES | No | NA | 1 | 1498 | 1 | 1498 | 1 | 772 | 1 | 772 | G2 |
| TCGA-62-A46O | 65 | FEMALE | WHITE | T2 | N0 | M0 | I | NA | NA | Yes | NO | No | NA | 1 | 1454 | 1 | 1454 | 1 | 943 | 1 | 943 | G2 |
| TCGA-62-A46U | 71 | FEMALE | WHITE | T2 | N1 | M0 | II | NA | NA | No | NO | No | Chemotherapy | 0 | 2067 | 0 | 2067 | 0 | 2067 | 0 | 2067 | G2 |
| TCGA-62-A471 | 64 | MALE | WHITE | T2 | N1 | M0 | II | NA | NA | Yes | NO | No | Chemotherapy | 0 | 1246 | 0 | 1246 | 0 | 1246 | 0 | 1246 | G2 |
| TCGA-63-5128 | NA | MALE | NA | T2 | N0 | M0 | I | NA | NA | Yes | NA | No | NA | 1 | NA | 1 | NA | NA | NA | 1 | NA | G2 |
| TCGA-63-5131 | NA | MALE | NA | T2 | N1 | M0 | II | NA | NA | Yes | NA | No | NA | 1 | NA | 0 | NA | 0 | NA | 0 | NA | G2 |
| TCGA-63-6202 | NA | MALE | NA | T2 | N0 | M0 | II | NA | NA | Yes | NA | No | NA | 0 | 1602 | 0 | 1602 | 0 | 1602 | 0 | 1602 | G2 |
| TCGA-63-7020 | NA | MALE | NA | T1 | N0 | M0 | I | NA | NA | Yes | NA | No | NA | 0 | 2133 | 0 | 2133 | 0 | 2133 | 0 | 2133 | G2 |
| TCGA-63-7021 | NA | MALE | NA | T1 | N0 | M0 | I | NA | Primary | Yes | NA | No | NA | 0 | 2142 | 0 | 2142 | 0 | 2142 | 1 | 1503 | G2 |
| TCGA-63-7022 | NA | FEMALE | NA | T1 | N0 | M0 | I | NA | NA | Yes | NA | No | NA | 0 | 2073 | 0 | 2073 | 0 | 2073 | 0 | 2073 | G2 |
| TCGA-63-7023 | NA | MALE | NA | T1 | N1 | M0 | II | NA | NA | Yes | NA | No | Chemotherapy | 0 | 1682 | 0 | 1682 | 0 | 1682 | 0 | 1682 | G2 |
| TCGA-63-A5M9 | NA | FEMALE | NA | T2 | N1 | M0 | II | NA | NA | Yes | NA | No | NA | 0 | 0 | 0 | 0 | NA | NA | 0 | 0 | G2 |
| TCGA-63-A5MB | 62 | MALE | NA | T2 | N0 | M0 | I | NA | NA | Yes | NO | No | Chemotherapy | 0 | 3123 | 0 | 3123 | 0 | 3123 | 0 | 3123 | G2 |
| TCGA-63-A5MG | 68 | MALE | NA | T2 | N0 | M0 | I | NA | NA | Yes | NO | No | NA | 0 | 2148 | 0 | 2148 | 0 | 2148 | 0 | 2148 | G2 |
| TCGA-63-A5MH | 68 | MALE | NA | T1 | N0 | M0 | I | NA | NA | Yes | NO | No | NA | 0 | 2026 | 0 | 2026 | 0 | 2026 | 0 | 2026 | G2 |
| TCGA-63-A5MI | 65 | MALE | NA | T2 | N2 | M0 | III | NA | NA | Yes | NO | No | NA | 0 | 1784 | 0 | 1784 | 0 | 1784 | 0 | 1784 | G2 |
| TCGA-63-A5MJ | 54 | MALE | NA | T2 | N1 | M0 | II | NA | NA | Yes | NO | No | Chemotherapy | 0 | 1824 | 0 | 1824 | 0 | 1824 | 0 | 1824 | G2 |
| TCGA-63-A5ML | 68 | MALE | NA | T2 | N0 | M0 | I | NA | NA | Yes | NO | No | Chemotherapy | 0 | 1386 | 0 | 1386 | 0 | 1386 | 0 | 1386 | G2 |
| TCGA-63-A5MM | 69 | FEMALE | NA | T2 | N1 | M0 | II | NA | NA | Yes | NO | No | Chemotherapy | 1 | 456 | 1 | 456 | 1 | 218 | 1 | 218 | G2 |
| TCGA-63-A5MN | 78 | FEMALE | NA | T3 | N0 | M0 | II | NA | NA | No | YES | No | NA | 1 | 345 | 1 | 345 | 1 | 320 | 1 | 320 | G2 |
| TCGA-63-A5MP | 56 | MALE | NA | T2 | N1 | M0 | II | NA | Recurrence | Yes | YES | No | Chemotherapy | 0 | 769 | 0 | 769 | 1 | 511 | 1 | 511 | G2 |
| TCGA-63-A5MR | 70 | FEMALE | NA | T2 | N0 | M0 | I | NA | NA | Yes | NO | No | NA | 0 | 2716 | 0 | 2716 | 0 | 2716 | 0 | 2716 | G2 |
| TCGA-63-A5MS | 78 | MALE | NA | T2 | N0 | M0 | I | NA | NA | Yes | NO | No | NA | 0 | 2381 | 0 | 2381 | 0 | 2381 | 0 | 2381 | G2 |
| TCGA-63-A5MT | 74 | MALE | NA | T3 | N0 | M0 | II | NA | Metastasis | Yes | NO | No | Chemotherapy | 0 | 498 | 0 | 498 | 1 | 427 | 1 | 427 | G2 |
| TCGA-63-A5MU | 48 | MALE | NA | T2 | N1 | M0 | II | NA | Metastasis | Yes | NO | No | Chemotherapy | 1 | NA | 1 | NA | 1 | 420 | 1 | 420 | G2 |
| TCGA-63-A5MV | 69 | MALE | NA | T2 | N0 | M0 | II | NA | NA | Yes | NO | No | Chemotherapy | 0 | 1100 | 0 | 1100 | 0 | 1100 | 0 | 1100 | G2 |
| TCGA-63-A5MW | 76 | MALE | NA | T2 | N0 | M0 | I | NA | NA | Yes | NO | No | NA | 0 | 1639 | 0 | 1639 | 0 | 1639 | 0 | 1639 | G2 |
| TCGA-63-A5MY | 63 | MALE | NA | T1 | N0 | M0 | I | NA | NA | Yes | NO | No | NA | 0 | 1052 | 0 | 1052 | 0 | 1052 | 0 | 1052 | G2 |
| TCGA-64-1676 | 58 | MALE | WHITE | T1 | N0 | M0 | I | NA | NA | Yes | NA | No | NA | 0 | 1728 | 0 | 1728 | 0 | 1728 | 0 | 1728 | G2 |
| TCGA-64-1677 | 77 | FEMALE | WHITE | T2 | N2 | M0 | III | NA | NA | Yes | NA | No | Chemotherapy | 1 | 628 | NA | 628 | NA | NA | 1 | 360 | G2 |
| TCGA-64-1678 | 70 | FEMALE | WHITE | T2 | N0 | M0 |  | NA | NA | Yes | NA | No | Chemotherapy | 0 | 1189 | 0 | 1189 | 0 | 1189 | 0 | 1189 | G2 |
| TCGA-64-1679 | 58 | FEMALE | WHITE | T1 | N2 | M0 | III | NA | NA | Yes | NA | No | Chemotherapy | 0 | 2488 | 0 | 2488 | 0 | 2488 | 0 | 2488 | G2 |
| TCGA-64-5774 | 60 | MALE | WHITE | T2 | N0 | M0 | I | NA | NA | Yes | NA | No | NA | 0 | 2676 | 0 | 2676 | 1 | 246 | 1 | 246 | G2 |
| TCGA-64-5775 | 71 | MALE | WHITE | T4 | N0 | M0 | III | NA | NA | Yes | NA | Yes | Chemotherapy | 1 | 62 | 1 | 62 | NA | NA | 1 | 62 | G2 |
| TCGA-64-5778 | 60 | MALE | WHITE | T2 | N0 | M0 | I | NA | Metastasis | Yes | NA | No | Chemotherapy | 0 | 1305 | 0 | 1305 | 1 | 962 | 1 | 962 | G2 |
| TCGA-64-5779 | 61 | MALE | WHITE | T2 | N2 | M0 | III | NA | Metastasis | Yes | NA | No | Chemotherapy | 0 | 864 | 0 | 864 | NA | NA | 1 | 795 | G2 |
| TCGA-64-5781 | 55 | FEMALE | WHITE | T2 | N0 | M0 | I | NA | NA | Yes | NA | No | Chemotherapy | 0 | 1559 | 0 | 1559 | NA | NA | 1 | 96 | G2 |
| TCGA-66-2727 | 55 | FEMALE | NA | T2 | N0 | M0 | I | NA | NA | Yes | NA | No | NA | 1 | 516 | NA | 516 | NA | NA | 0 | 516 | G2 |
| TCGA-66-2734 | 62 | FEMALE | NA | T2 | N0 | M0 | I | NA | NA | Yes | NA | No | NA | 0 | 1311 | 0 | 1311 | NA | NA | 0 | 1311 | G2 |
| TCGA-66-2737 | 72 | MALE | NA | T2 | N1 | M0 | II | NA | NA | Yes | NA | No | NA | 0 | 61 | 0 | 61 | NA | NA | 0 | 61 | G2 |
| TCGA-66-2742 | 70 | MALE | NA | T2 | N1 | M1 | IV | NA | NA | Yes | NA | No | NA | 0 | 641 | 0 | 641 | NA | NA | 0 | 641 | G2 |
| TCGA-66-2744 | 71 | MALE | NA | T2 | N1 | M0 | II | NA | NA | Yes | NA | No | NA | 0 | 30 | 0 | 30 | NA | NA | 0 | 30 | G2 |
| TCGA-66-2753 | 69 | MALE | NA | T2 | N0 | M0 | I | NA | NA | Yes | NA | No | NA | 0 | 31 | 0 | 31 | NA | NA | 0 | 31 | G2 |
| TCGA-66-2754 | 67 | MALE | NA | T2 | N2 | M0 | III | NA | NA | Yes | NA | No | NA | 0 | 61 | 0 | 61 | NA | NA | 0 | 61 | G2 |
| TCGA-66-2755 | 63 | MALE | NA | T2 | N0 | M0 | I | NA | NA | Yes | NA | No | NA | 0 | 28 | 0 | 28 | NA | NA | 0 | 28 | G2 |
| TCGA-66-2756 | 68 | MALE | NA | T4 | N0 | M0 | III | NA | NA | Yes | NA | No | NA | 0 | 30 | 0 | 30 | NA | NA | 0 | 30 | G2 |
| TCGA-66-2757 | 65 | FEMALE | NA | T1 | N0 | M0 | I | NA | NA | Yes | NA | No | Chemotherapy | 1 | 1338 | 1 | 1338 | NA | NA | 1 | 912 | G2 |
| TCGA-66-2758 | 71 | MALE | NA | T2 | N0 | M0 | I | NA | NA | Yes | NA | No | Chemotherapy | 0 | 639 | 0 | 639 | 0 | 639 | 0 | 639 | G2 |
| TCGA-66-2759 | 66 | MALE | NA | T2 | N2 | M0 | III | NA | NA | Yes | NA | No | Chemotherapy | 0 | 762 | 0 | 762 | 0 | 762 | 0 | 762 | G2 |
| TCGA-66-2763 | 63 | FEMALE | NA | T2 | N0 | M0 | I | NA | NA | Yes | NA | No | NA | 0 | 30 | 0 | 30 | NA | NA | 0 | 30 | G2 |
| TCGA-66-2765 | 64 | MALE | NA | T2 | N0 | M0 | I | NA | NA | Yes | NA | No | NA | 0 | 61 | 0 | 61 | NA | NA | 0 | 61 | G2 |
| TCGA-66-2766 | 54 | MALE | NA | T2 | N2 | M0 | III | NA | NA | Yes | NA | No | NA | 0 | 31 | 0 | 31 | NA | NA | 0 | 31 | G2 |
| TCGA-66-2767 | 62 | MALE | NA | T2 | N3 | M0 | III | NA | NA | Yes | NA | No | NA | 0 | 61 | 0 | 61 | NA | NA | 0 | 61 | G2 |
| TCGA-66-2768 | 57 | MALE | NA | T2 | N1 | M0 | II | NA | NA | Yes | NA | No | NA | 0 | 61 | 0 | 61 | NA | NA | 0 | 61 | G2 |
| TCGA-66-2769 | 75 | MALE | NA | T4 | N0 | M0 | III | NA | NA | Yes | NA | No | NA | 1 | 215 | NA | 215 | NA | NA | 0 | 215 | G2 |
| TCGA-66-2770 | 79 | MALE | NA | T2 | N0 | M0 | I | NA | NA | Yes | NA | No | NA | 0 | 700 | 0 | 700 | NA | NA | 0 | 700 | G2 |
| TCGA-66-2771 | 60 | MALE | NA | T2 | N1 | M0 | II | NA | NA | Yes | NA | No | Chemotherapy | 0 | 578 | 0 | 578 | 0 | 578 | 0 | 578 | G2 |
| TCGA-66-2773 | 69 | MALE | NA | T2 | N0 | M0 | I | NA | NA | Yes | NA | No | NA | 1 | 92 | 0 | 92 | NA | NA | 0 | 92 | G2 |
| TCGA-66-2777 | 71 | MALE | NA | T2 | N0 | M0 | I | NA | NA | Yes | NA | No | NA | 0 | 61 | 0 | 61 | NA | NA | 0 | 61 | G2 |
| TCGA-66-2778 | 68 | FEMALE | NA | T2 | N3 | M0 | III | NA | NA | Yes | NA | No | Chemotherapy | 0 | 578 | 0 | 578 | 0 | 578 | 0 | 578 | G2 |
| TCGA-66-2780 | 65 | MALE | NA | T2 | N0 | M0 | I | NA | NA | Yes | NA | No | NA | 1 | 366 | NA | 366 | NA | NA | 0 | 366 | G2 |
| TCGA-66-2781 | 67 | MALE | NA | T2 | N0 | M0 | I | NA | NA | Yes | NA | No | NA | 0 | 121 | 0 | 121 | NA | NA | 0 | 121 | G2 |
| TCGA-66-2782 | 71 | MALE | NA | T3 | N0 | M0 | II | NA | NA | Yes | NA | No | Chemotherapy | 1 | 365 | 1 | 365 | NA | NA | 1 | 304 | G2 |
| TCGA-66-2783 | 67 | MALE | NA | T2 | N3 | M0 | III | NA | NA | Yes | NA | No | Chemotherapy | 0 | 759 | 0 | 759 | 0 | 759 | 0 | 759 | G2 |
| TCGA-66-2785 | 65 | MALE | NA | T2 | N0 | M0 | I | NA | NA | Yes | NA | No | NA | 0 | 60 | 0 | 60 | NA | NA | 0 | 60 | G2 |
| TCGA-66-2786 | 68 | FEMALE | NA | T1 | N0 | M0 | I | NA | NA | Yes | NA | No | NA | 0 | 790 | 0 | 790 | NA | NA | 0 | 790 | G2 |
| TCGA-66-2787 | 57 | MALE | NA | T1 | N0 | M0 | I | NA | NA | Yes | NA | No | NA | 0 | 1217 | 0 | 1217 | NA | NA | 0 | 1217 | G2 |
| TCGA-66-2788 | 56 | MALE | NA | T2 | N0 | M0 | I | NA | NA | Yes | NA | No | NA | 0 | 699 | 0 | 699 | NA | NA | 0 | 699 | G2 |
| TCGA-66-2789 | 73 | MALE | NA | T1 | N3 | M0 | III | NA | NA | Yes | NA | No | NA | 1 | 123 | 0 | 123 | NA | NA | 0 | 123 | G2 |
| TCGA-66-2790 | 72 | MALE | NA | T2 | N1 | M0 | II | NA | NA | Yes | NA | No | Chemotherapy | 0 | 699 | 0 | 699 | NA | NA | 1 | 699 | G2 |
| TCGA-66-2791 | 66 | MALE | NA | T2 | N3 | M0 | III | NA | NA | Yes | NA | No | NA | 1 | 153 | NA | 153 | NA | NA | 0 | 153 | G2 |
| TCGA-66-2792 | 58 | MALE | NA | T2 | N1 | M0 | II | NA | NA | Yes | NA | No | Chemotherapy | 0 | 913 | 0 | 913 | 0 | 913 | 0 | 913 | G2 |
| TCGA-66-2793 | 68 | MALE | NA | T4 | N1 | M0 | III | NA | NA | Yes | NA | No | NA | 1 | 306 | 0 | 306 | NA | NA | 0 | 306 | G2 |
| TCGA-66-2794 | 64 | MALE | NA | T4 | N2 | M0 | III | NA | NA | Yes | NA | No | NA | 0 | 1645 | 0 | 1645 | 0 | 1645 | 0 | 1645 | G2 |
| TCGA-66-2795 | 68 | MALE | NA | T4 | N1 | M0 | III | NA | NA | Yes | NA | No | NA | 0 | 122 | 0 | 122 | NA | NA | 0 | 122 | G2 |
| TCGA-66-2800 | 70 | MALE | NA | T4 | N0 | M0 | III | NA | NA | Yes | NA | No | NA | 0 | 1492 | 0 | 1492 | NA | NA | 0 | 1492 | G2 |
| TCGA-67-3771 | 77 | FEMALE | BLACK | T1 | N0 | M0 | I | NA | NA | Yes | NA | No | NA | 0 | 610 | 0 | 610 | NA | NA | 0 | 610 | G2 |
| TCGA-68-7755 | 60 | FEMALE | WHITE | T1 | N1 | M0 | II | NA | NA | Yes | NA | No | Chemotherapy | 0 | 83 | 0 | 83 | NA | NA | 0 | 83 | G2 |
| TCGA-68-7756 | 84 | MALE | WHITE | T4 | N1 | NA | III | NA | NA | Yes | NA | No | Chemotherapy | 0 | 202 | 0 | 202 | NA | NA | 0 | 202 | G2 |
| TCGA-68-7757 | 74 | MALE | WHITE | T1 | N0 | NA | I | NA | NA | Yes | NO | No | NA | 0 | 211 | 0 | 211 | NA | NA | 0 | 211 | G2 |
| TCGA-68-8250 | 66 | MALE | BLACK | T1 | N0 | NA | I | NA | NA | Yes | NO | No | NA | 0 | 244 | 0 | 244 | NA | NA | 0 | 244 | G2 |
| TCGA-68-8251 | 78 | MALE | WHITE | T2 | N0 | M0 | I | NA | NA | Yes | NO | No | Chemotherapy | 0 | 406 | 0 | 406 | NA | NA | 0 | 406 | G2 |
| TCGA-68-A59I | 73 | FEMALE | BLACK | T3 | N1 | M0 | III | NA | Primary | Yes | NO | No | Chemotherapy | 0 | 492 | 0 | 492 | NA | NA | 1 | 159 | G2 |
| TCGA-68-A59J | 74 | FEMALE | WHITE | T2 | N0 | NA | I | NA | NA | Yes | NO | No | NA | 0 | 448 | 0 | 448 | 0 | 448 | 0 | 448 | G2 |
| TCGA-69-7760 | 73 | MALE | WHITE | T3 | N0 | M0 | II | NA | NA | No | NA | No | Chemotherapy | 0 | 202 | 0 | 202 | NA | NA | 0 | 202 | G2 |
| TCGA-69-7761 | 84 | MALE | WHITE | T2 | N0 | NA | I | NA | NA | Yes | NA | No | Chemotherapy | 0 | 186 | 0 | 186 | NA | NA | 0 | 186 | G2 |
| TCGA-69-7974 | 54 | FEMALE | WHITE | T2 | N2 | NA | III | NA | NA | Yes | NA | No | Chemotherapy | 0 | 184 | 0 | 184 | NA | NA | 0 | 184 | G2 |
| TCGA-69-7978 | 59 | MALE | WHITE | T2 | N1 | NA | II | NA | NA | Yes | NA | No | NA | 0 | 134 | 0 | 134 | NA | NA | 0 | 134 | G2 |
| TCGA-69-7979 | 71 | FEMALE | WHITE | T2 | N0 | NA | I | NA | NA | Yes | NA | No | NA | 0 | 408 | 0 | 408 | NA | NA | 0 | 408 | G2 |
| TCGA-69-8255 | 71 | MALE | WHITE | T1 | N0 | M0 | I | NA | NA | Yes | NO | No | NA | 0 | 129 | 0 | 129 | NA | NA | 0 | 129 | G2 |
| TCGA-69-A59K | 60 | FEMALE | BLACK | T3 | N0 | M0 | II | NA | NA | Yes | NO | No | Chemotherapy | 0 | 591 | 0 | 591 | NA | NA | 0 | 591 | G2 |
| TCGA-6A-AB49 | 73 | FEMALE | BLACK | T2 | N0 | NA | I | NA | NA | Yes | NO | No | NA | 1 | NA | 0 | NA | NA | NA | 0 | NA | G2 |
| TCGA-70-6722 | 47 | MALE | ASIAN | T3 | N1 | M0 | III | NA | Recurrence | No | NA | No | NA | 0 | 367 | 0 | 367 | NA | NA | 1 | 293 | G2 |
| TCGA-70-6723 | 65 | MALE | ASIAN | T3 | N0 | M0 | II | NA | Recurrence | No | NA | No | NA | 0 | 375 | 0 | 375 | NA | NA | 1 | 312 | G2 |
| TCGA-71-6725 | 48 | FEMALE | ASIAN | T2 | N0 | M0 | I | NA | Recurrence | Yes | NA | No | Chemotherapy | 0 | 256 | 0 | 256 | NA | NA | 1 | 164 | G2 |
| TCGA-73-4666 | 52 | FEMALE | WHITE | T1 | N0 | M1 | IV | NA | NA | Yes | NA | No | Chemotherapy | 0 | 800 | 0 | 800 | NA | NA | 0 | 800 | G2 |
| TCGA-73-4668 | 66 | FEMALE | AMERICAN INDIAN | T2 | N1 | M0 | II | NA | NA | Yes | NA | No | Chemotherapy | 0 | 467 | 0 | 467 | 1 | 236 | 1 | 236 | G2 |
| TCGA-73-4670 | 69 | FEMALE | WHITE | T2 | N0 | M1 | IV | NA | NA | Yes | NA | NA | Chemotherapy | 0 | 131 | 0 | 131 | NA | NA | 0 | 131 | G2 |
| TCGA-73-4675 | 59 | MALE | WHITE | T3 | N1 | M0 | III | NA | Recurrence | Yes | NA | No | Chemotherapy | 1 | 922 | 0 | 922 | NA | NA | 1 | 361 | G2 |
| TCGA-73-4676 | 45 | MALE | WHITE | T2 | N1 | M0 | II | NA | NA | Yes | NA | Yes | Chemotherapy | 1 | 281 | 1 | 281 | NA | NA | 1 | 281 | G2 |
| TCGA-73-7499 | 81 | FEMALE | WHITE | T2 | N0 | M0 | I | NA | Metastasis | No | NO | No | NA | 1 | 1531 | 0 | 1531 | 1 | 1447 | 1 | 1447 | G2 |
| TCGA-73-A9RS | 41 | MALE | BLACK | T3 | N0 | M0 | II | NA | Recurrence | Yes | YES | No | NA | 1 | 340 | 1 | 340 | NA | NA | 1 | 107 | G2 |
| TCGA-75-5122 | NA | MALE | NA | T2 | N0 | M0 | I | NA | NA | Yes | NA | No | NA | 1 | NA | 1 | NA | NA | NA | 1 | NA | G2 |
| TCGA-75-5125 | NA | MALE | NA | T2 | N1 | M0 | II | NA | NA | Yes | NA | No | NA | 1 | 2027 | 1 | 2027 | 1 | 1752 | 1 | 1752 | G2 |
| TCGA-75-5126 | NA | FEMALE | NA | T3 | N2 | M0 | III | NA | NA | Yes | NA | No | NA | 0 | NA | 0 | NA | NA | NA | 0 | NA | G2 |
| TCGA-75-5147 | NA | FEMALE | NA | T2 | N0 | M0 | I | NA | NA | No | NA | No | NA | 0 | 1333 | 0 | 1333 | 0 | 1333 | 0 | 1333 | G2 |
| TCGA-75-6205 | NA | FEMALE | NA | T2 | N0 | M0 | I | NA | NA | No | NA | No | NA | 1 | NA | 1 | NA | NA | NA | 1 | NA | G2 |
| TCGA-75-6207 | NA | MALE | NA | T2 | N2 | M0 | III | NA | NA | Yes | NA | No | NA | 1 | NA | NA | NA | 0 | NA | 0 | NA | G2 |
| TCGA-75-6211 | NA | FEMALE | NA | T2 | N0 | M0 | I | NA | NA | Yes | NA | No | NA | 1 | NA | 1 | NA | NA | NA | 1 | NA | G2 |
| TCGA-75-6214 | NA | FEMALE | NA | T2 | N2 | M0 | III | NA | Metastasis | Yes | NA | No | NA | 1 | 1115 | 1 | 1115 | 1 | 419 | 1 | 419 | G2 |
| TCGA-75-7027 | NA | MALE | NA | T2 | N0 | M0 | I | NA | Recurrence | Yes | NA | No | NA | 0 | 3059 | 0 | 3059 | 1 | 2949 | 1 | 2949 | G2 |
| TCGA-75-7031 | NA | FEMALE | NA | T2 | N0 | M0 | I | NA | NA | Yes | NA | No | NA | 0 | NA | 0 | NA | NA | NA | 0 | NA | G2 |
| TCGA-77-6842 | 79 | MALE | WHITE | T2 | N1 | M0 | II | NA | NA | Yes | NA | No | NA | 1 | 899 | NA | 899 | NA | NA | 1 | 201 | G2 |
| TCGA-77-6843 | 74 | MALE | WHITE | T1 | N1 | M0 | II | NA | NA | Yes | NA | No | NA | 1 | 2224 | NA | 2224 | NA | NA | 0 | 2224 | G2 |
| TCGA-77-6844 | 74 | MALE | WHITE | T3 | N1 | M0 | III | NA | NA | Yes | NA | No | NA | 1 | 2284 | NA | 2284 | 1 | 1788 | 1 | 1788 | G2 |
| TCGA-77-6845 | 69 | MALE | WHITE | T3 | N0 | M0 | II | NA | NA | Yes | NA | No | NA | 1 | 708 | NA | 708 | NA | NA | 0 | 708 | G2 |
| TCGA-77-7138 | 67 | MALE | NA | T2 | N0 | M0 | I | NA | NA | Yes | NA | No | NA | 1 | 340 | NA | 340 | NA | NA | 0 | 340 | G2 |
| TCGA-77-7139 | 56 | MALE | WHITE | T2 | N1 | M0 | II | NA | NA | Yes | NA | No | NA | 0 | 4261 | 0 | 4261 | 0 | 4261 | 0 | 4261 | G2 |
| TCGA-77-7140 | 69 | FEMALE | WHITE | T2 | N1 | M0 | II | NA | NA | No | NA | No | NA | 1 | 632 | NA | 632 | NA | NA | 1 | 250 | G2 |
| TCGA-77-7141 | 64 | MALE | WHITE | T2 | N0 | M0 | I | NA | NA | Yes | NA | No | NA | 0 | 15 | 0 | 15 | NA | NA | 0 | 15 | G2 |
| TCGA-77-7142 | 59 | FEMALE | WHITE | T2 | N0 | M0 | I | NA | NA | Yes | NA | No | NA | 0 | 2227 | 0 | 2227 | NA | NA | 0 | 2227 | G2 |
| TCGA-77-7335 | 62 | FEMALE | WHITE | T4 | N2 | M0 | III | NA | NA | Yes | NA | No | NA | 1 | 2133 | NA | 2133 | NA | NA | 0 | 2133 | G2 |
| TCGA-77-7337 | 65 | MALE | WHITE | T2 | N1 | M0 | II | NA | NA | Yes | NA | No | NA | 1 | 3253 | NA | 3253 | NA | NA | 0 | 3253 | G2 |
| TCGA-77-7338 | 64 | MALE | WHITE | T2 | N0 | M0 | I | NA | NA | Yes | NA | No | NA | 1 | 5 | NA | 5 | NA | NA | 0 | 5 | G2 |
| TCGA-77-7463 | 75 | MALE | WHITE | T2 | N0 | M0 | I | NA | NA | Yes | NA | No | NA | 1 | 1423 | NA | 1423 | NA | NA | 0 | 1423 | G2 |
| TCGA-77-7465 | 58 | MALE | WHITE | T2 | N1 | M0 | II | NA | NA | Yes | NA | No | NA | 0 | 990 | 0 | 990 | 0 | 990 | 0 | 990 | G2 |
| TCGA-77-8007 | 68 | MALE | NA | T2 | N1 | M0 | II | NA | NA | Yes | NA | No | NA | 1 | 198 | NA | 198 | NA | NA | 1 | 139 | G2 |
| TCGA-77-8008 | 68 | MALE | NA | T2 | N0 | M0 | I | NA | NA | Yes | NA | No | NA | 1 | 2639 | NA | 2639 | NA | NA | 0 | 2639 | G2 |
| TCGA-77-8009 | 68 | MALE | NA | T2 | N1 | M0 | II | NA | NA | Yes | NA | No | NA | 1 | 1695 | NA | 1695 | 1 | 607 | 1 | 607 | G2 |
| TCGA-77-8128 | 60 | MALE | NA | T2 | N2 | M0 | III | NA | NA | Yes | NO | No | NA | 1 | 1150 | NA | 1150 | NA | NA | 0 | 1150 | G2 |
| TCGA-77-8130 | 69 | MALE | NA | T2 | N1 | M0 | II | NA | Primary | Yes | NO | No | NA | 0 | 4765 | 0 | 4765 | 0 | 4765 | 1 | 4471 | G2 |
| TCGA-77-8131 | 72 | MALE | NA | T2 | NA | M0 | I | NA | NA | Yes | NA | No | NA | 1 | 383 | NA | 383 | NA | NA | 0 | 383 | G2 |
| TCGA-77-8133 | 74 | MALE | NA | T1 | N1 | M0 | II | NA | NA | Yes | NO | No | NA | 1 | 1640 | NA | 1640 | NA | NA | 0 | 1640 | G2 |
| TCGA-77-8136 | 74 | FEMALE | NA | T2 | N1 | M0 | II | NA | Metastasis | Yes | NO | No | NA | 1 | 1189 | NA | 1189 | NA | NA | 1 | 301 | G2 |
| TCGA-77-8138 | 74 | MALE | NA | T2 | N0 | M0 | I | NA | Recurrence | Yes | NO | No | NA | 1 | 539 | 1 | 539 | NA | NA | 1 | 289 | G2 |
| TCGA-77-8139 | 72 | MALE | NA | T3 | N0 | M0 | II | NA | NA | Yes | NO | No | NA | 0 | 3166 | 0 | 3166 | 0 | 3166 | 0 | 3166 | G2 |
| TCGA-77-8140 | 66 | FEMALE | NA | T2 | N1 | M0 | II | NA | NA | Yes | NO | No | NA | 1 | 351 | NA | 351 | NA | NA | 0 | 351 | G2 |
| TCGA-77-8143 | 76 | MALE | NA | T2 | N2 | M0 | III | NA | NA | Yes | YES | No | NA | 1 | 803 | 1 | 803 | NA | NA | 1 | 803 | G2 |
| TCGA-77-8144 | 70 | MALE | NA | T2 | N0 | M0 | I | NA | NA | Yes | NO | No | NA | 0 | 833 | 0 | 833 | 0 | 833 | 0 | 833 | G2 |
| TCGA-77-8145 | 73 | MALE | NA | T4 | N1 | M0 | III | NA | Metastasis | Yes | YES | No | NA | 1 | 212 | NA | 212 | NA | NA | 1 | 136 | G2 |
| TCGA-77-8146 | 72 | MALE | NA | T1 | N0 | M0 | I | NA | NA | Yes | NO | No | NA | 0 | 3189 | 0 | 3189 | 0 | 3189 | 0 | 3189 | G2 |
| TCGA-77-8148 | 68 | MALE | NA | T3 | N1 | M0 | III | NA | NA | Yes | NO | No | NA | 0 | 2023 | 0 | 2023 | 0 | 2023 | 0 | 2023 | G2 |
| TCGA-77-8150 | 64 | MALE | NA | T3 | N1 | M0 | III | NA | Recurrence | Yes | NO | No | NA | 1 | 1655 | 1 | 1655 | NA | NA | 1 | 1278 | G2 |
| TCGA-77-8153 | 77 | FEMALE | NA | T2 | N0 | M0 | I | NA | NA | Yes | NO | No | NA | 0 | 1992 | 0 | 1992 | 0 | 1992 | 0 | 1992 | G2 |
| TCGA-77-8154 | 67 | MALE | NA | T1 | N0 | M0 | I | NA | NA | Yes | NO | No | NA | 0 | 1841 | 0 | 1841 | 0 | 1841 | 0 | 1841 | G2 |
| TCGA-77-8156 | 60 | MALE | NA | T2 | N0 | M0 | I | NA | NA | Yes | NO | No | NA | 0 | 1106 | 0 | 1106 | 0 | 1106 | 0 | 1106 | G2 |
| TCGA-77-A5G1 | 75 | MALE | NA | T3 | N1 | M0 | III | NA | Primary | Yes | NO | No | NA | 0 | 4026 | 0 | 4026 | 0 | 4026 | 1 | 2197 | G2 |
| TCGA-77-A5G3 | 63 | MALE | NA | T2 | N1 | M0 | II | NA | NA | Yes | NO | No | NA | 0 | 4570 | 0 | 4570 | 0 | 4570 | 0 | 4570 | G2 |
| TCGA-77-A5G6 | 66 | MALE | NA | T2 | N2 | M0 | III | NA | Recurrence | Yes | NO | No | NA | 1 | 678 | 1 | 678 | 1 | 392 | 1 | 392 | G2 |
| TCGA-77-A5G7 | 63 | MALE | NA | T1 | N0 | M0 | I | NA | NA | Yes | NO | No | NA | 1 | 180 | NA | 180 | 0 | 180 | 0 | 180 | G2 |
| TCGA-77-A5G8 | 70 | MALE | NA | T3 | N0 | M0 | II | NA | NA | Yes | NA | No | NA | 0 | 1884 | 0 | 1884 | NA | NA | 0 | 1884 | G2 |
| TCGA-77-A5GA | 76 | MALE | NA | T2 | N0 | M0 | I | NA | NA | Yes | NO | No | NA | 0 | 1280 | 0 | 1280 | 0 | 1280 | 0 | 1280 | G2 |
| TCGA-77-A5GB | 90 | MALE | NA | T2 | NA | M0 | I | NA | Primary | Yes | NO | No | NA | 1 | 937 | NA | 937 | 0 | 937 | 1 | 229 | G2 |
| TCGA-77-A5GF | 70 | MALE | NA | T2 | N1 | M0 | II | NA | Recurrence | Yes | NO | No | NA | 1 | 840 | 1 | 840 | 1 | 722 | 1 | 722 | G2 |
| TCGA-77-A5GH | 81 | MALE | NA | T2 | N0 | M0 | I | NA | NA | Yes | NO | No | NA | 0 | 1182 | 0 | 1182 | 0 | 1182 | 0 | 1182 | G2 |
| TCGA-78-7145 | 52 | FEMALE | WHITE | T4 | N1 | M1 | IV | NA | NA | Yes | NA | No | NA | 1 | 826 | 1 | 826 | NA | NA | 1 | 422 | G2 |
| TCGA-78-7146 | 71 | FEMALE | WHITE | T2 | N2 | M0 | III | NA | NA | Yes | NA | No | NA | 1 | 173 | NA | 173 | NA | NA | 0 | 173 | G2 |
| TCGA-78-7147 | 67 | FEMALE | WHITE | T2 | N1 | M0 | II | NA | NA | Yes | NA | No | NA | 1 | 586 | 1 | 586 | NA | NA | 1 | 578 | G2 |
| TCGA-78-7148 | 71 | MALE | WHITE | T2 | N1 | M0 | II | NA | NA | Yes | NA | No | NA | 1 | 626 | NA | 626 | NA | NA | 1 | 182 | G2 |
| TCGA-78-7150 | 59 | MALE | WHITE | T2 | N1 | M0 | II | NA | NA | Yes | NA | No | Chemotherapy | 1 | 666 | 1 | 666 | NA | NA | 1 | 139 | G2 |
| TCGA-78-7153 | 65 | FEMALE | WHITE | T2 | N0 | M0 | I | NA | NA | Yes | NA | No | NA | 0 | 3635 | 0 | 3635 | 0 | 3635 | 0 | 3635 | G2 |
| TCGA-78-7154 | 72 | MALE | WHITE | T3 | N2 | M0 | III | NA | NA | Yes | NA | No | NA | 1 | 593 | NA | 593 | NA | NA | 0 | 593 | G2 |
| TCGA-78-7155 | 68 | MALE | WHITE | T2 | N0 | M0 | I | NA | NA | Yes | NA | No | Chemotherapy | 1 | 1171 | NA | 1171 | NA | NA | 1 | 209 | G2 |
| TCGA-78-7159 | 60 | FEMALE | WHITE | T1 | NA | M0 | I | NA | NA | Yes | NA | No | NA | 0 | 1974 | 0 | 1974 | 0 | 1974 | 0 | 1974 | G2 |
| TCGA-78-7161 | 69 | FEMALE | WHITE | T3 | N0 | M0 | II | NA | NA | Yes | NA | No | Chemotherapy | 1 | 291 | 1 | 291 | NA | NA | 1 | 162 | G2 |
| TCGA-78-7163 | 60 | MALE | WHITE | T2 | N0 | M0 | I | NA | NA | Yes | NA | No | NA | 0 | 7248 | 0 | 7248 | 0 | 7248 | 0 | 7248 | G2 |
| TCGA-78-7166 | 84 | MALE | WHITE | T2 | N1 | M0 | II | NA | NA | Yes | NA | No | NA | 1 | 258 | NA | 258 | NA | NA | 0 | 258 | G2 |
| TCGA-78-7220 | 53 | FEMALE | WHITE | T2 | N2 | M0 | III | NA | NA | Yes | NA | No | NA | 1 | 807 | 1 | 807 | NA | NA | 1 | 531 | G2 |
| TCGA-78-7535 | 45 | MALE | WHITE | T2 | N0 | M0 | I | NA | NA | Yes | NA | No | Chemotherapy | 1 | 949 | 1 | 949 | NA | NA | 1 | 809 | G2 |
| TCGA-78-7536 | 69 | MALE | WHITE | T2 | N2 | M0 | III | NA | NA | Yes | NA | No | NA | 1 | 244 | 1 | 244 | NA | NA | 1 | 231 | G2 |
| TCGA-78-7540 | 66 | FEMALE | WHITE | T2 | N0 | M0 | I | NA | NA | No | NA | No | NA | 1 | 1197 | NA | 1197 | 0 | 1197 | 0 | 1197 | G2 |
| TCGA-78-7542 | 56 | MALE | WHITE | T2 | N0 | M0 | I | NA | NA | Yes | NA | No | NA | 1 | 321 | NA | 321 | NA | NA | 0 | 321 | G2 |
| TCGA-78-8640 | 59 | MALE | NA | T1 | N1 | M0 | II | NA | NA | Yes | NO | No | NA | 0 | 7062 | 0 | 7062 | 0 | 7062 | 0 | 7062 | G2 |
| TCGA-78-8660 | 69 | MALE | NA | T2 | N1 | M0 | II | NA | Metastasis | Yes | YES | No | NA | 1 | 321 | 1 | 321 | NA | NA | 1 | 242 | G2 |
| TCGA-78-8662 | 53 | FEMALE | NA | T2 | N0 | M0 | I | NA | Recurrence | Yes | NO | No | NA | 1 | 3361 | NA | 3361 | 1 | 3044 | 1 | 3044 | G2 |
| TCGA-79-5596 | NA | MALE | NA | T3 | N1 | M0 | III | NA | NA | Yes | NA | No | NA | 0 | NA | 0 | NA | NA | NA | 0 | NA | G2 |
| TCGA-80-5608 | NA | FEMALE | NA | T1 | N0 | M0 | I | NA | NA | Yes | NA | No | NA | 0 | 2832 | 0 | 2832 | 0 | 2832 | 0 | 2832 | G2 |
| TCGA-80-5611 | NA | MALE | NA | T2 | N0 | M0 | I | NA | NA | Yes | NA | No | NA | 0 | 2595 | 0 | 2595 | 0 | 2595 | 0 | 2595 | G2 |
| TCGA-83-5908 | 59 | FEMALE | WHITE | T1 | N0 | M0 | I | NA | NA | Yes | NA | No | NA | 0 | 824 | 0 | 824 | NA | NA | 0 | 824 | G2 |
| TCGA-85-6175 | 63 | FEMALE | WHITE | T3 | N0 | M0 | II | NA | NA | No | NA | No | NA | 1 | 294 | 1 | 294 | NA | NA | 1 | 294 | G2 |
| TCGA-85-6560 | 59 | MALE | WHITE | T1 | N1 | M0 | II | NA | NA | Yes | NA | No | Chemotherapy | 0 | 1259 | 0 | 1259 | 0 | 1259 | 0 | 1259 | G2 |
| TCGA-85-6561 | 66 | MALE | WHITE | T2 | NA | M0 | I | NA | NA | Yes | NA | No | Chemotherapy | 0 | 1224 | 0 | 1224 | 0 | 1224 | 0 | 1224 | G2 |
| TCGA-85-6798 | 57 | MALE | WHITE | T3 | N1 | M0 | III | NA | Metastasis | Yes | NA | No | Chemotherapy | 1 | 195 | 1 | 195 | 1 | 180 | 1 | 180 | G2 |
| TCGA-85-7696 | 64 | MALE | WHITE | T1 | N0 | M0 | I | NA | NA | Yes | NA | No | NA | 0 | 1111 | 0 | 1111 | 0 | 1111 | 0 | 1111 | G2 |
| TCGA-85-7697 | 49 | MALE | WHITE | T3 | N0 | M0 | II | NA | NA | Yes | NA | No | Chemotherapy | 0 | 1063 | 0 | 1063 | 0 | 1063 | 0 | 1063 | G2 |
| TCGA-85-7698 | 48 | MALE | WHITE | T1 | N0 | M0 | I | NA | Metastasis | Yes | NA | No | Chemotherapy | 0 | 952 | 0 | 952 | 1 | 650 | 1 | 650 | G2 |
| TCGA-85-7699 | 73 | MALE | WHITE | T4 | N0 | M0 | III | NA | Metastasis | Yes | NA | No | Chemotherapy | 1 | 1001 | 1 | 1001 | 1 | 760 | 1 | 760 | G2 |
| TCGA-85-7710 | 59 | FEMALE | WHITE | T1 | N0 | M0 | I | NA | NA | Yes | NA | No | NA | 0 | 42 | 0 | 42 | 0 | 42 | 0 | 42 | G2 |
| TCGA-85-7843 | 50 | MALE | WHITE | T2 | N1 | M0 | II | NA | NA | Yes | NA | No | NA | 0 | 35 | 0 | 35 | NA | NA | 0 | 35 | G2 |
| TCGA-85-7844 | 71 | MALE | WHITE | T2 | N0 | M0 | I | NA | NA | Yes | NA | No | NA | 0 | 911 | 0 | 911 | 0 | 911 | 0 | 911 | G2 |
| TCGA-85-7950 | 46 | MALE | WHITE | T2 | N0 | M0 | I | NA | NA | Yes | NA | No | NA | 0 | 576 | 0 | 576 | 0 | 576 | 0 | 576 | G2 |
| TCGA-85-8049 | 57 | MALE | WHITE | T2 | N0 | M0 | I | NA | NA | No | NA | No | NA | 0 | 579 | 0 | 579 | 0 | 579 | 0 | 579 | G2 |
| TCGA-85-8052 | 53 | MALE | WHITE | T3 | N0 | M0 | II | NA | NA | Yes | NA | No | NA | 0 | 734 | 0 | 734 | 0 | 734 | 0 | 734 | G2 |
| TCGA-85-8070 | 71 | MALE | WHITE | T2 | N0 | M0 | I | NA | NA | Yes | NA | No | NA | 0 | 960 | 0 | 960 | 0 | 960 | 0 | 960 | G2 |
| TCGA-85-8071 | 52 | MALE | WHITE | T1 | N1 | M0 | II | NA | NA | Yes | NA | No | NA | 0 | 815 | 0 | 815 | 0 | 815 | 0 | 815 | G2 |
| TCGA-85-8072 | 60 | MALE | WHITE | T1 | N0 | M0 | I | NA | NA | Yes | NO | No | NA | 0 | 932 | 0 | 932 | 0 | 932 | 0 | 932 | G2 |
| TCGA-85-8276 | 62 | MALE | WHITE | T1 | N1 | M0 | II | NA | Metastasis | Yes | NA | No | NA | 0 | 1050 | 0 | 1050 | 1 | 1048 | 1 | 1048 | G2 |
| TCGA-85-8277 | 70 | MALE | WHITE | T3 | N1 | M0 | III | NA | NA | No | NA | No | NA | 1 | 307 | 0 | 307 | 0 | 307 | 0 | 307 | G2 |
| TCGA-85-8287 | 72 | MALE | WHITE | T1 | N0 | M0 | I | NA | NA | Yes | NA | No | NA | 1 | 23 | 0 | 23 | 0 | 23 | 0 | 23 | G2 |
| TCGA-85-8288 | 70 | MALE | WHITE | T1 | N1 | M0 | II | NA | NA | Yes | NA | No | NA | 1 | 402 | 0 | 402 | 0 | 402 | 0 | 402 | G2 |
| TCGA-85-8350 | 61 | MALE | WHITE | T1 | N0 | M0 | I | NA | NA | Yes | NA | No | NA | 0 | 683 | 0 | 683 | 0 | 683 | 0 | 683 | G2 |
| TCGA-85-8351 | 72 | MALE | WHITE | T2 | N1 | M0 | II | NA | NA | Yes | NA | No | Chemotherapy | 0 | 510 | 0 | 510 | 0 | 510 | 0 | 510 | G2 |
| TCGA-85-8352 | 67 | MALE | WHITE | T3 | N1 | M0 | III | NA | Metastasis | Yes | NA | No | NA | 1 | 161 | 1 | 161 | 1 | 128 | 1 | 128 | G2 |
| TCGA-85-8353 | 72 | MALE | WHITE | T3 | N1 | M0 | III | NA | NA | Yes | NA | No | NA | 1 | 94 | 1 | 94 | NA | NA | 1 | 94 | G2 |
| TCGA-85-8354 | 53 | MALE | WHITE | T2 | N0 | M0 | I | NA | NA | Yes | NA | No | NA | 0 | 995 | 0 | 995 | 0 | 995 | 0 | 995 | G2 |
| TCGA-85-8355 | 63 | MALE | WHITE | T1 | N0 | M0 | I | NA | NA | No | NA | No | NA | 0 | 61 | 0 | 61 | 0 | 61 | 0 | 61 | G2 |
| TCGA-85-8479 | 66 | MALE | WHITE | T1 | N0 | M0 | I | NA | NA | Yes | NA | No | NA | 0 | 468 | 0 | 468 | 0 | 468 | 0 | 468 | G2 |
| TCGA-85-8481 | 70 | MALE | WHITE | T3 | N0 | M0 | II | NA | NA | Yes | NA | No | NA | 1 | 236 | 0 | 236 | NA | NA | 0 | 236 | G2 |
| TCGA-85-8580 | 52 | FEMALE | WHITE | T2 | N0 | M0 | I | NA | NA | Yes | NA | No | NA | 0 | 1113 | 0 | 1113 | 0 | 1113 | 0 | 1113 | G2 |
| TCGA-85-8582 | 49 | MALE | WHITE | T1 | N0 | M0 | I | NA | NA | Yes | NA | No | NA | 0 | 1160 | 0 | 1160 | 0 | 1160 | 0 | 1160 | G2 |
| TCGA-85-8584 | 71 | MALE | WHITE | T2 | N1 | M0 | II | NA | NA | Yes | NA | No | NA | 1 | 383 | 0 | 383 | 0 | 383 | 0 | 383 | G2 |
| TCGA-85-8664 | 73 | MALE | WHITE | T2 | N1 | M0 | II | NA | Metastasis | Yes | NA | No | Chemotherapy | 1 | 550 | 1 | 550 | 1 | 434 | 1 | 434 | G2 |
| TCGA-85-8666 | 65 | MALE | WHITE | T2 | N0 | M0 | I | NA | NA | Yes | NA | No | NA | 1 | 687 | 0 | 687 | 0 | 687 | 0 | 687 | G2 |
| TCGA-85-A4CL | 65 | MALE | WHITE | T1 | N0 | M0 | I | NA | NA | Yes | NA | No | NA | 1 | 921 | 0 | 921 | 0 | 921 | 0 | 921 | G2 |
| TCGA-85-A4CN | 56 | FEMALE | WHITE | T2 | N1 | M0 | II | NA | NA | Yes | NA | No | Chemotherapy | 0 | 1038 | 0 | 1038 | 0 | 1038 | 0 | 1038 | G2 |
| TCGA-85-A4JB | 74 | MALE | WHITE | T3 | N0 | M0 | II | NA | NA | Yes | NA | No | Chemotherapy | 0 | 942 | 0 | 942 | 0 | 942 | 0 | 942 | G2 |
| TCGA-85-A4JC | 84 | MALE | WHITE | T2 | N0 | M0 | II | NA | Recurrence | Yes | NA | No | Chemotherapy | 1 | 604 | 1 | 604 | 1 | 548 | 1 | 548 | G2 |
| TCGA-85-A4QQ | 68 | MALE | WHITE | T2 | N0 | M0 | I | NA | NA | Yes | NA | No | NA | 1 | 927 | 0 | 927 | 0 | 927 | 0 | 927 | G2 |
| TCGA-85-A4QR | 67 | MALE | WHITE | T2 | N0 | M0 | I | NA | NA | Yes | NA | No | NA | 0 | 600 | 0 | 600 | 0 | 600 | 0 | 600 | G2 |
| TCGA-85-A50M | 47 | MALE | ASIAN | T2 | N0 | M0 | II | NA | NA | Yes | NO | No | NA | 1 | 826 | 0 | 826 | 0 | 826 | 0 | 826 | G2 |
| TCGA-85-A50Z | 57 | MALE | WHITE | T2 | N0 | M0 | II | NA | Primary | Yes | NA | No | Chemotherapy | 0 | 493 | 0 | 493 | 0 | 493 | 1 | 462 | G2 |
| TCGA-85-A510 | 74 | FEMALE | WHITE | T2 | N1 | M0 | II | NA | Metastasis | Yes | NA | No | NA | 0 | 482 | 0 | 482 | 1 | 363 | 1 | 363 | G2 |
| TCGA-85-A511 | 62 | MALE | WHITE | T2 | N1 | M0 | II | NA | Metastasis | Yes | NA | No | NA | 1 | 455 | 1 | 455 | 1 | 390 | 1 | 390 | G2 |
| TCGA-85-A512 | 46 | MALE | WHITE | T1 | N1 | M0 | II | NA | NA | Yes | NA | No | Chemotherapy | 0 | 465 | 0 | 465 | 0 | 465 | 0 | 465 | G2 |
| TCGA-85-A53L | 63 | MALE | ASIAN | T2 | N0 | M0 | II | NA | NA | Yes | NA | No | NA | 0 | 377 | 0 | 377 | 0 | 377 | 0 | 377 | G2 |
| TCGA-85-A5B5 | 58 | MALE | WHITE | T1 | N0 | M0 | I | NA | NA | Yes | NO | No | NA | 0 | 111 | 0 | 111 | 0 | 111 | 0 | 111 | G2 |
| TCGA-86-6562 | 52 | MALE | WHITE | T2 | N1 | M0 | II | NA | NA | No | NA | No | Chemotherapy | 1 | 376 | 1 | 376 | NA | NA | 1 | 274 | G2 |
| TCGA-86-6851 | 73 | FEMALE | WHITE | T1 | N1 | M0 | II | NA | NA | Yes | NA | No | NA | 0 | 179 | 0 | 179 | 0 | 179 | 0 | 179 | G2 |
| TCGA-86-7701 | 66 | MALE | WHITE | T2 | N0 | M1 | IV | NA | Metastasis | No | NA | No | NA | 0 | 947 | 0 | 947 | NA | NA | 1 | 424 | G2 |
| TCGA-86-7711 | 70 | MALE | WHITE | T2 | N1 | M0 | II | NA | NA | Yes | NA | No | Chemotherapy | 1 | 1046 | 1 | 1046 | NA | NA | 1 | 1046 | G2 |
| TCGA-86-7713 | 70 | MALE | WHITE | T2 | N0 | M0 | II | NA | NA | No | NA | No | Chemotherapy | 0 | 1157 | 0 | 1157 | 0 | 1157 | 0 | 1157 | G2 |
| TCGA-86-7953 | 69 | FEMALE | WHITE | T1 | N0 | M0 | I | NA | NA | No | NA | No | NA | 0 | 997 | 0 | 997 | 0 | 997 | 0 | 997 | G2 |
| TCGA-86-7954 | 68 | FEMALE | WHITE | T2 | N0 | M0 | I | NA | NA | Yes | NA | No | Chemotherapy | 0 | 605 | 0 | 605 | 0 | 605 | 0 | 605 | G2 |
| TCGA-86-7955 | 62 | MALE | WHITE | T2 | N0 | M0 | I | NA | Recurrence | No | NA | No | Chemotherapy | 0 | 1072 | 0 | 1072 | 1 | 820 | 1 | 820 | G2 |
| TCGA-86-8054 | 61 | MALE | WHITE | T2 | N1 | M0 | II | NA | NA | Yes | NA | No | Chemotherapy | 0 | 1148 | 0 | 1148 | 0 | 1148 | 0 | 1148 | G2 |
| TCGA-86-8279 | 46 | MALE | WHITE | T2 | N1 | M0 | II | NA | NA | No | YES | No | Chemotherapy | 0 | 949 | 0 | 949 | 0 | 949 | 0 | 949 | G2 |
| TCGA-86-8358 | 44 | MALE | WHITE | T2 | N0 | M0 | I | NA | NA | Yes | NA | No | NA | 0 | 653 | 0 | 653 | 0 | 653 | 0 | 653 | G2 |
| TCGA-86-8359 | 52 | MALE | WHITE | T3 | N2 | M0 | III | NA | NA | Yes | NA | No | NA | 1 | 444 | 0 | 444 | NA | NA | 0 | 444 | G2 |
| TCGA-86-8585 | 57 | MALE | WHITE | T2 | N0 | M0 | I | NA | NA | No | NA | No | NA | 0 | 353 | 0 | 353 | 0 | 353 | 0 | 353 | G2 |
| TCGA-86-8669 | 64 | MALE | WHITE | T1 | N0 | M0 | I | NA | Metastasis | Yes | NA | No | Chemotherapy | 0 | 938 | 0 | 938 | 1 | 849 | 1 | 849 | G2 |
| TCGA-86-8672 | 59 | MALE | WHITE | T3 | N0 | M0 | II | NA | NA | No | NA | No | NA | 1 | 19 | 0 | 19 | 0 | 19 | 0 | 19 | G2 |
| TCGA-86-8673 | 61 | MALE | WHITE | T2 | N0 | M0 | I | NA | Recurrence | Yes | NA | No | NA | 0 | 862 | 0 | 862 | 1 | 636 | 1 | 636 | G2 |
| TCGA-86-8674 | 50 | MALE | WHITE | T2 | N1 | M0 | II | NA | Recurrence | Yes | NA | No | Chemotherapy | 0 | 806 | 0 | 806 | 1 | 334 | 1 | 334 | G2 |
| TCGA-86-A4D0 | 48 | MALE | WHITE | T2 | N0 | M0 | II | NA | NA | Yes | NA | No | Chemotherapy | 1 | 116 | 0 | 116 | 0 | 116 | 0 | 116 | G2 |
| TCGA-86-A4JF | 56 | MALE | WHITE | T3 | N0 | M0 | II | NA | Metastasis | Yes | NA | No | Chemotherapy | 1 | 737 | 1 | 737 | 1 | 436 | 1 | 436 | G2 |
| TCGA-90-6837 | 64 | MALE | WHITE | T3 | N0 | NA | II | NA | NA | Yes | NA | No | Chemotherapy | 0 | 758 | 0 | 758 | 0 | 758 | 0 | 758 | G2 |
| TCGA-90-7766 | 66 | FEMALE | WHITE | T1 | N0 | NA | I | NA | Recurrence | Yes | NA | No | NA | 0 | 289 | 0 | 289 | NA | NA | 1 | 289 | G2 |
| TCGA-90-7767 | 56 | MALE | WHITE | T2 | N1 | NA | II | NA | NA | Yes | NA | No | NA | 0 | 89 | 0 | 89 | NA | NA | 0 | 89 | G2 |
| TCGA-90-7769 | 55 | MALE | WHITE | T2 | N1 | NA | II | NA | NA | Yes | NA | No | NA | 0 | 358 | 0 | 358 | 0 | 358 | 0 | 358 | G2 |
| TCGA-90-7964 | 70 | MALE | WHITE | T2 | N0 | NA | I | NA | NA | Yes | NA | No | NA | 0 | 428 | 0 | 428 | 0 | 428 | 0 | 428 | G2 |
| TCGA-90-A4ED | 69 | MALE | WHITE | T2 | N0 | NA | I | NA | NA | Yes | NO | No | NA | 0 | 615 | 0 | 615 | 0 | 615 | 0 | 615 | G2 |
| TCGA-90-A4EE | 53 | MALE | WHITE | T2 | N1 | NA | II | NA | NA | Yes | NO | No | Chemotherapy | 0 | 688 | 0 | 688 | 0 | 688 | 0 | 688 | G2 |
| TCGA-90-A59Q | 61 | FEMALE | WHITE | T2 | N1 | NA | II | NA | Metastasis | Yes | NO | No | Chemotherapy | 1 | 322 | 1 | 322 | 1 | 274 | 1 | 274 | G2 |
| TCGA-91-6829 | 78 | MALE | WHITE | T2 | N0 | NA | I | NA | NA | Yes | NA | No | NA | 1 | 1258 | 0 | 1258 | NA | NA | 0 | 1258 | G2 |
| TCGA-91-6831 | 66 | MALE | WHITE | T2 | N0 | NA | I | NA | NA | Yes | NA | No | NA | 0 | 310 | 0 | 310 | 0 | 310 | 0 | 310 | G2 |
| TCGA-91-6836 | 52 | FEMALE | WHITE | T2 | N0 | NA | I | NA | NA | Yes | NA | No | NA | 0 | 417 | 0 | 417 | 0 | 417 | 0 | 417 | G2 |
| TCGA-91-6840 | 59 | FEMALE | WHITE | T1 | N0 | M0 | I | NA | NA | Yes | NA | No | NA | 0 | 372 | 0 | 372 | 0 | 372 | 0 | 372 | G2 |
| TCGA-91-6847 | 62 | FEMALE | WHITE | T2 | N0 | NA | I | NA | Metastasis | Yes | NA | No | NA | 0 | 842 | 0 | 842 | NA | NA | 1 | 772 | G2 |
| TCGA-91-6848 | 59 | MALE | WHITE | T2 | N2 | NA | III | NA | NA | Yes | NA | No | Chemotherapy | 0 | 224 | 0 | 224 | 0 | 224 | 0 | 224 | G2 |
| TCGA-91-8499 | 76 | FEMALE | WHITE | T1 | N0 | NA | I | NA | NA | Yes | NO | No | NA | 0 | 36 | 0 | 36 | NA | NA | 0 | 36 | G2 |
| TCGA-91-A4BC | 59 | MALE | WHITE | T2 | N0 | NA | II | NA | NA | Yes | NA | No | NA | 0 | 44 | 0 | 44 | NA | NA | 0 | 44 | G2 |
| TCGA-92-7340 | 45 | FEMALE | WHITE | T2 | N1 | NA | II | NA | NA | Yes | NA | No | Chemotherapy | 0 | 82 | 0 | 82 | NA | NA | 0 | 82 | G2 |
| TCGA-92-7341 | 71 | MALE | WHITE | T2 | N0 | NA | I | NA | NA | Yes | NA | No | NA | 0 | 106 | 0 | 106 | NA | NA | 0 | 106 | G2 |
| TCGA-92-8063 | 52 | MALE | WHITE | T2 | N2 | NA | III | NA | NA | Yes | YES | No | Chemotherapy | 0 | 122 | 0 | 122 | 0 | 122 | 0 | 122 | G2 |
| TCGA-92-8064 | 58 | MALE | WHITE | T2 | N0 | NA |  | NA | NA | Yes | NO | No | Chemotherapy | 0 | 160 | 0 | 160 | 0 | 160 | 0 | 160 | G2 |
| TCGA-92-8065 | 74 | FEMALE | WHITE | T3 | N0 | NA | II | NA | NA | Yes | NO | No | NA | 0 | 70 | 0 | 70 | 0 | 70 | 0 | 70 | G2 |
| TCGA-93-8067 | 77 | MALE | ASIAN | T2 | N0 | NA | I | NA | NA | Yes | NO | No | NA | 0 | 186 | 0 | 186 | 0 | 186 | 0 | 186 | G2 |
| TCGA-94-7033 | 73 | MALE | WHITE | T2 | N0 | NA | I | NA | NA | Yes | NA | No | Chemotherapy | 0 | 640 | 0 | 640 | 0 | 640 | 0 | 640 | G2 |
| TCGA-94-7557 | 73 | MALE | BLACK | T2 | N0 | M0 | I | NA | NA | Yes | NA | No | NA | 1 | 5 | 0 | 5 | 0 | 5 | 0 | 5 | G2 |
| TCGA-94-7943 | 80 | MALE | WHITE | T1 | NA | NA | I | NA | Recurrence | Yes | NA | No | NA | 0 | 559 | 0 | 559 | 1 | 556 | 1 | 556 | G2 |
| TCGA-94-8035 | 64 | MALE | WHITE | T3 | N0 | NA | II | NA | NA | Yes | NO | No | Chemotherapy | 0 | 122 | 0 | 122 | 0 | 122 | 0 | 122 | G2 |
| TCGA-94-8490 | 70 | MALE | WHITE | T3 | N0 | M0 | II | NA | NA | Yes | NO | No | NA | 0 | 153 | 0 | 153 | NA | NA | 0 | 153 | G2 |
| TCGA-94-8491 | 73 | MALE | WHITE | T2 | N1 | M0 | II | NA | Metastasis | Yes | NO | No | Chemotherapy | 0 | 810 | 0 | 810 | NA | NA | 1 | 641 | G2 |
| TCGA-94-A4VJ | 71 | FEMALE | WHITE | T1 | N0 | M0 | I | NA | NA | Yes | NO | No | NA | 0 | 430 | 0 | 430 | 0 | 430 | 0 | 430 | G2 |
| TCGA-94-A5I4 | 61 | MALE | WHITE | T2 | N1 | NA | II | NA | Metastasis | Yes | NO | No | NA | 0 | 491 | 0 | 491 | 1 | 314 | 1 | 314 | G2 |
| TCGA-94-A5I6 | 62 | MALE | WHITE | T3 | N0 | M0 | II | NA | Metastasis | Yes | NO | No | Chemotherapy | 0 | 538 | 0 | 538 | NA | NA | 1 | 481 | G2 |
| TCGA-95-7043 | 63 | FEMALE | WHITE | T1 | N0 | NA | I | NA | NA | Yes | NA | No | NA | 1 | 503 | 1 | 503 | NA | NA | 1 | 503 | G2 |
| TCGA-95-7562 | 71 | MALE | BLACK | T2 | N1 | M0 | II | NA | NA | Yes | NO | No | Chemotherapy | 1 | 87 | 0 | 87 | NA | NA | 0 | 87 | G2 |
| TCGA-95-7567 | 61 | MALE | WHITE | T2 | N1 | M0 | II | NA | NA | Yes | NA | No | Chemotherapy | 0 | 568 | 0 | 568 | NA | NA | 0 | 568 | G2 |
| TCGA-95-7944 | 71 | MALE | WHITE | T1 | N0 | M0 | I | NA | NA | Yes | NA | No | NA | 0 | 377 | 0 | 377 | NA | NA | 0 | 377 | G2 |
| TCGA-95-7948 | 42 | FEMALE | WHITE | T2 | N0 | M0 | I | NA | NA | Yes | NA | No | NA | 0 | 476 | 0 | 476 | 0 | 476 | 0 | 476 | G2 |
| TCGA-95-8494 | 67 | MALE | WHITE | T2 | N1 | M0 | II | NA | NA | Yes | NO | No | NA | 0 | 84 | 0 | 84 | 0 | 84 | 0 | 84 | G2 |
| TCGA-95-A4VN | 62 | FEMALE | WHITE | T2 | N1 | M0 | II | NA | NA | Yes | NO | No | NA | 0 | 553 | 0 | 553 | NA | NA | 0 | 553 | G2 |
| TCGA-96-7544 | 83 | MALE | WHITE | T2 | N1 | NA | II | NA | NA | Yes | NA | No | NA | 1 | 2160 | 0 | 2160 | 0 | 2160 | 0 | 2160 | G2 |
| TCGA-96-7545 | 73 | MALE | WHITE | T1 | N0 | NA | I | NA | Recurrence | Yes | NA | No | NA | 1 | 1736 | NA | 1736 | NA | NA | 1 | 1693 | G2 |
| TCGA-96-8169 | 67 | FEMALE | WHITE | T1 | N0 | M0 | I | NA | NA | Yes | NO | No | NA | 0 | 557 | 0 | 557 | 0 | 557 | 0 | 557 | G2 |
| TCGA-96-8170 | 75 | FEMALE | WHITE | T1 | N1 | M0 | II | NA | NA | Yes | NO | No | NA | 0 | 531 | 0 | 531 | 0 | 531 | 0 | 531 | G2 |
| TCGA-96-A4JK | 65 | MALE | WHITE | T2 | N1 | M0 | II | NA | NA | Yes | NO | Yes | Chemotherapy | 0 | 589 | 0 | 589 | 0 | 589 | 0 | 589 | G2 |
| TCGA-96-A4JL | 78 | FEMALE | ASIAN | T2 | N1 | M0 | II | NA | NA | No | NO | No | Chemotherapy | 0 | 842 | 0 | 842 | 0 | 842 | 0 | 842 | G2 |
| TCGA-97-7554 | 83 | FEMALE | WHITE | T2 | N2 | M0 | III | NA | NA | Yes | NA | No | Chemotherapy | 0 | 775 | 0 | 775 | 0 | 775 | 0 | 775 | G2 |
| TCGA-97-7937 | 65 | MALE | WHITE | T2 | N0 | NA | I | NA | NA | Yes | NA | No | NA | 0 | 564 | 0 | 564 | NA | NA | 0 | 564 | G2 |
| TCGA-97-8176 | 63 | MALE | WHITE | T3 | N1 | M0 | III | NA | Metastasis | Yes | YES | No | NA | 1 | 468 | 0 | 468 | NA | NA | 1 | 39 | G2 |
| TCGA-98-8020 | 56 | FEMALE | BLACK | T2 | N2 | M0 | III | NA | Metastasis | No | NO | No | NA | 1 | 84 | 1 | 84 | NA | NA | 1 | 11 | G2 |
| TCGA-98-8021 | 75 | FEMALE | WHITE | T1 | N0 | M0 | I | NA | Recurrence | Yes | NO | No | NA | 0 | 937 | 0 | 937 | 1 | 918 | 1 | 918 | G2 |
| TCGA-98-8022 | 61 | MALE | WHITE | T1 | N0 | M0 | I | NA | NA | Yes | NO | No | NA | 1 | 933 | 0 | 933 | 0 | 933 | 0 | 933 | G2 |
| TCGA-98-8023 | 70 | MALE | WHITE | T3 | N1 | M0 | III | NA | NA | Yes | NO | No | NA | 0 | 649 | 0 | 649 | 0 | 649 | 0 | 649 | G2 |
| TCGA-98-A538 | 67 | MALE | WHITE | T3 | N0 | M0 | II | NA | NA | Yes | NO | No | NA | 0 | 826 | 0 | 826 | 0 | 826 | 0 | 826 | G2 |
| TCGA-98-A539 | 63 | MALE | WHITE | T3 | N0 | M0 | II | NA | Recurrence | Yes | NO | No | NA | 0 | 173 | 0 | 173 | NA | NA | 1 | 173 | G2 |
| TCGA-98-A53A | 70 | MALE | BLACK | T2 | N0 | M0 | I | NA | NA | Yes | NO | No | NA | 1 | 552 | NA | 552 | 0 | 552 | 0 | 552 | G2 |
| TCGA-98-A53B | 69 | MALE | WHITE | T2 | N0 | M0 | I | NA | NA | Yes | NO | No | NA | 1 | 61 | 0 | 61 | 0 | 61 | 0 | 61 | G2 |
| TCGA-98-A53I | 64 | MALE | WHITE | T2 | N1 | M0 | II | NA | NA | Yes | NO | No | Chemotherapy | 0 | 565 | 0 | 565 | 0 | 565 | 0 | 565 | G2 |
| TCGA-98-A53J | 77 | MALE | WHITE | T2 | N0 | M0 | I | NA | NA | Yes | NO | No | NA | 0 | 630 | 0 | 630 | 0 | 630 | 0 | 630 | G2 |
| TCGA-99-8028 | 50 | FEMALE | BLACK | T1 | N0 | M0 | I | NA | NA | Yes | NO | No | NA | 0 | 1118 | 0 | 1118 | 0 | 1118 | 0 | 1118 | G2 |
| TCGA-99-8032 | 61 | MALE | WHITE | T1 | N0 | M0 | I | NA | NA | Yes | NO | No | NA | 0 | 44 | 0 | 44 | 0 | 44 | 0 | 44 | G2 |
| TCGA-99-8033 | 74 | FEMALE | WHITE | NA | NA | M1 | IV | NA | NA | No | NO | No | Chemotherapy | 1 | 656 | 1 | 656 | NA | NA | 1 | 656 | G2 |
| TCGA-J1-A4AH | 70 | MALE | WHITE | T2 | N0 | NA | II | NA | NA | Yes | NO | No | Chemotherapy | 0 | 581 | 0 | 581 | 0 | 581 | 0 | 581 | G2 |
| TCGA-L3-A4E7 | 71 | MALE | WHITE | T2 | N0 | M0 | I | NA | NA | Yes | NO | No | NA | 0 | 392 | 0 | 392 | 0 | 392 | 0 | 392 | G2 |
| TCGA-L3-A524 | 45 | FEMALE | WHITE | T3 | N0 | M0 | II | NA | NA | Yes | NO | No | Chemotherapy | 1 | 490 | 0 | 490 | 0 | 490 | 0 | 490 | G2 |
| TCGA-L4-A4E5 | 48 | FEMALE | WHITE | T1 | N0 | M0 | I | NA | NA | Yes | NO | No | NA | 0 | 578 | 0 | 578 | 0 | 578 | 0 | 578 | G2 |
| TCGA-L9-A444 | 60 | FEMALE | WHITE | T1 | N0 | NA | I | NA | NA | Yes | NO | No | NA | 0 | 307 | 0 | 307 | 0 | 307 | 0 | 307 | G2 |
| TCGA-L9-A5IP | 40 | FEMALE | BLACK | T3 | N2 | M1 | IV | NA | NA | Yes | NO | No | NA | 1 | 58 | 1 | 58 | NA | NA | 1 | 49 | G2 |
| TCGA-L9-A8F4 | 64 | FEMALE | BLACK | T2 | N0 | NA | I | NA | NA | Yes | YES | No | NA | 0 | 476 | 0 | 476 | NA | NA | 0 | 476 | G2 |
| TCGA-LA-A446 | 68 | MALE | WHITE | T1 | N0 | NA | I | NA | NA | Yes | NO | No | NA | 0 | 401 | 0 | 401 | 0 | 401 | 0 | 401 | G2 |
| TCGA-LA-A7SW | 71 | MALE | BLACK | T3 | N1 | NA | III | NA | Recurrence | Yes | NO | No | Chemotherapy | 1 | 408 | 1 | 408 | NA | NA | 1 | 235 | G2 |
| TCGA-MF-A522 | 54 | MALE | WHITE | T2 | N0 | NA | I | NA | Metastasis | Yes | NO | No | Chemotherapy | 1 | 360 | 1 | 360 | NA | NA | 1 | 173 | G2 |
| TCGA-MN-A4N1 | 60 | MALE | BLACK | T2 | N1 | M0 | II | NA | NA | Yes | NO | No | NA | 0 | 827 | 0 | 827 | 0 | 827 | 0 | 827 | G2 |
| TCGA-MN-A4N4 | 57 | MALE | WHITE | T1 | N0 | M0 | I | NA | NA | Yes | NO | No | NA | 0 | 1175 | 0 | 1175 | 0 | 1175 | 0 | 1175 | G2 |
| TCGA-MP-A4T4 | 68 | FEMALE | WHITE | T2 | N1 | M0 | II | NA | NA | Yes | NO | No | NA | 1 | 2617 | NA | 2617 | 0 | 2617 | 0 | 2617 | G2 |
| TCGA-MP-A4T8 | 68 | MALE | NA | T2 | N2 | M0 | III | NA | NA | Yes | NO | No | Chemotherapy | 1 | 161 | NA | 161 | NA | NA | 0 | 161 | G2 |
| TCGA-MP-A4TA | 75 | FEMALE | WHITE | T1 | N0 | M0 | I | NA | Metastasis | Yes | NO | No | NA | 1 | 950 | 1 | 950 | 1 | 725 | 1 | 725 | G2 |
| TCGA-MP-A4TC | 77 | MALE | WHITE | T1 | N2 | M0 | III | NA | NA | Yes | NO | No | Chemotherapy | 1 | 74 | 0 | 74 | 0 | 74 | 0 | 74 | G2 |
| TCGA-MP-A4TD | 71 | MALE | WHITE | T2 | N2 | M0 | III | NA | Recurrence | Yes | NO | No | Chemotherapy | 1 | 307 | NA | 307 | 1 | 226 | 1 | 226 | G2 |
| TCGA-MP-A4TE | 56 | MALE | WHITE | T2 | N0 | NA | II | NA | Recurrence | Yes | NO | No | NA | 1 | 896 | 1 | 896 | 1 | 226 | 1 | 226 | G2 |
| TCGA-MP-A4TF | 58 | FEMALE | WHITE | T2 | N0 | M0 | II | NA | Metastasis | Yes | NO | No | Chemotherapy | 1 | 336 | 1 | 336 | NA | NA | 1 | 195 | G2 |
| TCGA-MP-A4TI | 72 | MALE | WHITE | T2 | N1 | M0 | II | NA | NA | Yes | NA | No | NA | 1 | 429 | 1 | 429 | NA | NA | 1 | 84 | G2 |
| TCGA-NC-A5HD | 79 | MALE | WHITE | T3 | N0 | M0 | II | NA | NA | Yes | NO | No | NA | 1 | 2 | 0 | 2 | NA | NA | 0 | 2 | G2 |
| TCGA-NC-A5HE | 60 | MALE | WHITE | T2 | N1 | M0 | II | NA | NA | Yes | NO | No | Chemotherapy | 0 | 2336 | 0 | 2336 | 0 | 2336 | 0 | 2336 | G2 |
| TCGA-NC-A5HF | 74 | MALE | WHITE | T4 | N0 | NA | III | NA | Metastasis | Yes | NO | No | NA | 1 | 138 | 1 | 138 | NA | NA | 1 | 132 | G2 |
| TCGA-NC-A5HG | 59 | MALE | WHITE | T2 | N2 | M0 | III | NA | NA | Yes | YES | No | Chemotherapy | 0 | 1963 | 0 | 1963 | 0 | 1963 | 0 | 1963 | G2 |
| TCGA-NC-A5HH | 53 | MALE | WHITE | T1 | N0 | M0 | I | NA | NA | Yes | NO | No | NA | 0 | 37 | 0 | 37 | 0 | 37 | 0 | 37 | G2 |
| TCGA-NC-A5HI | 68 | FEMALE | WHITE | T2 | N0 | M0 | I | NA | Primary | Yes | NO | No | Chemotherapy | 0 | 1743 | 0 | 1743 | 0 | 1743 | 1 | 70 | G2 |
| TCGA-NC-A5HK | 58 | FEMALE | WHITE | T3 | N0 | M0 | II | NA | NA | Yes | NO | No | Chemotherapy | 0 | 128 | 0 | 128 | 0 | 128 | 0 | 128 | G2 |
| TCGA-NC-A5HL | 73 | MALE | WHITE | T2 | N0 | M0 | II | NA | NA | Yes | NO | No | NA | 1 | 88 | 0 | 88 | NA | NA | 0 | 88 | G2 |
| TCGA-NC-A5HM | 76 | MALE | WHITE | T2 | N0 | M0 | I | NA | NA | Yes | NO | No | NA | 0 | 1212 | 0 | 1212 | 0 | 1212 | 0 | 1212 | G2 |
| TCGA-NC-A5HN | 77 | MALE | WHITE | T2 | N1 | M0 | II | NA | NA | Yes | NO | No | NA | 0 | 1499 | 0 | 1499 | 0 | 1499 | 0 | 1499 | G2 |
| TCGA-NC-A5HO | 70 | FEMALE | WHITE | T3 | N1 | M0 | III | NA | NA | Yes | NO | No | Chemotherapy | 0 | 1336 | 0 | 1336 | 0 | 1336 | 0 | 1336 | G2 |
| TCGA-NC-A5HP | 69 | MALE | WHITE | T2 | N0 | M1 | IV | NA | Metastasis | Yes | NO | No | Chemotherapy | 1 | 770 | 1 | 770 | NA | NA | 1 | 317 | G2 |
| TCGA-NC-A5HQ | 70 | MALE | WHITE | T3 | N2 | M0 | III | NA | NA | Yes | YES | No | Chemotherapy | 1 | 448 | NA | 448 | 0 | 448 | 0 | 448 | G2 |
| TCGA-NC-A5HR | 75 | FEMALE | WHITE | T2 | N1 | M0 | II | NA | NA | Yes | NO | No | Chemotherapy | 0 | 1244 | 0 | 1244 | 0 | 1244 | 0 | 1244 | G2 |
| TCGA-NC-A5HT | 69 | MALE | WHITE | T3 | N1 | M0 | III | NA | Recurrence | Yes | NO | No | Chemotherapy | 0 | 804 | 0 | 804 | 1 | 203 | 1 | 203 | G2 |
| TCGA-NJ-A4YF | 50 | FEMALE | BLACK | T1 | N0 | M0 | I | NA | NA | Yes | NA | No | NA | 0 | 2161 | 0 | 2161 | NA | NA | 0 | 2161 | G2 |
| TCGA-NJ-A4YQ | 69 | FEMALE | WHITE | T1 | N0 | M0 | I | NA | NA | Yes | NO | No | NA | 0 | 1432 | 0 | 1432 | NA | NA | 0 | 1432 | G2 |
| TCGA-NJ-A55R | 67 | MALE | WHITE | T1 | N0 | NA | I | NA | NA | Yes | NO | No | NA | 0 | 603 | 0 | 603 | NA | NA | 0 | 603 | G2 |
| TCGA-NK-A5CR | 77 | MALE | NA | T2 | N0 | NA | I | NA | NA | Yes | NO | No | NA | 0 | 2542 | 0 | 2542 | NA | NA | 0 | 2542 | G2 |
| TCGA-NK-A5CT | 70 | MALE | WHITE | T1 | N0 | M0 | I | NA | Recurrence | NA | NO | No | NA | 0 | 1997 | 0 | 1997 | NA | NA | 1 | 1990 | G2 |
| TCGA-NK-A5CX | 73 | MALE | WHITE | T2 | N0 | NA | II | NA | NA | Yes | NO | No | NA | 0 | 111 | 0 | 111 | NA | NA | 0 | 111 | G2 |
| TCGA-NK-A5D1 | 57 | MALE | WHITE | T2 | N1 | M0 | II | NA | Metastasis | NA | NA | No | Chemotherapy | 0 | 511 | 0 | 511 | NA | NA | 1 | 151 | G2 |
| TCGA-NK-A7XE | 66 | MALE | BLACK | T4 | N2 | M0 | III | NA | NA | Yes | NA | No | NA | 0 | 13 | 0 | 13 | NA | NA | 0 | 13 | G2 |
| TCGA-O2-A52N | 78 | MALE | WHITE | T2 | N0 | NA | I | NA | NA | Yes | NO | No | NA | 1 | 1006 | 0 | 1006 | NA | NA | 0 | 1006 | G2 |
| TCGA-O2-A52Q | 44 | FEMALE | WHITE | T3 | N1 | NA | III | NA | Metastasis | Yes | NO | No | NA | 1 | 113 | 1 | 113 | NA | NA | 1 | 87 | G2 |
| TCGA-O2-A52S | 57 | FEMALE | WHITE | T4 | N2 | NA | III | NA | Metastasis | Yes | YES | No | NA | 1 | 387 | 1 | 387 | NA | NA | 1 | 246 | G2 |
| TCGA-O2-A52V | 75 | FEMALE | BLACK | T3 | N0 | NA | II | NA | Metastasis | Yes | YES | No | NA | 1 | 1335 | 0 | 1335 | NA | NA | 1 | 690 | G2 |
| TCGA-O2-A52W | 63 | MALE | BLACK | T2 | N0 | NA | I | NA | NA | Yes | NO | No | NA | 1 | 261 | 0 | 261 | NA | NA | 0 | 261 | G2 |
| TCGA-O2-A5IB | 71 | FEMALE | WHITE | T3 | N1 | NA | III | NA | Metastasis | Yes | NO | No | NA | 1 | 340 | 1 | 340 | NA | NA | 1 | 253 | G2 |
| TCGA-XC-AA0X | 77 | FEMALE | BLACK | T1 | N0 | M0 | I | NA | NA | Yes | NO | No | NA | 1 | 6 | 0 | 6 | NA | NA | 0 | 6 | G2 |
