# Supplementary material for: Prognostic and immunological roles of ammonia-induced cell death-related genes in non-small cell lung cancer
Source: BMC Pulm Med. 2026 Feb 21;26:138. doi: 10.1186/s12890-026-04181-7 (PMC13032429; doi:10.1186/s12890-026-04181-7)
Supplement: Supplementary file 1 — Supplementary Material 1. [file 12890_2026_4181_MOESM1_ESM.zip › Supplementary Table 2.docx]

**Table S2. Glutamine metabolism-related genes**

| Gene Symbol | Description | GC Id | Relevance score |
| --- | --- | --- | --- |
| GLUL | Glutamate-Ammonia Ligase | GC01M182378 | 133.0415649 |
| GLS | Glutaminase | GC02P190880 | 67.47396851 |
| MTR | 5-Methyltetrahydrofolate-Homocysteine Methyltransferase | GC01P236795 | 57.8577652 |
| MMACHC | Metabolism Of Cobalamin Associated C | GC01P045500 | 51.8321991 |
| ABCA3 | ATP Binding Cassette Subfamily A Member 3 | GC16M002275 | 50.56692505 |
| GFPT1 | Glutamine--Fructose-6-Phosphate Transaminase 1 | GC02M069319 | 46.6684227 |
| CYP2D6 | Cytochrome P450 Family 2 Subfamily D Member 6 (Gene/Pseudogene) | GC22M042126 | 45.15413666 |
| INS | Insulin | GC11M002159 | 44.91599274 |
| MTRR | 5-Methyltetrahydrofolate-Homocysteine Methyltransferase Reductase | GC05P007851 | 44.8885498 |
| CYP3A4 | Cytochrome P450 Family 3 Subfamily A Member 4 | GC07M106386 | 43.23743439 |
| PPARG | Peroxisome Proliferator Activated Receptor Gamma | GC03P012287 | 42.87533188 |
| MMADHC | Metabolism Of Cobalamin Associated D | GC02M149569 | 41.85834885 |
| OTC | Ornithine Transcarbamylase | GC0XP038345 | 41.10964966 |
| GLUD1 | Glutamate Dehydrogenase 1 | GC10M087050 | 41.05294037 |
| APOE | Apolipoprotein E | GC19P143132 | 40.23343277 |
| ACADM | Acyl-CoA Dehydrogenase Medium Chain | GC01P075724 | 40.17958832 |
| MTHFR | Methylenetetrahydrofolate Reductase | GC01M011785 | 36.69086838 |
| ASNS | Asparagine Synthetase (Glutamine-Hydrolyzing) | GC07M097854 | 36.60455704 |
| CBS | Cystathionine Beta-Synthase | GC21M043053 | 36.10847473 |
| HADHA | Hydroxyacyl-CoA Dehydrogenase Trifunctional Multienzyme Complex Subunit Alpha | GC02M026190 | 36.01848602 |
| APOB | Apolipoprotein B | GC02M020956 | 35.24920654 |
| LDLR | Low Density Lipoprotein Receptor | GC19P142248 | 34.43500519 |
| CYP1A2 | Cytochrome P450 Family 1 Subfamily A Member 2 | GC15P074748 | 34.37072754 |
| LPL | Lipoprotein Lipase | GC08P019901 | 34.22617722 |
| LOC126805944 | CDK7 Strongly-Dependent Group 2 Enhancer GRCh37_chr1:182354799-182355998 | GC01P182497 | 34.01922607 |
| MMAA | Metabolism Of Cobalamin Associated A | GC04P145652 | 33.63479233 |
| CPS1 | Carbamoyl-Phosphate Synthase 1 | GC02P210477 | 33.4892807 |
| ACADVL | Acyl-CoA Dehydrogenase Very Long Chain | GC17P143337 | 33.11848831 |
| CSF2RA | Colony Stimulating Factor 2 Receptor Subunit Alpha | GC0XP002791 | 33.01657867 |
| MMAB | Metabolism Of Cobalamin Associated B | GC12M109553 | 32.7816925 |
| PC | Pyruvate Carboxylase | GC11M066848 | 32.28200531 |
| GFPT2 | Glutamine-Fructose-6-Phosphate Transaminase 2 | GC05M180300 | 31.8392601 |
| CAD | Carbamoyl-Phosphate Synthetase 2, Aspartate Transcarbamylase, And Dihydroorotase | GC02P027217 | 31.40532303 |
| CPT2 | Carnitine Palmitoyltransferase 2 | GC01P053196 | 31.38608551 |
| SLC2A1 | Solute Carrier Family 2 Member 1 | GC01M042925 | 31.26261139 |
| TGM2 | Transglutaminase 2 | GC20M038127 | 31.18532181 |
| IGF1 | Insulin Like Growth Factor 1 | GC12M102395 | 30.43609619 |
| INSR | Insulin Receptor | GC19M007112 | 30.41908455 |
| GCDH | Glutaryl-CoA Dehydrogenase | GC19P012891 | 30.23653412 |
| SFTPC | Surfactant Protein C | GC08P022156 | 29.95597076 |
| CPT1A | Carnitine Palmitoyltransferase 1A | GC11M068754 | 29.7647686 |
| MMUT | Methylmalonyl-CoA Mutase | GC06M049430 | 29.70526123 |
| GCK | Glucokinase | GC07M045609 | 29.65429497 |
| PAH | Phenylalanine Hydroxylase | GC12M102836 | 29.51060104 |
| CETP | Cholesteryl Ester Transfer Protein | GC16P056961 | 29.34126282 |
| OPA3 | Outer Mitochondrial Membrane Lipid Metabolism Regulator OPA3 | GC19M045527 | 29.34054375 |
| UCP2 | Uncoupling Protein 2 | GC11M073974 | 29.17129135 |
| ADIPOQ | Adiponectin, C1Q And Collagen Domain Containing | GC03P186842 | 29.14568901 |
| ETFDH | Electron Transfer Flavoprotein Dehydrogenase | GC04P158672 | 29.12215996 |
| LEP | Leptin | GC07P128241 | 29.04101563 |
| HMGCL | 3-Hydroxy-3-Methylglutaryl-CoA Lyase | GC01M023801 | 28.98355865 |
| GLS2 | Glutaminase 2 | GC12M056470 | 28.92495346 |
| ASL | Argininosuccinate Lyase | GC07P066075 | 28.83018112 |
| ASS1 | Argininosuccinate Synthase 1 | GC09P130444 | 28.48062706 |
| SLC1A5 | Solute Carrier Family 1 Member 5 | GC19M105179 | 28.11820984 |
| SLC22A5 | Solute Carrier Family 22 Member 5 | GC05P132369 | 28.09404564 |
| GMPS | Guanine Monophosphate Synthase | GC03P155870 | 27.98342896 |
| GALT | Galactose-1-Phosphate Uridylyltransferase | GC09P061647 | 27.86751175 |
| G6PD | Glucose-6-Phosphate Dehydrogenase | GC0XM154618 | 27.57452774 |
| APOA1 | Apolipoprotein A1 | GC11M116835 | 27.5163765 |
| POLG | DNA Polymerase Gamma, Catalytic Subunit | GC15M159705 | 27.44630623 |
| ALDH5A1 | Aldehyde Dehydrogenase 5 Family Member A1 | GC06P024494 | 27.25138664 |
| ACADS | Acyl-CoA Dehydrogenase Short Chain | GC12P137479 | 27.19763756 |
| HADHB | Hydroxyacyl-CoA Dehydrogenase Trifunctional Multienzyme Complex Subunit Beta | GC02P026243 | 27.18616676 |
| G6PC1 | Glucose-6-Phosphatase Catalytic Subunit 1 | GC17P145863 | 27.04343033 |
| OAT | Ornithine Aminotransferase | GC10M124397 | 26.75937271 |
| SLC25A13 | Solute Carrier Family 25 Member 13 | GC07M096120 | 26.70244408 |
| IL6 | Interleukin 6 | GC07P022725 | 26.2990303 |
| PCCB | Propionyl-CoA Carboxylase Subunit Beta | GC03P136250 | 26.29894638 |
| ALDH2 | Aldehyde Dehydrogenase 2 Family Member | GC12P111766 | 26.10132599 |
| LIPC | Lipase C, Hepatic Type | GC15P058410 | 26.04963112 |
| GGT1 | Gamma-Glutamyltransferase 1 | GC22P024583 | 26.00296593 |
| CYP1B1 | Cytochrome P450 Family 1 Subfamily B Member 1 | GC02M038066 | 25.812397 |
| DHFR | Dihydrofolate Reductase | GC05M080626 | 25.79200745 |
| ETFA | Electron Transfer Flavoprotein Subunit Alpha | GC15M159336 | 25.67604446 |
| AKT1 | AKT Serine/Threonine Kinase 1 | GC14M104769 | 25.63826752 |
| ADA | Adenosine Deaminase | GC20M044620 | 25.6092186 |
| CYP2E1 | Cytochrome P450 Family 2 Subfamily E Member 1 | GC10P133520 | 25.55585861 |
| CYP2A6 | Cytochrome P450 Family 2 Subfamily A Member 6 | GC19M040843 | 25.47603035 |
| SLC17A5 | Solute Carrier Family 17 Member 5 | GC06M073593 | 25.47360992 |
| NAGS | N-Acetylglutamate Synthase | GC17P044004 | 25.47084045 |
| HNF4A | Hepatocyte Nuclear Factor 4 Alpha | GC20P044355 | 25.45920372 |
| LPIN1 | Lipin 1 | GC02P011677 | 25.2291832 |
| TPMT | Thiopurine S-Methyltransferase | GC06M018128 | 25.12112045 |
| CYP1A1 | Cytochrome P450 Family 1 Subfamily A Member 1 | GC15M074719 | 25.10283852 |
| ALDOB | Aldolase, Fructose-Bisphosphate B | GC09M101420 | 25.07484245 |
| ETFB | Electron Transfer Flavoprotein Subunit Beta | GC19M051345 | 24.92923737 |
| ARG1 | Arginase 1 | GC06P174283 | 24.8999176 |
| HSD17B10 | Hydroxysteroid 17-Beta Dehydrogenase 10 | GC0XM053431 | 24.87879944 |
| AR | Androgen Receptor | GC0XP067544 | 24.84384155 |
| ACADSB | Acyl-CoA Dehydrogenase Short/Branched Chain | GC10P123008 | 24.81898308 |
| CP | Ceruloplasmin | GC03M149162 | 24.56560898 |
| HPRT1 | Hypoxanthine Phosphoribosyltransferase 1 | GC0XP134460 | 24.51510429 |
| ECHS1 | Enoyl-CoA Hydratase, Short Chain 1 | GC10M133362 | 24.45316505 |
| ACAT1 | Acetyl-CoA Acetyltransferase 1 | GC11P108121 | 24.42531586 |
| SLC25A20 | Solute Carrier Family 25 Member 20 | GC03M048969 | 24.4195385 |
| DPYD | Dihydropyrimidine Dehydrogenase | GC01M097015 | 24.39544106 |
| FLAD1 | Flavin Adenine Dinucleotide Synthetase 1 | GC01P154983 | 24.2844162 |
| HSD11B1 | Hydroxysteroid 11-Beta Dehydrogenase 1 | GC01P209686 | 24.2605648 |
| LMBRD1 | LMBR1 Domain Containing 1 | GC06M103806 | 24.15536308 |
| CFTR | CF Transmembrane Conductance Regulator | GC07P117287 | 24.12023544 |
| XDH | Xanthine Dehydrogenase | GC02M031334 | 24.10588837 |
| CRP | C-Reactive Protein | GC01M166465 | 24.05121613 |
| QDPR | Quinoid Dihydropteridine Reductase | GC04M017460 | 24.01028824 |
| HADH | Hydroxyacyl-CoA Dehydrogenase | GC04P107989 | 23.91245651 |
| ACACA | Acetyl-CoA Carboxylase Alpha | GC17M037084 | 23.90981102 |
| DDC | Dopa Decarboxylase | GC07M050458 | 23.88750839 |
| ADSL | Adenylosuccinate Lyase | GC22P040346 | 23.81202316 |
| HFE | Homeostatic Iron Regulator | GC06P026087 | 23.77478027 |
| ALB | Albumin | GC04P073397 | 23.68918228 |
| ACAD8 | Acyl-CoA Dehydrogenase Family Member 8 | GC11P134253 | 23.66450119 |
| ABCG5 | ATP Binding Cassette Subfamily G Member 5 | GC02M043806 | 23.65341377 |
| PCCA | Propionyl-CoA Carboxylase Subunit Alpha | GC13P100089 | 23.64493561 |
| KYAT1 | Kynurenine Aminotransferase 1 | GC09M131296 | 23.63552475 |
| PPARA | Peroxisome Proliferator Activated Receptor Alpha | GC22P046150 | 23.57177353 |
| ACAD9 | Acyl-CoA Dehydrogenase Family Member 9 | GC03P141955 | 23.54672241 |
| SGTA | Small Glutamine Rich Tetratricopeptide Repeat Co-Chaperone Alpha | GC19M002754 | 23.47813225 |
| CYP19A1 | Cytochrome P450 Family 19 Subfamily A Member 1 | GC15M051208 | 23.46461296 |
| TH | Tyrosine Hydroxylase | GC11M002163 | 23.44963074 |
| COMT | Catechol-O-Methyltransferase | GC22P019941 | 23.42580414 |
| ATP7B | ATPase Copper Transporting Beta | GC13M051930 | 23.38887405 |
| SLC7A7 | Solute Carrier Family 7 Member 7 | GC14M022773 | 23.38279533 |
| LCAT | Lecithin-Cholesterol Acyltransferase | GC16M067939 | 23.33376312 |
| NPC1 | NPC Intracellular Cholesterol Transporter 1 | GC18M023506 | 23.26256561 |
| CYP27A1 | Cytochrome P450 Family 27 Subfamily A Member 1 | GC02P218781 | 23.21388054 |
| BTD | Biotinidase | GC03P025710 | 23.16692924 |
| GPT | Glutamic--Pyruvic Transaminase | GC08P144502 | 23.16687775 |
| TANGO2 | Transport And Golgi Organization 2 Homolog | GC22P020017 | 23.15789795 |
| MLYCD | Malonyl-CoA Decarboxylase | GC16P083899 | 23.09885025 |
| GALK1 | Galactokinase 1 | GC17M075751 | 22.95829391 |
| CYP17A1 | Cytochrome P450 Family 17 Subfamily A Member 1 | GC10M102830 | 22.91939545 |
| BDNF-AS | BDNF Antisense RNA | GC11P027466 | 22.90629768 |
| LGSN | Lengsin, Lens Protein With Glutamine Synthetase Domain | GC06M063275 | 22.86301422 |
| TNF | Tumor Necrosis Factor | GC06P173080 | 22.80966187 |
| ABCG8 | ATP Binding Cassette Subfamily G Member 8 | GC02P044543 | 22.79985237 |
| TAFAZZIN | Tafazzin, Phospholipid-Lysophospholipid Transacylase | GC0XP155056 | 22.7860775 |
| QRSL1 | Glutaminyl-TRNA Amidotransferase Subunit QRSL1 | GC06P106629 | 22.65826416 |
| IVD | Isovaleryl-CoA Dehydrogenase | GC15P040405 | 22.5483551 |
| VDR | Vitamin D Receptor | GC12M047841 | 22.51963806 |
| GAA | Alpha Glucosidase | GC17P080101 | 22.49263573 |
| NR3C1 | Nuclear Receptor Subfamily 3 Group C Member 1 | GC05M143277 | 22.48007202 |
| LEPR | Leptin Receptor | GC01P070891 | 22.47677994 |
| CYP27B1 | Cytochrome P450 Family 27 Subfamily B Member 1 | GC12M060216 | 22.46755219 |
| ABCA1 | ATP Binding Cassette Subfamily A Member 1 | GC09M104781 | 22.42837143 |
| GOT2 | Glutamic-Oxaloacetic Transaminase 2 | GC16M058707 | 22.39387512 |
| TP53 | Tumor Protein P53 | GC17M007661 | 22.27466202 |
| UGT1A1 | UDP Glucuronosyltransferase Family 1 Member A1 | GC02P233760 | 22.18102455 |
| PON1 | Paraoxonase 1 | GC07M095297 | 22.17742157 |
| BCKDHB | Branched Chain Keto Acid Dehydrogenase E1 Subunit Beta | GC06P080106 | 22.14316177 |
| FGF23 | Fibroblast Growth Factor 23 | GC12M004368 | 22.14302063 |
| SOD1 | Superoxide Dismutase 1 | GC21P031659 | 22.11113739 |
| SI | Sucrase-Isomaltase | GC03M164978 | 22.06298828 |
| SFPQ | Splicing Factor Proline And Glutamine Rich | GC01M035176 | 22.04893684 |
| PDP1 | Pyruvate Dehydrogenase Phosphatase Catalytic Subunit 1 | GC08P093857 | 21.96642113 |
| PCSK9 | Proprotein Convertase Subtilisin/Kexin Type 9 | GC01P055039 | 21.89010048 |
| HNF1A | HNF1 Homeobox A | GC12P120978 | 21.87823677 |
| DLD | Dihydrolipoamide Dehydrogenase | GC07P109995 | 21.86719513 |
| MTOR | Mechanistic Target Of Rapamycin Kinase | GC01M011106 | 21.82019997 |
| TAT | Tyrosine Aminotransferase | GC16M071565 | 21.78702164 |
| NADSYN1 | NAD Synthetase 1 | GC11P103605 | 21.76685333 |
| SLC38A3 | Solute Carrier Family 38 Member 3 | GC03P050205 | 21.75046921 |
| QARS1 | Glutaminyl-TRNA Synthetase 1 | GC03M054494 | 21.72714615 |
| GBA1 | Glucosylceramidase Beta 1 | GC01M166343 | 21.66935921 |
| ALDH18A1 | Aldehyde Dehydrogenase 18 Family Member A1 | GC10M095605 | 21.64329529 |
| PPARGC1A | PPARG Coactivator 1 Alpha | GC04M023755 | 21.58520889 |
| APP | Amyloid Beta Precursor Protein | GC21M025880 | 21.58506584 |
| EPHX2 | Epoxide Hydrolase 2 | GC08P027490 | 21.55528069 |
| PCK2 | Phosphoenolpyruvate Carboxykinase 2, Mitochondrial | GC14P024094 | 21.54626465 |
| HMGCS2 | 3-Hydroxy-3-Methylglutaryl-CoA Synthase 2 | GC01M119747 | 21.54093742 |
| LDHA | Lactate Dehydrogenase A | GC11P018394 | 21.5357666 |
| FH | Fumarate Hydratase | GC01M241499 | 21.46308899 |
| HBB | Hemoglobin Subunit Beta | GC11M014141 | 21.34690094 |
| SLC25A4 | Solute Carrier Family 25 Member 4 | GC04P185143 | 21.32532883 |
| GAMT | Guanidinoacetate N-Methyltransferase | GC19M001397 | 21.31381226 |
| DHCR7 | 7-Dehydrocholesterol Reductase | GC11M136756 | 21.25915909 |
| PYGM | Glycogen Phosphorylase, Muscle Associated | GC11M064746 | 21.10837364 |
| CASR | Calcium Sensing Receptor | GC03P122183 | 21.05082703 |
| SUOX | Sulfite Oxidase | GC12P055997 | 21.03121185 |
| PTEN | Phosphatase And Tensin Homolog | GC10P120068 | 20.99691963 |
| KCNJ11 | Potassium Inwardly Rectifying Channel Subfamily J Member 11 | GC11M018064 | 20.9567852 |
| HLCS | Holocarboxylase Synthetase | GC21M036750 | 20.93933868 |
| PNPO | Pyridoxamine 5'-Phosphate Oxidase | GC17P047941 | 20.89313507 |
| APOA5 | Apolipoprotein A5 | GC11M116789 | 20.86918068 |
| EGF | Epidermal Growth Factor | GC04P109912 | 20.86066055 |
| NTAQ1 | N-Terminal Glutamine Amidase 1 | GC08P123913 | 20.82694626 |
| SMPD1 | Sphingomyelin Phosphodiesterase 1 | GC11P006390 | 20.765028 |
| ACE | Angiotensin I Converting Enzyme | GC17P063477 | 20.74549294 |
| CYP11A1 | Cytochrome P450 Family 11 Subfamily A Member 1 | GC15M074337 | 20.68933868 |
| LMNA | Lamin A/C | GC01P156082 | 20.67454529 |
| H6PD | Hexose-6-Phosphate Dehydrogenase/Glucose 1-Dehydrogenase | GC01P009234 | 20.6286869 |
| TTR | Transthyretin | GC18P031557 | 20.58160019 |
| FAH | Fumarylacetoacetate Hydrolase | GC15P080152 | 20.57499695 |
| AGXT | Alanine--Glyoxylate Aminotransferase | GC02P240868 | 20.56587029 |
| GCG | Glucagon | GC02M162142 | 20.53535843 |
| HIF1A | Hypoxia Inducible Factor 1 Subunit Alpha | GC14P061695 | 20.51301765 |
| CYP11B1 | Cytochrome P450 Family 11 Subfamily B Member 1 | GC08M142872 | 20.44431305 |
| SLC38A1 | Solute Carrier Family 38 Member 1 | GC12M046183 | 20.4195919 |
| PTS | 6-Pyruvoyltetrahydropterin Synthase | GC11P112226 | 20.40223312 |
| TGM1 | Transglutaminase 1 | GC14M024249 | 20.39488983 |
| ADK | Adenosine Kinase | GC10P075599 | 20.37301064 |
| MCCC2 | Methylcrotonyl-CoA Carboxylase Subunit 2 | GC05P081216 | 20.30431175 |
| SLC25A15 | Solute Carrier Family 25 Member 15 | GC13P040789 | 20.30291557 |
| SRD5A2 | Steroid 5 Alpha-Reductase 2 | GC02M031522 | 20.29137421 |
| RYR1 | Ryanodine Receptor 1 | GC19P142895 | 20.28730774 |
| CA5A | Carbonic Anhydrase 5A | GC16M087882 | 20.25760269 |
| EPHX1 | Epoxide Hydrolase 1 | GC01P225810 | 20.25632668 |
| SUCLG1 | Succinate-CoA Ligase GDP/ADP-Forming Subunit Alpha | GC02M084423 | 20.24814796 |
| TF | Transferrin | GC03P142020 | 20.16407967 |
| POR | Cytochrome P450 Oxidoreductase | GC07P075899 | 20.14804649 |
| FBP1 | Fructose-Bisphosphatase 1 | GC09M094603 | 19.92920876 |
| CTSD | Cathepsin D | GC11M001752 | 19.88511276 |
| ABCC8 | ATP Binding Cassette Subfamily C Member 8 | GC11M017392 | 19.86622047 |
| AMPD3 | Adenosine Monophosphate Deaminase 3 | GC11P016185 | 19.8508606 |
| AGL | Amylo-Alpha-1,6-Glucosidase And 4-Alpha-Glucanotransferase | GC01P099850 | 19.80657578 |
| PIK3CA | Phosphatidylinositol-4,5-Bisphosphate 3-Kinase Catalytic Subunit Alpha | GC03P179148 | 19.78471756 |
| PNPLA2 | Patatin Like Phospholipase Domain Containing 2 | GC11P015747 | 19.78211594 |
| SLC5A1 | Solute Carrier Family 5 Member 1 | GC22P032043 | 19.7594986 |
| SLCO1B1 | Solute Carrier Organic Anion Transporter Family Member 1B1 | GC12P068706 | 19.75306511 |
| LIPE | Lipase E, Hormone Sensitive Type | GC19M042401 | 19.72363663 |
| MDH2 | Malate Dehydrogenase 2 | GC07P076048 | 19.71294403 |
| MTHFD1 | Methylenetetrahydrofolate Dehydrogenase, Cyclohydrolase And Formyltetrahydrofolate Synthetase 1 | GC14P064388 | 19.69843674 |
| ARSA | Arylsulfatase A | GC22M050622 | 19.69776917 |
| PDHA1 | Pyruvate Dehydrogenase E1 Subunit Alpha 1 | GC0XP019343 | 19.69140244 |
| MYC | MYC Proto-Oncogene, BHLH Transcription Factor | GC08P127735 | 19.66751862 |
| PFKM | Phosphofructokinase, Muscle | GC12P048105 | 19.65953255 |
| BGLAP | Bone Gamma-Carboxyglutamate Protein | GC01P156242 | 19.64526558 |
| GAPDH | Glyceraldehyde-3-Phosphate Dehydrogenase | GC12P068260 | 19.64242554 |
| HTT | Huntingtin | GC04P003041 | 19.64128876 |
| HMOX1 | Heme Oxygenase 1 | GC22P035380 | 19.61670876 |
| APOC3 | Apolipoprotein C3 | GC11P116829 | 19.60332298 |
| SUCLA2 | Succinate-CoA Ligase ADP-Forming Subunit Beta | GC13M047745 | 19.58219528 |
| GHRL | Ghrelin And Obestatin Prepropeptide | GC03M010285 | 19.57793999 |
| ALPL | Alkaline Phosphatase, Biomineralization Associated | GC01P021508 | 19.56937408 |
| ACADL | Acyl-CoA Dehydrogenase Long Chain | GC02M210187 | 19.53763008 |
| PNP | Purine Nucleoside Phosphorylase | GC14P054161 | 19.51828766 |
| PRODH | Proline Dehydrogenase 1 | GC22M018912 | 19.5166378 |
| GATM | Glycine Amidinotransferase | GC15M045361 | 19.51517868 |
| CAT | Catalase | GC11P034460 | 19.44950867 |
| FASN | Fatty Acid Synthase | GC17M082078 | 19.43382263 |
| MTTP | Microsomal Triglyceride Transfer Protein | GC04P099563 | 19.43089104 |
| NAGLU | N-Acetyl-Alpha-Glucosaminidase | GC17P144465 | 19.40770531 |
| ENPP1 | Ectonucleotide Pyrophosphatase/Phosphodiesterase 1 | GC06P131808 | 19.33537865 |
| PCK1 | Phosphoenolpyruvate Carboxykinase 1 | GC20P057561 | 19.307827 |
| GALE | UDP-Galactose-4-Epimerase | GC01M023795 | 19.29329681 |
| MPV17 | Mitochondrial Inner Membrane Protein MPV17 | GC02M027309 | 19.28572655 |
| REN | Renin | GC01M204154 | 19.20922089 |
| AHCY | Adenosylhomocysteinase | GC20M036643 | 19.16066742 |
| SLC2A2 | Solute Carrier Family 2 Member 2 | GC03M171024 | 19.11683273 |
| F9 | Coagulation Factor IX | GC0XP139530 | 19.11206818 |
| ACY1 | Aminoacylase 1 | GC03P051983 | 19.06637383 |
| GPT2 | Glutamic--Pyruvic Transaminase 2 | GC16P113459 | 19.00457191 |
| RBP4 | Retinol Binding Protein 4 | GC10M093591 | 18.99750519 |
| HK1 | Hexokinase 1 | GC10P069269 | 18.99523926 |
| CYP21A2 | Cytochrome P450 Family 21 Subfamily A Member 2 | GC06P173102 | 18.96476746 |
| GLA | Galactosidase Alpha | GC0XM101393 | 18.89758873 |
| GSS | Glutathione Synthetase | GC20M034928 | 18.88777542 |
| MCCC1 | Methylcrotonyl-CoA Carboxylase Subunit 1 | GC03M183015 | 18.85186386 |
| GCH1 | GTP Cyclohydrolase 1 | GC14M054842 | 18.84169197 |
| AMPD1 | Adenosine Monophosphate Deaminase 1 | GC01M114673 | 18.82588768 |
| FMO3 | Flavin Containing Dimethylaniline Monoxygenase 3 | GC01P171090 | 18.82170868 |
| CTNNB1 | Catenin Beta 1 | GC03P041194 | 18.81895065 |
| PLIN1 | Perilipin 1 | GC15M089664 | 18.80947113 |
| TCN2 | Transcobalamin 2 | GC22P086685 | 18.80133438 |
| ADRB2 | Adrenoceptor Beta 2 | GC05P157205 | 18.80069733 |
| ALDH7A1 | Aldehyde Dehydrogenase 7 Family Member A1 | GC05M126541 | 18.78224182 |
| CD36 | CD36 Molecule (CD36 Blood Group) | GC07P080369 | 18.75903511 |
| OGDH | Oxoglutarate Dehydrogenase | GC07P044606 | 18.74275208 |
| GSTM1 | Glutathione S-Transferase Mu 1 | GC01P109687 | 18.67640114 |
| MOCS2 | Molybdenum Cofactor Synthesis 2 | GC05M053095 | 18.66995239 |
| BCKDHA | Branched Chain Keto Acid Dehydrogenase E1 Subunit Alpha | GC19P143009 | 18.66414642 |
| HCFC1 | Host Cell Factor C1 | GC0XM153947 | 18.65014458 |
| PPAT | Phosphoribosyl Pyrophosphate Amidotransferase | GC04M056393 | 18.62553024 |
| SDHA | Succinate Dehydrogenase Complex Flavoprotein Subunit A | GC05P000238 | 18.59492302 |
| PEPD | Peptidase D | GC19M033386 | 18.58164978 |
| POMC | Proopiomelanocortin | GC02M025160 | 18.58058739 |
| ABAT | 4-Aminobutyrate Aminotransferase | GC16P008674 | 18.55527115 |
| CYP11B2 | Cytochrome P450 Family 11 Subfamily B Member 2 | GC08M142910 | 18.48915291 |
| HPD | 4-Hydroxyphenylpyruvate Dioxygenase | GC12M121839 | 18.48314285 |
| ACSF3 | Acyl-CoA Synthetase Family Member 3 | GC16P089088 | 18.47593307 |
| STAR | Steroidogenic Acute Regulatory Protein | GC08M038792 | 18.46557808 |
| LIPA | Lipase A, Lysosomal Acid Type | GC10M089213 | 18.43756676 |
| ABCD1 | ATP Binding Cassette Subfamily D Member 1 | GC0XP153724 | 18.41179276 |
| ALDH4A1 | Aldehyde Dehydrogenase 4 Family Member A1 | GC01M018871 | 18.38610077 |
| PSAP | Prosaposin | GC10M071816 | 18.37735367 |
| SOD2-OT1 | SOD2 Overlapping Transcript 1 | GC06M159772 | 18.28451538 |
| ACOX1 | Acyl-CoA Oxidase 1 | GC17M075941 | 18.27331924 |
| OPA1 | OPA1 Mitochondrial Dynamin Like GTPase | GC03P193594 | 18.23489952 |
| PGK1 | Phosphoglycerate Kinase 1 | GC0XP078241 | 18.21278191 |
| AHR | Aryl Hydrocarbon Receptor | GC07P016916 | 18.20828629 |
| PKM | Pyruvate Kinase M1/2 | GC15M072199 | 18.18262482 |
| SLC16A1 | Solute Carrier Family 16 Member 1 | GC01M113143 | 18.15345764 |
| IDH2 | Isocitrate Dehydrogenase (NADP(+)) 2 | GC15M090083 | 18.14611435 |
| QRICH1 | Glutamine Rich 1 | GC03M054492 | 18.12508965 |
| MCEE | Methylmalonyl-CoA Epimerase | GC02M071110 | 18.11828995 |
| TKT | Transketolase | GC03M053224 | 18.10780716 |
| HSD17B4 | Hydroxysteroid 17-Beta Dehydrogenase 4 | GC05P119452 | 18.09960938 |
| HEXB | Hexosaminidase Subunit Beta | GC05P074640 | 18.08813477 |
| TYMP | Thymidine Phosphorylase | GC22M050525 | 18.08417892 |
| TFAM | Transcription Factor A, Mitochondrial | GC10P058385 | 18.06835175 |
| ATP5F1A | ATP Synthase F1 Subunit Alpha | GC18M046081 | 18.06552505 |
| LIPT1 | Lipoyltransferase 1 | GC02P099276 | 18.05468941 |
| AKT2 | AKT Serine/Threonine Kinase 2 | GC19M040230 | 18.02804184 |
| PRKAG3 | Protein Kinase AMP-Activated Non-Catalytic Subunit Gamma 3 | GC02M218823 | 18.00855827 |
| HAMP | Hepcidin Antimicrobial Peptide | GC19P142820 | 17.98264694 |
| PPOX | Protoporphyrinogen Oxidase | GC01P173897 | 17.96339607 |
| UMPS | Uridine Monophosphate Synthetase | GC03P124730 | 17.91564178 |
| SLC46A1 | Solute Carrier Family 46 Member 1 | GC17M092927 | 17.88433647 |
| CS | Citrate Synthase | GC12M056271 | 17.85374069 |
| IDUA | Alpha-L-Iduronidase | GC04P000986 | 17.84083366 |
| GBE1 | 1,4-Alpha-Glucan Branching Enzyme 1 | GC03M081489 | 17.7784729 |
| SLC19A1 | Solute Carrier Family 19 Member 1 | GC21M045493 | 17.76539612 |
| GLDC | Glycine Decarboxylase | GC09M006522 | 17.73600769 |
| GATB | Glutamyl-TRNA Amidotransferase Subunit B | GC04M151670 | 17.72926331 |
| SGTB | Small Glutamine Rich Tetratricopeptide Repeat Co-Chaperone Beta | GC05M065665 | 17.70452881 |
| SIRT1 | Sirtuin 1 | GC10P067884 | 17.69978333 |
| SLC37A4 | Solute Carrier Family 37 Member 4 | GC11M137443 | 17.69911575 |
| SLC6A8 | Solute Carrier Family 6 Member 8 | GC0XP153774 | 17.67004204 |
| GUSB | Glucuronidase Beta | GC07M065960 | 17.57087135 |
| ASPA | Aspartoacylase | GC17P003472 | 17.5256691 |
| TYR | Tyrosinase | GC11P089177 | 17.49830246 |
| PRKAG2 | Protein Kinase AMP-Activated Non-Catalytic Subunit Gamma 2 | GC07M151556 | 17.48981857 |
| GSR | Glutathione-Disulfide Reductase | GC08M030678 | 17.47490311 |
| SLC6A19 | Solute Carrier Family 6 Member 19 | GC05P001201 | 17.47372437 |
| VCP | Valosin Containing Protein | GC09M035572 | 17.46434593 |
| NFE2L2 | NFE2 Like BZIP Transcription Factor 2 | GC02M177227 | 17.43132973 |
| CUBN | Cubilin | GC10M016824 | 17.38332748 |
| AGK | Acylglycerol Kinase | GC07P141551 | 17.37636185 |
| ABCB11 | ATP Binding Cassette Subfamily B Member 11 | GC02M168922 | 17.37401581 |
| ALAS2 | 5'-Aminolevulinate Synthase 2 | GC0XM055009 | 17.32673073 |
| PANK2 | Pantothenate Kinase 2 | GC20P010259 | 17.28809929 |
| APRT | Adenine Phosphoribosyltransferase | GC16M088810 | 17.26647758 |
| BCS1L | BCS1 Homolog, Ubiquinol-Cytochrome C Reductase Complex Chaperone | GC02P218658 | 17.2482605 |
| MAOA | Monoamine Oxidase A | GC0XP043654 | 17.23619652 |
| MT-TP | Mitochondrially Encoded TRNA-Pro (CCN) | GCMTM015957 | 17.17080307 |
| HIBCH | 3-Hydroxyisobutyryl-CoA Hydrolase | GC02M190189 | 17.1654644 |
| ABCD4 | ATP Binding Cassette Subfamily D Member 4 | GC14M074285 | 17.14815521 |
| AASS | Aminoadipate-Semialdehyde Synthase | GC07M122073 | 17.13108253 |
| PRPS1 | Phosphoribosyl Pyrophosphate Synthetase 1 | GC0XP107628 | 17.10741425 |
| HSPD1 | Heat Shock Protein Family D (Hsp60) Member 1 | GC02M197486 | 17.10207176 |
| TFR2 | Transferrin Receptor 2 | GC07M100620 | 17.10148621 |
| MOCS1 | Molybdenum Cofactor Synthesis 1 | GC06M039899 | 17.09845924 |
| GAD1 | Glutamate Decarboxylase 1 | GC02P170813 | 17.03144646 |
| GLB1 | Galactosidase Beta 1 | GC03M032963 | 16.99834824 |
| SDHB | Succinate Dehydrogenase Complex Iron Sulfur Subunit B | GC01M021192 | 16.9724865 |
| ASAH1 | N-Acylsphingosine Amidohydrolase 1 | GC08M018055 | 16.96798706 |
| SERPINA1 | Serpin Family A Member 1 | GC14M094376 | 16.96650505 |
| HRAS | HRas Proto-Oncogene, GTPase | GC11M013956 | 16.95895386 |
| CAV1 | Caveolin 1 | GC07P116524 | 16.9503212 |
| L2HGDH | L-2-Hydroxyglutarate Dehydrogenase | GC14M050237 | 16.94116211 |
| TGFB1 | Transforming Growth Factor Beta 1 | GC19M041301 | 16.93700218 |
| PRKAA1 | Protein Kinase AMP-Activated Catalytic Subunit Alpha 1 | GC05M040759 | 16.93467712 |
| AUH | AU RNA Binding Methylglutaconyl-CoA Hydratase | GC09M091213 | 16.92996407 |
| NDUFS4 | NADH:Ubiquinone Oxidoreductase Subunit S4 | GC05P053560 | 16.92063141 |
| PPARD | Peroxisome Proliferator Activated Receptor Delta | GC06P173188 | 16.89057732 |
| GM2A | Ganglioside GM2 Activator | GC05P151212 | 16.87038994 |
| PCBD1 | Pterin-4 Alpha-Carbinolamine Dehydratase 1 | GC10M070882 | 16.86841393 |
| TTPA | Alpha Tocopherol Transfer Protein | GC08M063048 | 16.84734344 |
| GALC | Galactosylceramidase | GC14M087837 | 16.84535599 |
| CYB5R3 | Cytochrome B5 Reductase 3 | GC22M083923 | 16.82510567 |
| TK2 | Thymidine Kinase 2 | GC16M066508 | 16.8147068 |
| DPYS | Dihydropyrimidinase | GC08M104331 | 16.80970573 |
| CYP2R1 | Cytochrome P450 Family 2 Subfamily R Member 1 | GC11M014877 | 16.77778244 |
| GNE | Glucosamine (UDP-N-Acetyl)-2-Epimerase/N-Acetylmannosamine Kinase | GC09M036214 | 16.74526405 |
| NR1H2 | Nuclear Receptor Subfamily 1 Group H Member 2 | GC19P050329 | 16.73924828 |
| PGM1 | Phosphoglucomutase 1 | GC01P063593 | 16.73778343 |
| AGPAT2 | 1-Acylglycerol-3-Phosphate O-Acyltransferase 2 | GC09M136673 | 16.73317337 |
| ALDOA | Aldolase, Fructose-Bisphosphate A | GC16P030064 | 16.73203468 |
| PRKAA2 | Protein Kinase AMP-Activated Catalytic Subunit Alpha 2 | GC01P056645 | 16.72472572 |
| DNM1L | Dynamin 1 Like | GC12P032679 | 16.71701622 |
| AMACR | Alpha-Methylacyl-CoA Racemase | GC05M033986 | 16.71162224 |
| HJV | Hemojuvelin BMP Co-Receptor | GC01M165818 | 16.70772552 |
| HSD3B2 | Hydroxy-Delta-5-Steroid Dehydrogenase, 3 Beta- And Steroid Delta-Isomerase 2 | GC01P119414 | 16.68609047 |
| APOA2 | Apolipoprotein A2 | GC01M161222 | 16.67035103 |
| MIR122 | MicroRNA 122 | GC18P058451 | 16.60645676 |
| COX5A | Cytochrome C Oxidase Subunit 5A | GC15M074919 | 16.60494423 |
| PRKAB1 | Protein Kinase AMP-Activated Non-Catalytic Subunit Beta 1 | GC12P119667 | 16.60268974 |
| DGUOK | Deoxyguanosine Kinase | GC02P073926 | 16.59827232 |
| GSTP1 | Glutathione S-Transferase Pi 1 | GC11P067583 | 16.58107948 |
| RRM2B | Ribonucleotide Reductase Regulatory TP53 Inducible Subunit M2B | GC08M102204 | 16.5655365 |
| NPC2 | NPC Intracellular Cholesterol Transporter 2 | GC14M074476 | 16.53853607 |
| HEXA | Hexosaminidase Subunit Alpha | GC15M072340 | 16.53436661 |
| CES1 | Carboxylesterase 1 | GC16M055836 | 16.52998161 |
| PHGDH | Phosphoglycerate Dehydrogenase | GC01P119692 | 16.50519943 |
| MFN2 | Mitofusin 2 | GC01P011980 | 16.49911118 |
| UCP3 | Uncoupling Protein 3 | GC11M074000 | 16.48086548 |
| GNAS | GNAS Complex Locus | GC20P058839 | 16.45789528 |
| APOC2 | Apolipoprotein C2 | GC19P143136 | 16.41922951 |
| CACNA1A | Calcium Voltage-Gated Channel Subunit Alpha1 A | GC19M013206 | 16.39812088 |
| STK11 | Serine/Threonine Kinase 11 | GC19P001177 | 16.39467812 |
| ESR1 | Estrogen Receptor 1 | GC06P151656 | 16.38023949 |
| NPY | Neuropeptide Y | GC07P024647 | 16.37617683 |
| SREBF1 | Sterol Regulatory Element Binding Transcription Factor 1 | GC17M017810 | 16.35624504 |
| DMD | Dystrophin | GC0XM031097 | 16.34635162 |
| APOA4 | Apolipoprotein A4 | GC11M116820 | 16.29575157 |
| LRPPRC | Leucine Rich Pentatricopeptide Repeat Containing | GC02M043886 | 16.29436684 |
| GFAP | Glial Fibrillary Acidic Protein | GC17M093547 | 16.2666378 |
| PMM2 | Phosphomannomutase 2 | GC16P008788 | 16.25964355 |
| ATP7A | ATPase Copper Transporting Alpha | GC0XP078240 | 16.24713898 |
| UGT1A6 | UDP Glucuronosyltransferase Family 1 Member A6 | GC02P233691 | 16.21947479 |
| IRS1 | Insulin Receptor Substrate 1 | GC02M226731 | 16.20698166 |
| ATM | ATM Serine/Threonine Kinase | GC11P108223 | 16.20644188 |
| TMEM70 | Transmembrane Protein 70 | GC08P073972 | 16.19172478 |
| GH1 | Growth Hormone 1 | GC17M063917 | 16.17326355 |
| CTH | Cystathionine Gamma-Lyase | GC01P070411 | 16.15917587 |
| PRKN | Parkin RBR E3 Ubiquitin Protein Ligase | GC06M161348 | 16.12716293 |
| BSCL2 | BSCL2 Lipid Droplet Biogenesis Associated, Seipin | GC11M136455 | 16.11197472 |
| SLC40A1 | Solute Carrier Family 40 Member 1 | GC02M189560 | 16.07135963 |
| GALNS | Galactosamine (N-Acetyl)-6-Sulfatase | GC16M088813 | 16.06380844 |
| PYCR1 | Pyrroline-5-Carboxylate Reductase 1 | GC17M081932 | 16.04586411 |
| GOT1 | Glutamic-Oxaloacetic Transaminase 1 | GC10M099396 | 16.02178574 |
| PTH | Parathyroid Hormone | GC11M013492 | 16.00484085 |
| GSTT1 | Glutathione S-Transferase Theta 1 | GC22Mi00270 | 15.99896431 |
| GYS1 | Glycogen Synthase 1 | GC19M105253 | 15.99036598 |
| SOD2 | Superoxide Dismutase 2 | GC06M159669 | 15.97086716 |
| TGM3 | Transglutaminase 3 | GC20P002296 | 15.93732643 |
| SLC1A2 | Solute Carrier Family 1 Member 2 | GC11M035281 | 15.92210293 |
| PLA2G6 | Phospholipase A2 Group VI | GC22M085492 | 15.91388607 |
| NQO1 | NAD(P)H Quinone Dehydrogenase 1 | GC16M069706 | 15.89709663 |
| PDHX | Pyruvate Dehydrogenase Complex Component X | GC11P034894 | 15.87425423 |
| SLC12A3 | Solute Carrier Family 12 Member 3 | GC16P056865 | 15.87391853 |
| SLC7A9 | Solute Carrier Family 7 Member 9 | GC19M032830 | 15.85131264 |
| IGF1R | Insulin Like Growth Factor 1 Receptor | GC15P098648 | 15.83020306 |
| FTO | FTO Alpha-Ketoglutarate Dependent Dioxygenase | GC16P113652 | 15.80249786 |
| HOGA1 | 4-Hydroxy-2-Oxoglutarate Aldolase 1 | GC10P120254 | 15.75343132 |
| TFRC | Transferrin Receptor | GC03M196646 | 15.74420166 |
| GNMT | Glycine N-Methyltransferase | GC06P042960 | 15.72995281 |
| GCLC | Glutamate-Cysteine Ligase Catalytic Subunit | GC06M053497 | 15.71915245 |
| F13A1 | Coagulation Factor XIII A Chain | GC06M006144 | 15.70565796 |
| LAMP2 | Lysosomal Associated Membrane Protein 2 | GC0XM120426 | 15.7018404 |
| IDH1 | Isocitrate Dehydrogenase (NADP(+)) 1 | GC02M208236 | 15.68078709 |
| TPI1 | Triosephosphate Isomerase 1 | GC12P006867 | 15.6678896 |
| MAPT | Microtubule Associated Protein Tau | GC17P045894 | 15.64515686 |
| BCHE | Butyrylcholinesterase | GC03M165772 | 15.64099884 |
| DBT | Dihydrolipoamide Branched Chain Transacylase E2 | GC01M100186 | 15.63937092 |
| CTPS1 | CTP Synthase 1 | GC01P040979 | 15.63312531 |
| HSP90AA1 | Heat Shock Protein 90 Alpha Family Class A Member 1 | GC14M102080 | 15.62770081 |
| NR3C2 | Nuclear Receptor Subfamily 3 Group C Member 2 | GC04M148078 | 15.61531258 |
| HMBS | Hydroxymethylbilane Synthase | GC11P119084 | 15.61501217 |
| IL10 | Interleukin 10 | GC01M206767 | 15.60362816 |
| MPO | Myeloperoxidase | GC17M058269 | 15.59631729 |
| IDS | Iduronate 2-Sulfatase | GC0XM149476 | 15.58253479 |
| ATXN3 | Ataxin 3 | GC14M123129 | 15.55195904 |
| GYG1 | Glycogenin 1 | GC03P148991 | 15.54134369 |
| GATC | Glutamyl-TRNA Amidotransferase Subunit C | GC12P120446 | 15.51094437 |
| PYGL | Glycogen Phosphorylase L | GC14M050857 | 15.50233746 |
| PTPN11 | Protein Tyrosine Phosphatase Non-Receptor Type 11 | GC12P112418 | 15.49956703 |
| DNAH8 | Dynein Axonemal Heavy Chain 8 | GC06P173224 | 15.48936653 |
| CTSA | Cathepsin A | GC20P045890 | 15.47819042 |
| PIK3R1 | Phosphoinositide-3-Kinase Regulatory Subunit 1 | GC05P068215 | 15.46767521 |
| GATD3 | Glutamine Amidotransferase Class 1 Domain Containing 3 | GC21P050333 | 15.46698666 |
| ISCU | Iron-Sulfur Cluster Assembly Enzyme | GC12P108561 | 15.45922661 |
| TRMT10A | TRNA Methyltransferase 10A | GC04M099546 | 15.42005539 |
| COQ9 | Coenzyme Q9 | GC16P057447 | 15.37403297 |
| MPI | Mannose Phosphate Isomerase | GC15P074890 | 15.36924839 |
| PTGS2 | Prostaglandin-Endoperoxide Synthase 2 | GC01M186671 | 15.35823154 |
| TALDO1 | Transaldolase 1 | GC11P015745 | 15.35083008 |
| FAS | Fas Cell Surface Death Receptor | GC10P120086 | 15.33085632 |
| SLC1A3 | Solute Carrier Family 1 Member 3 | GC05P036721 | 15.31641579 |
| CYP7A1 | Cytochrome P450 Family 7 Subfamily A Member 1 | GC08M058490 | 15.31361866 |
| AMT | Aminomethyltransferase | GC03M054511 | 15.30105686 |
| DHODH | Dihydroorotate Dehydrogenase (Quinone) | GC16P072008 | 15.28635788 |
| BDNF | Brain Derived Neurotrophic Factor | GC11M027654 | 15.27171516 |
| PDHB | Pyruvate Dehydrogenase E1 Subunit Beta | GC03M058475 | 15.25350952 |
| SGSH | N-Sulfoglucosamine Sulfohydrolase | GC17M094409 | 15.22201824 |
| FECH | Ferrochelatase | GC18M057544 | 15.21343708 |
| SLC38A2 | Solute Carrier Family 38 Member 2 | GC12M046358 | 15.19400024 |
| EP300 | E1A Binding Protein P300 | GC22P087011 | 15.16973305 |
| HSPA8 | Heat Shock Protein Family A (Hsp70) Member 8 | GC11M123057 | 15.15667725 |
| AKR1A1 | Aldo-Keto Reductase Family 1 Member A1 | GC01P045550 | 15.15547371 |
| MT-ATP6 | Mitochondrially Encoded ATP Synthase Membrane Subunit 6 | GCMTP008531 | 15.13911915 |
| COQ2 | Coenzyme Q2, Polyprenyltransferase | GC04M083261 | 15.11831284 |
| MT-ND1 | Mitochondrially Encoded NADH:Ubiquinone Oxidoreductase Core Subunit 1 | GCMTP003309 | 15.11233616 |
| HSPA4 | Heat Shock Protein Family A (Hsp70) Member 4 | GC05P133122 | 15.10570526 |
| ATXN2 | Ataxin 2 | GC12M111443 | 15.06100845 |
| GPD1 | Glycerol-3-Phosphate Dehydrogenase 1 | GC12P069535 | 15.04690361 |
| PNLIP | Pancreatic Lipase | GC10P116545 | 15.0345974 |
| DLAT | Dihydrolipoamide S-Acetyltransferase | GC11P112859 | 15.0144558 |
| OXCT1 | 3-Oxoacid CoA-Transferase 1 | GC05M041732 | 14.97385979 |
| SNCA | Synuclein Alpha | GC04M089724 | 14.9691143 |
| NOS3 | Nitric Oxide Synthase 3 | GC07P163654 | 14.96820927 |
| NR1I2 | Nuclear Receptor Subfamily 1 Group I Member 2 | GC03P119780 | 14.95454979 |
| DAO | D-Amino Acid Oxidase | GC12P108896 | 14.93966389 |
| LRAT | Lecithin Retinol Acyltransferase | GC04P154626 | 14.93613243 |
| ATIC | 5-Aminoimidazole-4-Carboxamide Ribonucleotide Formyltransferase/IMP Cyclohydrolase | GC02P215311 | 14.91714859 |
| KRAS | KRAS Proto-Oncogene, GTPase | GC12M034710 | 14.8991394 |
| MT-TL1 | Mitochondrially Encoded TRNA-Leu (UUA/G) 1 | GCMTP003232 | 14.89740181 |
| ADAR | Adenosine Deaminase RNA Specific | GC01M166236 | 14.89712334 |
| POLG2 | DNA Polymerase Gamma 2, Accessory Subunit | GC17M064477 | 14.89076805 |
| NEU1 | Neuraminidase 1 | GC06M031857 | 14.87501621 |
| NR1H4 | Nuclear Receptor Subfamily 1 Group H Member 4 | GC12P100473 | 14.87167358 |
| SLC2A4 | Solute Carrier Family 2 Member 4 | GC17P143349 | 14.86826324 |
| PDK4 | Pyruvate Dehydrogenase Kinase 4 | GC07M095583 | 14.86480808 |
| ACO2 | Aconitase 2 | GC22P087035 | 14.85613251 |
| GNPTAB | N-Acetylglucosamine-1-Phosphate Transferase Subunits Alpha And Beta | GC12M101745 | 14.83795547 |
| XRCC1 | X-Ray Repair Cross Complementing 1 | GC19M043543 | 14.83112144 |
| ODC1 | Ornithine Decarboxylase 1 | GC02M010432 | 14.82935524 |
| ATN1 | Atrophin 1 | GC12P068286 | 14.81712914 |
| GPI | Glucose-6-Phosphate Isomerase | GC19P034359 | 14.812356 |
| F2 | Coagulation Factor II, Thrombin | GC11P048666 | 14.81230545 |
| UPB1 | Beta-Ureidopropionase 1 | GC22P024494 | 14.80981541 |
| COG2 | Component Of Oligomeric Golgi Complex 2 | GC01P230642 | 14.80696392 |
| PEX5 | Peroxisomal Biogenesis Factor 5 | GC12P068301 | 14.80564976 |
| ABCB1 | ATP Binding Cassette Subfamily B Member 1 | GC07M087504 | 14.80196857 |
| EPM2A | EPM2A Glucan Phosphatase, Laforin | GC06M145382 | 14.77818489 |
| HSD17B3 | Hydroxysteroid 17-Beta Dehydrogenase 3 | GC09M119952 | 14.77707863 |
| SERAC1 | Serine Active Site Containing 1 | GC06M158109 | 14.77155304 |
| ITPA | Inosine Triphosphatase | GC20P010242 | 14.76491547 |
| EIF2AK3 | Eukaryotic Translation Initiation Factor 2 Alpha Kinase 3 | GC02M088556 | 14.75196362 |
| FTCD | Formimidoyltransferase Cyclodeaminase | GC21M054604 | 14.74279881 |
| OCRL | OCRL Inositol Polyphosphate-5-Phosphatase | GC0XP129539 | 14.73995972 |
| MIR7-3HG | MIR7-3 Host Gene | GC19P141972 | 14.73498249 |
| LOC654780 | Splicing Factor Proline/Glutamine-Rich | GC16M084193 | 14.72984219 |
| PHKA2 | Phosphorylase Kinase Regulatory Subunit Alpha 2 | GC0XM018892 | 14.7246933 |
| NDUFS1 | NADH:Ubiquinone Oxidoreductase Core Subunit S1 | GC02M206114 | 14.71605968 |
| MT-CO1 | Mitochondrially Encoded Cytochrome C Oxidase I | GCMTP005906 | 14.70777988 |
| PNPLA3 | Patatin Like Phospholipase Domain Containing 3 | GC22P043923 | 14.69652748 |
| NFU1 | NFU1 Iron-Sulfur Cluster Scaffold | GC02M069395 | 14.67958546 |
| QSER1 | Glutamine And Serine Rich 1 | GC11P032892 | 14.66253567 |
| NAT2 | N-Acetyltransferase 2 | GC08P018391 | 14.64902496 |
| CREBBP | CREB Binding Protein | GC16M046544 | 14.64069176 |
| ARSB | Arylsulfatase B | GC05M078777 | 14.63484859 |
| SIRT4 | Sirtuin 4 | GC12P120291 | 14.62068939 |
| MT-CYB | Mitochondrially Encoded Cytochrome B | GCMTP014749 | 14.61839485 |
| PHKA1 | Phosphorylase Kinase Regulatory Subunit Alpha 1 | GC0XM072578 | 14.61180496 |
| DPAGT1 | Dolichyl-Phosphate N-Acetylglucosaminephosphotransferase 1 | GC11M137457 | 14.60395145 |
| SERPINE1 | Serpin Family E Member 1 | GC07P101127 | 14.57228374 |
| PEX1 | Peroxisomal Biogenesis Factor 1 | GC07M092487 | 14.56379604 |
| UGT1A9 | UDP Glucuronosyltransferase Family 1 Member A9 | GC02P233671 | 14.55094242 |
| PKLR | Pyruvate Kinase L/R | GC01M155289 | 14.54144096 |
| ACLY | ATP Citrate Lyase | GC17M041866 | 14.52614689 |
| IL1B | Interleukin 1 Beta | GC02M112829 | 14.51982594 |
| SLC1A4 | Solute Carrier Family 1 Member 4 | GC02P064988 | 14.49840736 |
| COQ8A | Coenzyme Q8A | GC01P226942 | 14.46128178 |
| RAF1 | Raf-1 Proto-Oncogene, Serine/Threonine Kinase | GC03M012583 | 14.45078754 |
| LBR | Lamin B Receptor | GC01M225401 | 14.40380001 |
| SP1 | Sp1 Transcription Factor | GC12P053380 | 14.40061378 |
| SUMF1 | Sulfatase Modifying Factor 1 | GC03M003700 | 14.3818903 |
| PFAS | Phosphoribosylformylglycinamidine Synthase | GC17P008247 | 14.36368942 |
| FXN | Frataxin | GC09P069035 | 14.34944916 |
| QRICH2 | Glutamine Rich 2 | GC17M076274 | 14.33277702 |
| PPIG | Peptidylprolyl Isomerase G | GC02P169584 | 14.30800533 |
| CREB1 | CAMP Responsive Element Binding Protein 1 | GC02P207529 | 14.30618477 |
| DBH | Dopamine Beta-Hydroxylase | GC09P133636 | 14.29290581 |
| GPHN | Gephyrin | GC14P066507 | 14.28629494 |
| BCAT2 | Branched Chain Amino Acid Transaminase 2 | GC19M048795 | 14.28439617 |
| FUCA1 | Alpha-L-Fucosidase 1 | GC01M023845 | 14.26974678 |
| PPT1 | Palmitoyl-Protein Thioesterase 1 | GC01M040249 | 14.2632494 |
| OPLAH | 5-Oxoprolinase, ATP-Hydrolysing | GC08M147183 | 14.22975731 |
| HGSNAT | Heparan-Alpha-Glucosaminide N-Acetyltransferase | GC08P043140 | 14.21787643 |
| FOLR1 | Folate Receptor Alpha | GC11P103661 | 14.21000957 |
| SLC6A3 | Solute Carrier Family 6 Member 3 | GC05M001392 | 14.18315125 |
| MB | Myoglobin | GC22M035606 | 14.17474174 |
| FBXL4 | F-Box And Leucine Rich Repeat Protein 4 | GC06M098868 | 14.16959667 |
| NSDHL | NAD(P) Dependent Steroid Dehydrogenase-Like | GC0XP152830 | 14.15214252 |
| ARG2 | Arginase 2 | GC14P067619 | 14.12821198 |
| UQCRC2 | Ubiquinol-Cytochrome C Reductase Core Protein 2 | GC16P112805 | 14.12075996 |
| AKR1B1 | Aldo-Keto Reductase Family 1 Member B | GC07M134442 | 14.09860325 |
| CD320 | CD320 Molecule | GC19M008302 | 14.09231853 |
| STS | Steroid Sulfatase | GC0XP007146 | 14.0853653 |
| IGFBP3 | Insulin Like Growth Factor Binding Protein 3 | GC07M045912 | 14.0759964 |
| CKB | Creatine Kinase B | GC14M103519 | 14.05749226 |
| SLC25A1 | Solute Carrier Family 25 Member 1 | GC22M083332 | 14.05409622 |
| AK2 | Adenylate Kinase 2 | GC01M033007 | 14.05240536 |
| GATD1 | Glutamine Amidotransferase Class 1 Domain Containing 1 | GC11M000767 | 14.05073738 |
| CPOX | Coproporphyrinogen Oxidase | GC03M098576 | 14.04942799 |
| TNFRSF11B | TNF Receptor Superfamily Member 11b | GC08M118923 | 14.03931808 |
| CLCNKB | Chloride Voltage-Gated Channel Kb | GC01P068466 | 14.02709198 |
| CCL2 | C-C Motif Chemokine Ligand 2 | GC17P034255 | 14.00492287 |
| GNS | Glucosamine (N-Acetyl)-6-Sulfatase | GC12M064713 | 14.00201702 |
| PIK3CG | Phosphatidylinositol-4,5-Bisphosphate 3-Kinase Catalytic Subunit Gamma | GC07P106865 | 13.98567009 |
| ABCG2 | ATP Binding Cassette Subfamily G Member 2 (JR Blood Group) | GC04M088090 | 13.96784878 |
| LIAS | Lipoic Acid Synthetase | GC04P039682 | 13.96598625 |
| PRDX1 | Peroxiredoxin 1 | GC01M045861 | 13.96443176 |
| PHKB | Phosphorylase Kinase Regulatory Subunit Beta | GC16P113489 | 13.94336033 |
| IGF2 | Insulin Like Growth Factor 2 | GC11M014033 | 13.92907715 |
| GFM1 | G Elongation Factor Mitochondrial 1 | GC03P158644 | 13.91714096 |
| MT-CO2 | Mitochondrially Encoded Cytochrome C Oxidase II | GCMTP007587 | 13.91683578 |
| TPP1 | Tripeptidyl Peptidase 1 | GC11M014225 | 13.91242504 |
| HGD | Homogentisate 1,2-Dioxygenase | GC03M120628 | 13.89665699 |
| CLPB | ClpB Family Mitochondrial Disaggregase | GC11M136787 | 13.88647842 |
| UROD | Uroporphyrinogen Decarboxylase | GC01P070108 | 13.86645794 |
| ANGPTL3 | Angiopoietin Like 3 | GC01P062597 | 13.85178947 |
| PLA2G7 | Phospholipase A2 Group VII | GC06M046704 | 13.84952545 |
| PTPN1 | Protein Tyrosine Phosphatase Non-Receptor Type 1 | GC20P050510 | 13.84834671 |
| SIRT3 | Sirtuin 3 | GC11M000215 | 13.84182739 |
| D2HGDH | D-2-Hydroxyglutarate Dehydrogenase | GC02P241734 | 13.83947659 |
| MT-RNR1 | Mitochondrially Encoded 12S RRNA | GCMTP000642 | 13.83602715 |
| ATAD3A | ATPase Family AAA Domain Containing 3A | GC01P067767 | 13.83166409 |
| MOCOS | Molybdenum Cofactor Sulfurase | GC18P036187 | 13.82790089 |
| COQ4 | Coenzyme Q4 | GC09P128322 | 13.82016468 |
| CTPS2 | CTP Synthase 2 | GC0XM016587 | 13.81685257 |
| NDUFS3 | NADH:Ubiquinone Oxidoreductase Core Subunit S3 | GC11P048705 | 13.81122875 |
| GLYATL1 | Glycine-N-Acyltransferase Like 1 | GC11P059335 | 13.7926712 |
| PHKG2 | Phosphorylase Kinase Catalytic Subunit Gamma 2 | GC16P113182 | 13.78648186 |
| TBP | TATA-Box Binding Protein | GC06P170554 | 13.78192711 |
| TSHR | Thyroid Stimulating Hormone Receptor | GC14P080954 | 13.77513313 |
| SCN5A | Sodium Voltage-Gated Channel Alpha Subunit 5 | GC03M038812 | 13.74676132 |
| PGAM2 | Phosphoglycerate Mutase 2 | GC07M044062 | 13.70983601 |
| SLC25A12 | Solute Carrier Family 25 Member 12 | GC02M171783 | 13.6703186 |
| VWF | Von Willebrand Factor | GC12M034378 | 13.65810966 |
| COX10 | Cytochrome C Oxidase Assembly Factor Heme A:Farnesyltransferase COX10 | GC17P014069 | 13.65043736 |
| ABCB4 | ATP Binding Cassette Subfamily B Member 4 | GC07M087365 | 13.64583969 |
| UGT1A8 | UDP Glucuronosyltransferase Family 1 Member A8 | GC02P234639 | 13.63882923 |
| HSPB1 | Heat Shock Protein Family B (Small) Member 1 | GC07P076302 | 13.63550377 |
| MANBA | Mannosidase Beta | GC04M102631 | 13.62981892 |
| MAN2B1 | Mannosidase Alpha Class 2B Member 1 | GC19M104434 | 13.61197281 |
| ATXN1 | Ataxin 1 | GC06M016299 | 13.59837341 |
| UCP1 | Uncoupling Protein 1 | GC04M140559 | 13.59739399 |
| PHYH | Phytanoyl-CoA 2-Hydroxylase | GC10M013277 | 13.5906868 |
| TWNK | Twinkle MtDNA Helicase | GC10P120366 | 13.58551884 |
| GPX1 | Glutathione Peroxidase 1 | GC03M054509 | 13.5473156 |
| WFS1 | Wolframin ER Transmembrane Glycoprotein | GC04P006269 | 13.53629398 |
| CSF2RB | Colony Stimulating Factor 2 Receptor Subunit Beta | GC22P036913 | 13.52288055 |
| NGLY1 | N-Glycanase 1 | GC03M025718 | 13.52057934 |
| ALAD | Aminolevulinate Dehydratase | GC09M113386 | 13.51011372 |
| ERCC2 | ERCC Excision Repair 2, TFIIH Core Complex Helicase Subunit | GC19M045349 | 13.49334908 |
| CTSC | Cathepsin C | GC11M137061 | 13.48190689 |
| PDK3 | Pyruvate Dehydrogenase Kinase 3 | GC0XP024465 | 13.46706486 |
| CLN3 | CLN3 Lysosomal/Endosomal Transmembrane Protein, Battenin | GC16M028466 | 13.44673061 |
| TSFM | Ts Translation Elongation Factor, Mitochondrial | GC12P057782 | 13.44645309 |
| TXN | Thioredoxin | GC09M110243 | 13.4298954 |
| RPE65 | Retinoid Isomerohydrolase RPE65 | GC01M068428 | 13.42804337 |
| EHHADH | Enoyl-CoA Hydratase And 3-Hydroxyacyl CoA Dehydrogenase | GC03M185190 | 13.4221735 |
| SLC25A22 | Solute Carrier Family 25 Member 22 | GC11M013973 | 13.41573715 |
| ABCD3 | ATP Binding Cassette Subfamily D Member 3 | GC01P094385 | 13.40934181 |
| CTNS | Cystinosin, Lysosomal Cystine Transporter | GC17P003636 | 13.37807751 |
| ALOX5 | Arachidonate 5-Lipoxygenase | GC10P045374 | 13.37061882 |
| NAGA | Alpha-N-Acetylgalactosaminidase | GC22M042058 | 13.36969852 |
| TYMS | Thymidylate Synthetase | GC18P000657 | 13.36332321 |
| FKRP | Fukutin Related Protein | GC19P143178 | 13.36197186 |
| MPC1 | Mitochondrial Pyruvate Carrier 1 | GC06M166364 | 13.36184788 |
| FABP2 | Fatty Acid Binding Protein 2 | GC04M119317 | 13.35743332 |
| NPPA | Natriuretic Peptide A | GC01M020958 | 13.35473061 |
| GYS2 | Glycogen Synthase 2 | GC12M034669 | 13.3349905 |
| SLC3A1 | Solute Carrier Family 3 Member 1 | GC02P044275 | 13.3203392 |
| SLC25A3 | Solute Carrier Family 25 Member 3 | GC12P098593 | 13.32026005 |
| TSPO | Translocator Protein | GC22P043151 | 13.31768036 |
| CAVIN1 | Caveolae Associated Protein 1 | GC17M093442 | 13.30992222 |
| CST3 | Cystatin C | GC20M024169 | 13.30826569 |
| GNPAT | Glyceronephosphate O-Acyltransferase | GC01P231241 | 13.2820797 |
| BSND | Barttin CLCNK Type Accessory Subunit Beta | GC01P054998 | 13.278615 |
| ALOX12 | Arachidonate 12-Lipoxygenase, 12S Type | GC17P143321 | 13.27715683 |
| CLN6 | CLN6 Transmembrane ER Protein | GC15M068206 | 13.27486992 |
| TUFM | Tu Translation Elongation Factor, Mitochondrial | GC16M048286 | 13.23579216 |
| MT-ND2 | Mitochondrially Encoded NADH:Ubiquinone Oxidoreductase Core Subunit 2 | GCMTP004472 | 13.2296648 |
| AGA | Aspartylglucosaminidase | GC04M177430 | 13.21972847 |
| EBP | EBP Cholestenol Delta-Isomerase | GC0XP048521 | 13.19826984 |
| DNMT1 | DNA Methyltransferase 1 | GC19M010133 | 13.1879034 |
| TCF7L2 | Transcription Factor 7 Like 2 | GC10P112950 | 13.17388153 |
| CERNA3 | Competing Endogenous LncRNA 3 For MiR-645 | GC08P056411 | 13.1144619 |
| MCOLN1 | Mucolipin TRP Cation Channel 1 | GC19P142070 | 13.11416054 |
| PIGA | Phosphatidylinositol Glycan Anchor Biosynthesis Class A | GC0XM015319 | 13.10779667 |
| G6PC3 | Glucose-6-Phosphatase Catalytic Subunit 3 | GC17P044070 | 13.0974369 |
| GCLM | Glutamate-Cysteine Ligase Modifier Subunit | GC01M093885 | 13.062994 |
| TSC2 | TSC Complex Subunit 2 | GC16P112285 | 13.06258774 |
| MECP2 | Methyl-CpG Binding Protein 2 | GC0XM154021 | 13.05792046 |
| HK2 | Hexokinase 2 | GC02P075366 | 13.03720951 |
| PDSS2 | Decaprenyl Diphosphate Synthase Subunit 2 | GC06M107152 | 13.02834034 |
| ADRB3 | Adrenoceptor Beta 3 | GC08M037962 | 13.0241251 |
| CNR1 | Cannabinoid Receptor 1 | GC06M088139 | 13.02087307 |
| MT-ND4 | Mitochondrially Encoded NADH:Ubiquinone Oxidoreductase Core Subunit 4 | GCMTP010762 | 12.98029423 |
| PSPH | Phosphoserine Phosphatase | GC07M079457 | 12.97346878 |
| HYAL1 | Hyaluronidase 1 | GC03M050299 | 12.9728384 |
| PEX7 | Peroxisomal Biogenesis Factor 7 | GC06P136822 | 12.96350765 |
| UGT1A7 | UDP Glucuronosyltransferase Family 1 Member A7 | GC02P233681 | 12.95150185 |
| GCSH | Glycine Cleavage System Protein H | GC16M081081 | 12.92965031 |
| MIR132 | MicroRNA 132 | GC17M002049 | 12.92381096 |
| PSAT1 | Phosphoserine Aminotransferase 1 | GC09P078297 | 12.91954231 |
| PDSS1 | Decaprenyl Diphosphate Synthase Subunit 1 | GC10P026697 | 12.91294479 |
| MC4R | Melanocortin 4 Receptor | GC18M060371 | 12.90785503 |
| UROS | Uroporphyrinogen III Synthase | GC10M125784 | 12.89621449 |
| MTRFR | Mitochondrial Translation Release Factor In Rescue | GC12P137518 | 12.88881874 |
| FAM3B | FAM3 Metabolism Regulating Signaling Molecule B | GC21P041304 | 12.88794708 |
| STAT3 | Signal Transducer And Activator Of Transcription 3 | GC17M042313 | 12.87752533 |
| COX4I1 | Cytochrome C Oxidase Subunit 4I1 | GC16P085798 | 12.86610889 |
| UGT1A10 | UDP Glucuronosyltransferase Family 1 Member A10 | GC02P233636 | 12.8623991 |
| SLC7A5 | Solute Carrier Family 7 Member 5 | GC16M087830 | 12.83634758 |
| LCT | Lactase | GC02M135787 | 12.82924843 |
| SLC12A1 | Solute Carrier Family 12 Member 1 | GC15P184282 | 12.82480717 |
| MT-TQ | Mitochondrially Encoded TRNA-Gln (CAA/G) | GCMTM004331 | 12.7746315 |
| ALG2 | ALG2 Alpha-1,3/1,6-Mannosyltransferase | GC09M099216 | 12.764431 |
| NHLRC1 | NHL Repeat Containing E3 Ubiquitin Protein Ligase 1 | GC06M018120 | 12.73751163 |
| ATP13A2 | ATPase Cation Transporting 13A2 | GC01M016985 | 12.72915268 |
| SRD5A1 | Steroid 5 Alpha-Reductase 1 | GC05P006633 | 12.70484829 |
| FOXRED1 | FAD Dependent Oxidoreductase Domain Containing 1 | GC11P126269 | 12.70427513 |
| RORA | RAR Related Orphan Receptor A | GC15M060488 | 12.6974802 |
| MED15 | Mediator Complex Subunit 15 | GC22P086331 | 12.69250393 |
| HSPA5 | Heat Shock Protein Family A (Hsp70) Member 5 | GC09M125234 | 12.69063568 |
| BOLA3 | BolA Family Member 3 | GC02M074136 | 12.69008732 |
| MIR21 | MicroRNA 21 | GC17P059841 | 12.68189907 |
| UQCRFS1 | Ubiquinol-Cytochrome C Reductase, Rieske Iron-Sulfur Polypeptide 1 | GC19M029205 | 12.67806053 |
| SQSTM1 | Sequestosome 1 | GC05P179806 | 12.66576958 |
| ITGAM | Integrin Subunit Alpha M | GC16P113208 | 12.66528606 |
| CLN5 | CLN5 Intracellular Trafficking Protein | GC13P076990 | 12.65024662 |
| PSEN1 | Presenilin 1 | GC14P073136 | 12.64830875 |
| IFNG | Interferon Gamma | GC12M068154 | 12.64067268 |
| CLN8 | CLN8 Transmembrane ER And ERGIC Protein | GC08P001755 | 12.63858128 |
| SLC30A10 | Solute Carrier Family 30 Member 10 | GC01M219685 | 12.62404251 |
| CCK | Cholecystokinin | GC03M042274 | 12.62237549 |
| ERN1 | Endoplasmic Reticulum To Nucleus Signaling 1 | GC17M064039 | 12.60813332 |
| AIFM1 | Apoptosis Inducing Factor Mitochondria Associated 1 | GC0XM130129 | 12.60359001 |
| ACTB | Actin Beta | GC07M005790 | 12.59575272 |
| PEX2 | Peroxisomal Biogenesis Factor 2 | GC08M076980 | 12.58831501 |
| FDXR | Ferredoxin Reductase | GC17M074862 | 12.5736084 |
| ADCY5 | Adenylate Cyclase 5 | GC03M123282 | 12.57247448 |
| MT-ATP8 | Mitochondrially Encoded ATP Synthase Membrane Subunit 8 | GCMTP008368 | 12.55530834 |
| BMP6 | Bone Morphogenetic Protein 6 | GC06P007726 | 12.54849625 |
| MYH7 | Myosin Heavy Chain 7 | GC14M023412 | 12.54461575 |
| CEL | Carboxyl Ester Lipase | GC09P133061 | 12.53616142 |
| ENO3 | Enolase 3 | GC17P004948 | 12.52120209 |
| RLBP1 | Retinaldehyde Binding Protein 1 | GC15M089209 | 12.51600838 |
| SDHD | Succinate Dehydrogenase Complex Subunit D | GC11P112862 | 12.51434135 |
| MIR22 | MicroRNA 22 | GC17M001713 | 12.51418209 |
| BRAF | B-Raf Proto-Oncogene, Serine/Threonine Kinase | GC07M140788 | 12.51153755 |
| LRP1 | LDL Receptor Related Protein 1 | GC12P057128 | 12.50920296 |
| MAPK10 | Mitogen-Activated Protein Kinase 10 | GC04M085990 | 12.50396729 |
| SLC1A1 | Solute Carrier Family 1 Member 1 | GC09P004490 | 12.46531296 |
| ZMPSTE24 | Zinc Metallopeptidase STE24 | GC01P040258 | 12.45055485 |
| ALG1 | ALG1 Chitobiosyldiphosphodolichol Beta-Mannosyltransferase | GC16P005033 | 12.44358921 |
| PEX10 | Peroxisomal Biogenesis Factor 10 | GC01M002403 | 12.44139099 |
| PEX12 | Peroxisomal Biogenesis Factor 12 | GC17M035574 | 12.43019962 |
| ATP6V0A2 | ATPase H+ Transporting V0 Subunit A2 | GC12P123712 | 12.42525673 |
| FTL | Ferritin Light Chain | GC19P048965 | 12.42347908 |
| DPM1 | Dolichyl-Phosphate Mannosyltransferase Subunit 1, Catalytic | GC20M050934 | 12.41867924 |
| COQ6 | Coenzyme Q6, Monooxygenase | GC14P073949 | 12.41574764 |
| DOLK | Dolichol Kinase | GC09M128945 | 12.38204002 |
| AGPS | Alkylglycerone Phosphate Synthase | GC02P177392 | 12.38133717 |
| FN1 | Fibronectin 1 | GC02M215360 | 12.3755312 |
| SRD5A3 | Steroid 5 Alpha-Reductase 3 | GC04P055346 | 12.37516594 |
| NFS1 | NFS1 Cysteine Desulfurase | GC20M035668 | 12.37061596 |
| IL18 | Interleukin 18 | GC11M112143 | 12.36921024 |
| SCARB1 | Scavenger Receptor Class B Member 1 | GC12M124776 | 12.36771774 |
| SLC19A2 | Solute Carrier Family 19 Member 2 | GC01M169463 | 12.35756779 |
| MAPK1 | Mitogen-Activated Protein Kinase 1 | GC22M021759 | 12.35515881 |
| MOGS | Mannosyl-Oligosaccharide Glucosidase | GC02M074461 | 12.34451866 |
| BRCA1 | BRCA1 DNA Repair Associated | GC17M043044 | 12.33970833 |
| PINK1 | PTEN Induced Kinase 1 | GC01P068700 | 12.32969093 |
| GCKR | Glucokinase Regulator | GC02P027496 | 12.32953835 |
| GLRX5 | Glutaredoxin 5 | GC14P095533 | 12.32454109 |
| DHDDS | Dehydrodolichyl Diphosphate Synthase Subunit | GC01P026432 | 12.31220913 |
| UGT1A4 | UDP Glucuronosyltransferase Family 1 Member A4 | GC02P233718 | 12.29725552 |
| TREX1 | Three Prime Repair Exonuclease 1 | GC03P063183 | 12.27761745 |
| EARS2 | Glutamyl-TRNA Synthetase 2, Mitochondrial | GC16M048066 | 12.27739716 |
| ATXN7 | Ataxin 7 | GC03P064545 | 12.24694252 |
| SAMHD1 | SAM And HD Domain Containing Deoxynucleoside Triphosphate Triphosphohydrolase 1 | GC20M036890 | 12.197855 |
| H19 | H19 Imprinted Maternally Expressed Transcript | GC11M001995 | 12.19695568 |
| SULT1A1 | Sulfotransferase Family 1A Member 1 | GC16M048228 | 12.1852417 |
| AOC1 | Amine Oxidase Copper Containing 1 | GC07P150824 | 12.17409611 |
| LARS1 | Leucyl-TRNA Synthetase 1 | GC05M146114 | 12.16685581 |
| ECI1 | Enoyl-CoA Delta Isomerase 1 | GC16M002239 | 12.16445255 |
| COQ7 | Coenzyme Q7, Hydroxylase | GC16P019067 | 12.14958286 |
| SUCLG2 | Succinate-CoA Ligase GDP-Forming Subunit Beta | GC03M067358 | 12.13508415 |
| NAXE | NAD(P)HX Epimerase | GC01P156591 | 12.10945702 |
| IL1A | Interleukin 1 Alpha | GC02M112773 | 12.09841156 |
| ALG6 | ALG6 Alpha-1,3-Glucosyltransferase | GC01P063367 | 12.07705975 |
| CTSK | Cathepsin K | GC01M166075 | 12.07399368 |
| BMP1 | Bone Morphogenetic Protein 1 | GC08P022165 | 12.06568146 |
| HNF1B | HNF1 Homeobox B | GC17M037686 | 12.05640793 |
| RDH12 | Retinol Dehydrogenase 12 | GC14P067701 | 12.04811859 |
| ATP6V1A | ATPase H+ Transporting V1 Subunit A | GC03P113811 | 12.04808331 |
| BMP2 | Bone Morphogenetic Protein 2 | GC20P006767 | 12.03752804 |
| COX6B1 | Cytochrome C Oxidase Subunit 6B1 | GC19P142828 | 12.03247929 |
| CA2 | Carbonic Anhydrase 2 | GC08P085463 | 12.03240681 |
| MIR34A | MicroRNA 34a | GC01M009151 | 12.03101158 |
| NR1H3 | Nuclear Receptor Subfamily 1 Group H Member 3 | GC11P047248 | 12.02497959 |
| PDX1 | Pancreatic And Duodenal Homeobox 1 | GC13P028549 | 12.02401543 |
| TGM6 | Transglutaminase 6 | GC20P002380 | 12.00145531 |
| SLC18A2 | Solute Carrier Family 18 Member A2 | GC10P117241 | 11.99970531 |
| PEX14 | Peroxisomal Biogenesis Factor 14 | GC01P010472 | 11.999156 |
| GHR | Growth Hormone Receptor | GC05P042429 | 11.99784851 |
| HSPG2 | Heparan Sulfate Proteoglycan 2 | GC01M021822 | 11.99405479 |
| CXCL8 | C-X-C Motif Chemokine Ligand 8 | GC04P073740 | 11.984869 |
| RNASEH2B | Ribonuclease H2 Subunit B | GC13P050909 | 11.97900009 |
| CBLIF | Cobalamin Binding Intrinsic Factor | GC11M059829 | 11.97219944 |
| AMN | Amnion Associated Transmembrane Protein | GC14P102922 | 11.94844151 |
| F5 | Coagulation Factor V | GC01M169511 | 11.92692757 |
| ALG8 | ALG8 Alpha-1,3-Glucosyltransferase | GC11M136954 | 11.88745403 |
| HBA1 | Hemoglobin Subunit Alpha 1 | GC16P112211 | 11.8846035 |
| RPS6KB1 | Ribosomal Protein S6 Kinase B1 | GC17P059893 | 11.87008286 |
| NDUFA9 | NADH:Ubiquinone Oxidoreductase Subunit A9 | GC12P004649 | 11.86451149 |
| STUB1 | STIP1 Homology And U-Box Containing Protein 1 | GC16P112233 | 11.83772659 |
| PEX6 | Peroxisomal Biogenesis Factor 6 | GC06M042963 | 11.83764935 |
| NDUFAF2 | NADH:Ubiquinone Oxidoreductase Complex Assembly Factor 2 | GC05P060945 | 11.81647205 |
| FGFR1 | Fibroblast Growth Factor Receptor 1 | GC08M038400 | 11.80832481 |
| CAPN3 | Calpain 3 | GC15P042359 | 11.79447269 |
| CLDN16 | Claudin 16 | GC03P190290 | 11.78479099 |
| FOXO3 | Forkhead Box O3 | GC06P108559 | 11.77525711 |
| B4GALT1 | Beta-1,4-Galactosyltransferase 1 | GC09M033100 | 11.76719666 |
| MIR126 | MicroRNA 126 | GC09P136670 | 11.75726891 |
| LONP1 | Lon Peptidase 1, Mitochondrial | GC19M005691 | 11.75391674 |
| MGAM | Maltase-Glucoamylase | GC07P165315 | 11.75201988 |
| LOC102724023 | Glutamine Amidotransferase Class 1 Domain Containing 3B | GC21M005161 | 11.75059605 |
| HSPA1A | Heat Shock Protein Family A (Hsp70) Member 1A | GC06P173097 | 11.74705315 |
| SARS2 | Seryl-TRNA Synthetase 2, Mitochondrial | GC19M104949 | 11.7422905 |
| KCNJ1 | Potassium Inwardly Rectifying Channel Subfamily J Member 1 | GC11M137616 | 11.74026966 |
| TLR2 | Toll Like Receptor 2 | GC04P153684 | 11.73698616 |
| NDUFS8 | NADH:Ubiquinone Oxidoreductase Core Subunit S8 | GC11P068030 | 11.73024368 |
| SAT1 | Spermidine/Spermine N1-Acetyltransferase 1 | GC0XP023784 | 11.72956181 |
| FGF21 | Fibroblast Growth Factor 21 | GC19P143268 | 11.68255234 |
| CHST6 | Carbohydrate Sulfotransferase 6 | GC16M075472 | 11.6601553 |
| CACNA1S | Calcium Voltage-Gated Channel Subunit Alpha1 S | GC01M201008 | 11.64640617 |
| LDHB | Lactate Dehydrogenase B | GC12M021635 | 11.64400101 |
| C1QBP | Complement C1q Binding Protein | GC17M005432 | 11.62441349 |
| NANS | N-Acetylneuraminate Synthase | GC09P098056 | 11.62330818 |
| MLXIPL | MLX Interacting Protein Like | GC07M073593 | 11.61709213 |
| SLC25A10 | Solute Carrier Family 25 Member 10 | GC17P081712 | 11.61107826 |
| PGM3 | Phosphoglucomutase 3 | GC06M103994 | 11.60212135 |
| UMOD | Uromodulin | GC16M047825 | 11.59011459 |
| MT-TK | Mitochondrially Encoded TRNA-Lys (AAA/G) | GCMTP008297 | 11.57831955 |
| CRH | Corticotropin Releasing Hormone | GC08M066176 | 11.57795334 |
| UGT1A3 | UDP Glucuronosyltransferase Family 1 Member A3 | GC02P233729 | 11.57577419 |
| BAAT | Bile Acid-CoA:Amino Acid N-Acyltransferase | GC09M101354 | 11.57321548 |
| SPG7 | SPG7 Matrix AAA Peptidase Subunit, Paraplegin | GC16P115056 | 11.56144238 |
| ALG11 | ALG11 Alpha-1,2-Mannosyltransferase | GC13P052012 | 11.55691338 |
| SLC35A2 | Solute Carrier Family 35 Member A2 | GC0XM048903 | 11.54799366 |
| FLNA | Filamin A | GC0XM154348 | 11.54266548 |
| IGFBP1 | Insulin Like Growth Factor Binding Protein 1 | GC07P050731 | 11.5408392 |
| IRS2 | Insulin Receptor Substrate 2 | GC13M109752 | 11.51904202 |
| MIR17 | MicroRNA 17 | GC13P091350 | 11.51851463 |
| ISCA2 | Iron-Sulfur Cluster Assembly 2 | GC14P074493 | 11.50975132 |
| AGT | Angiotensinogen | GC01M230690 | 11.49765587 |
| ALDH9A1 | Aldehyde Dehydrogenase 9 Family Member A1 | GC01M166652 | 11.49586678 |
| DDIT3 | DNA Damage Inducible Transcript 3 | GC12M057516 | 11.48817062 |
| NBAS | NBAS Subunit Of NRZ Tethering Complex | GC02M014802 | 11.48739338 |
| PEX11B | Peroxisomal Biogenesis Factor 11 Beta | GC01M145911 | 11.47562981 |
| BCL2 | BCL2 Apoptosis Regulator | GC18M063123 | 11.45497131 |
| MAN1B1 | Mannosidase Alpha Class 1B Member 1 | GC09P137086 | 11.4483881 |
| MIR140 | MicroRNA 140 | GC16P114132 | 11.43494987 |
| MRPS22 | Mitochondrial Ribosomal Protein S22 | GC03P139005 | 11.43214893 |
| SLC27A4 | Solute Carrier Family 27 Member 4 | GC09P128340 | 11.41343498 |
| TNFSF11 | TNF Superfamily Member 11 | GC13P042562 | 11.40683079 |
| PRL | Prolactin | GC06M022287 | 11.40584755 |
| ALG12 | ALG12 Alpha-1,6-Mannosyltransferase | GC22M049859 | 11.3920517 |
| DPM2 | Dolichyl-Phosphate Mannosyltransferase Subunit 2, Regulatory | GC09M127935 | 11.38686752 |
| MEG3 | Maternally Expressed 3 | GC14P120211 | 11.36033154 |
| NFKBIA | NFKB Inhibitor Alpha | GC14M035401 | 11.35929012 |
| SREBF2 | Sterol Regulatory Element Binding Transcription Factor 2 | GC22P041833 | 11.32972813 |
| ALDH1A3 | Aldehyde Dehydrogenase 1 Family Member A3 | GC15P185363 | 11.32918262 |
| MIR29C | MicroRNA 29c | GC01M207838 | 11.31453705 |
| GRIN1 | Glutamate Ionotropic Receptor NMDA Type Subunit 1 | GC09P137138 | 11.30193996 |
| RPS27A | Ribosomal Protein S27a | GC02P055231 | 11.29724312 |
| DNM2 | Dynamin 2 | GC19P010718 | 11.29441261 |
| GNPTG | N-Acetylglucosamine-1-Phosphate Transferase Subunit Gamma | GC16P001351 | 11.29387093 |
| SCARNA5 | Small Cajal Body-Specific RNA 5 | GC02P233275 | 11.26504326 |
| B2M | Beta-2-Microglobulin | GC15P044711 | 11.2598362 |
| SLC7A6 | Solute Carrier Family 7 Member 6 | GC16P114029 | 11.25870037 |
| PEX16 | Peroxisomal Biogenesis Factor 16 | GC11M136213 | 11.25359821 |
| C19orf12 | Chromosome 19 Open Reading Frame 12 | GC19M104809 | 11.2534523 |
| EXT1 | Exostosin Glycosyltransferase 1 | GC08M117798 | 11.25114059 |
| ALG13 | ALG13 UDP-N-Acetylglucosaminyltransferase Subunit | GC0XP111665 | 11.24210739 |
| NT5C3A | 5'-Nucleotidase, Cytosolic IIIA | GC07M033014 | 11.22895622 |
| CYCS | Cytochrome C, Somatic | GC07M025118 | 11.19246006 |
| GSK3B | Glycogen Synthase Kinase 3 Beta | GC03M119821 | 11.17722321 |
| PNPLA6 | Patatin Like Phospholipase Domain Containing 6 | GC19P007534 | 11.17516518 |
| YAP1 | Yes1 Associated Transcriptional Regulator | GC11P102110 | 11.1747551 |
| PYY | Peptide YY | GC17M043952 | 11.1689539 |
| SERPINC1 | Serpin Family C Member 1 | GC01M175109 | 11.16003418 |
| PEX13 | Peroxisomal Biogenesis Factor 13 | GC02P061017 | 11.15744972 |
| APTX | Aprataxin | GC09M032886 | 11.1542263 |
| FGFR2 | Fibroblast Growth Factor Receptor 2 | GC10M121478 | 11.14712238 |
| PLCB1 | Phospholipase C Beta 1 | GC20P008077 | 11.14561844 |
| AFG3L2 | AFG3 Like Matrix AAA Peptidase Subunit 2 | GC18M012328 | 11.14281178 |
| BHMT | Betaine--Homocysteine S-Methyltransferase | GC05P079111 | 11.13214302 |
| VDAC1 | Voltage Dependent Anion Channel 1 | GC05M133975 | 11.12866688 |
| ATP6AP1 | ATPase H+ Transporting Accessory Protein 1 | GC0XP154428 | 11.10608673 |
| CYC1 | Cytochrome C1 | GC08P144095 | 11.10112476 |
| POMT1 | Protein O-Mannosyltransferase 1 | GC09P131502 | 11.09024239 |
| ST3GAL3 | ST3 Beta-Galactoside Alpha-2,3-Sialyltransferase 3 | GC01P043705 | 11.08789635 |
| GLUD2 | Glutamate Dehydrogenase 2 | GC0XP121047 | 11.08719635 |
| MPDU1 | Mannose-P-Dolichol Utilization Defect 1 | GC17P007583 | 11.07850838 |
| CHKA | Choline Kinase Alpha | GC11M068052 | 11.07675838 |
| KCTD7 | Potassium Channel Tetramerization Domain Containing 7 | GC07P066628 | 11.07123947 |
| CHROMR | Cholesterol Induced Regulator Of Metabolism RNA | GC02P178459 | 11.07118225 |
| ALG3 | ALG3 Alpha-1,3- Mannosyltransferase | GC03M184244 | 11.05346298 |
| CYBA | Cytochrome B-245 Alpha Chain | GC16M088643 | 11.05134392 |
| TUBB | Tubulin Beta Class I | GC06P173060 | 11.0314579 |
| PLP1 | Proteolipid Protein 1 | GC0XP103773 | 11.01271915 |
| DARS1 | Aspartyl-TRNA Synthetase 1 | GC02M135905 | 11.00008297 |
| TUG1 | Taurine Up-Regulated 1 | GC22P030969 | 10.99765301 |
| HAL | Histidine Ammonia-Lyase | GC12M095972 | 10.983181 |
| KCNJ10 | Potassium Inwardly Rectifying Channel Subfamily J Member 10 | GC01M159998 | 10.98106861 |
| MIR125A | MicroRNA 125a | GC19P143413 | 10.97682953 |
| MAPK8 | Mitogen-Activated Protein Kinase 8 | GC10P048306 | 10.96812057 |
| CTSB | Cathepsin B | GC08M011842 | 10.9356575 |
| PEX3 | Peroxisomal Biogenesis Factor 3 | GC06P143450 | 10.93384457 |
| SLC6A9 | Solute Carrier Family 6 Member 9 | GC01M043991 | 10.92117977 |
| AKR1C3 | Aldo-Keto Reductase Family 1 Member C3 | GC10P005035 | 10.91040516 |
| COX16 | Cytochrome C Oxidase Assembly Factor COX16 | GC14M070326 | 10.90239716 |
| CD40LG | CD40 Ligand | GC0XP136649 | 10.89560223 |
| ATF4 | Activating Transcription Factor 4 | GC22P039519 | 10.87639904 |
| EPRS1 | Glutamyl-Prolyl-TRNA Synthetase 1 | GC01M219969 | 10.87620258 |
| LINC02605 | Long Intergenic Non-Protein Coding RNA 2605 | GC08P078838 | 10.8758316 |
| MFSD8 | Major Facilitator Superfamily Domain Containing 8 | GC04M127917 | 10.85881615 |
| SUGCT | Succinyl-CoA:Glutarate-CoA Transferase | GC07P041295 | 10.81506729 |
| DHFR2 | Dihydrofolate Reductase 2 | GC03M094048 | 10.81399822 |
| POLR3A | RNA Polymerase III Subunit A | GC10M079303 | 10.80871105 |
| FOXO1 | Forkhead Box O1 | GC13M040555 | 10.80407906 |
| POMGNT2 | Protein O-Linked Mannose N-Acetylglucosaminyltransferase 2 (Beta 1,4-) | GC03M043121 | 10.80118084 |
| TRAP1 | TNF Receptor Associated Protein 1 | GC16M046535 | 10.78721333 |
| SLC5A6 | Solute Carrier Family 5 Member 6 | GC02M027201 | 10.78467655 |
| SLC38A5 | Solute Carrier Family 38 Member 5 | GC0XM048458 | 10.78462982 |
| ARNT | Aryl Hydrocarbon Receptor Nuclear Translocator | GC01M150809 | 10.77245235 |
| POMT2 | Protein O-Mannosyltransferase 2 | GC14M077274 | 10.76476097 |
| SLC39A4 | Solute Carrier Family 39 Member 4 | GC08M144409 | 10.76169872 |
| SLC7A8 | Solute Carrier Family 7 Member 8 | GC14M023125 | 10.75879097 |
| ST3GAL5 | ST3 Beta-Galactoside Alpha-2,3-Sialyltransferase 5 | GC02M085880 | 10.7522707 |
| FKTN | Fukutin | GC09P105558 | 10.75154209 |
| ACACB | Acetyl-CoA Carboxylase Beta | GC12P109116 | 10.74660301 |
| SCN1A | Sodium Voltage-Gated Channel Alpha Subunit 1 | GC02M165989 | 10.74294472 |
| TIMM8A | Translocase Of Inner Mitochondrial Membrane 8A | GC0XM101345 | 10.74285698 |
| GGH | Gamma-Glutamyl Hydrolase | GC08M063014 | 10.73801231 |
| DDOST | Dolichyl-Diphosphooligosaccharide--Protein Glycosyltransferase Non-Catalytic Subunit | GC01M020651 | 10.73528671 |
| PARP1 | Poly(ADP-Ribose) Polymerase 1 | GC01M226360 | 10.72192574 |
| TARS2 | Threonyl-TRNA Synthetase 2, Mitochondrial | GC01P173171 | 10.70739079 |
| MAPK3 | Mitogen-Activated Protein Kinase 3 | GC16M048460 | 10.70246601 |
| PDE5A | Phosphodiesterase 5A | GC04M119494 | 10.70125866 |
| TXN2 | Thioredoxin 2 | GC22M036467 | 10.69442844 |
| DPM3 | Dolichyl-Phosphate Mannosyltransferase Subunit 3, Regulatory | GC01M155139 | 10.67701435 |
| CANT1 | Calcium Activated Nucleotidase 1 | GC17M094382 | 10.67049694 |
| MDH1 | Malate Dehydrogenase 1 | GC02P063557 | 10.66633701 |
| TIMP1 | TIMP Metallopeptidase Inhibitor 1 | GC0XP060044 | 10.66416645 |
| COQ5 | Coenzyme Q5, Methyltransferase | GC12M120503 | 10.66295147 |
| ADH1C | Alcohol Dehydrogenase 1C (Class I), Gamma Polypeptide | GC04M099336 | 10.62932205 |
| RNASEH2A | Ribonuclease H2 Subunit A | GC19P142366 | 10.6287775 |
| FAR1 | Fatty Acyl-CoA Reductase 1 | GC11P013668 | 10.62806892 |
| SHMT2 | Serine Hydroxymethyltransferase 2 | GC12P057229 | 10.62048531 |
| SPP1 | Secreted Phosphoprotein 1 | GC04P087975 | 10.59573078 |
| TG | Thyroglobulin | GC08P132866 | 10.5941391 |
| IL13 | Interleukin 13 | GC05P132656 | 10.59398174 |
| SLC35A1 | Solute Carrier Family 35 Member A1 | GC06P087470 | 10.59163284 |
| FBN1 | Fibrillin 1 | GC15M048408 | 10.58854675 |
| ELOVL4 | ELOVL Fatty Acid Elongase 4 | GC06M079914 | 10.58157063 |
| MT-TW | Mitochondrially Encoded TRNA-Trp (UGA/G) | GCMTP005514 | 10.57981777 |
| CHAT | Choline O-Acetyltransferase | GC10P049609 | 10.57715416 |
| CALM1 | Calmodulin 1 | GC14P090396 | 10.57622623 |
| PGR | Progesterone Receptor | GC11M137169 | 10.57378387 |
| SLC35C1 | Solute Carrier Family 35 Member C1 | GC11P048644 | 10.53328705 |
| FMR1 | Fragile X Messenger Ribonucleoprotein 1 | GC0XP148000 | 10.53322887 |
| GPC3 | Glypican 3 | GC0XM133535 | 10.52923393 |
| PGR-AS1 | PGR Antisense RNA 1 | GC11P104498 | 10.52827835 |
| TGM4 | Transglutaminase 4 | GC03P044874 | 10.52811718 |
| PEX19 | Peroxisomal Biogenesis Factor 19 | GC01M160276 | 10.52682495 |
| FUT8 | Fucosyltransferase 8 | GC14P065356 | 10.52526283 |
| COL1A1 | Collagen Type I Alpha 1 Chain | GC17M093791 | 10.51510715 |
| ALG14 | ALG14 UDP-N-Acetylglucosaminyltransferase Subunit | GC01M094974 | 10.51188087 |
| PRNP | Prion Protein (Kanno Blood Group) | GC20P004686 | 10.51057243 |
| VEGFA | Vascular Endothelial Growth Factor A | GC06P043770 | 10.50355721 |
| MAP2K1 | Mitogen-Activated Protein Kinase Kinase 1 | GC15P066386 | 10.4947443 |
| MT-TI | Mitochondrially Encoded TRNA-Ile (AUU/C) | GCMTP004265 | 10.46230984 |
| ELN | Elastin | GC07P074027 | 10.45741081 |
| AGXT2 | Alanine--Glyoxylate Aminotransferase 2 | GC05M034998 | 10.4467001 |
| NDUFB10 | NADH:Ubiquinone Oxidoreductase Subunit B10 | GC16P112280 | 10.44127083 |
| GMPPB | GDP-Mannose Pyrophosphorylase B | GC03M049716 | 10.43841648 |
| CSN1S1 | Casein Alpha S1 | GC04P070085 | 10.43154716 |
| GRIA2 | Glutamate Ionotropic Receptor AMPA Type Subunit 2 | GC04P157204 | 10.42880726 |
| LOC107133510 | Origin Of Replication At HBB | GC11P005222 | 10.42868137 |
| AGTR2 | Angiotensin II Receptor Type 2 | GC0XP116170 | 10.42147064 |
| PIK3C2A | Phosphatidylinositol-4-Phosphate 3-Kinase Catalytic Subunit Type 2 Alpha | GC11M018080 | 10.40409851 |
| GMPPA | GDP-Mannose Pyrophosphorylase A | GC02P219498 | 10.39910126 |
| APC | APC Regulator Of WNT Signaling Pathway | GC05P112707 | 10.38339806 |
| CDK5 | Cyclin Dependent Kinase 5 | GC07M151053 | 10.3801899 |
| MGAT2 | Alpha-1,6-Mannosyl-Glycoprotein 2-Beta-N-Acetylglucosaminyltransferase | GC14P049620 | 10.37322044 |
| SEC23B | SEC23 Homolog B, COPII Coat Complex Component | GC20P018507 | 10.36030293 |
| ATP6V1E1 | ATPase H+ Transporting V1 Subunit E1 | GC22M017592 | 10.35076141 |
| NCOA3 | Nuclear Receptor Coactivator 3 | GC20P047501 | 10.34651375 |
| SETX | Senataxin | GC09M132261 | 10.34380436 |
| SMN1 | Survival Of Motor Neuron 1, Telomeric | GC05P070924 | 10.33611107 |
| LAMA2 | Laminin Subunit Alpha 2 | GC06P174242 | 10.3329401 |
| TRPM6 | Transient Receptor Potential Cation Channel Subfamily M Member 6 | GC09M074725 | 10.32810402 |
| KEAP1 | Kelch Like ECH Associated Protein 1 | GC19M010486 | 10.32637882 |
| HNRNPA2B1 | Heterogeneous Nuclear Ribonucleoprotein A2/B1 | GC07M026174 | 10.30999374 |
| NOS2 | Nitric Oxide Synthase 2 | GC17M027756 | 10.3086977 |
| MIPEP | Mitochondrial Intermediate Peptidase | GC13M023730 | 10.3061285 |
| KCNA1 | Potassium Voltage-Gated Channel Subfamily A Member 1 | GC12P068224 | 10.30032444 |
| IKBKB | Inhibitor Of Nuclear Factor Kappa B Kinase Subunit Beta | GC08P042271 | 10.29836655 |
| OGT | O-Linked N-Acetylglucosamine (GlcNAc) Transferase | GC0XP071969 | 10.28530121 |
| SLC25A21 | Solute Carrier Family 25 Member 21 | GC14M036677 | 10.28021431 |
| TGFBR2 | Transforming Growth Factor Beta Receptor 2 | GC03P030608 | 10.27769089 |
| HAAO | 3-Hydroxyanthranilate 3,4-Dioxygenase | GC02M042767 | 10.27198696 |
| PIGV | Phosphatidylinositol Glycan Anchor Biosynthesis Class V | GC01P026787 | 10.26628876 |
| TRA-TGC7-1 | TRNA-Ala (Anticodon TGC) 7-1 | GC06M103186 | 10.26441765 |
| GLO1 | Glyoxalase I | GC06M103484 | 10.25751305 |
| MIR29A | MicroRNA 29a | GC07M130876 | 10.25665855 |
| MAOB | Monoamine Oxidase B | GC0XM043766 | 10.25353718 |
| CUL3 | Cullin 3 | GC02M224470 | 10.25195122 |
| UGT1A | UDP Glucuronosyltransferase Family 1 Member A Complex Locus | GC02P234634 | 10.24012852 |
| FOXP2 | Forkhead Box P2 | GC07P114086 | 10.23982048 |
| PYCR2 | Pyrroline-5-Carboxylate Reductase 2 | GC01M225919 | 10.23709679 |
| ANO10 | Anoctamin 10 | GC03M043355 | 10.2087574 |
| KMT2D | Lysine Methyltransferase 2D | GC12M049018 | 10.19955635 |
| SACS | Sacsin Molecular Chaperone | GC13M023288 | 10.18536949 |
| PIGT | Phosphatidylinositol Glycan Anchor Biosynthesis Class T | GC20P045416 | 10.17785549 |
| EGFR | Epidermal Growth Factor Receptor | GC07P055019 | 10.16936302 |
| CLOCK | Clock Circadian Regulator | GC04M055427 | 10.16184425 |
| THAP11 | THAP Domain Containing 11 | GC16P114015 | 10.16147232 |
| ALG9 | ALG9 Alpha-1,2-Mannosyltransferase | GC11M137313 | 10.15806866 |
| AFP | Alpha Fetoprotein | GC04P073431 | 10.14544964 |
| GPIHBP1 | Glycosylphosphatidylinositol Anchored High Density Lipoprotein Binding Protein 1 | GC08P143213 | 10.14039707 |
| CAV3 | Caveolin 3 | GC03P008733 | 10.13596439 |
| NOS1 | Nitric Oxide Synthase 1 | GC12M117208 | 10.13528824 |
| MT-TS1 | Mitochondrially Encoded TRNA-Ser (UCN) 1 | GCMTM007447 | 10.12875748 |
| LOX | Lysyl Oxidase | GC05M122063 | 10.10852623 |
| PEX26 | Peroxisomal Biogenesis Factor 26 | GC22P086254 | 10.10341358 |
| PLCB4 | Phospholipase C Beta 4 | GC20P009067 | 10.09358692 |
| IARS2 | Isoleucyl-TRNA Synthetase 2, Mitochondrial | GC01P220094 | 10.07344151 |
| NMNAT1 | Nicotinamide Nucleotide Adenylyltransferase 1 | GC01P068109 | 10.06043339 |
| BRCA2 | BRCA2 DNA Repair Associated | GC13P032315 | 10.04496574 |
| RNASEH2C | Ribonuclease H2 Subunit C | GC11M065714 | 10.03872013 |
| EXT2 | Exostosin Glycosyltransferase 2 | GC11P044095 | 10.03415298 |
| ALDH1A1 | Aldehyde Dehydrogenase 1 Family Member A1 | GC09M072900 | 10.03242016 |
| COG8 | Component Of Oligomeric Golgi Complex 8 | GC16M069320 | 10.03160381 |
| MAF | MAF BZIP Transcription Factor | GC16M079204 | 10.0229702 |
| SLC2A3 | Solute Carrier Family 2 Member 3 | GC12M007919 | 9.988202095 |
| CALR | Calreticulin | GC19P012938 | 9.968200684 |
| RPL10 | Ribosomal Protein L10 | GC0XP154389 | 9.950051308 |
| UGT2B7 | UDP Glucuronosyltransferase Family 2 Member B7 | GC04P069051 | 9.932118416 |
| GABRG2 | Gamma-Aminobutyric Acid Type A Receptor Subunit Gamma2 | GC05P162000 | 9.925057411 |
| HCRT | Hypocretin Neuropeptide Precursor | GC17M093436 | 9.916570663 |
| SLC6A20 | Solute Carrier Family 6 Member 20 | GC03M045755 | 9.908359528 |
| SLC13A5 | Solute Carrier Family 13 Member 5 | GC17M006684 | 9.896652222 |
| EPO | Erythropoietin | GC07P100720 | 9.87967205 |
| STXBP1 | Syntaxin Binding Protein 1 | GC09P149721 | 9.876630783 |
| PRKACA | Protein Kinase CAMP-Activated Catalytic Subunit Alpha | GC19M104483 | 9.853081703 |
| ABCA2 | ATP Binding Cassette Subfamily A Member 2 | GC09M137007 | 9.851828575 |
| DYSF | Dysferlin | GC02P071453 | 9.850201607 |
| KRIT1 | KRIT1 Ankyrin Repeat Containing | GC07M092198 | 9.817598343 |
| TLR4 | Toll Like Receptor 4 | GC09P117704 | 9.811986923 |
| PDK1 | Pyruvate Dehydrogenase Kinase 1 | GC02P172555 | 9.806559563 |
| NRF1 | Nuclear Respiratory Factor 1 | GC07P129611 | 9.805028915 |
| TRIT1 | TRNA Isopentenyltransferase 1 | GC01M039842 | 9.799651146 |
| FCSK | Fucose Kinase | GC16P070454 | 9.796504974 |
| RARS1 | Arginyl-TRNA Synthetase 1 | GC05P168487 | 9.790771484 |
| XPC | XPC Complex Subunit, DNA Damage Recognition And Repair Factor | GC03M028072 | 9.786586761 |
| DNAJC5 | DnaJ Heat Shock Protein Family (Hsp40) Member C5 | GC20P063895 | 9.782218933 |
| LGALS3 | Galectin 3 | GC14P055124 | 9.772520065 |
| CYBB | Cytochrome B-245 Beta Chain | GC0XP037780 | 9.752924919 |
| NDUFC2 | NADH:Ubiquinone Oxidoreductase Subunit C2 | GC11M078068 | 9.752007484 |
| FABP1 | Fatty Acid Binding Protein 1 | GC02M088122 | 9.748911858 |
| RDH5 | Retinol Dehydrogenase 5 | GC12P055720 | 9.748641968 |
| IL3 | Interleukin 3 | GC05P132060 | 9.740466118 |
| LOC106099062 | HBB Recombination Region | GC11P015945 | 9.73939991 |
| ESRRA | Estrogen Related Receptor Alpha | GC11P064305 | 9.736309052 |
| AAAS | Aladin WD Repeat Nucleoporin | GC12M053307 | 9.72961998 |
| NSF | N-Ethylmaleimide Sensitive Factor, Vesicle Fusing ATPase | GC17P046590 | 9.725997925 |
| GRIN2A | Glutamate Ionotropic Receptor NMDA Type Subunit 2A | GC16M009753 | 9.721117973 |
| IDE | Insulin Degrading Enzyme | GC10M092451 | 9.709253311 |
| CASP3 | Caspase 3 | GC04M184627 | 9.707775116 |
| FAAH | Fatty Acid Amide Hydrolase | GC01P046394 | 9.700348854 |
| TNNI3 | Troponin I3, Cardiac Type | GC19M055151 | 9.695373535 |
| CNNM2 | Cyclin And CBS Domain Divalent Metal Cation Transport Mediator 2 | GC10P102918 | 9.695175171 |
| CALM3 | Calmodulin 3 | GC19P046601 | 9.693282127 |
| HEMK1 | HemK Methyltransferase Family Member 1 | GC03P050569 | 9.691810608 |
| GFER | Growth Factor, Augmenter Of Liver Regeneration | GC16P001984 | 9.691785812 |
| HLA-DQB1 | Major Histocompatibility Complex, Class II, DQ Beta 1 | GC06M103395 | 9.688340187 |
| COG4 | Component Of Oligomeric Golgi Complex 4 | GC16M074198 | 9.670291901 |
| COG5 | Component Of Oligomeric Golgi Complex 5 | GC07M107201 | 9.666934967 |
| SKIC2 | SKI2 Subunit Of Superkiller Complex | GC06P181637 | 9.660303116 |
| IL6R | Interleukin 6 Receptor | GC01P154405 | 9.660153389 |
| MBP | Myelin Basic Protein | GC18M076978 | 9.654903412 |
| NR0B2 | Nuclear Receptor Subfamily 0 Group B Member 2 | GC01M034192 | 9.654506683 |
| RFT1 | RFT1 Homolog | GC03M054672 | 9.626420975 |
| MME | Membrane Metalloendopeptidase | GC03P155024 | 9.625106812 |
| NAT1 | N-Acetyltransferase 1 | GC08P018183 | 9.59965992 |
| UQCC3 | Ubiquinol-Cytochrome C Reductase Complex Assembly Factor 3 | GC11P062670 | 9.598880768 |
| PQBP1 | Polyglutamine Binding Protein 1 | GC0XP048890 | 9.593372345 |
| NLRP3 | NLR Family Pyrin Domain Containing 3 | GC01P247583 | 9.583311081 |
| IDO1 | Indoleamine 2,3-Dioxygenase 1 | GC08P041407 | 9.580322266 |
| PAX4 | Paired Box 4 | GC07M127610 | 9.57721138 |
| NR5A1 | Nuclear Receptor Subfamily 5 Group A Member 1 | GC09M124481 | 9.572153091 |
| RBCK1 | RANBP2-Type And C3HC4-Type Zinc Finger Containing 1 | GC20P000407 | 9.569239616 |
| ELANE | Elastase, Neutrophil Expressed | GC19P141796 | 9.569225311 |
| STX1A | Syntaxin 1A | GC07M079904 | 9.553916931 |
| MC2R | Melanocortin 2 Receptor | GC18M037149 | 9.553747177 |
| PGAP1 | Post-GPI Attachment To Proteins Inositol Deacylase 1 | GC02M196833 | 9.553399086 |
| SLC6A4 | Solute Carrier Family 6 Member 4 | GC17M030194 | 9.524742126 |
| F7 | Coagulation Factor VII | GC13P113105 | 9.521521568 |
| SYP | Synaptophysin | GC0XM049187 | 9.516494751 |
| GARS1 | Glycyl-TRNA Synthetase 1 | GC07P030580 | 9.516155243 |
| FXYD2 | FXYD Domain Containing Ion Transport Regulator 2 | GC11M117800 | 9.509632111 |
| F10 | Coagulation Factor X | GC13P113122 | 9.504648209 |
| TXNIP | Thioredoxin Interacting Protein | GC01M145992 | 9.499753952 |
| WT1 | WT1 Transcription Factor | GC11M032365 | 9.497394562 |
| SLC9A1 | Solute Carrier Family 9 Member A1 | GC01M034197 | 9.495974541 |
| CRYAB | Crystallin Alpha B | GC11M111908 | 9.495055199 |
| TGM5 | Transglutaminase 5 | GC15M047033 | 9.492005348 |
| LTA4H | Leukotriene A4 Hydrolase | GC12M096000 | 9.490644455 |
| RAP1A | RAP1A, Member Of RAS Oncogene Family | GC01P111542 | 9.489685059 |
| HTR1A | 5-Hydroxytryptamine Receptor 1A | GC05M063960 | 9.475104332 |
| TRAPPC11 | Trafficking Protein Particle Complex Subunit 11 | GC04P183659 | 9.468728065 |
| PLAT | Plasminogen Activator, Tissue Type | GC08M042174 | 9.467177391 |
| GDAP1 | Ganglioside Induced Differentiation Associated Protein 1 | GC08P074315 | 9.460536003 |
| WNK1 | WNK Lysine Deficient Protein Kinase 1 | GC12P000804 | 9.450969696 |
| SSBP1 | Single Stranded DNA Binding Protein 1 | GC07P165436 | 9.450058937 |
| TMEM126A | Transmembrane Protein 126A | GC11P085647 | 9.448159218 |
| F8 | Coagulation Factor VIII | GC0XM154835 | 9.447523117 |
| MSTO1 | Misato Mitochondrial Distribution And Morphology Regulator 1 | GC01P173498 | 9.445036888 |
| NGF | Nerve Growth Factor | GC01M115285 | 9.429849625 |
| PCNA | Proliferating Cell Nuclear Antigen | GC20M005114 | 9.424021721 |
| VAMP2 | Vesicle Associated Membrane Protein 2 | GC17M092463 | 9.421070099 |
| HBA2 | Hemoglobin Subunit Alpha 2 | GC16P112212 | 9.411801338 |
| SFTPD | Surfactant Protein D | GC10M079937 | 9.404728889 |
| HLA-B | Major Histocompatibility Complex, Class I, B | GC06M103303 | 9.399683952 |
| HSF1 | Heat Shock Transcription Factor 1 | GC08P144291 | 9.397157669 |
| ANK2 | Ankyrin 2 | GC04P112784 | 9.396910667 |
| SSR4 | Signal Sequence Receptor Subunit 4 | GC0XP153793 | 9.382707596 |
| NAXD | NAD(P)HX Dehydratase | GC13P111112 | 9.378912926 |
| PDE10A | Phosphodiesterase 10A | GC06M165327 | 9.368116379 |
| SLC25A26 | Solute Carrier Family 25 Member 26 | GC03P066120 | 9.359298706 |
| CKM | Creatine Kinase, M-Type | GC19M045306 | 9.356999397 |
| HGF | Hepatocyte Growth Factor | GC07M081699 | 9.353874207 |
| PRDX2 | Peroxiredoxin 2 | GC19M012796 | 9.350566864 |
| SLC38A6 | Solute Carrier Family 38 Member 6 | GC14P060982 | 9.341609955 |
| RAI1 | Retinoic Acid Induced 1 | GC17P143676 | 9.341053009 |
| PNMT | Phenylethanolamine N-Methyltransferase | GC17P039667 | 9.334937096 |
| LTF | Lactotransferrin | GC03M046435 | 9.329304695 |
| VIM | Vimentin | GC10P017227 | 9.321445465 |
| EEF2 | Eukaryotic Translation Elongation Factor 2 | GC19M003976 | 9.314314842 |
| GNRH1 | Gonadotropin Releasing Hormone 1 | GC08M025419 | 9.314014435 |
| OCLN | Occludin | GC05P069492 | 9.313781738 |
| PARK7 | Parkinsonism Associated Deglycase | GC01P068004 | 9.313253403 |
| MAGT1 | Magnesium Transporter 1 | GC0XM078304 | 9.306678772 |
| COG7 | Component Of Oligomeric Golgi Complex 7 | GC16M023388 | 9.305734634 |
| MIR151A | MicroRNA 151a | GC08M141021 | 9.305450439 |
| MIRLET7D | MicroRNA Let-7d | GC09P094178 | 9.299412727 |
| TUSC3 | Tumor Suppressor Candidate 3 | GC08P015417 | 9.278841972 |
| SLC16A2 | Solute Carrier Family 16 Member 2 | GC0XP075074 | 9.274560928 |
| HDAC1 | Histone Deacetylase 1 | GC01P032292 | 9.271518707 |
| CDH1 | Cadherin 1 | GC16P068737 | 9.258285522 |
| ACO1 | Aconitase 1 | GC09P032374 | 9.25583744 |
| ANO5 | Anoctamin 5 | GC11P021799 | 9.252451897 |
| FGF7 | Fibroblast Growth Factor 7 | GC15P049423 | 9.25217247 |
| SIL1 | SIL1 Nucleotide Exchange Factor | GC05M139006 | 9.238401413 |
| ABCC9 | ATP Binding Cassette Subfamily C Member 9 | GC12M021797 | 9.229595184 |
| GNA11 | G Protein Subunit Alpha 11 | GC19P003094 | 9.227647781 |
| PRKDC | Protein Kinase, DNA-Activated, Catalytic Subunit | GC08M047773 | 9.226218224 |
| CALM2 | Calmodulin 2 | GC02M047160 | 9.224974632 |
| TJP1 | Tight Junction Protein 1 | GC15M029699 | 9.212213516 |
| PLCD1 | Phospholipase C Delta 1 | GC03M038008 | 9.207649231 |
| SMAD4 | SMAD Family Member 4 | GC18P051028 | 9.20464325 |
| CLDN19 | Claudin 19 | GC01M042733 | 9.204106331 |
| EEF1A1 | Eukaryotic Translation Elongation Factor 1 Alpha 1 | GC06M103850 | 9.201346397 |
| PON2 | Paraoxonase 2 | GC07M095404 | 9.195746422 |
| CHST3 | Carbohydrate Sulfotransferase 3 | GC10P071964 | 9.183648109 |
| KCNJ5 | Potassium Inwardly Rectifying Channel Subfamily J Member 5 | GC11P128891 | 9.182001114 |
| GRIK2 | Glutamate Ionotropic Receptor Kainate Type Subunit 2 | GC06P100962 | 9.179366112 |
| ADORA2A | Adenosine A2a Receptor | GC22P024417 | 9.17698288 |
| ITGB1 | Integrin Subunit Beta 1 | GC10M036806 | 9.173579216 |
| PDYN | Prodynorphin | GC20M001978 | 9.172734261 |
| IMPDH2 | Inosine Monophosphate Dehydrogenase 2 | GC03M054491 | 9.164712906 |
| FADD | Fas Associated Via Death Domain | GC11P070203 | 9.162599564 |
| POU1F1 | POU Class 1 Homeobox 1 | GC03M087259 | 9.158439636 |
| SLC9A3 | Solute Carrier Family 9 Member A3 | GC05M000472 | 9.151894569 |
| C9orf72 | C9orf72-SMCR8 Complex Subunit | GC09M030021 | 9.14689827 |
| MRPS7 | Mitochondrial Ribosomal Protein S7 | GC17P145204 | 9.143486023 |
| B3GLCT | Beta 3-Glucosyltransferase | GC13P031225 | 9.13739872 |
| TRC-GCA24-1 | TRNA-Cys (GCA) 24-1 | GC17M093302 | 9.135396004 |
| ADARB1 | Adenosine Deaminase RNA Specific B1 | GC21P045073 | 9.12549305 |
| UGCG | UDP-Glucose Ceramide Glucosyltransferase | GC09P111896 | 9.124979019 |
| FUS | FUS RNA Binding Protein | GC16P031180 | 9.112576485 |
| RHO | Rhodopsin | GC03P141962 | 9.10483551 |
| GRM1 | Glutamate Metabotropic Receptor 1 | GC06P174683 | 9.099840164 |
| COG6 | Component Of Oligomeric Golgi Complex 6 | GC13P039655 | 9.094035149 |
| EIF4EBP1 | Eukaryotic Translation Initiation Factor 4E Binding Protein 1 | GC08P041341 | 9.080421448 |
| PRDX5 | Peroxiredoxin 5 | GC11P103214 | 9.068185806 |
| LOC129930446 | ATAC-STARR-Seq Lymphoblastoid Active Region 972 | GC01P074513 | 9.066045761 |
| TFAP2A | Transcription Factor AP-2 Alpha | GC06M010393 | 9.062432289 |
| SRC | SRC Proto-Oncogene, Non-Receptor Tyrosine Kinase | GC20P037344 | 9.056348801 |
| ADRA2A | Adrenoceptor Alpha 2A | GC10P111077 | 9.055519104 |
| SPG11 | SPG11 Vesicle Trafficking Associated, Spatacsin | GC15M047072 | 9.054834366 |
| NCOA2 | Nuclear Receptor Coactivator 2 | GC08M070109 | 9.052975655 |
| GRN | Granulin Precursor | GC17P044345 | 9.038557053 |
| MED23 | Mediator Complex Subunit 23 | GC06M131573 | 9.034222603 |
| IAPP | Islet Amyloid Polypeptide | GC12P021354 | 9.019491196 |
| LPA | Lipoprotein(A) | GC06M160531 | 9.018770218 |
| B3GALNT2 | Beta-1,3-N-Acetylgalactosaminyltransferase 2 | GC01M235440 | 9.007840157 |
| MIR155 | MicroRNA 155 | GC21P025573 | 9.00476265 |
| CRAT | Carnitine O-Acetyltransferase | GC09M129094 | 9.001458168 |
| PFKFB3 | 6-Phosphofructo-2-Kinase/Fructose-2,6-Biphosphatase 3 | GC10P006144 | 8.9957304 |
| SCN4A | Sodium Voltage-Gated Channel Alpha Subunit 4 | GC17M063938 | 8.993818283 |
| TGM7 | Transglutaminase 7 | GC15M043276 | 8.980308533 |
| PLCG1 | Phospholipase C Gamma 1 | GC20P041136 | 8.978312492 |
| N6AMT1 | N-6 Adenine-Specific DNA Methyltransferase 1 | GC21M028692 | 8.9759655 |
| PPA1 | Inorganic Pyrophosphatase 1 | GC10M070202 | 8.96472168 |
| COG1 | Component Of Oligomeric Golgi Complex 1 | GC17P073193 | 8.960363388 |
| HARS2 | Histidyl-TRNA Synthetase 2, Mitochondrial | GC05P157533 | 8.95861721 |
| SST | Somatostatin | GC03M187668 | 8.95777607 |
| ACP1 | Acid Phosphatase 1 | GC02P001141 | 8.951177597 |
| FTH1 | Ferritin Heavy Chain 1 | GC11M061959 | 8.949199677 |
| PGAP2 | Post-GPI Attachment To Proteins 2 | GC11P003797 | 8.940356255 |
| MAP2K2 | Mitogen-Activated Protein Kinase Kinase 2 | GC19M004090 | 8.937620163 |
| MFF | Mitochondrial Fission Factor | GC02P227325 | 8.932580948 |
| ABCC1 | ATP Binding Cassette Subfamily C Member 1 (ABCC1 Blood Group) | GC16P015949 | 8.929198265 |
| NFYA | Nuclear Transcription Factor Y Subunit Alpha | GC06P173234 | 8.918970108 |
| PIGM | Phosphatidylinositol Glycan Anchor Biosynthesis Class M | GC01M166491 | 8.914554596 |
| RERE | Arginine-Glutamic Acid Dipeptide Repeats | GC01M020880 | 8.912902832 |
| WNK4 | WNK Lysine Deficient Protein Kinase 4 | GC17P042780 | 8.912543297 |
| COL2A1 | Collagen Type II Alpha 1 Chain | GC12M047972 | 8.897524834 |
| GPX4 | Glutathione Peroxidase 4 | GC19P001103 | 8.893237114 |
| MRPL12 | Mitochondrial Ribosomal Protein L12 | GC17P145399 | 8.877819061 |
| DRD2 | Dopamine Receptor D2 | GC11M113409 | 8.867740631 |
| SLC10A1 | Solute Carrier Family 10 Member 1 | GC14M069775 | 8.86554718 |
| HDAC9 | Histone Deacetylase 9 | GC07P018086 | 8.858863831 |
| TPO | Thyroid Peroxidase | GC02P001374 | 8.857061386 |
| ACE2 | Angiotensin Converting Enzyme 2 | GC0XM015494 | 8.855583191 |
| FGFR3 | Fibroblast Growth Factor Receptor 3 | GC04P009307 | 8.844777107 |
| PIGY | Phosphatidylinositol Glycan Anchor Biosynthesis Class Y | GC04M088520 | 8.825649261 |
| NF1 | Neurofibromin 1 | GC17P031094 | 8.824237823 |
| TDO2 | Tryptophan 2,3-Dioxygenase | GC04P155854 | 8.822389603 |
| DCTN1 | Dynactin Subunit 1 | GC02M074361 | 8.819462776 |
| MT-TN | Mitochondrially Encoded TRNA-Asn (AAU/C) | GCMTM005659 | 8.817559242 |
| MIRLET7C | MicroRNA Let-7c | GC21P020733 | 8.81715107 |
| STT3B | STT3 Oligosaccharyltransferase Complex Catalytic Subunit B | GC03P031532 | 8.810343742 |
| EIF2B5 | Eukaryotic Translation Initiation Factor 2B Subunit Epsilon | GC03P184135 | 8.807779312 |
| CYP4V2 | Cytochrome P450 Family 4 Subfamily V Member 2 | GC04P186191 | 8.806609154 |
| SLC6A14 | Solute Carrier Family 6 Member 14 | GC0XP116436 | 8.806038857 |
| NEDD4L | NEDD4 Like E3 Ubiquitin Protein Ligase | GC18P058044 | 8.788567543 |
| UGDH | UDP-Glucose 6-Dehydrogenase | GC04M039502 | 8.785675049 |
| LRP5 | LDL Receptor Related Protein 5 | GC11P068298 | 8.778719902 |
| NCOA1 | Nuclear Receptor Coactivator 1 | GC02P024523 | 8.772275925 |
| STT3A | STT3 Oligosaccharyltransferase Complex Catalytic Subunit A | GC11P125592 | 8.769359589 |
| TMEM165 | Transmembrane Protein 165 | GC04P055395 | 8.763388634 |
| MED12 | Mediator Complex Subunit 12 | GC0XP071118 | 8.754285812 |
| PDE4A | Phosphodiesterase 4A | GC19P010416 | 8.752727509 |
| KMT2A | Lysine Methyltransferase 2A | GC11P118436 | 8.751491547 |
| HELLS | Helicase, Lymphoid Specific | GC10P120190 | 8.748425484 |
| TARDBP | TAR DNA Binding Protein | GC01P068175 | 8.748035431 |
| PABPN1 | Poly(A) Binding Protein Nuclear 1 | GC14P056868 | 8.743257523 |
| MIR200C | MicroRNA 200c | GC12P068289 | 8.723919868 |
| FPGS | Folylpolyglutamate Synthase | GC09P127794 | 8.720049858 |
| PIK3C3 | Phosphatidylinositol 3-Kinase Catalytic Subunit Type 3 | GC18P041955 | 8.718434334 |
| DYM | Dymeclin | GC18M049041 | 8.710954666 |
| CACNA1C | Calcium Voltage-Gated Channel Subunit Alpha1 C | GC12P001970 | 8.70710659 |
| SMAD3 | SMAD Family Member 3 | GC15P067063 | 8.705763817 |
| MMP3 | Matrix Metallopeptidase 3 | GC11M102835 | 8.681900024 |
| VCL | Vinculin | GC10P073995 | 8.671857834 |
| NFKB1 | Nuclear Factor Kappa B Subunit 1 | GC04P102501 | 8.67108345 |
| ARX | Aristaless Related Homeobox | GC0XM025003 | 8.666729927 |
| MGP | Matrix Gla Protein | GC12M034613 | 8.66147995 |
| POMK | Protein O-Mannose Kinase | GC08P043093 | 8.660154343 |
| TGFB2 | Transforming Growth Factor Beta 2 | GC01P218345 | 8.651433945 |
| JUN | Jun Proto-Oncogene, AP-1 Transcription Factor Subunit | GC01M058780 | 8.647699356 |
| KIT | KIT Proto-Oncogene, Receptor Tyrosine Kinase | GC04P054657 | 8.647194862 |
| TACR1 | Tachykinin Receptor 1 | GC02M075798 | 8.644498825 |
| SPTAN1 | Spectrin Alpha, Non-Erythrocytic 1 | GC09P128552 | 8.642086029 |
| CERKL | CERK Like Autophagy Regulator | GC02M181536 | 8.640823364 |
| AGTR1 | Angiotensin II Receptor Type 1 | GC03P148697 | 8.639716148 |
| NME1 | NME/NM23 Nucleoside Diphosphate Kinase 1 | GC17P144771 | 8.626558304 |
| SETD2 | SET Domain Containing 2, Histone Lysine Methyltransferase | GC03M047033 | 8.619268417 |
| PLOD1 | Procollagen-Lysine,2-Oxoglutarate 5-Dioxygenase 1 | GC01P011934 | 8.618924141 |
| PAX6 | Paired Box 6 | GC11M031784 | 8.610114098 |
| CALCA | Calcitonin Related Polypeptide Alpha | GC11M014966 | 8.607903481 |
| PDXK | Pyridoxal Kinase | GC21P043719 | 8.604496002 |
| ESR2 | Estrogen Receptor 2 | GC14M064084 | 8.602386475 |
| SOAT1 | Sterol O-Acyltransferase 1 | GC01P179309 | 8.585945129 |
| ENO1 | Enolase 1 | GC01M008861 | 8.583614349 |
| PCSK1 | Proprotein Convertase Subtilisin/Kexin Type 1 | GC05M096391 | 8.582565308 |
| ATP1A2 | ATPase Na+/K+ Transporting Subunit Alpha 2 | GC01P160115 | 8.581669807 |
| NIT2 | Nitrilase Family Member 2 | GC03P100334 | 8.576919556 |
| OPRM1 | Opioid Receptor Mu 1 | GC06P174892 | 8.574793816 |
| ALDH3A1 | Aldehyde Dehydrogenase 3 Family Member A1 | GC17M019737 | 8.56739521 |
| ATG7 | Autophagy Related 7 | GC03P025586 | 8.566742897 |
| XBP1 | X-Box Binding Protein 1 | GC22M028794 | 8.556726456 |
| PHEX | Phosphate Regulating Endopeptidase X-Linked | GC0XP022032 | 8.546815872 |
| PTF1A | Pancreas Associated Transcription Factor 1a | GC10P023194 | 8.540128708 |
| SMAD2 | SMAD Family Member 2 | GC18M047809 | 8.538376808 |
| THBD | Thrombomodulin | GC20M023026 | 8.529045105 |
| PRKACG | Protein Kinase CAMP-Activated Catalytic Subunit Gamma | GC09M071741 | 8.52439785 |
| VTN | Vitronectin | GC17M092926 | 8.514310837 |
| ATP5F1B | ATP Synthase F1 Subunit Beta | GC12M060145 | 8.509048462 |
| IL4 | Interleukin 4 | GC05P132673 | 8.499679565 |
| IREB2 | Iron Responsive Element Binding Protein 2 | GC15P078437 | 8.497420311 |
| GAST | Gastrin | GC17P041712 | 8.479734421 |
| TXNRD1 | Thioredoxin Reductase 1 | GC12P104215 | 8.478024483 |
| SLC3A2 | Solute Carrier Family 3 Member 2 | GC11P062856 | 8.469745636 |
| SLC7A11 | Solute Carrier Family 7 Member 11 | GC04M138164 | 8.469064713 |
| CBL | Cbl Proto-Oncogene | GC11P119206 | 8.466121674 |
| RANBP2 | RAN Binding Protein 2 | GC02P108719 | 8.455323219 |
| STAT2 | Signal Transducer And Activator Of Transcription 2 | GC12M056341 | 8.450799942 |
| MT-TV | Mitochondrially Encoded TRNA-Val (GUN) | GCMTP001605 | 8.450724602 |
| PLA2G2A | Phospholipase A2 Group IIA | GC01M019975 | 8.447377205 |
| PCYT2 | Phosphate Cytidylyltransferase 2, Ethanolamine | GC17M081900 | 8.444787025 |
| ALPP | Alkaline Phosphatase, Placental | GC02P232378 | 8.442931175 |
| HNRNPU | Heterogeneous Nuclear Ribonucleoprotein U | GC01M244844 | 8.436237335 |
| GCGR | Glucagon Receptor | GC17P081804 | 8.419469833 |
| CISD2 | CDGSH Iron Sulfur Domain 2 | GC04P102868 | 8.416184425 |
| ATRX | ATRX Chromatin Remodeler | GC0XM077504 | 8.404655457 |
| FOXA2 | Forkhead Box A2 | GC20M022581 | 8.403936386 |
| IVL | Involucrin | GC01P173311 | 8.393100739 |
| UGT1A5 | UDP Glucuronosyltransferase Family 1 Member A5 | GC02P233712 | 8.389980316 |
| RXRA | Retinoid X Receptor Alpha | GC09P134317 | 8.385263443 |
| MYH3 | Myosin Heavy Chain 3 | GC17M010628 | 8.385016441 |
| MT-TC | Mitochondrially Encoded TRNA-Cys (UGU/C) | GCMTM005763 | 8.384670258 |
| NONO | Non-POU Domain Containing Octamer Binding | GC0XP071274 | 8.384373665 |
| MIR23A | MicroRNA 23a | GC19M104477 | 8.384336472 |
| NSD1 | Nuclear Receptor Binding SET Domain Protein 1 | GC05P189455 | 8.376207352 |
| HSD3B1 | Hydroxy-Delta-5-Steroid Dehydrogenase, 3 Beta- And Steroid Delta-Isomerase 1 | GC01P119507 | 8.37441349 |
| GRIN2B | Glutamate Ionotropic Receptor NMDA Type Subunit 2B | GC12M013437 | 8.367534637 |
| FAM111A | FAM111 Trypsin Like Peptidase A | GC11P059142 | 8.35185051 |
| ITIH4 | Inter-Alpha-Trypsin Inhibitor Heavy Chain 4 | GC03M052812 | 8.344554901 |
| IL17A | Interleukin 17A | GC06P052186 | 8.338589668 |
| PRRT2 | Proline Rich Transmembrane Protein 2 | GC16P029811 | 8.33594799 |
| IGF2BP2 | Insulin Like Growth Factor 2 MRNA Binding Protein 2 | GC03M185643 | 8.335947037 |
| RIMKLB | Ribosomal Modification Protein RimK Like Family Member B | GC12P068380 | 8.317174911 |
| ACSM2B | Acyl-CoA Synthetase Medium Chain Family Member 2B | GC16M047829 | 8.314609528 |
| HACL1 | 2-Hydroxyacyl-CoA Lyase 1 | GC03M028112 | 8.314120293 |
| CACNA1D | Calcium Voltage-Gated Channel Subunit Alpha1 D | GC03P053328 | 8.312785149 |
| ATP1A3 | ATPase Na+/K+ Transporting Subunit Alpha 3 | GC19M041966 | 8.30006218 |
| ALOX15 | Arachidonate 15-Lipoxygenase | GC17M004630 | 8.295314789 |
| ATP5PO | ATP Synthase Peripheral Stalk Subunit OSCP | GC21M033904 | 8.295099258 |
| PTGS1 | Prostaglandin-Endoperoxide Synthase 1 | GC09P122370 | 8.293404579 |
| EOGT | EGF Domain Specific O-Linked N-Acetylglucosamine Transferase | GC03M068975 | 8.291427612 |
| GPD2 | Glycerol-3-Phosphate Dehydrogenase 2 | GC02P156408 | 8.276306152 |
| ENO2 | Enolase 2 | GC12P006913 | 8.271967888 |
| VIP | Vasoactive Intestinal Peptide | GC06P152750 | 8.257572174 |
| FOS | Fos Proto-Oncogene, AP-1 Transcription Factor Subunit | GC14P075278 | 8.256561279 |
| SELP | Selectin P | GC01M169558 | 8.254713058 |
| SLC12A2 | Solute Carrier Family 12 Member 2 | GC05P128083 | 8.253222466 |
| PNKD | PNKD Metallo-Beta-Lactamase Domain Containing | GC02P218270 | 8.251700401 |
| MIR145 | MicroRNA 145 | GC05P149430 | 8.223443031 |
| EDEM3 | ER Degradation Enhancing Alpha-Mannosidase Like Protein 3 | GC01M184690 | 8.222463608 |
| SOX9 | SRY-Box Transcription Factor 9 | GC17P072121 | 8.217172623 |
| TRIM37 | Tripartite Motif Containing 37 | GC17M093936 | 8.212416649 |
| CSF2 | Colony Stimulating Factor 2 | GC05P132073 | 8.209000587 |
| HIP1 | Huntingtin Interacting Protein 1 | GC07M075533 | 8.208332062 |
| KCNH2 | Potassium Voltage-Gated Channel Subfamily H Member 2 | GC07M150944 | 8.203716278 |
| COA7 | Cytochrome C Oxidase Assembly Factor 7 | GC01M052684 | 8.200410843 |
| PDK2 | Pyruvate Dehydrogenase Kinase 2 | GC17P144738 | 8.196218491 |
| MMP9 | Matrix Metallopeptidase 9 | GC20P046008 | 8.184925079 |
| SYNJ1 | Synaptojanin 1 | GC21M032628 | 8.179420471 |
| ADCY10 | Adenylate Cyclase 10 | GC01M167809 | 8.178582191 |
| GLRA1 | Glycine Receptor Alpha 1 | GC05M151822 | 8.173946381 |
| OXT | Oxytocin/Neurophysin I Prepropeptide | GC20P010170 | 8.166658401 |
| NEAT1 | Nuclear Paraspeckle Assembly Transcript 1 | GC11P103328 | 8.160253525 |
| ANTXR2 | ANTXR Cell Adhesion Molecule 2 | GC04M079901 | 8.155888557 |
| SMARCA4 | SWI/SNF Related BAF Chromatin Remodeling Complex Subunit ATPase 4 | GC19P142240 | 8.148674965 |
| CASP1 | Caspase 1 | GC11M105025 | 8.148670197 |
| HPGD | 15-Hydroxyprostaglandin Dehydrogenase | GC04M174490 | 8.133262634 |
| PYGB | Glycogen Phosphorylase B | GC20P025248 | 8.124546051 |
| SLC38A7 | Solute Carrier Family 38 Member 7 | GC16M058665 | 8.121731758 |
| RYR2 | Ryanodine Receptor 2 | GC01P237042 | 8.107397079 |
| ATP1A1 | ATPase Na+/K+ Transporting Subunit Alpha 1 | GC01P116372 | 8.106987 |
| DSP | Desmoplakin | GC06P007541 | 8.104977608 |
| CUL4B | Cullin 4B | GC0XM120524 | 8.102921486 |
| SCN2A | Sodium Voltage-Gated Channel Alpha Subunit 2 | GC02P165194 | 8.101264954 |
| CEP290 | Centrosomal Protein 290 | GC12M088049 | 8.100214005 |
| MAPK14 | Mitogen-Activated Protein Kinase 14 | GC06P173199 | 8.096892357 |
| HMGA1 | High Mobility Group AT-Hook 1 | GC06P173175 | 8.093466759 |
| CTBP1 | C-Terminal Binding Protein 1 | GC04M001211 | 8.086405754 |
| MYBPC3 | Myosin Binding Protein C3 | GC11M136236 | 8.086271286 |
| TYRP1 | Tyrosinase Related Protein 1 | GC09P012683 | 8.083860397 |
| FABP5 | Fatty Acid Binding Protein 5 | GC08P081282 | 8.0804739 |
| CDK2 | Cyclin Dependent Kinase 2 | GC12P055966 | 8.078052521 |
| SLC38A9 | Solute Carrier Family 38 Member 9 | GC05M055625 | 8.07277298 |
| HSD17B13 | Hydroxysteroid 17-Beta Dehydrogenase 13 | GC04M087303 | 8.069081306 |
| KCNQ2 | Potassium Voltage-Gated Channel Subfamily Q Member 2 | GC20M063400 | 8.049947739 |
| DNM3 | Dynamin 3 | GC01P174456 | 8.048723221 |
| MTHFD2 | Methylenetetrahydrofolate Dehydrogenase (NADP+ Dependent) 2, Methenyltetrahydrofolate Cyclohydrolase | GC02P074186 | 8.046760559 |
| MT-TA | Mitochondrially Encoded TRNA-Ala (GCN) | GCMTM005589 | 8.046584129 |
| TBC1D4 | TBC1 Domain Family Member 4 | GC13M075284 | 8.044921875 |
| PER2 | Period Circadian Regulator 2 | GC02M238244 | 8.044244766 |
| SULT2A1 | Sulfotransferase Family 2A Member 1 | GC19M047870 | 8.041877747 |
| KAT5 | Lysine Acetyltransferase 5 | GC11P065711 | 8.034737587 |
| DUOX2 | Dual Oxidase 2 | GC15M045092 | 8.025200844 |
| DAG1 | Dystroglycan 1 | GC03P063215 | 8.01982975 |
| BECN1 | Beclin 1 | GC17M042810 | 8.015447617 |
| MEFV | MEFV Innate Immunity Regulator, Pyrin | GC16M046510 | 8.014885902 |
| IL4R | Interleukin 4 Receptor | GC16P112904 | 8.014816284 |
| GAD2 | Glutamate Decarboxylase 2 | GC10P026216 | 8.010079384 |
| GNAO1 | G Protein Subunit Alpha O1 | GC16P113698 | 8.010019302 |
| ITPR1 | Inositol 1,4,5-Trisphosphate Receptor Type 1 | GC03P004495 | 8.007608414 |
| RIMKLA | Ribosomal Modification Protein RimK Like Family Member A | GC01P042380 | 8.001731873 |
| DLST | Dihydrolipoamide S-Succinyltransferase | GC14P074881 | 7.983827114 |
| SLC26A4 | Solute Carrier Family 26 Member 4 | GC07P107660 | 7.972998142 |
| YY1 | YY1 Transcription Factor | GC14P100238 | 7.970047951 |
| ACHE | Acetylcholinesterase (Yt Blood Group) | GC07M100889 | 7.968139648 |
| FBP2 | Fructose-Bisphosphatase 2 | GC09M094558 | 7.962796211 |
| MLX | MAX Dimerization Protein MLX | GC17P042567 | 7.962445259 |
| CSNK2B | Casein Kinase 2 Beta | GC06P173083 | 7.961890221 |
| HDAC4 | Histone Deacetylase 4 | GC02M239048 | 7.961318016 |
| TUBB4A | Tubulin Beta 4A Class IVa | GC19M104185 | 7.958325863 |
| TRE-TTC3-1 | TRNA-Glu (Anticodon TTC) 3-1 | GC01P068562 | 7.955482006 |
| TUBA1A | Tubulin Alpha 1a | GC12M049184 | 7.941314697 |
| PPP2CA | Protein Phosphatase 2 Catalytic Subunit Alpha | GC05M134194 | 7.940159798 |
| TGFBR1 | Transforming Growth Factor Beta Receptor 1 | GC09P105622 | 7.939293861 |
| MMP2 | Matrix Metallopeptidase 2 | GC16P113682 | 7.93747139 |
| GATA3 | GATA Binding Protein 3 | GC10P008045 | 7.920713425 |
| AQP2 | Aquaporin 2 | GC12P049950 | 7.9124856 |
| CHERP | Calcium Homeostasis Endoplasmic Reticulum Protein | GC19M016517 | 7.91006422 |
| CKMT2 | Creatine Kinase, Mitochondrial 2 | GC05P081233 | 7.898715973 |
| LRRK2 | Leucine Rich Repeat Kinase 2 | GC12P040196 | 7.896663189 |
| SNORD15A | Small Nucleolar RNA, C/D Box 15A | GC11P075400 | 7.872387409 |
| RXYLT1 | Ribitol Xylosyltransferase 1 | GC12P063779 | 7.871010303 |
| RELA | RELA Proto-Oncogene, NF-KB Subunit | GC11M065653 | 7.865684509 |
| GAS5 | Growth Arrest Specific 5 | GC01M175098 | 7.850930214 |
| PRKAG1 | Protein Kinase AMP-Activated Non-Catalytic Subunit Gamma 1 | GC12M049002 | 7.847611427 |
| NOTCH3 | Notch Receptor 3 | GC19M015159 | 7.847205639 |
| BAX | BCL2 Associated X, Apoptosis Regulator | GC19P048954 | 7.844941616 |
| DSG1 | Desmoglein 1 | GC18P031318 | 7.837490082 |
| SPAST | Spastin | GC02P032063 | 7.834443092 |
| IL5 | Interleukin 5 | GC05M132541 | 7.834085464 |
| TOP1 | DNA Topoisomerase I | GC20P041028 | 7.816466331 |
| BMP4 | Bone Morphogenetic Protein 4 | GC14M053949 | 7.804695129 |
| BLVRB | Biliverdin Reductase B | GC19M040447 | 7.804096222 |
| EIF2B4 | Eukaryotic Translation Initiation Factor 2B Subunit Delta | GC02M027364 | 7.798873425 |
| FOLH1 | Folate Hydrolase 1 | GC11M136261 | 7.79512167 |
| MEN1 | Menin 1 | GC11M064803 | 7.794550896 |
| LMNB1 | Lamin B1 | GC05P126776 | 7.790050983 |
| PM20D1 | Peptidase M20 Domain Containing 1 | GC01M205829 | 7.788228035 |
| COL18A1 | Collagen Type XVIII Alpha 1 Chain | GC21P045405 | 7.786314011 |
| MT-TM | Mitochondrially Encoded TRNA-Met (AUA/G) | GCMTP004404 | 7.780203819 |
| IL2 | Interleukin 2 | GC04M122451 | 7.776171684 |
| ME2 | Malic Enzyme 2 | GC18P050879 | 7.771995544 |
| PGM2L1 | Phosphoglucomutase 2 Like 1 | GC11M074330 | 7.759802341 |
| IFIH1 | Interferon Induced With Helicase C Domain 1 | GC02M162267 | 7.751023293 |
| CKMT1B | Creatine Kinase, Mitochondrial 1B | GC15P184191 | 7.743916512 |
| NTRK2 | Neurotrophic Receptor Tyrosine Kinase 2 | GC09P084668 | 7.734851837 |
| MYCN | MYCN Proto-Oncogene, BHLH Transcription Factor | GC02P015974 | 7.722681046 |
| ALDH1A2 | Aldehyde Dehydrogenase 1 Family Member A2 | GC15M158963 | 7.720340252 |
| SELENOI | Selenoprotein I | GC02P029242 | 7.718239307 |
| RAB6A | RAB6A, Member RAS Oncogene Family | GC11M136812 | 7.708667755 |
| S100B | S100 Calcium Binding Protein B | GC21M054609 | 7.707233429 |
| MIR142 | MicroRNA 142 | GC17M058331 | 7.700267792 |
| UBE3A | Ubiquitin Protein Ligase E3A | GC15M025333 | 7.698262215 |
| ARID1A | AT-Rich Interaction Domain 1A | GC01P026693 | 7.696621895 |
| EIF2AK2 | Eukaryotic Translation Initiation Factor 2 Alpha Kinase 2 | GC02M037099 | 7.688798904 |
| MTHFD1L | Methylenetetrahydrofolate Dehydrogenase (NADP+ Dependent) 1 Like | GC06P150865 | 7.683426857 |
| MIR200A | MicroRNA 200a | GC01P067742 | 7.670555115 |
| GATA1 | GATA Binding Protein 1 | GC0XP048786 | 7.665660858 |
| U2AF2 | U2 Small Nuclear RNA Auxiliary Factor 2 | GC19P055654 | 7.660038471 |
| TOP2A | DNA Topoisomerase II Alpha | GC17M040388 | 7.659296989 |
| BTK | Bruton Tyrosine Kinase | GC0XM101349 | 7.657463551 |
| SIRT2 | Sirtuin 2 | GC19M038878 | 7.655106544 |
| CEBPA | CCAAT Enhancer Binding Protein Alpha | GC19M033299 | 7.653105259 |
| ADAMTSL2 | ADAMTS Like 2 | GC09P150060 | 7.651970863 |
| YWHAE | Tyrosine 3-Monooxygenase/Tryptophan 5-Monooxygenase Activation Protein Epsilon | GC17M092123 | 7.651399136 |
| RUNX2 | RUNX Family Transcription Factor 2 | GC06P173274 | 7.64777565 |
| ICAM1 | Intercellular Adhesion Molecule 1 | GC19P142230 | 7.643219948 |
| TNXB | Tenascin XB | GC06M103361 | 7.629268646 |
| USF1 | Upstream Transcription Factor 1 | GC01M161039 | 7.623073578 |
| KCNJ2 | Potassium Inwardly Rectifying Channel Subfamily J Member 2 | GC17P070168 | 7.621566296 |
| SELE | Selectin E | GC01M169722 | 7.619717121 |
| ATXN10 | Ataxin 10 | GC22P087139 | 7.612990379 |
| PTK2 | Protein Tyrosine Kinase 2 | GC08M140657 | 7.608383179 |
| VPS13A | Vacuolar Protein Sorting 13 Homolog A | GC09P077177 | 7.596244812 |
| MIR146A | MicroRNA 146a | GC05P160485 | 7.595888138 |
| TAF1 | TATA-Box Binding Protein Associated Factor 1 | GC0XP071366 | 7.594488144 |
| CSNK2A1 | Casein Kinase 2 Alpha 1 | GC20M000472 | 7.592903137 |
| SORT1 | Sortilin 1 | GC01M109310 | 7.589745998 |
| PFKP | Phosphofructokinase, Platelet | GC10P003066 | 7.589072227 |
| PDE4D | Phosphodiesterase 4D | GC05M058969 | 7.58735323 |
| H3-3B | H3.3 Histone B | GC17M094293 | 7.587078571 |
| HSPA9 | Heat Shock Protein Family A (Hsp70) Member 9 | GC05M138554 | 7.584821701 |
| PDE3B | Phosphodiesterase 3B | GC11P014643 | 7.580919743 |
| MT-TF | Mitochondrially Encoded TRNA-Phe (UUU/C) | GCMTP000580 | 7.571543217 |
| MMP1 | Matrix Metallopeptidase 1 | GC11M137190 | 7.563008785 |
| NTS | Neurotensin | GC12P085876 | 7.558875561 |
| CDC42 | Cell Division Cycle 42 | GC01P022052 | 7.558388233 |
| ADPRH | ADP-Ribosylarginine Hydrolase | GC03P119579 | 7.557754517 |
| DLG4 | Discs Large MAGUK Scaffold Protein 4 | GC17M092409 | 7.557167053 |
| PNKP | Polynucleotide Kinase 3'-Phosphatase | GC19M105287 | 7.540770531 |
| VCAM1 | Vascular Cell Adhesion Molecule 1 | GC01P100719 | 7.538982391 |
| SERPINF2 | Serpin Family F Member 2 | GC17P001742 | 7.53416729 |
| MT-TY | Mitochondrially Encoded TRNA-Tyr (UAU/C) | GCMTM005828 | 7.529373646 |
| FOXP3 | Forkhead Box P3 | GC0XM049250 | 7.526286602 |
| ATP6V1B2 | ATPase H+ Transporting V1 Subunit B2 | GC08P020197 | 7.522710323 |
| MIR532 | MicroRNA 532 | GC0XP060165 | 7.513988972 |
| ANPEP | Alanyl Aminopeptidase, Membrane | GC15M089784 | 7.512180805 |
| ACSM1 | Acyl-CoA Synthetase Medium Chain Family Member 1 | GC16M047832 | 7.512040615 |
| NAT8L | N-Acetyltransferase 8 Like | GC04P009317 | 7.510066986 |
| TGFA | Transforming Growth Factor Alpha | GC02M070447 | 7.503126144 |
| OGG1 | 8-Oxoguanine DNA Glycosylase | GC03P025530 | 7.502356052 |
| GNB1 | G Protein Subunit Beta 1 | GC01M001785 | 7.501042366 |
| GRP | Gastrin Releasing Peptide | GC18P059220 | 7.492141724 |
| SOST | Sclerostin | GC17M043753 | 7.490190029 |
| RNF216 | Ring Finger Protein 216 | GC07M005620 | 7.484732628 |
| NKX2-1 | NK2 Homeobox 1 | GC14M036516 | 7.484641552 |
| PPP2R1A | Protein Phosphatase 2 Scaffold Subunit Aalpha | GC19P143427 | 7.483341217 |
| SLC20A2 | Solute Carrier Family 20 Member 2 | GC08M042416 | 7.467317104 |
| ATP2A2 | ATPase Sarcoplasmic/Endoplasmic Reticulum Ca2+ Transporting 2 | GC12P110280 | 7.460154057 |
| TUBA1B | Tubulin Alpha 1b | GC12M049127 | 7.456658363 |
| HNRNPK | Heterogeneous Nuclear Ribonucleoprotein K | GC09M119624 | 7.451663494 |
| DES | Desmin | GC02P219418 | 7.444146156 |
| SLC15A1 | Solute Carrier Family 15 Member 1 | GC13M098683 | 7.438258171 |
| PTPRC | Protein Tyrosine Phosphatase Receptor Type C | GC01P198607 | 7.437558651 |
| GAL | Galanin And GMAP Prepropeptide | GC11P103539 | 7.430492401 |
| MSTN | Myostatin | GC02M190055 | 7.429765224 |
| MT-TD | Mitochondrially Encoded TRNA-Asp (GAU/C) | GCMTP007520 | 7.425289154 |
| ADD1 | Adducin 1 | GC04P009396 | 7.414868355 |
| DGKD | Diacylglycerol Kinase Delta | GC02P234613 | 7.414766788 |
| GGCX | Gamma-Glutamyl Carboxylase | GC02M085544 | 7.409678936 |
| GNAI1 | G Protein Subunit Alpha I1 | GC07P093152 | 7.405898094 |
| HMGB1 | High Mobility Group Box 1 | GC13M030456 | 7.404634476 |
| TNFSF10 | TNF Superfamily Member 10 | GC03M172505 | 7.394180298 |
| UBC | Ubiquitin C | GC12M124911 | 7.373379707 |
| TRMT112 | TRNA Methyltransferase Activator Subunit 11-2 | GC11M064316 | 7.364256859 |
| CDKAL1 | CDK5 Regulatory Subunit Associated Protein 1 Like 1 | GC06P020534 | 7.359016418 |
| LCN2 | Lipocalin 2 | GC09P128149 | 7.341784954 |
| CDKN2A | Cyclin Dependent Kinase Inhibitor 2A | GC09M021967 | 7.340119362 |
| NRAS | NRAS Proto-Oncogene, GTPase | GC01M114704 | 7.339721203 |
| HDAC3 | Histone Deacetylase 3 | GC05M141620 | 7.335883617 |
| ERCC1 | ERCC Excision Repair 1, Endonuclease Non-Catalytic Subunit | GC19M105114 | 7.333277702 |
| NOTCH1 | Notch Receptor 1 | GC09M139117 | 7.328620911 |
| LMNB2 | Lamin B2 | GC19M104058 | 7.32734251 |
| SDHC | Succinate Dehydrogenase Complex Subunit C | GC01P161314 | 7.326922894 |
| KCND3 | Potassium Voltage-Gated Channel Subfamily D Member 3 | GC01M111770 | 7.321941853 |
| NPPB | Natriuretic Peptide B | GC01M020959 | 7.318058014 |
| ANOS1 | Anosmin 1 | GC0XM008528 | 7.316493988 |
| ALDH1L1 | Aldehyde Dehydrogenase 1 Family Member L1 | GC03M126103 | 7.314570427 |
| CNTNAP2 | Contactin Associated Protein 2 | GC07P146116 | 7.312737942 |
| PDGFRB | Platelet Derived Growth Factor Receptor Beta | GC05M150113 | 7.297045231 |
| APOH | Apolipoprotein H | GC17M066212 | 7.295294285 |
| PABPC1 | Poly(A) Binding Protein Cytoplasmic 1 | GC08M100685 | 7.295075417 |
| GSTO1 | Glutathione S-Transferase Omega 1 | GC10P104235 | 7.28857851 |
| AVPR2 | Arginine Vasopressin Receptor 2 | GC0XP153902 | 7.287927151 |
| ME1 | Malic Enzyme 1 | GC06M083210 | 7.28234148 |
| RAC1 | Rac Family Small GTPase 1 | GC07P017475 | 7.278645515 |
| VHL | Von Hippel-Lindau Tumor Suppressor | GC03P025565 | 7.276751518 |
| PHOX2B | Paired Like Homeobox 2B | GC04M041746 | 7.274650574 |
| HSP90B1 | Heat Shock Protein 90 Beta Family Member 1 | GC12P103930 | 7.27442646 |
| ERCC8 | ERCC Excision Repair 8, CSA Ubiquitin Ligase Complex Subunit | GC05M060919 | 7.259871006 |
| TFEB | Transcription Factor EB | GC06M103530 | 7.255251884 |
| ACSL5 | Acyl-CoA Synthetase Long Chain Family Member 5 | GC10P112374 | 7.250842094 |
| PRKG1 | Protein Kinase CGMP-Dependent 1 | GC10P052081 | 7.246520996 |
| DDX3X | DEAD-Box Helicase 3 X-Linked | GC0XP041333 | 7.246436119 |
| USP7 | Ubiquitin Specific Peptidase 7 | GC16M008892 | 7.243258476 |
| GPLD1 | Glycosylphosphatidylinositol Specific Phospholipase D1 | GC06M024566 | 7.239953518 |
| SLC26A3 | Solute Carrier Family 26 Member 3 | GC07M107765 | 7.231235504 |
| FCGR2A | Fc Gamma Receptor IIa | GC01P161505 | 7.228515625 |
| PLAU | Plasminogen Activator, Urokinase | GC10P073909 | 7.222958565 |
| SULT1A3 | Sulfotransferase Family 1A Member 3 | GC16P030199 | 7.217173576 |
| FOXP1 | Forkhead Box P1 | GC03M070954 | 7.216155052 |
| CD14 | CD14 Molecule | GC05M140631 | 7.21421957 |
| SUMO1 | Small Ubiquitin Like Modifier 1 | GC02M202206 | 7.206022263 |
| CASK | Calcium/Calmodulin Dependent Serine Protein Kinase | GC0XM041514 | 7.202850819 |
| ADH5 | Alcohol Dehydrogenase 5 (Class III), Chi Polypeptide | GC04M099070 | 7.202615261 |
| CD8A | CD8 Subunit Alpha | GC02M086784 | 7.192557812 |
| PROP1 | PROP Paired-Like Homeobox 1 | GC05M177992 | 7.181645393 |
| EZH2 | Enhancer Of Zeste 2 Polycomb Repressive Complex 2 Subunit | GC07M148807 | 7.179532051 |
| PVT1 | Pvt1 Oncogene | GC08P128310 | 7.166604042 |
| CDK6 | Cyclin Dependent Kinase 6 | GC07M092604 | 7.161592484 |
| RPS6KA3 | Ribosomal Protein S6 Kinase A3 | GC0XM020149 | 7.141894341 |
| CSF1R | Colony Stimulating Factor 1 Receptor | GC05M150053 | 7.138968945 |
| TUBB3 | Tubulin Beta 3 Class III | GC16P115082 | 7.138869762 |
| CDKN3 | Cyclin Dependent Kinase Inhibitor 3 | GC14P055603 | 7.133357048 |
| SULT1E1 | Sulfotransferase Family 1E Member 1 | GC04M069823 | 7.13312149 |
| PAICS | Phosphoribosylaminoimidazole Carboxylase And Phosphoribosylaminoimidazolesuccinocarboxamide Synthase | GC04P056435 | 7.128750801 |
| LGALS1 | Galectin 1 | GC22P037675 | 7.123792648 |
| PRPF8 | Pre-MRNA Processing Factor 8 | GC17M001650 | 7.117331505 |
| F3 | Coagulation Factor III, Tissue Factor | GC01M094997 | 7.105412483 |
| PRTN3 | Proteinase 3 | GC19P000840 | 7.100646019 |
| P4HB | Prolyl 4-Hydroxylase Subunit Beta | GC17M081843 | 7.096831799 |
| CYB5A | Cytochrome B5 Type A | GC18M074250 | 7.09677124 |
| DYRK1A | Dual Specificity Tyrosine Phosphorylation Regulated Kinase 1A | GC21P037365 | 7.081241608 |
| CCR5 | C-C Motif Chemokine Receptor 5 | GC03P063059 | 7.081235886 |
| SIRT5 | Sirtuin 5 | GC06P013574 | 7.080095291 |
| CD40 | CD40 Molecule | GC20P046118 | 7.077231884 |
| CLTRN | Collectrin, Amino Acid Transport Regulator | GC0XM016042 | 7.076663971 |
| RAB27A | RAB27A, Member RAS Oncogene Family | GC15M055202 | 7.073971748 |
| HUWE1 | HECT, UBA And WWE Domain Containing E3 Ubiquitin Protein Ligase 1 | GC0XM053532 | 7.064956665 |
| TAF4 | TATA-Box Binding Protein Associated Factor 4 | GC20M062012 | 7.056445122 |
| DNM1 | Dynamin 1 | GC09P128191 | 7.050777435 |
| FURIN | Furin, Paired Basic Amino Acid Cleaving Enzyme | GC15P090868 | 7.045512199 |
| TCERG1 | Transcription Elongation Regulator 1 | GC05P146447 | 7.045483589 |
| ITPR3 | Inositol 1,4,5-Trisphosphate Receptor Type 3 | GC06P033620 | 7.040857792 |
| RAG1 | Recombination Activating 1 | GC11P036554 | 7.040530205 |
| CRBN | Cereblon | GC03M003144 | 7.006428719 |
| OGDHL | Oxoglutarate Dehydrogenase L | GC10M049734 | 7.001672745 |
| NOX4 | NADPH Oxidase 4 | GC11M089324 | 7.001053333 |
| SCN1B | Sodium Voltage-Gated Channel Beta Subunit 1 | GC19P035030 | 6.999047279 |
| THBS1 | Thrombospondin 1 | GC15P039581 | 6.993292332 |
| SPTBN1 | Spectrin Beta, Non-Erythrocytic 1 | GC02P054456 | 6.990820885 |
| CDKN1B | Cyclin Dependent Kinase Inhibitor 1B | GC12P068529 | 6.99069643 |
| NOTCH2 | Notch Receptor 2 | GC01M119911 | 6.979052544 |
| FGA | Fibrinogen Alpha Chain | GC04M154583 | 6.978610992 |
| EPAS1 | Endothelial PAS Domain Protein 1 | GC02P046293 | 6.977585316 |
| SIRT7 | Sirtuin 7 | GC17M081911 | 6.974484444 |
| BACE1 | Beta-Secretase 1 | GC11M117285 | 6.973061562 |
| TOP3A | DNA Topoisomerase III Alpha | GC17M018271 | 6.969982147 |
| SMN2 | Survival Of Motor Neuron 2, Centromeric | GC05P070049 | 6.966377735 |
| SNRPN | Small Nuclear Ribonucleoprotein Polypeptide N | GC15P024823 | 6.960752487 |
| SPHK1 | Sphingosine Kinase 1 | GC17P076376 | 6.956059933 |
| ST3GAL4 | ST3 Beta-Galactoside Alpha-2,3-Sialyltransferase 4 | GC11P126355 | 6.952857018 |
| SMPD2 | Sphingomyelin Phosphodiesterase 2 | GC06P109440 | 6.94627142 |
| SLC30A8 | Solute Carrier Family 30 Member 8 | GC08P116950 | 6.944516659 |
| TGFBI | Transforming Growth Factor Beta Induced | GC05P136027 | 6.938262939 |
| FABP12 | Fatty Acid Binding Protein 12 | GC08M081524 | 6.938121796 |
| PML | PML Nuclear Body Scaffold | GC15P073994 | 6.933957577 |
| PPP1CA | Protein Phosphatase 1 Catalytic Subunit Alpha | GC11M136656 | 6.932367325 |
| GATA4 | GATA Binding Protein 4 | GC08P011676 | 6.928163052 |
| SCN10A | Sodium Voltage-Gated Channel Alpha Subunit 10 | GC03M038813 | 6.927970886 |
| PAX3 | Paired Box 3 | GC02M222199 | 6.926390648 |
| MIR330 | MicroRNA 330 | GC19M105128 | 6.921786308 |
| GART | Phosphoribosylglycinamide Formyltransferase, Phosphoribosylglycinamide Synthetase, Phosphoribosylaminoimidazole Synthetase | GC21M033503 | 6.921758652 |
| MIR451A | MicroRNA 451a | GC17M028861 | 6.910225391 |
| STAT1 | Signal Transducer And Activator Of Transcription 1 | GC02M190908 | 6.900700569 |
| SCG5 | Secretogranin V | GC15P032641 | 6.892454624 |
| SMC1A | Structural Maintenance Of Chromosomes 1A | GC0XM053374 | 6.889140606 |
| AVP | Arginine Vasopressin | GC20M003082 | 6.887694836 |
| ATF6 | Activating Transcription Factor 6 | GC01P161766 | 6.871023178 |
| HSP90AB1 | Heat Shock Protein 90 Alpha Family Class B Member 1 | GC06P044246 | 6.869912148 |
| ADRA2B | Adrenoceptor Alpha 2B | GC02M096112 | 6.867357254 |
| GLYATL1B | Glycine-N-Acyltransferase Like 1B | GC11P059086 | 6.859425068 |
| RPGR | Retinitis Pigmentosa GTPase Regulator | GC0XM038269 | 6.857671738 |
| TNFRSF1A | TNF Receptor Superfamily Member 1A | GC12M006328 | 6.855262756 |
| SIN3A | SIN3 Transcription Regulator Family Member A | GC15M075369 | 6.854660511 |
| NR2F2 | Nuclear Receptor Subfamily 2 Group F Member 2 | GC15P096325 | 6.850878716 |
| TAC1 | Tachykinin Precursor 1 | GC07P097734 | 6.839540005 |
| SNAP25 | Synaptosome Associated Protein 25 | GC20P010516 | 6.831334591 |
| SHH | Sonic Hedgehog Signaling Molecule | GC07M155799 | 6.817792416 |
| PLG | Plasminogen | GC06P160702 | 6.81043005 |
| CCND1 | Cyclin D1 | GC11P069641 | 6.806768894 |
| TUBB2A | Tubulin Beta 2A Class IIa | GC06M003153 | 6.801758289 |
| RRM1 | Ribonucleotide Reductase Catalytic Subunit M1 | GC11P015892 | 6.796823502 |
| CD4 | CD4 Molecule | GC12P006786 | 6.796327114 |
| DNAJB1 | DnaJ Heat Shock Protein Family (Hsp40) Member B1 | GC19M104494 | 6.796224594 |
| DHPS | Deoxyhypusine Synthase | GC19M104435 | 6.786311626 |
| CPQ | Carboxypeptidase Q | GC08P096645 | 6.782552719 |
| PRKAB2 | Protein Kinase AMP-Activated Non-Catalytic Subunit Beta 2 | GC01M147155 | 6.781729698 |
| BAIAP2L1 | BAR/IMD Domain Containing Adaptor Protein 2 Like 1 | GC07M098319 | 6.776586056 |
| RNF2 | Ring Finger Protein 2 | GC01P185045 | 6.763832092 |
| SCN8A | Sodium Voltage-Gated Channel Alpha Subunit 8 | GC12P051590 | 6.755608082 |
| HCN4 | Hyperpolarization Activated Cyclic Nucleotide Gated Potassium Channel 4 | GC15M073319 | 6.753918171 |
| AIP | Aryl Hydrocarbon Receptor Interacting Protein | GC11P067468 | 6.751142502 |
| USP9X | Ubiquitin Specific Peptidase 9 X-Linked | GC0XP041085 | 6.744086266 |
| SCT | Secretin | GC11M000626 | 6.739366531 |
| MET | MET Proto-Oncogene, Receptor Tyrosine Kinase | GC07P116672 | 6.734103203 |
| BAG3 | BAG Cochaperone 3 | GC10P119651 | 6.721960068 |
| ANKH | ANKH Inorganic Pyrophosphate Transport Regulator | GC05M014852 | 6.718658447 |
| H4C16 | H4 Histone 16 | GC12M035008 | 6.718200684 |
| FBL | Fibrillarin | GC19M039834 | 6.710307121 |
| BCR | BCR Activator Of RhoGEF And GTPase | GC22P023179 | 6.710024834 |
| APOM | Apolipoprotein M | GC06P173087 | 6.708650589 |
| PSEN2 | Presenilin 2 | GC01P226870 | 6.68769455 |
| RECQL4 | RecQ Like Helicase 4 | GC08M147210 | 6.676860809 |
| SYNE1 | Spectrin Repeat Containing Nuclear Envelope Protein 1 | GC06M152121 | 6.67519474 |
| TXNRD2 | Thioredoxin Reductase 2 | GC22M083345 | 6.672195435 |
| UCHL1 | Ubiquitin C-Terminal Hydrolase L1 | GC04P041256 | 6.671936035 |
| CSTB | Cystatin B | GC21M043772 | 6.667035103 |
| NCOR1 | Nuclear Receptor Corepressor 1 | GC17M016029 | 6.6620574 |
| CD79A | CD79a Molecule | GC19P041877 | 6.657525539 |
| PDGFB | Platelet Derived Growth Factor Subunit B | GC22M083986 | 6.656937122 |
| YWHAG | Tyrosine 3-Monooxygenase/Tryptophan 5-Monooxygenase Activation Protein Gamma | GC07M080025 | 6.652376175 |
| LALBA | Lactalbumin Alpha | GC12M048567 | 6.643548965 |
| TACR3 | Tachykinin Receptor 3 | GC04M103586 | 6.641368389 |
| BRIP1 | BRCA1 Interacting DNA Helicase 1 | GC17M061679 | 6.640007973 |
| MIR143 | MicroRNA 143 | GC05P157239 | 6.633893967 |
| ERBB2 | Erb-B2 Receptor Tyrosine Kinase 2 | GC17P039687 | 6.630290031 |
| RAPSN | Receptor Associated Protein Of The Synapse | GC11M136239 | 6.63006258 |
| MIR148A | MicroRNA 148a | GC07M025950 | 6.624579906 |
| FBXW7 | F-Box And WD Repeat Domain Containing 7 | GC04M152321 | 6.622961521 |
| SLC4A4 | Solute Carrier Family 4 Member 4 | GC04P071063 | 6.621101379 |
| FRRS1L | Ferric Chelate Reductase 1 Like | GC09M109130 | 6.62055397 |
| ATP4A | ATPase H+/K+ Transporting Subunit Alpha | GC19M105677 | 6.617990971 |
| NARS1 | Asparaginyl-TRNA Synthetase 1 | GC18M057601 | 6.612771034 |
| DCK | Deoxycytidine Kinase | GC04P070992 | 6.608788013 |
| NUP214 | Nucleoporin 214 | GC09P131125 | 6.608379364 |
| UFD1 | Ubiquitin Recognition Factor In ER Associated Degradation 1 | GC22M083337 | 6.606611252 |
| CD44 | CD44 Molecule (IN Blood Group) | GC11P035139 | 6.602041721 |
| HNRNPC | Heterogeneous Nuclear Ribonucleoprotein C | GC14M028270 | 6.601411819 |
| TSHB | Thyroid Stimulating Hormone Subunit Beta | GC01P115029 | 6.599347591 |
| GRIN2D | Glutamate Ionotropic Receptor NMDA Type Subunit 2D | GC19P143257 | 6.597560883 |
| MIR10B | MicroRNA 10b | GC02P176150 | 6.590835571 |
| MIR223 | MicroRNA 223 | GC0XP066018 | 6.590157509 |
| SREK1 | Splicing Regulatory Glutamic Acid And Lysine Rich Protein 1 | GC05P066139 | 6.589156628 |
| TPM1 | Tropomyosin 1 | GC15P192130 | 6.589012623 |
| SAFB | Scaffold Attachment Factor B | GC19P005623 | 6.587001801 |
| MSR1 | Macrophage Scavenger Receptor 1 | GC08M016107 | 6.585490227 |
| NAA10 | N-Alpha-Acetyltransferase 10, NatA Catalytic Subunit | GC0XM153929 | 6.580570221 |
| MYL2 | Myosin Light Chain 2 | GC12M111940 | 6.571843147 |
| CXCR4 | C-X-C Motif Chemokine Receptor 4 | GC02M136114 | 6.566839218 |
| CSF1 | Colony Stimulating Factor 1 | GC01P111495 | 6.555706978 |
| SCN9A | Sodium Voltage-Gated Channel Alpha Subunit 9 | GC02M166195 | 6.55340004 |
| GRIA1 | Glutamate Ionotropic Receptor AMPA Type Subunit 1 | GC05P153489 | 6.550468445 |
| SAMD9 | Sterile Alpha Motif Domain Containing 9 | GC07M093099 | 6.546702385 |
| RPGRIP1L | RPGRIP1 Like | GC16M053786 | 6.54006958 |
| GPBAR1 | G Protein-Coupled Bile Acid Receptor 1 | GC02P218259 | 6.537994385 |
| DNASE1 | Deoxyribonuclease 1 | GC16P003611 | 6.536830902 |
| MIR133B | MicroRNA 133b | GC06P052148 | 6.532292843 |
| SMARCA2 | SWI/SNF Related BAF Chromatin Remodeling Complex Subunit ATPase 2 | GC09P001980 | 6.531588554 |
| ADCY3 | Adenylate Cyclase 3 | GC02M024819 | 6.527186394 |
| ENG | Endoglin | GC09M131239 | 6.524551392 |
| BAIAP2 | BAR/IMD Domain Containing Adaptor Protein 2 | GC17P081035 | 6.522853851 |
| GJB2 | Gap Junction Protein Beta 2 | GC13M020187 | 6.512528896 |
| ANK3 | Ankyrin 3 | GC10M060026 | 6.50707674 |
| TBC1D20 | TBC1 Domain Family Member 20 | GC20M000423 | 6.502242565 |
| TBC1D24 | TBC1 Domain Family Member 24 | GC16P002475 | 6.499353886 |
| MYH14 | Myosin Heavy Chain 14 | GC19P143344 | 6.495202541 |
| TRRAP | Transformation/Transcription Domain Associated Protein | GC07P098877 | 6.494898796 |
| MIR9-1 | MicroRNA 9-1 | GC01M156420 | 6.491605282 |
| CD274 | CD274 Molecule | GC09P005450 | 6.490168095 |
| CHRNA7 | Cholinergic Receptor Nicotinic Alpha 7 Subunit | GC15P031923 | 6.489618301 |
| SLC25A11 | Solute Carrier Family 25 Member 11 | GC17M004937 | 6.482804298 |
| PIGS | Phosphatidylinositol Glycan Anchor Biosynthesis Class S | GC17M028553 | 6.480194092 |
| GNAQ | G Protein Subunit Alpha Q | GC09M077716 | 6.47816658 |
| PRKCA | Protein Kinase C Alpha | GC17P066302 | 6.467977524 |
| SLC6A1 | Solute Carrier Family 6 Member 1 | GC03P025584 | 6.465103626 |
| LACTB | Lactamase Beta | GC15P186086 | 6.451145649 |
| EMD | Emerin | GC0XP154379 | 6.439695835 |
| ACTG1 | Actin Gamma 1 | GC17M081509 | 6.438172817 |
| FGF2 | Fibroblast Growth Factor 2 | GC04P122826 | 6.43799305 |
| CCKAR | Cholecystokinin A Receptor | GC04M026483 | 6.436206818 |
| SRR | Serine Racemase | GC17P002303 | 6.421791553 |
| TERT | Telomerase Reverse Transcriptase | GC05M001253 | 6.419739246 |
| HNRNPL | Heterogeneous Nuclear Ribonucleoprotein L | GC19M038836 | 6.419375896 |
| STIM1 | Stromal Interaction Molecule 1 | GC11P015861 | 6.415051937 |
| SV2A | Synaptic Vesicle Glycoprotein 2A | GC01M149903 | 6.414175034 |
| MIR214 | MicroRNA 214 | GC01M172234 | 6.411346912 |
| MIR144 | MicroRNA 144 | GC17M092933 | 6.40084362 |
| HDC | Histidine Decarboxylase | GC15M050241 | 6.400503635 |
| SLC16A10 | Solute Carrier Family 16 Member 10 | GC06P111087 | 6.399999619 |
| ECM1 | Extracellular Matrix Protein 1 | GC01P150508 | 6.399327278 |
| SLC25A5 | Solute Carrier Family 25 Member 5 | GC0XP119468 | 6.394471645 |
| BRD4 | Bromodomain Containing 4 | GC19M104518 | 6.389134407 |
| GRM5 | Glutamate Metabotropic Receptor 5 | GC11M088504 | 6.383728504 |
| SON | SON DNA And RNA Binding Protein | GC21P033542 | 6.376540184 |
| ALDOC | Aldolase, Fructose-Bisphosphate C | GC17M092928 | 6.372499943 |
| ITGB3 | Integrin Subunit Beta 3 | GC17P144661 | 6.371126652 |
| XK | X-Linked Kx Blood Group Antigen, Kell And VPS13A Binding Protein | GC0XP037685 | 6.370956421 |
| SH3GL3 | SH3 Domain Containing GRB2 Like 3, Endophilin A3 | GC15P083447 | 6.354639053 |
| POGLUT1 | Protein O-Glucosyltransferase 1 | GC03P119468 | 6.351911545 |
| PLAUR | Plasminogen Activator, Urokinase Receptor | GC19M043646 | 6.350399971 |
| TPH2 | Tryptophan Hydroxylase 2 | GC12P071938 | 6.349813461 |
| MYB | MYB Proto-Oncogene, Transcription Factor | GC06P135181 | 6.348267555 |
| ASNSD1 | Asparagine Synthetase Domain Containing 1 | GC02P189661 | 6.341559887 |
| PAFAH1B1 | Platelet Activating Factor Acetylhydrolase 1b Regulatory Subunit 1 | GC17P002593 | 6.335303783 |
| SPTA1 | Spectrin Alpha, Erythrocytic 1 | GC01M158610 | 6.331541061 |
| REEP1 | Receptor Accessory Protein 1 | GC02M086213 | 6.329171181 |
| BCAT1 | Branched Chain Amino Acid Transaminase 1 | GC12M024810 | 6.319494247 |
| RDH11 | Retinol Dehydrogenase 11 | GC14M067676 | 6.317456722 |
| TRAF6 | TNF Receptor Associated Factor 6 | GC11M036467 | 6.310220718 |
| THRB | Thyroid Hormone Receptor Beta | GC03M024117 | 6.303806305 |
| SARS1 | Seryl-TRNA Synthetase 1 | GC01P111321 | 6.302204132 |
| AGFG1 | ArfGAP With FG Repeats 1 | GC02P227473 | 6.298496246 |
| GRM7 | Glutamate Metabotropic Receptor 7 | GC03P006770 | 6.297811985 |
| ITLN1 | Intelectin 1 | GC01M160876 | 6.297304153 |
| EIF4E | Eukaryotic Translation Initiation Factor 4E | GC04M098879 | 6.296720505 |
| SPRY4 | Sprouty RTK Signaling Antagonist 4 | GC05M142310 | 6.294253826 |
| MATR3 | Matrin 3 | GC05P139274 | 6.289617538 |
| AKR1B10 | Aldo-Keto Reductase Family 1 Member B10 | GC07P134527 | 6.286525249 |
| ACVR1 | Activin A Receptor Type 1 | GC02M157736 | 6.283008575 |
| MYH6 | Myosin Heavy Chain 6 | GC14M023381 | 6.277713776 |
| SLC2A5 | Solute Carrier Family 2 Member 5 | GC01M020899 | 6.270421982 |
| GOSR2 | Golgi SNAP Receptor Complex Member 2 | GC17P144629 | 6.269145966 |
| GRM3 | Glutamate Metabotropic Receptor 3 | GC07P086643 | 6.264697075 |
| CLCN1 | Chloride Voltage-Gated Channel 1 | GC07P143316 | 6.262806416 |
| ALOX15B | Arachidonate 15-Lipoxygenase Type B | GC17P008039 | 6.261483192 |
| LHCGR | Luteinizing Hormone/Choriogonadotropin Receptor | GC02M048686 | 6.249456406 |
| CA9 | Carbonic Anhydrase 9 | GC09P035673 | 6.249109745 |
| GABRA1 | Gamma-Aminobutyric Acid Type A Receptor Subunit Alpha1 | GC05P161847 | 6.248141766 |
| PSMC4 | Proteasome 26S Subunit, ATPase 4 | GC19P142936 | 6.245414734 |
| NR5A2 | Nuclear Receptor Subfamily 5 Group A Member 2 | GC01P199996 | 6.242137909 |
| ULK1 | Unc-51 Like Autophagy Activating Kinase 1 | GC12P131894 | 6.240604877 |
| DHRS4 | Dehydrogenase/Reductase 4 | GC14P023953 | 6.233038425 |
| MIR199A1 | MicroRNA 199a-1 | GC19M010817 | 6.227244377 |
| HLA-A | Major Histocompatibility Complex, Class I, A | GC06P173041 | 6.210738182 |
| TRAF3IP2 | TRAF3 Interacting Protein 2 | GC06M111555 | 6.201737404 |
