# Supplementary material for: Prognostic and immunological roles of ammonia-induced cell death-related genes in non-small cell lung cancer
Source: BMC Pulm Med. 2026 Feb 21;26:138. doi: 10.1186/s12890-026-04181-7 (PMC13032429; doi:10.1186/s12890-026-04181-7)
Supplement: Supplementary file 1 — Supplementary Material 1. [file 12890_2026_4181_MOESM1_ESM.zip › Supplementary Table 3.docx]

**Table S3. Primers sequences used in this study**

| **Primers for PCR** | | |
| --- | --- | --- |
| Gene | Forward | Reverse |
| hSPP1 | GAAGTTTCGCAGACCTGACAT | GTATGCACCATTCAACTCCTCG |
| hSLC2A1 | TCTGGCATCAACGCTGTCTTC | CGATACCGGAGCCAATGGT |
| hSLC7A5 | CCGTGAACTGCTACAGCGT | CTTCCCGATCTGGACGAAGC |
| hCAV1 | GCGACCCTAAACACCTCAAC | ATGCCGTCAAAACTGTGTGTC |
| hGAPDH | GTCAAGGCTGAGAACGGGAA | AAATGAGCCCCAGCCTTCTC |
| **siRNA** | | |
| Gene | sense(5'-3') | antisense(5'-3') |
| hSPP1-1 | GAACGACUCUGAUGAUGUATT | UACAUCAUCAGAGUCGUUCTT |
| hSPP1-2 | GAGGUGAUAGUGUGGUUUATT | UAAACCACACUAUCACCUCTT |
| hSPP1-3 | AGAGUUCAAUUCCAGUUGATT | UCAACUGGAAUUGAACUCUTT |
| hSLC2A1-1 | CCACGAGCAUCUUCGAGAATT | UUCUCGAAGAUGCUCGUGGTT |
| hSLC2A1-2 | GCUUCAUCAUCGGUGUGUATT | UACACACCGAUGAUGAAGCTT |
| hSLC2A1-3 | GGCGGAAUUCAAUGCUGAUTT | AUCAGCAUUGAAUUCCGCCTT |
| hSLC7A5-1 | GGGUGAUGUGUCCAAUCUATT | UAGAUUGGACACAUCACCCTT |
| hSLC7A5-2 | GGAUCGAGCUGCUCAUCAUTT | AUGAUGAGCAGCUCGAUCCTT |
| hSLC7A5-3 | GGAAGGGUGAUGUGUCCAATT | UUGGACACAUCACCCUUCCTT |
| hCAV1-1 | GGUCAACCGCGACCCUAAATT | UUUAGGGUCGCGGUUGACCTT |
| hCAV1-2 | CGAUGACGUGGUCAAGAUUTT | AAUCUUGACCACGUCAUCGTT |
| hCAV1-3 | CCACCUUCACUGUGACGAATT | UUCGUCACAGUGAAGGUGGTT |

**h, Homo sapiens**
