# Supplementary material for: Prognostic and immunological roles of ammonia-induced cell death-related genes in non-small cell lung cancer
Source: BMC Pulm Med. 2026 Feb 21;26:138. doi: 10.1186/s12890-026-04181-7 (PMC13032429; doi:10.1186/s12890-026-04181-7)
Supplement: Supplementary file 1 — Supplementary Material 1. [file 12890_2026_4181_MOESM1_ESM.zip › Supplementary Table 4.docx]

| **Table S4. Univariate Logistic regression and multivariate Logistic regression** | | | | | |
| --- | --- | --- | --- | --- | --- |
| Characteristics | Total(N) | OR(95% CI) Univariate analysis | P value Univariate analysis | OR(95% CI) Multivariate analysis | P value Multivariate analysis |
| Age | 983 | 0.984 (0.970 – 0.997) | 0.019 | 0.978 (0.963 – 0.993) | 0.003 |
| T | 997 |  |  |  |  |
| T2 | 317 | Reference |  | Reference |  |
| T4 | 45 | 0.728 (0.389 – 1.362) | 0.321 | 0.823 (0.408 – 1.660) | 0.586 |
| T1 | 117 | 1.005 (0.657 – 1.539) | 0.981 | 0.873 (0.556 – 1.372) | 0.556 |
| T2a | 175 | 2.024 (1.365 – 3.001) | < 0.001 | 2.072 (1.373 – 3.127) | < 0.001 |
| T1b | 87 | 2.616 (1.527 – 4.483) | < 0.001 | 2.442 (1.392 – 4.283) | 0.002 |
| T3 | 113 | 0.818 (0.532 – 1.257) | 0.359 | 0.884 (0.564 – 1.386) | 0.591 |
| T1a | 72 | 1.387 (0.820 – 2.347) | 0.222 | 1.256 (0.721 – 2.188) | 0.421 |
| T2b | 70 | 2.809 (1.542 – 5.119) | < 0.001 | 3.153 (1.698 – 5.855) | < 0.001 |
| TX | 1 |  | 0.969 | 0.000 (0.000 – Inf) | 0.992 |
| N | 997 |  |  |  |  |
| N0 | 627 | Reference |  | Reference |  |
| N1 | 236 | 0.713 (0.526 – 0.966) | 0.029 | 0.761 (0.548 – 1.057) | 0.103 |
| N2 | 114 | 0.592 (0.396 – 0.884) | 0.010 | 0.625 (0.404 – 0.968) | 0.035 |
| NX | 13 | 0.914 (0.296 – 2.828) | 0.876 | 1.052 (0.279 – 3.967) | 0.941 |
| N3 | 7 |  | 0.967 | 3824081.7112 (0.000 – Inf) | 0.978 |
| M | 986 |  |  |  |  |
| M0 | 746 | Reference |  | Reference |  |
| M1 | 21 | 0.400 (0.164 – 0.978) | 0.044 | 0.492 (0.190 – 1.272) | 0.143 |
| MX | 211 | 1.087 (0.793 – 1.489) | 0.605 | 0.946 (0.675 – 1.327) | 0.750 |
| M1b | 5 | 0.434 (0.072 – 2.611) | 0.362 | 0.247 (0.036 – 1.681) | 0.153 |
| M1a | 3 | 0.325 (0.029 – 3.603) | 0.360 | 0.315 (0.025 – 3.993) | 0.373 |
| Smoking | 411 |  |  |  |  |
| No | 266 | Reference |  |  |  |
| Yes | 145 | 1.277 (0.835 – 1.953) | 0.259 |  |  |
| Gender | 997 |  |  |  |  |
| male | 619 | Reference |  |  |  |
| female | 378 | 1.183 (0.909 – 1.539) | 0.211 |  |  |
| riskScore | 997 | 0.081 (0.029 – 0.225) | < 0.001 | 0.093 (0.032 – 0.272) | < 0.001 |
