# Supplementary material for: Prognostic and immunological roles of ammonia-induced cell death-related genes in non-small cell lung cancer
Source: BMC Pulm Med. 2026 Feb 21;26:138. doi: 10.1186/s12890-026-04181-7 (PMC13032429; doi:10.1186/s12890-026-04181-7)
Supplement: Supplementary file 2 — Supplementary Material 2. [file 12890_2026_4181_MOESM2_ESM.docx]

**Supplementary Materials for**

**Prognostic and immunological roles of ammonia-induced cell death-related genes in non-small cell lung cancer**

Hongbin Li, Kai Xue, Jianying Pei, Xiaoli Ma, Xueshan Zhao, Chong Zhang

**This file includes:**

Supplementary Figures 1-7 (pp2-10)

**Supplementary Figures**

**Supp Figure 1**
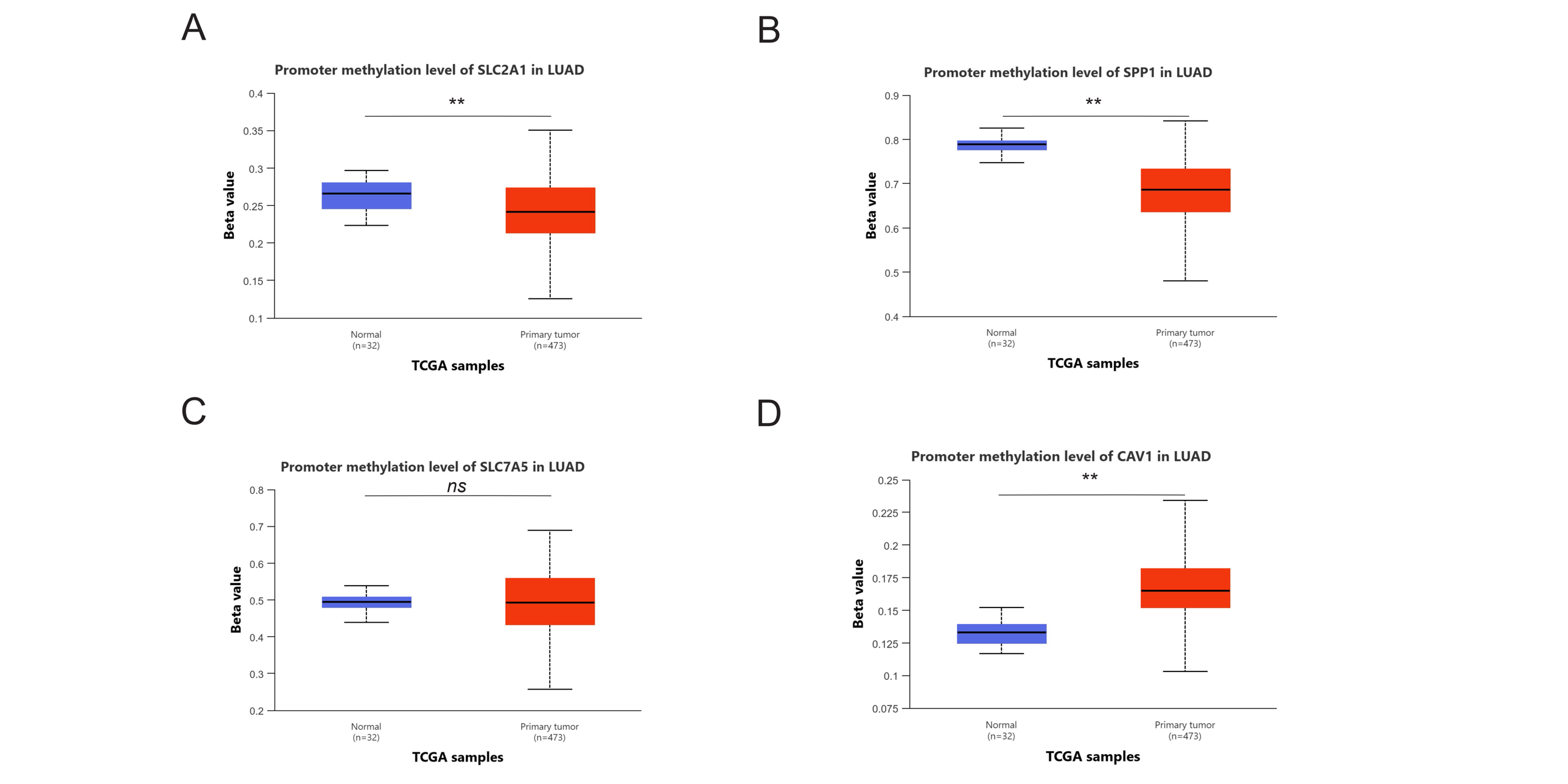


**Supp Figure 1. Promoter methylation profiles of four key *ADRGs* in NSCLC.**

**
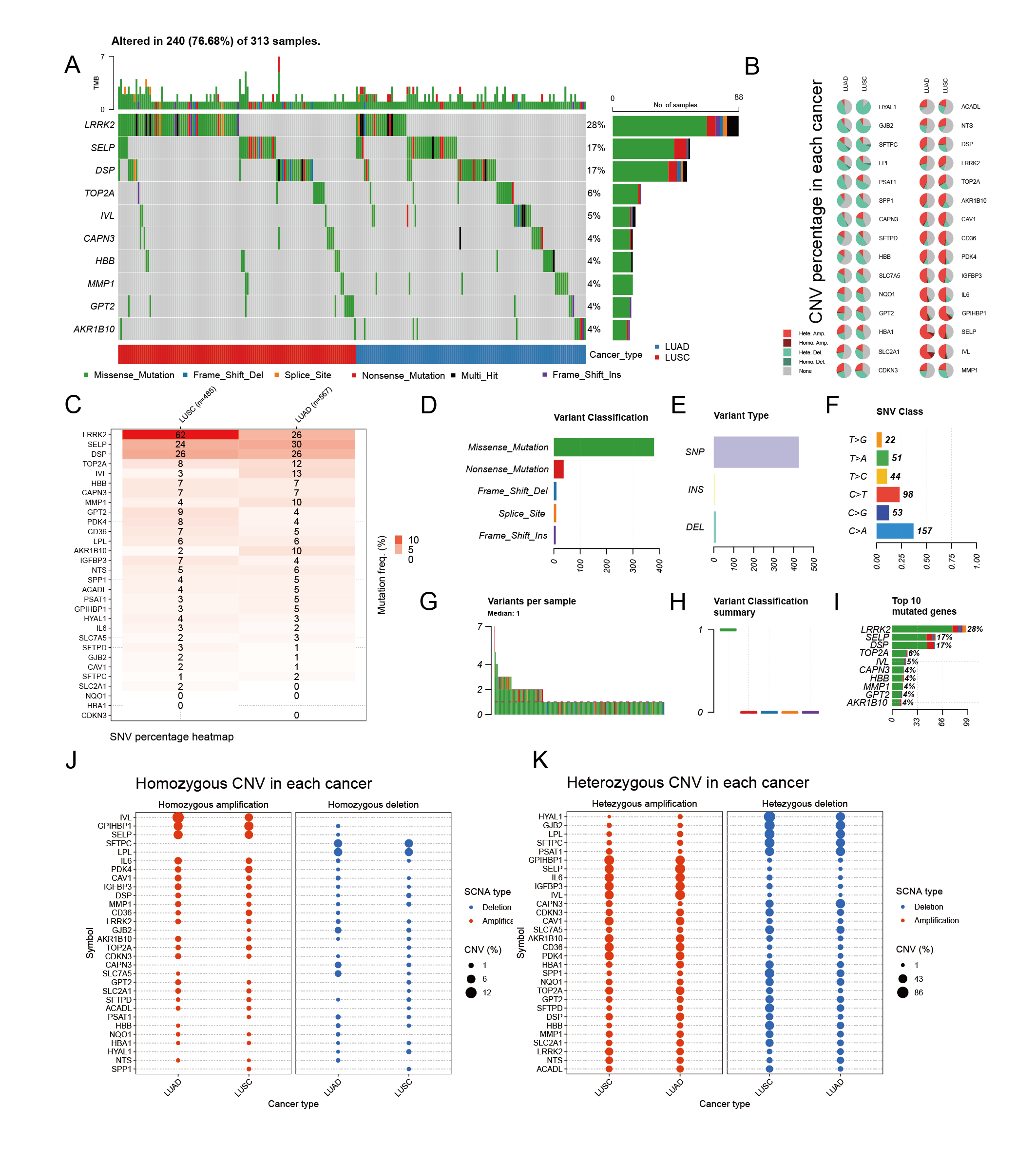
Supp** **Figure 2**

**Supp Figure 2. Genetic alterations in *ADRG* across NSCLC cohorts.** (A) OncoPrint summarizes somatic mutations in 30 *ADRGs* across 313 NSCLC samples (76.68% altered), stratified by LUAD and LUSC, with mutation types color-coded. (B) Pie chart of mutation frequencies (%) for 30 *ADRGs*. (C) Heatmap of single-nucleotide variant (SNV) percentages in 30 *ADRGs* for LUAD and LUSC. (D–F) Bar plots of variant classification, variant type, and SNV class distributions. (G) Bar plot of variants per sample (median: 1). (H) Bar plot summarizing variant classification frequencies. (I) Stacked bar chart of the top 10 mutated genes by frequency. (J, K) Bubble charts of heterozygous and homozygous copy number variations (CNVs) in 30 *ADRGs*, stratified by LUAD/LUSC and alteration type (amplification/deletion).

**
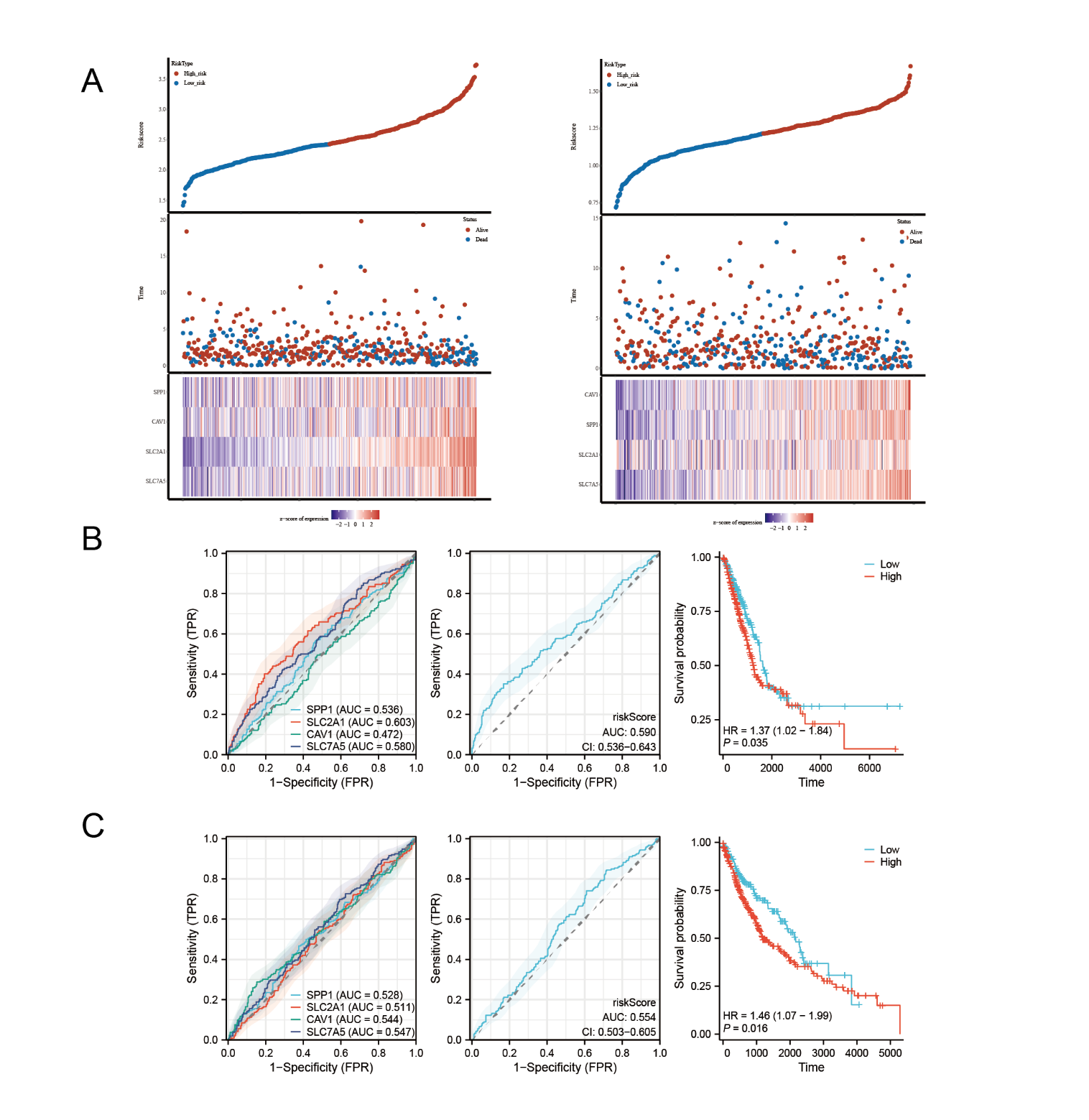
Supp Figure 3**

**Supp Figure 3.** **Model revalidation using LUSC and LUAD TCGA data.** (A) Dot plots illustrating the distribution of risk scores in high-risk (red dots) and low-risk (blue dots) patient groups. Risk scores were significantly higher in the high-risk group compared with the low-risk group. The left panel represents the TCGA-LUAD cohort, and the right panel represents the TCGA-LUSC cohort. (B–C) Kaplan–Meier survival analysis and receiver operating characteristic (ROC) curve analysis were performed based on the risk score and the expression levels of four key genes in the TCGA-LUAD cohort(B) and LUSC cohort (C).

**Supp Figure 4**





**Supp Figure 4.** **Associations between *ADRG* risk signature and clinicopathological features in NSCLC.** (A) Violin plots of SLC2A1, SLC7A5, SPP1, and CAV1 expression across pathologic T stages (***P < 0.001, **P < 0.01, *P < 0.05). (B) Stacked bar plots showing risk group distributions across clinical variables (age, race, pN stage, pM stage, pTNM stage, new tumor event, smoking, radiation therapy, neoadjuvant treatment, therapy type). (C) Bar plots depict the distribution of high- and low-risk groups across clinical subgroups (e.g., age ≤67 vs. >67; P values indicated).

**
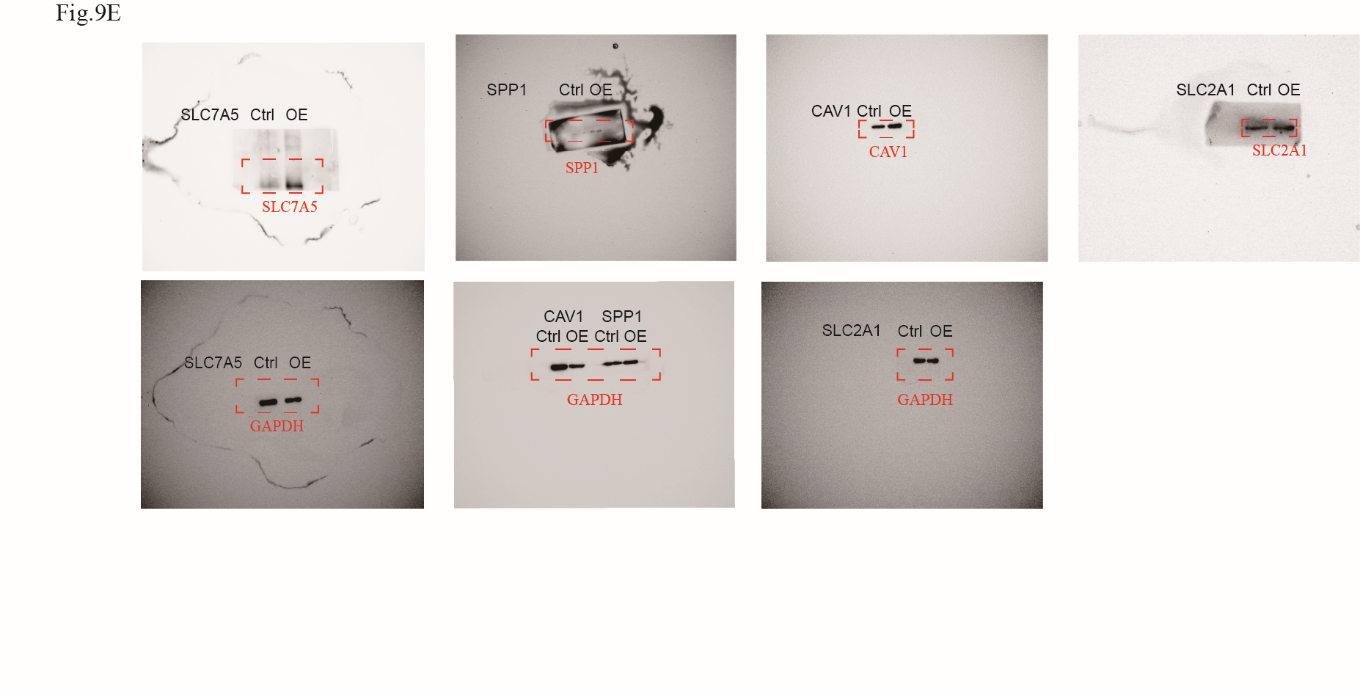
Supp Figure 5**


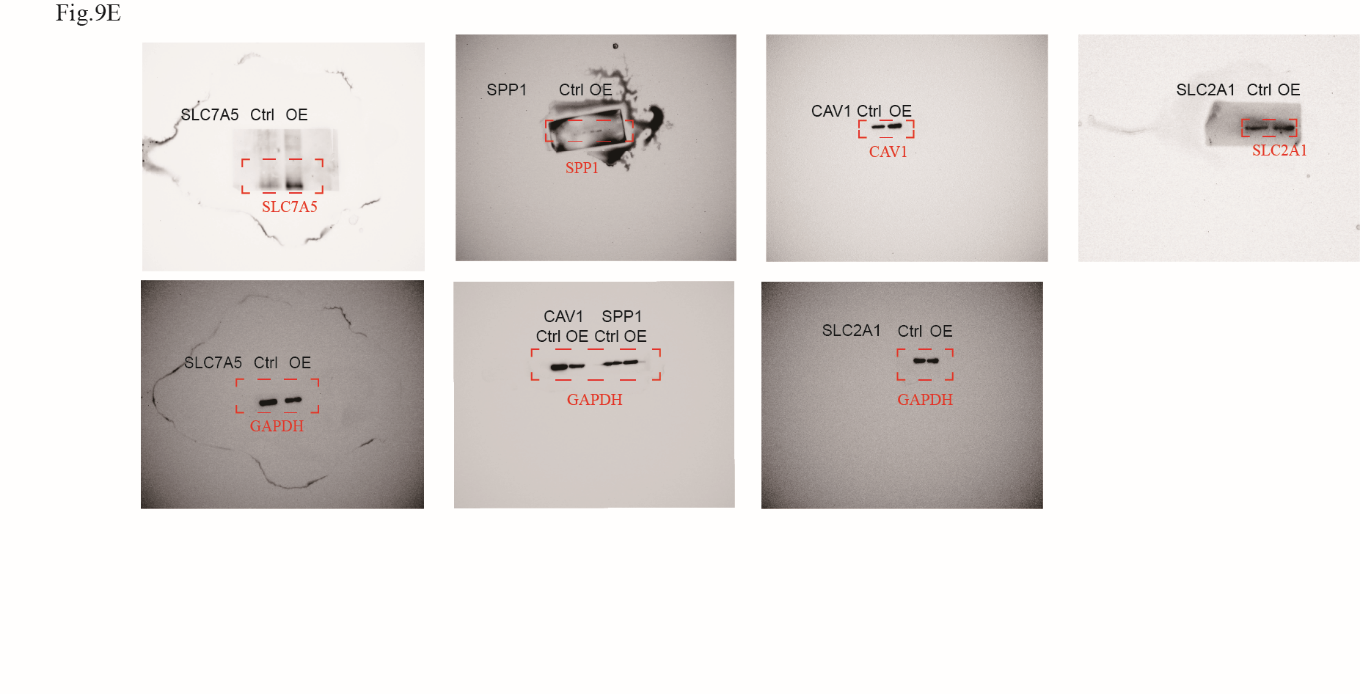

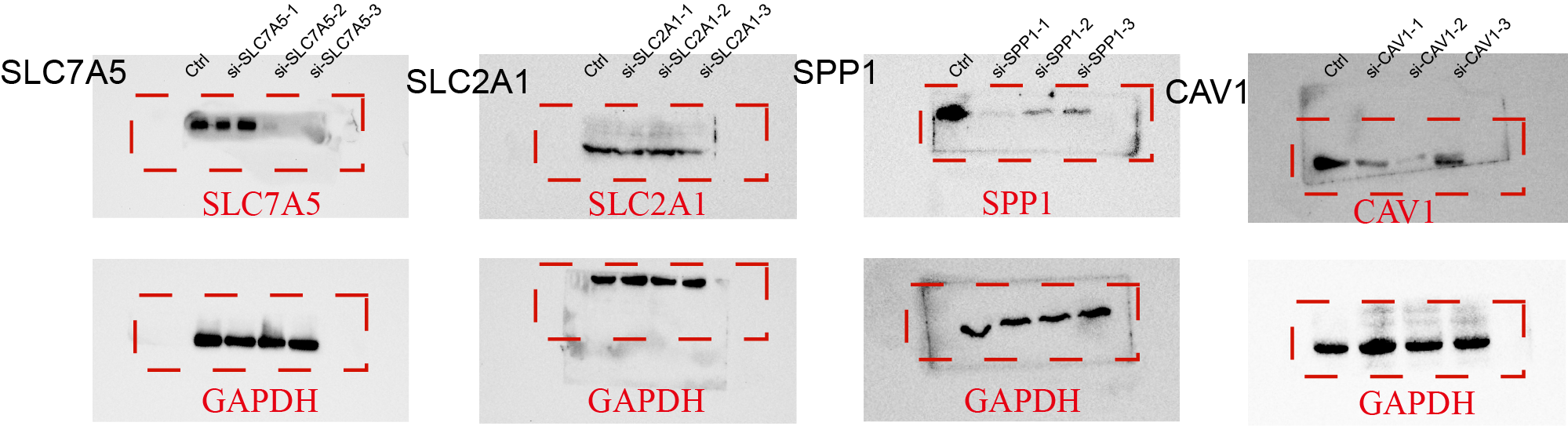


**Supp Figure 5. Figures depict Western blot (WB) results.** Quantification of WB band intensities is expressed as the ratio of target protein to GAPDH.

**
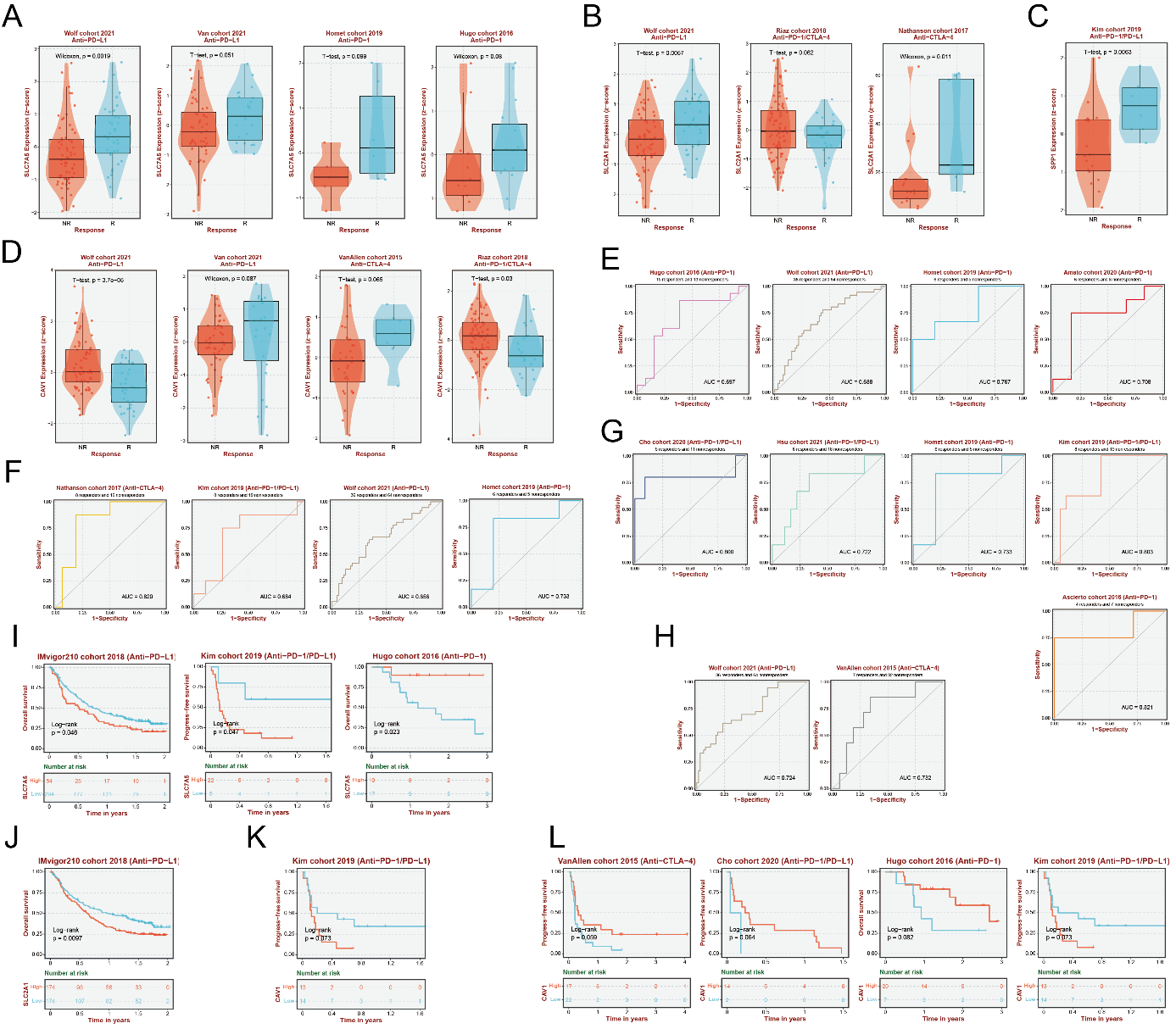
Supp Figure 6**

**Supp Figure 6. The correlation between the four-gene signature and immunotherapy response in clinical cohorts.** (A–D) Violin plots showing the expression levels of **SLC7A5**, **SLC2A1**, **SPP1**, and **CAV1** in patients with different immunotherapy response statuses. (E–H) ROC curves evaluating the ability of four genes (**SLC7A5, SLC2A1, SPP1, CAV1**) to predict response to immunotherapy. (I–L) Kaplan–Meier survival curves of immunotherapy-treated patients stratified by high and low expression of the four genes (**SLC7A5, SLC2A1, SPP1, CAV1**).


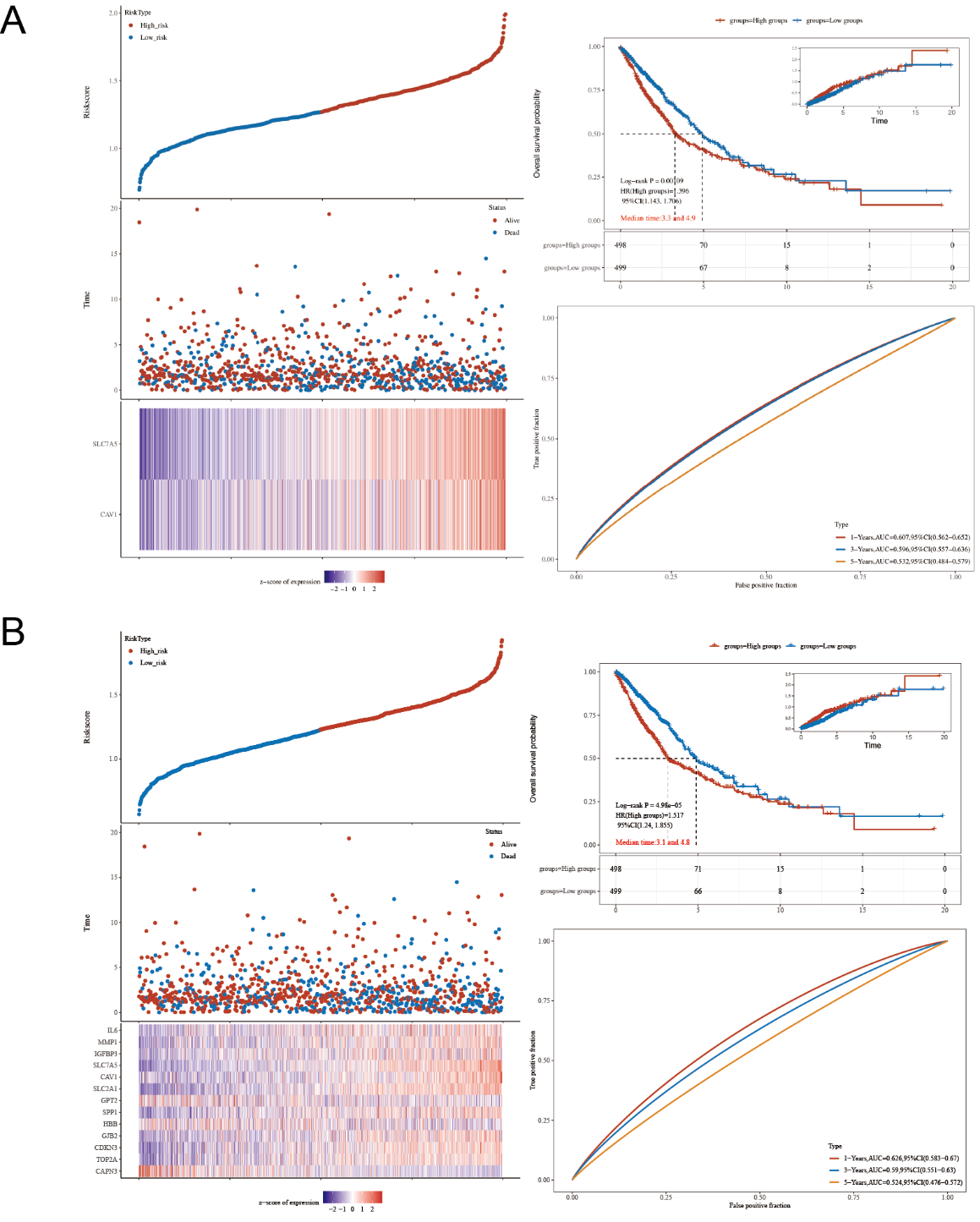
**Supp Figure 7**

**Supp Figure 7. Construction of prognostic models using multivariate Cox regression and stepwise regression analyses.** (A–B) Prognostic models were constructed in the TCGA NSCLC dataset using 13 favorable prognostic ADRGs based on multivariate Cox regression (A) and stepwise regression (B).
